# New TRPM8 Amino Acid-Based Antagonists Induce Antiproliferative Effect on 2D and 3D Melanoma and Prostate Cancer Models

**DOI:** 10.1021/acs.jmedchem.6c00479

**Published:** 2026-08-06

**Authors:** Tania Ciaglia, Marzia Di Donato, Veronica Di Sarno, Gerardina Smaldone, Sofia Pellecchia, Francesca Di Matteo, Valeria Napolitano, Eleonora Boccia, Carmela Sorrentino, Giacomo Pepe, Manuela Giovanna Basilicata, Carmine Lauretta, Giovanna Aquino, Pia Giovannelli, Isabel M. Gomez-Monterrey, Vincenzo Vestuto, Antimo Migliaccio, Pietro Campiglia, Gianluigi Lauro, Carmine Ostacolo, Giuseppe Bifulco, Gabriella Castoria, Antonio Ferrer-Montiel, Asia Fernández-Carvajal, Simona Musella, Alessia Bertamino

**Affiliations:** † Department of Pharmacy, 19028 University of Salerno, Via G. Paolo II 132, Fisciano, Salerno 84084, Italy; ‡ Department of Precision Medicine, 507855 University of Campania, ‘L. Vanvitelli’, Via L. De Crecchio 7, 80138, Naples, Italy; § Department of Advanced Medical and Surgical Sciences, University of Campania, “Luigi Vanvitelli” P.zza L. Miraglia 2, Naples 80138, Italy; ∥ AREA Science Park, Laboratorio di Multi-Omica Area Sud (LAAS), Baronissi, Salerno 84081, Italy; ⊥ Department of Pharmacy, School of Medicine and Surgery, University of Naples Federico II, Via D. Montesano 49, Naples 80131, Italy; # Instituto de Investigación, Desarrollo e Innovación en Biotecnología Sanitaria de Elche (IDiBE), Universidad Miguel Hernández de Elche, 16753 Avenida de la Universidad, Elche 03202, Spain

## Abstract

The pharmacological potential of TRPM8 modulators ranges from neuropathic pain to tumor treatment, as this channel is involved in intracellular calcium homeostasis. We acquired considerable expertise in developing TRPM8 antagonists, which were pharmacologically characterized for their analgesic properties and their antiproliferative activity in prostate cancer. Based on the structural knowledge gained in this field, we designed and synthesized a new series of TRPM8 blockers which was validated by calcium fluorimetry and electrophysiology experiments. *In silico* studies helped to rationalize compounds activity, improving the structural insights about this target. The most promising TRPM8 antagonists were evaluated in melanoma and prostate 2D and 3D cancer models. From this investigation, three compounds, **7**, **29,** and **34**, emerged as the most promising candidates; thus, they were further analyzed for their druggability by *in vitro* pharmacokinetic experiments. This study revealed compound **7** as the most chemically and metabolically stable derivative, highlighting its suitability for further pharmacological evaluation.

## Graphical abstract

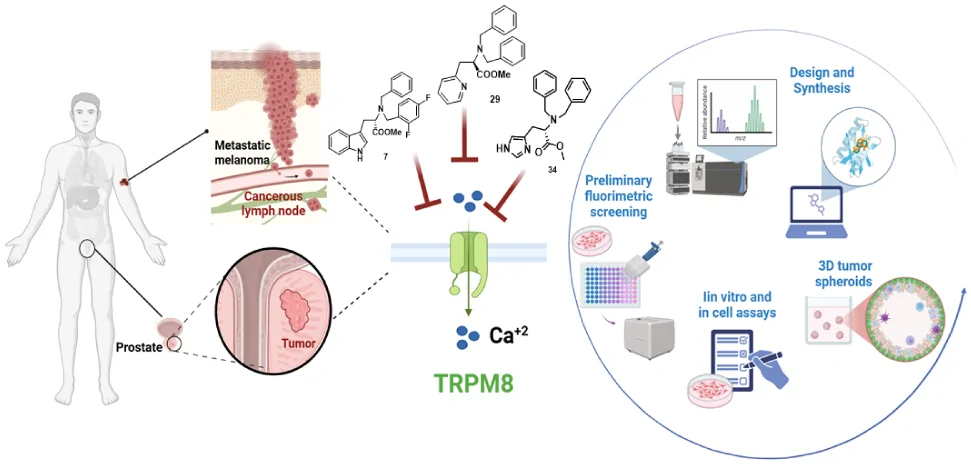

## Introduction

The transient receptor potential melastatin member 8 (TRPM8) channel, a polymodal calcium-permeable ion channel, is a therapeutically compelling target for several pathophysiological conditions.^1^ TRPM8 is predominantly expressed in sensory neurons of dorsal root and trigeminal ganglia, where it functions as a cold thermoreceptor activated by temperatures below 25–28 °C, as well as by cooling agents such as menthol and icilin.^2^ In addition to its neuronal localization, TRPM8 is widely distributed in peripheral tissues including the urogenital tract, respiratory and gastrointestinal epithelia, mucosal tissues, immune cells, and vascular smooth muscle cells.^3–5^ Beyond thermosensation, TRPM8 activity is modulated by osmotic and electrical stimuli and critically depends on phosphatidylinositol 4,5-bisphosphate (PIP_2_) availability at the plasma membrane.^6–7^

The fundamental role of TRPM8 in calcium homeostasis positions it as a critical regulator in both physiological and pathological processes.

TRPM8 was initially characterized for its role in cold pain transduction,^8^ work that contributed to the discoveries recognized by the 2021 Nobel Prize awarded to Julius and Patapoutian.^9,10^ However, different investigations have revealed its involvement in diverse biological functions including bladder contractility,^11^ prostate functions and sperm motility,^12–14^ airway responses to cold stimuli,^15,16^ immune response modulation,^17,18^ ocular surface wetness and tear flow,^19,20^ as well as melanin synthesis in melanocytes.^21^ Growing evidence demonstrates that TRPM8 dysregulation contributes to malignant transformation and cancer progression through mechanisms encompassing uncontrolled cellular proliferation, enhanced invasiveness, and acquired chemotherapy resistance.^22–24^ TRPM8 overexpression has been reported in multiple human malignancies. Although initially identified in androgen-dependent prostate cancer,^25,26^ subsequent studies established TRPM8 up-regulation in breast,^27^ osteosarcoma,^28^ pancreatic,^29^ lung,^30^ melanoma^31^ and several additional cancer types. This widespread dysregulation suggests that TRPM8 may represent a common vulnerability across distinct cancer contexts and establishes TRPM8 as a relevant prognostic and diagnostic biomarker, with clinical implications in prostate and cutaneous cancers.^32^ These observations support TRPM8 as a promising molecular target for anticancer drug discovery.

Therapeutic interest in TRPM8 has stimulated the development of selective channel modulators, an effort greatly facilitated by advances in structural biology. Early antagonists were disclosed using homology models derived from avian TRPM8 orthologs, *Ficedula albicollis* (FaTRPM8) and *Parus major* (PmTRPM8).^33–35^ A breakthrough occurred in 2019 with the resolution of TRPM8 cryo-electron microscopy structures in multiple functional states including ligand-free, antagonist-bound, and calcium-bound conformations, providing unprecedented molecular detail to drive subsequent antagonist optimization.^35^ Structural knowledge of the human TRPM8 channel^36^ will further enhance the discovery and development of selective TRPM8 antagonists as potential disease-modifying agents, offering new mechanistic approaches to disrupting oncogenic calcium signaling pathways.

Despite these advances, only a limited number of TRPM8 modulators have progressed to clinical evaluation: PF-05105679 and AMG333, developed by Pfizer and Amgen for neuropathic pain and migraine, respectively, have progressed to clinical trials, while only WS-12 (Acoltremon), advanced by Alcon Laboratories, has been approved for treating symptoms of dry eye disease (Figure 1).^37–39^ However, except for WS-12, most clinical candidates were discontinued due to limited selectivity, side effects, or pharmacokinetic limitations, highlighting the need for TRPM8 modulators with improved pharmacological and drug-like properties to support further clinical development.

**Figure 1:**
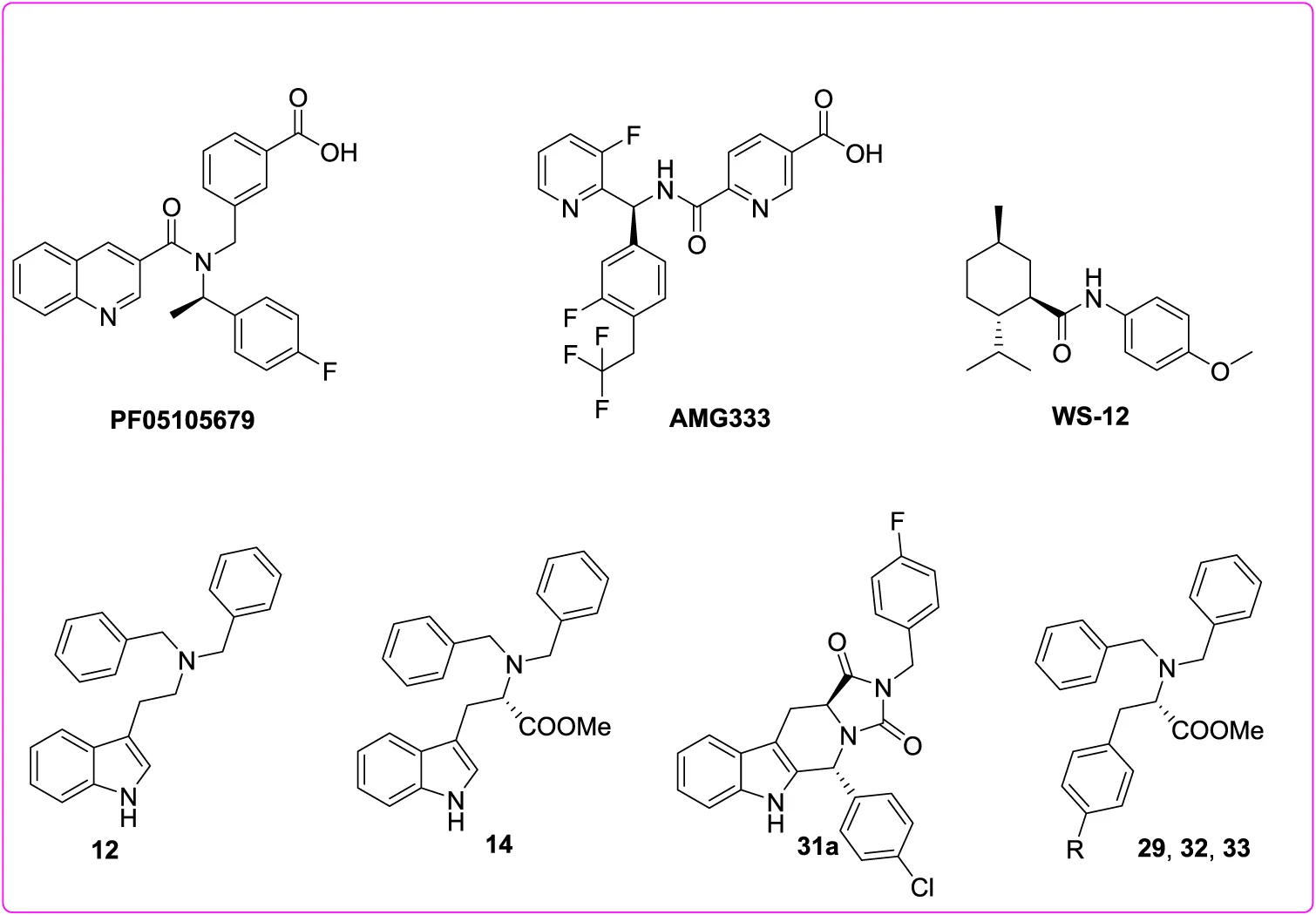
Chemical structure of TRPM8 reference antagonists and our derivatives.

For over ten years, our research has focused on developing novel and selective TRPM8 antagonists. We successfully identified several compounds exhibiting strong inhibitory activity against the TRPM8 channel. Among these, a potent tryptamine-based compound (**12,** Figure 1) was evaluated *in vivo* as an analgesic agent.^8^ Building on this, we optimized related structures and discovered a tryptophan-derived compound (**14,** Figure 1), which was evaluated in *in vivo* models of icilin-induced WDS and oxaliplatin-induced cold allodynia in mice.^40^ To address pharmacokinetic limitations of compound **14**, we utilized a scaffold hopping strategy to synthesize tetrahydro-β-carboline derivatives, including compound **31a** (Figure 1), which demonstrated both analgesic efficacy *in vivo* and improved chemical and metabolic stability.^41^

Further exploring TRPM8 as a pharmacological target, we selected the most promising antagonists to test in androgen-dependent prostate cancer models, assessing the cellular mechanisms underlying their antitumoral effects.^42^ This work inspired a subsequent medicinal chemistry program that yielded a new series of phenylalanine-based compounds (**29**, **32**, **33**; Figure 1), specifically evaluated in prostate cancer 3D models.^43^

More recently, we expanded our investigation of TRPM8 antagonism to metastatic melanoma. Our findings revealed that TRPM8 blockade induces cancer cell death through a calcium-independent mitochondrial apoptotic pathway involving mitochondrial membrane depolarization, cytochrome c release, caspase-3 activation and engagement of the ATM/p53 signaling axis. In parallel, TRPM8 inhibition enhanced the expression of the natural killer (NK) cell-activating ligand ULBP1, increasing melanoma cells’ susceptibility to immune-mediated cytotoxicity.^44^

Despite these promising findings, the anticancer potential of TRPM8 antagonists remains underexplored, particularly with respect to chemically diverse scaffolds, tumor selectivity, and their validation in advanced cancer models.

Building on our extensive expertise in the development of TRPM8 antagonists and recent advances in cancer research, we designed a new series of TRPM8 through a ligand-based approach to obtain compounds with potential anticancer properties. Starting from compound **14,** our previously validated lead TRPM8 antagonist, we initiated this project with two main objectives. First, to broaden the chemical diversity of this class by modifying both the molecular scaffold and side chains of compound **14** to better explore the TRPM8 binding pocket (Figure 2). Second, to validate the new series of compounds in both 2D and 3D models of melanoma and prostate cancer. We considered modifying the 3-substituted indole scaffold with other electron-rich heterocyclic moieties such as imidazole, thiophene, pyridine as well as 2- or 5-substituted indoles. We also modified the methyl ester function with bulkier ester groups or different amide substitution to reduce the methyl ester metabolic liability. Concerning the amine side chains, we introduced differently decorated benzyl groups to explore the occupancy of the TRPM8 binding pocket. This integrated medicinal chemistry and pharmacological approach aims to advance TRPM8 antagonists as promising candidates for anticancer drug development.

**Figure 2:**
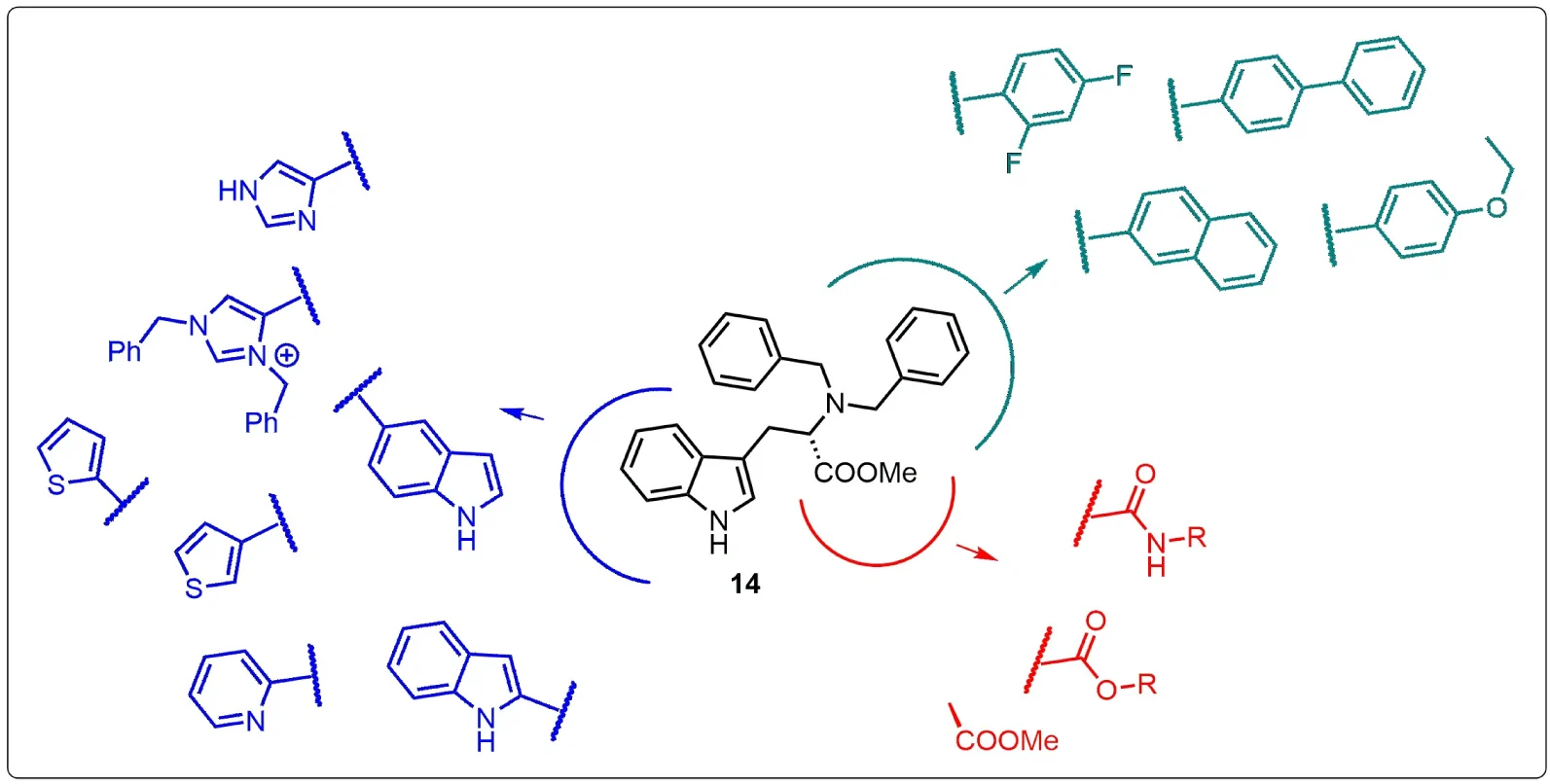
Design scheme of new TRPM8 antagonists.

## Results and Discussion

### Chemistry

Derivatives **3, 5–10**, **12** and **13** were synthesized as depicted in Scheme 1. *L*-Tryptophan methyl ester, *L*-tryptophan ethyl ester or *L*-tryptophan benzyl ester was reacted with benzaldehyde under reductive amination conditions leading to intermediates and**1**-**3** in 65–75% yield. *L*-Tryptophan methyl ester was also reacted with 4-ethoxybenzaldehyde in the same conditions described above furnishing **4** in 69% yield. Microwave-assisted alkylation of **1–4** in basic medium using 4-phenylbenzyl bromide (**5**), 2-(bromomethyl)­naphthalene (**6**), 2,4-difluorobenzyl bromide (**7**, **10**) and benzyl bromide (**8**, **9**) yielded the final compounds **5**-**10** in 48–67% yield. Compound **8** was further hydrolyzed under basic conditions to the carboxylic acid **11** in almost quantitively yield and then coupled with methylamine or butylamine leading to the final derivatives **12** and **13** in 46 and 55% yield, respectively.

**Scheme 1:**
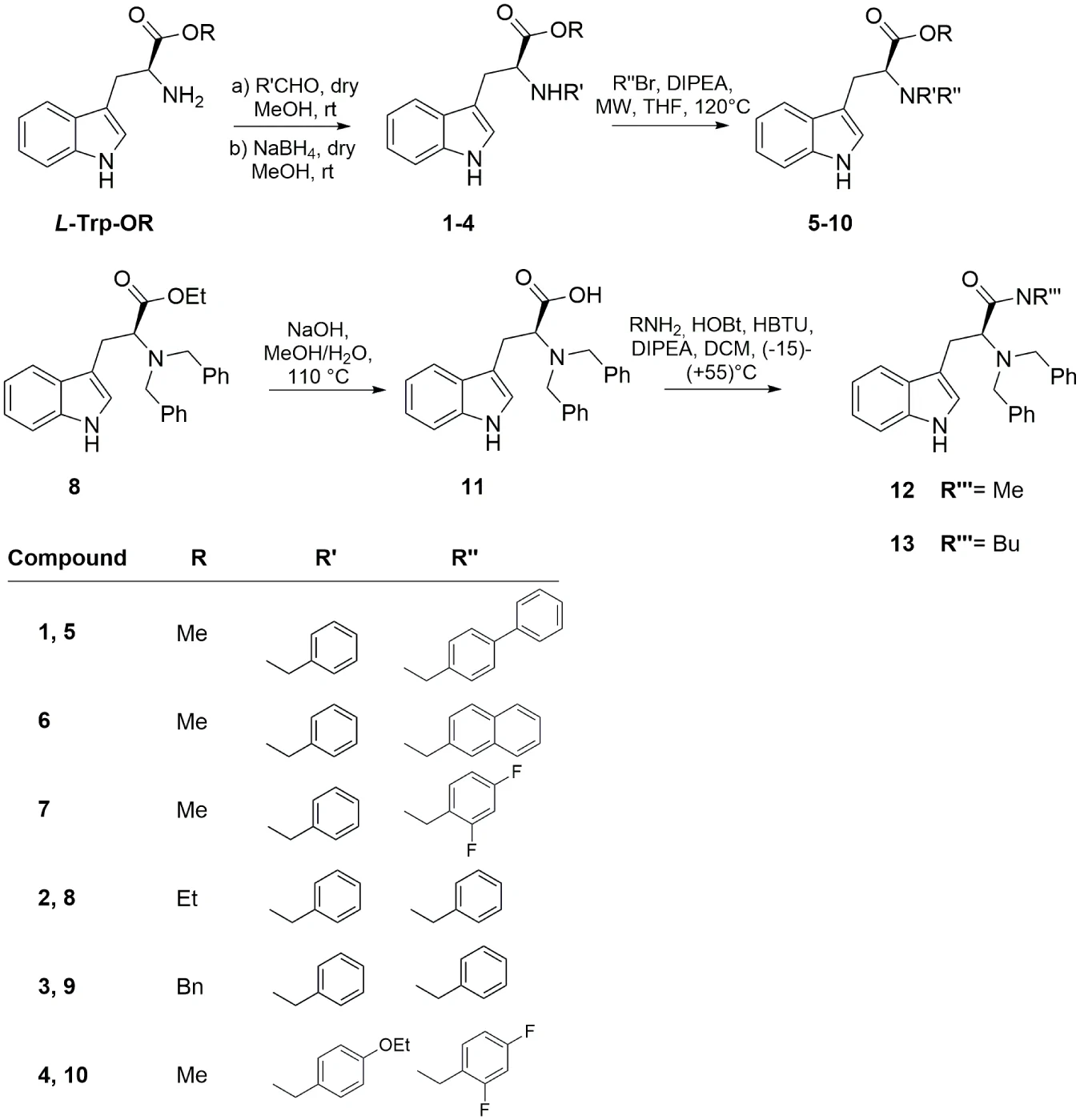
Synthesis of Derivatives **3**, **5–10**, **12**, **13**

The synthesis of compounds **23**–**32** was performed as shown in Scheme 2. N-(*tert*-Butoxycarbonyl)-*L*-serine methyl ester or N-(*tert*-butoxycarbonyl)-*D*-serine methyl ester was converted into the corresponding iodide **14a** or **14b** (89% and 92% yield, respectively) in the presence of iodine, triphenylphosphine, and imidazole.^45^ **14a** or **14b** was reacted with activated zinc under anhydrous conditions, furnishing quantitatively the organozinc derivative **15a** or **15b**, respectively, which was used without any purification step. For the Negishi coupling transmetalation Pd_2_(dba)_3_, XPhos, and the appropriate aryl bromide were used leading to the non-natural intermediates **16**–**22** in 43–54% yield. The further deprotection reaction in acid medium followed by microwave-assisted alkylation with benzyl bromide gave the final derivatives **23**–**29** (47–50% yield). Moreover, **23**, **26** and **28** were subjected to the ester hydrolysis followed by coupling with methylamine at −15 °C, yielding **30**–**32** in 40–45% yield.

**Scheme 2:**
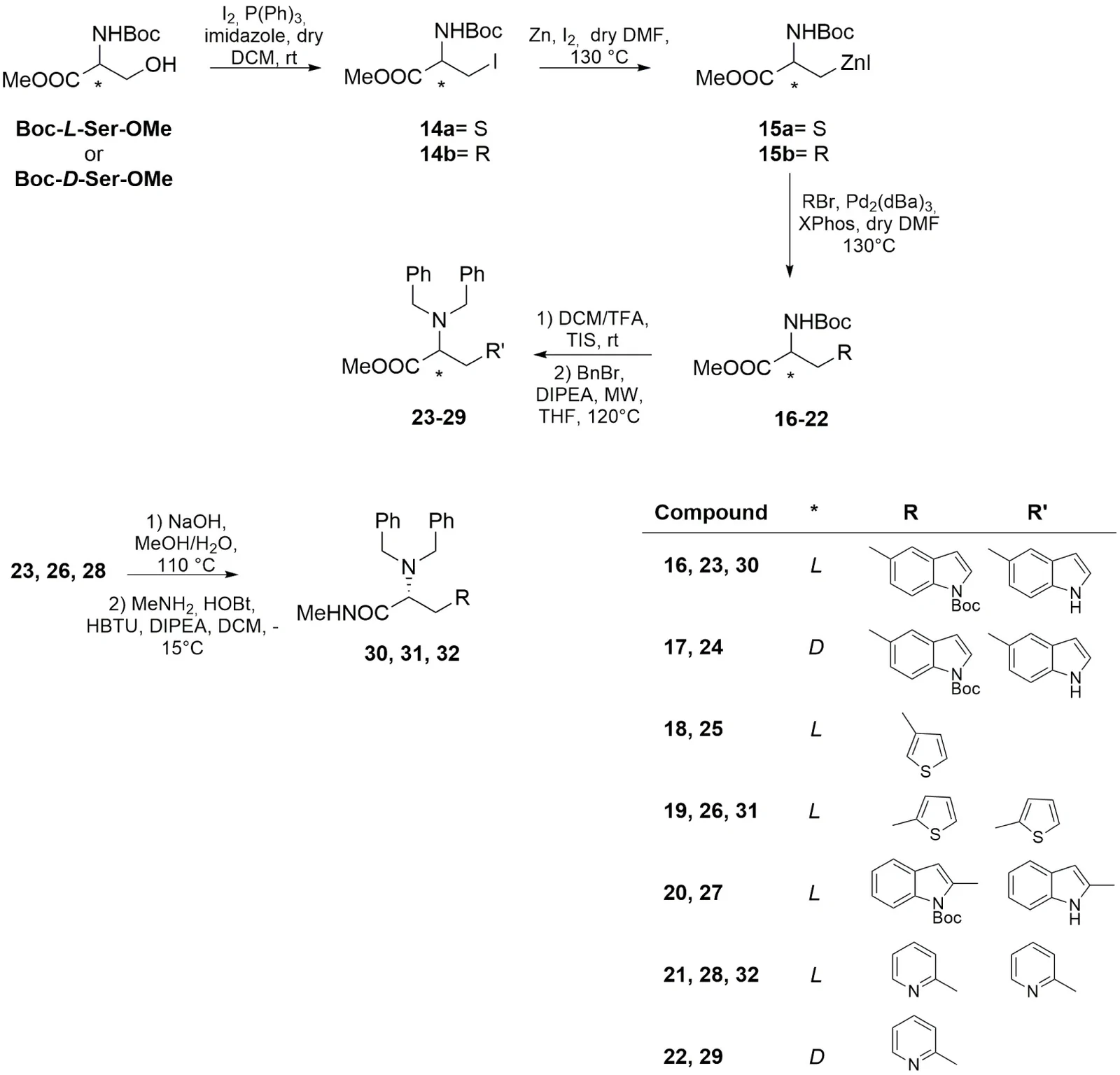
Synthesis of Derivatives **23–32.**

Final compounds **34** and **35** were obtained as reported in Scheme 3. *L*-Histidine methyl ester was derivatized at the α-NH_2_ group by a reductive amination reaction with benzaldehyde, giving **33** (61% yield). The subsequent alkylation reaction with benzyl bromide under the previously reported conditions led to the dibenzyl compound **34** and tetrabenzyl compound **35**, in 23% and 28% yield, respectively.

**Scheme 3:**
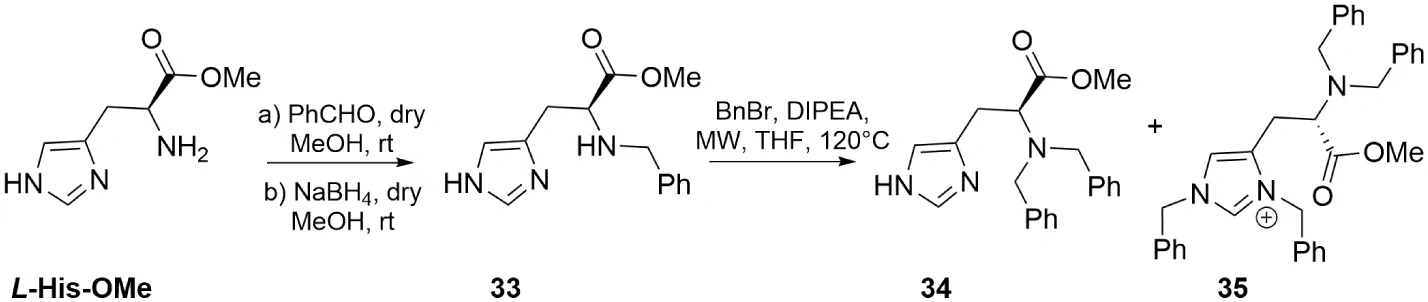
Synthesis of Derivatives **34** and **35.**

### Pharmacological Characterization by Ca^2+^-Microfluorimetric Assay

All synthesized derivatives were initially screened by Ca^2+^ microfluorimetric assay to evaluate their inhibitory activity on TRPM8 mediated responses in HEK293 cells stably expressing the channel. Menthol was used as agonist and AMTB as reference antagonist. The IC_50_ values obtained from these experiments are summarized in Table 1, revealing a subset of compounds with sub micromolar or low micromolar potency against TRPM8. Among them, several indole-based derivatives (e.g., **3**, **7**, **8**, **10**, **12**, **23**) and non-indole analogues bearing thiophene, pyridine or imidazole cores (**25**, **26**, **28**, **29**, **31**, **34**) displayed particularly favorable IC_50_ values. As IC_50_ values in Table 1 are reported as the mean ± SD of independent biological experiments performed on different days, the relatively large SD observed for some compounds (e.g., **7**, **10**, **23**, **26**) reflects inter experiment variability inherent to imaging based functional assays, rather than poor definition or instability of the dose–response relationship within individual experiments. Some representative curves of the most promising derivatives (**7**, **29** and **34**) are illustrated in Figure S64.

**Table 1:**
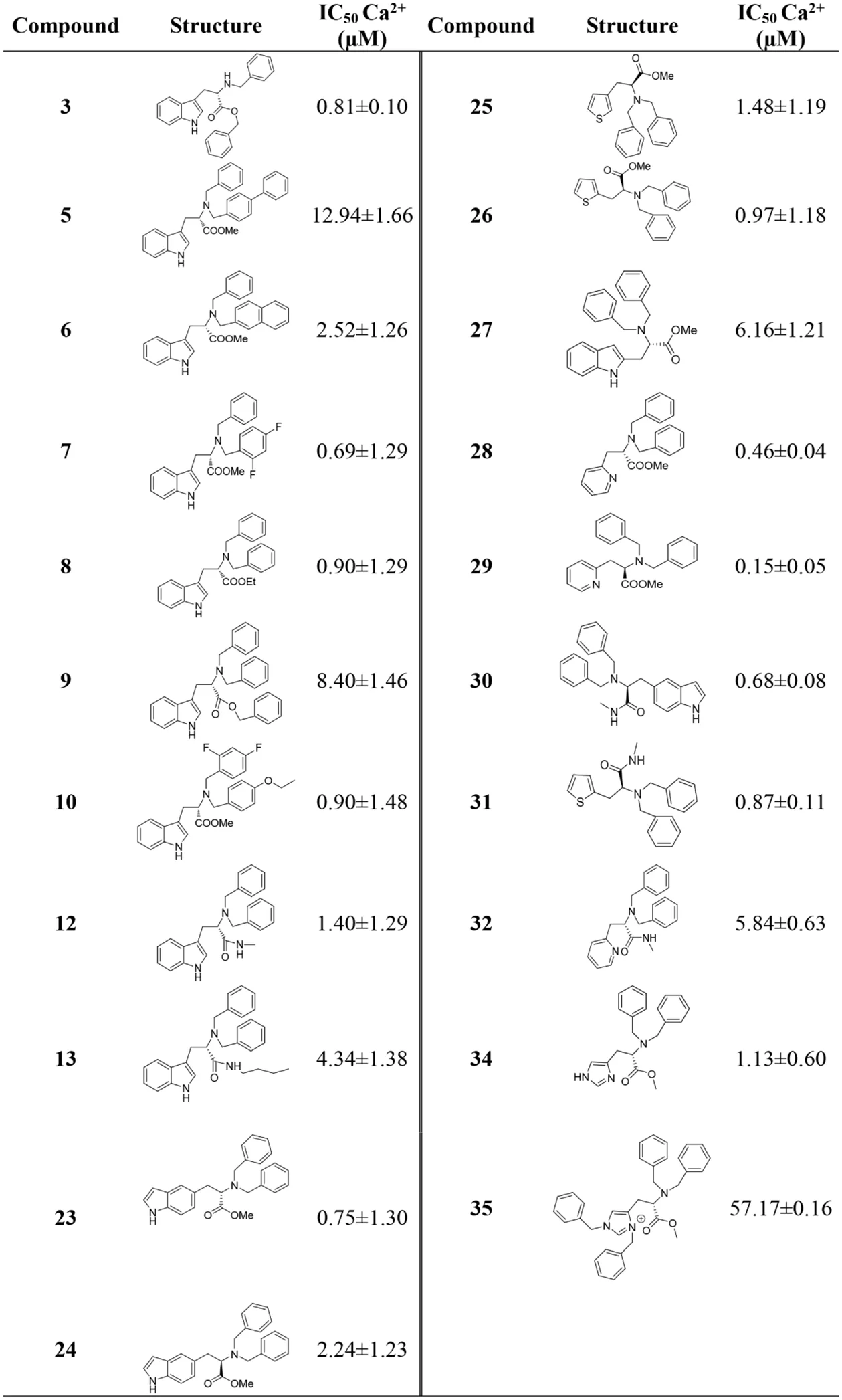
IC_50_ (μM) of Synthesized Compounds in Calcium Fluorimetric Assay

### Molecular Modeling Studies

Based on calcium fluorimetry screening, which identified several compounds displaying submicromolar or low-micromolar inhibitory activity against TRPM8 (Table 1), molecular docking calculations were performed using the TRPM8 electron microscopy (EM) structure (PDB: 6O72), released by Diver et al. in 2019 ^35^ to investigate the potential interactions underlying channel antagonism.

This protein structure, in complex with a known antagonist (**TC-I 2014**), provided a reliable framework for exploring the molecular bases responsible for ligand recognition and channel interference. The *in silico* analysis involved the entire series of synthesized compounds, mainly characterized by the presence of an indole core (**3**, **5–10**, **12**, **13**, **23, 24**, **27**, **30**), while a smaller subset of molecules incorporates thiophene (**25**, **26**, **31**), pyridine (**28**, **29**, **32**), and imidazole (**34**, **35**) moieties (Table 1). Docking analysis, as reported in Figure 3, revealed that all ligands occupy the same hydrophobic cavity defined by the transmembrane helices of TRPM8, corresponding to the binding pocket of the known antagonist **TC-I 2014**, as well as to that of other series of TRPM8 antagonists. The analysis of the putative binding modes was performed considering the ability of the compounds to engage key residues (e.g., Phe729, Asn790, Arg832) (Table S3, Supporting Information).^35,43^

**Figure 3:**
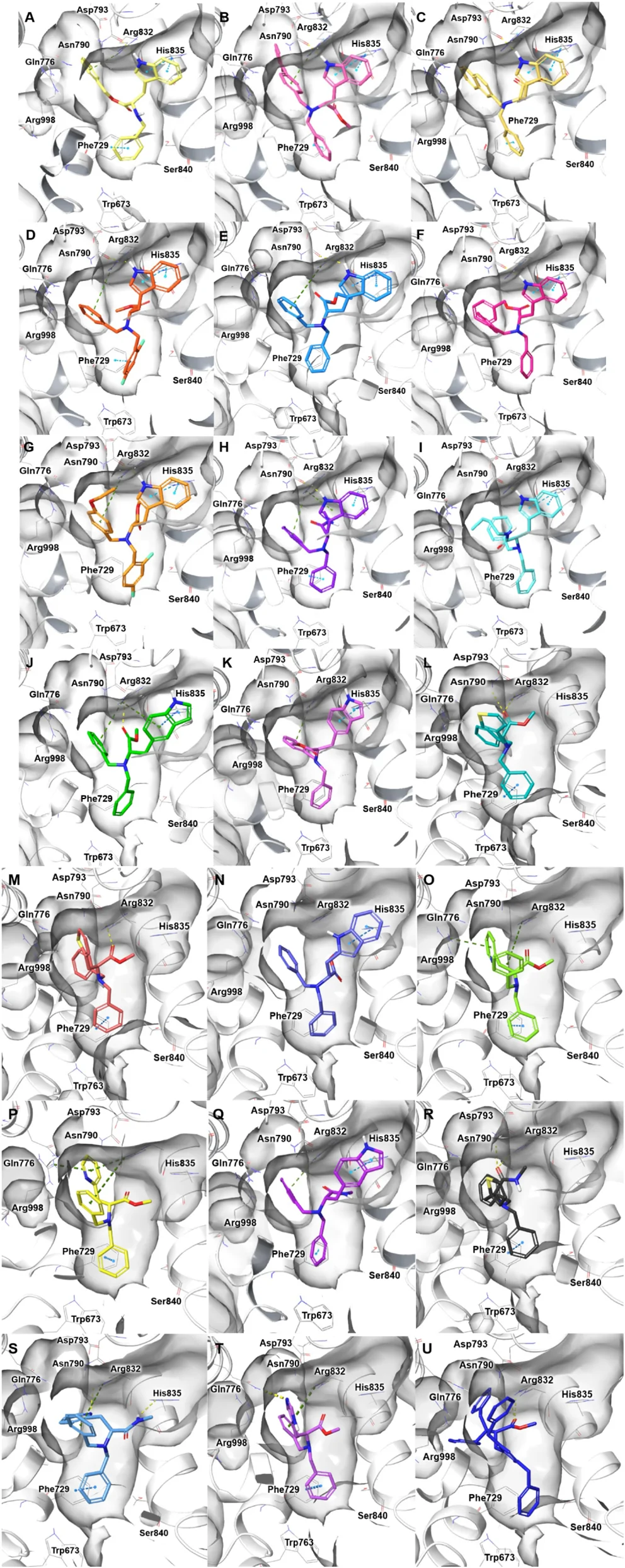
Binding mode of: A) **3** (colored by atom type: C faded yellow), B) **5** (colored by atom type: C faded salmon), C) **6** (colored by atom type: C faded orange), D) **7** (colored by atom type: C orange red), E) **8** (colored by atom type: C azure), F) **9** (colored by atom type: C salmon pink), G) **10** (colored by atom type: C orange), H) **12** (colored by atom type: C violet), I) **13** (colored by atom type: C cyan), J) **23** (colored by atom type: C green), K) **24** (colored by atom type: C faded magenta), L) **25** (colored by atom type: C teal), M) **26** (colored by atom type: C faded red), N) **27** (colored by atom type: C grape), O) **28** (colored by atom type: C yellow green), P) **29** (colored by atom type: C yellow), Q) **30** (colored by atom type: C plum), R) **31** (colored by atom type: C dark gray), S) **32** (colored by atom type: C faded azure), T) **34** (colored by atom type: C faded plum), U) **35** (colored by atom type: C blue) (all the other atoms colored by atom type: O red, N blue, F light green, polar H white) in the TRPM8 binding site (in light gray). H bonds, π–π stacking and π-cation interactions are reported in yellow, light blue and green dotted lines, respectively.

Specifically, all indole-based compounds (**3**, **5–10**, **12**, **13**, **23, 24**, **27**, **30**, Table 1) displayed a conserved π-π stacking interaction with His835, which appears as one of the critical residues for modulating TRPM8 activity toward antagonism (Figure 3). Most of these derivatives also established an H bond with Arg832 through the indole nitrogen, further stabilizing the ligand within the binding pocket and ensuring stable ligand anchoring within the TRPM8 binding cavity. In addition to the conserved interactions previously mentioned, most of indole-based derivatives established further stabilizing contacts, namely π–cation interactions with Asn790 or Arg832 and/or π–π stacking with Phe729 mediated by the aromatic moieties.

Among this subset of compounds, **23**, **24**, and **27** featured a different attachment point of the indole ring if compared with the remaining compounds (C-2 or C-5 instead of C-3) on the backbone (Table 1, Scheme 1, and Scheme 2). Compound **23** (Figure 3J) exhibited an orientation of the indole ring opposite to that observed for compounds with indole moiety attached at C-3, thus positioning the nitrogen atom far from Arg832, thereby preventing H-bond formation (Figure 3). However, the loss of this interaction was counterbalanced by π–cation contacts with Arg832, which may contribute to the experimentally observed activity (IC_50_ = 0.75 ± 1.30 μM, Table 1). This interaction pattern was also observed for compound **24** (Table 1 and Figure 3K), in which the opposite configuration at the chiral center also leads to a different orientation of the methyl ester group within the cavity, which may provide a possible structural rationale for its lower potency. Regarding compound **27**, the different attachment point at the indole (C-2 instead of C-5) caused the loss of π-π stacking with Phe729 and π–cation with Arg832, which could contribute to the reduced activity experimentally observed (IC_50_ = 6.16 ± 1.21 μM).

Non-indole derivatives (**25**, **26**, **28**, **29**, **31**, **32** and **34**, Figure 3L, 3M, 3O, 3P, 3R–3T) showed a slightly different binding mode within the binding cavity, establishing a distinct interaction pattern. In particular, these ligands maintain key contacts with Phe729 and Arg832, while losing the direct aromatic stacking with His835. Among them, a subset of compounds (**25**, **26** and **31**, Figure 3L, 3M and 3R), featuring a thiophene ring in place of the indole core, was analyzed. Although **25**, **26** and **31** are able to bind within the same TRPM8 binding pocket as the indole analogues **3**, **5–10**, **12**, **13**, **23**, **24**, and **27–30**, these compounds adopt a different binding mode, with the thiophene oriented in a different region occupied by the indole core. Specifically, compounds **25**, **26**, and **31** (Figure 3L, 3M and 3R) form a π-cation and π-π interactions with Arg832 and Phe729, ensuring effective anchoring within the cavity, although the thiophene moiety does not participate in aromatic stacking with His835, which may slightly affect the inhibitory potencies (0.87 ≤ IC_50_ ≤ 1.48 μM). Similarly, compounds **28**, its enantiomer compound **29**, and **32** (Figure 3O, 3P and 3S), all characterized by a pyridine ring instead of the indole core, exhibited a binding mode highly similar to that of the thiophene derivatives. All ligands occupy the same binding region of the protein counterpart, maintaining the interactions with Arg832 and Phe729. These results suggest that the replacement of the indole core with smaller aromatic systems drives the ligand toward the left hydrophobic region of the pocket. Compound **34**, featuring an imidazole ring instead of the indole core, shares a similar orientation within the TRPM8 pocket, as illustrated in Figure 3T, lacking π–π interactions with His835 and instead stabilizing through π–π interactions with Arg832, maintaining an IC_50_ around 1 μM. Finally, compound **35**, characterized by a bulkier and more extended scaffold, occupies the same region as the other non-indole derivatives (Figure 3U), losing most of the stabilizing interactions and retaining only a H-bond with Arg832. This finding may provide a possible structural explanation for the observed loss of activity in the Ca^2+^ assay (IC_50_ = 57.17 ± 0.16 μM).

Overall, these results indicate that while indole-based ligands share a conserved pose centered around His835, replacement of the indole core with alternative heteroaromatic systems (e.g., thiophene, pyridine, or imidazole) induces a shift in the binding orientation toward the left region of the cavity. This rearrangement preserves effective interactions with Phe729 and Arg832, consistent with previously reported TRPM8 antagonists featuring similar structural frameworks.^43^

### In Cell Cytotoxicity Evaluation

Based on the results of the preliminary Ca^2+^-imaging screening, we established 2 μM as the IC_50_ threshold to select candidates for further investigation through in cell-based cytotoxicity assays. This cutoff was adopted to prioritize the most potent TRPM8 antagonists, in line with our previous experience and the potency requirements generally adopted for progression to subsequent pharmacological validation studies. According to this criterion, 13 compounds (**3**, **7**, **8**, **10**, **12**, **23**, **25**, **26**, **28**, **29**, **30**, **31** and **34**) were selected for antitumor activity assessment. Cytotoxicity was evaluated using the CCK-8 assay in different melanoma and prostate cancer cell lines, while normal prostatic epithelium PNT-2 cells and murine fibroblasts NIH3T3 were included as nontransformed controls. All tested cell lines expressed TRPM8, although at different levels, as confirmed by Western blotting (Figures S65–S70). Melanoma MEL-CAL and Malme-3M were selected as representative metastatic cell lines, whereas LNCaP and PC3 cells were used as metastatic prostate cancer models. Cell viability was assessed after 24 and 72 h of treatment, and IC_50_ values and cytotoxicity profiles are summarized in Table 2 and Figures S65–S70, respectively.

**Table 2:**
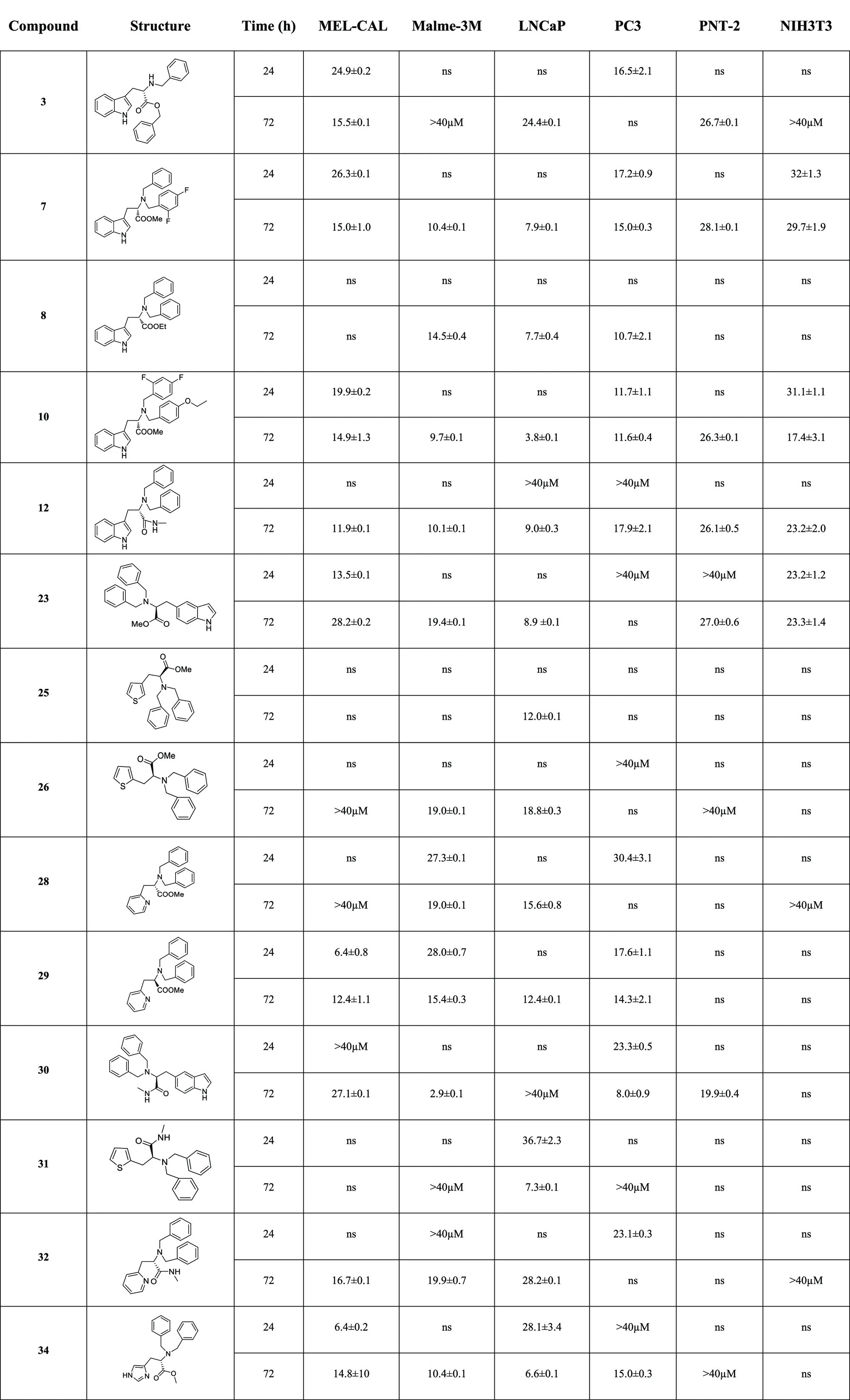
Overview of the Results of the *In Vitro* Antitumor Screening for the Synthesized Compounds^a^,^b^,^c^

a IC_50_ values are reported as mean ± SD and expressed in μM.

b ns: not significant.

c Data are expressed as IC_50_ values (μM) ± SD. Values >40 μM indicate that 50% inhibition was not achieved within the concentration range tested. ns (not significant) indicates that treatment did not produce a statistically significant reduction in cell viability relative to control cells; therefore, IC_50_ values were not determined.

In MEL-CAL cells (Table 2 and Figure S65), compounds **29** and **34** exhibited the strongest cytotoxic effects, with IC_50_ values of 6.4 ± 0.8 μM and 6.4 ± 0.2 μM at 24 h, respectively, and retained robust activity at 72 h. Compounds **7**, **10** and **12** also significantly reduced cell viability, particularly after prolonged exposure, indicating a time-dependent cytotoxic profile.

In Malme-3M cells (Table 2 and Figure S66), sensitivity to the selected derivatives was more heterogeneous. Compounds **7**, **10**, **12**, **30**, and **34** exhibited the highest cytotoxicity, with IC_50_ values ranging from 2.9 to 10.4 μM at 72 h. Most compounds (particularly compound **30**) showed increased cytotoxicity over time, again suggesting delayed but sustained cytotoxicity.

In LNCaP cells (Table 2 and Figure S67), several compounds (**7**, **8**, **10**, **12**, **34**) showed marked cytotoxicity with IC_50_ values below 10 μM at 72 h.

In PC3 cells (Table 2 and Figure S70), among the tested compounds **7, 8**, **10**, **29** and **30** exhibited the most relevant cytotoxic effects, with IC_50_ values below 15 μM at 72 h. However, compounds **7**, **8**, **12, 29**, **30** and **34** exhibited a time-dependent cytotoxic profile.

Importantly, nontransformed PNT-2 (Table 2 and Figure S68) and NIH3T3 cells (Table 2 and Figure S69) were largely insensitive to the majority of compounds, with IC_50_ values generally above the highest concentration tested (>40 μM) or associated with nonsignificant effects on cell viability (ns). This preferential activity toward cancer cells may reflect a differential dependency on TRPM8 signaling. Although TRPM8 is physiologically expressed in several tissues, its expression is frequently elevated in multiple malignancies, including melanoma and PC. Consistent with this concept, publicly available transcriptomic data sets revealed increased TRPM8 expression in several tumor types compared with corresponding normal tissues (Figure S78). Moreover, in our recent study on melanoma,^44^ TRPM8 antagonists induced robust antitumor responses while exerting negligible effects on normal melanocytes, HaCaT keratinocytes, human dermal fibroblasts and NIH3T3 fibroblasts, all characterized by low or barely detectable TRPM8 expression. In the same study, single-cell transcriptomic analyses demonstrated low TRPM8 expression across several nonmalignant cell populations within the melanoma microenvironment, including NK cells, whose viability was not affected by TRPM8 modulation. Together, these observations suggest that malignant cells may exhibit a greater dependency on TRPM8 signaling than their normal counterparts, providing a biological rationale for the preferential antitumor activity observed following TRPM8 inhibition. This tumor-selective cytotoxicity may reflect intrinsic differences in proliferative capacity or metabolic requirements between malignant and nonmalignant cells.

For contextualization of the biological activity reported herein, the IC_50_ values of compound **14**, a previously reported and biologically validated TRPM8 antagonist, are provided in Table S1 as a benchmark within the same target class.

Overall, compounds **10**, **12**, **29** and **34** displayed the broadest and most consistent antitumor activity across the melanoma and prostate cancer models investigated. In particular, compound **29** exhibited a rapid and sustained cytotoxic profile, retaining strong cytotoxicity already at 24 h and maintaining it at 72 h, whereas compounds **12** and **30** showed a more pronounced time-dependent effect.

Based on their overall biological profile, including antiproliferative activity, selectivity toward tumor cells, and suitability for further pharmacological evaluation, compounds **7**, **29** and **34** were selected as lead candidates for subsequent studies. Although the most active compounds displayed preferential activity toward tumor cells compared with nontransformed controls, the observed selectivity should be considered preliminary and will require further validation through dedicated pharmacological and *in vivo* studies.

In conclusion, as summarized in Table 2 and previously discussed, most compounds displayed micromolar cytotoxicity across melanoma and prostate cancer cells, with markedly reduced effects in nontransformed controls. Based on these results, compounds **7**, **8**, **10**, **12**, **23**, **25**, **29**, **30, 31** and **34** were selected for further biological characterization in 3D models. This selection was guided by their low IC_50_ values in at least one tumor model (MEL-CAL, Malme-3M, LNCaP or PC3) in 2D assays, together with their limited effects on PNT-2 and NIH3T3 cells. Notably, compounds **10**, **12**, **29**, and **34** showed the broadest and most consistent antitumor activity, whereas compounds **7**, **8**, **23**, **25**, **30** and **31** were retained because of their tumor-selective, time-dependent, or otherwise distinctive biological profiles. Collectively, this subset represents a mechanistically diverse panel of TRPM8 antagonists suitable for advanced biological characterization.

### The Effect of the Compounds in Melanoma- and PC-Derived 3D Models

To overcome the intrinsic limitations of 2D cultures, the antitumor activity of selected TRPM8 antagonists was further evaluated using 3D spheroid models embedded in extracellular matrix (ECM).

Melanoma- and prostate cancer-derived spheroids were generated in ECM as described in the Experimental Section and treated for 21 days with the selected compounds at the concentrations reported in Table S2 and in the corresponding figure legends. Compound concentrations were selected through a stepwise optimization strategy aimed at identifying the minimum effective concentration for each compound during long-term spheroid treatment. All compounds were initially tested at 5 μM. When no reproducible biological effect was observed, the concentration was increased to 10 μM and, if necessary, to 20 μM in subsequent independent spheroid experiments, in order to identify the minimum effective concentration under long-term 3D culture conditions.

After 21 days, phase-contrast and fluorescence microscopy revealed pronounced morphological and viability differences between untreated and treated spheroids (Figures 4, 5, 6 and 7). Untreated spheroids retained a compact architecture with strong Calcein-AM staining at the periphery and limited propidium iodide (PI) signal confined to the hypoxic core, a characteristic feature of 3D cultures. In MEL-CAL-derived spheroids (Figure 4), all treatments led to severe structural disruption, accompanied by a reduction in Calcein-AM fluorescence and widespread PI staining throughout the spheroid mass. Quantitative analysis confirmed a significant reduction in spheroid size and a robust increase in the red/green fluorescence *ratio* for all tested compounds. Notably, while most derivatives were tested at 20 μM, compounds **12** and **29** were already effective at 10 μM, inducing extensive cytotoxicity and complete spheroid disaggregation at lower concentrations (Table S2). Similarly, Malme-3M-derived spheroids (Figure 5) displayed reduced size and viability following treatment, as evidenced by decreased Calcein-AM fluorescence and increased PI positivity. Quantitative analysis demonstrated a pronounced reduction in spheroid area together with an increased red/green fluorescence *ratio* for all compounds tested. Among the tested conditions, compound **30** induced extensive spheroid disruption at 5 μM, whereas compounds **12**, **29** and **34** also markedly reduced spheroid size and viability at their respective tested concentrations (Table S2).

**Figure 4:**
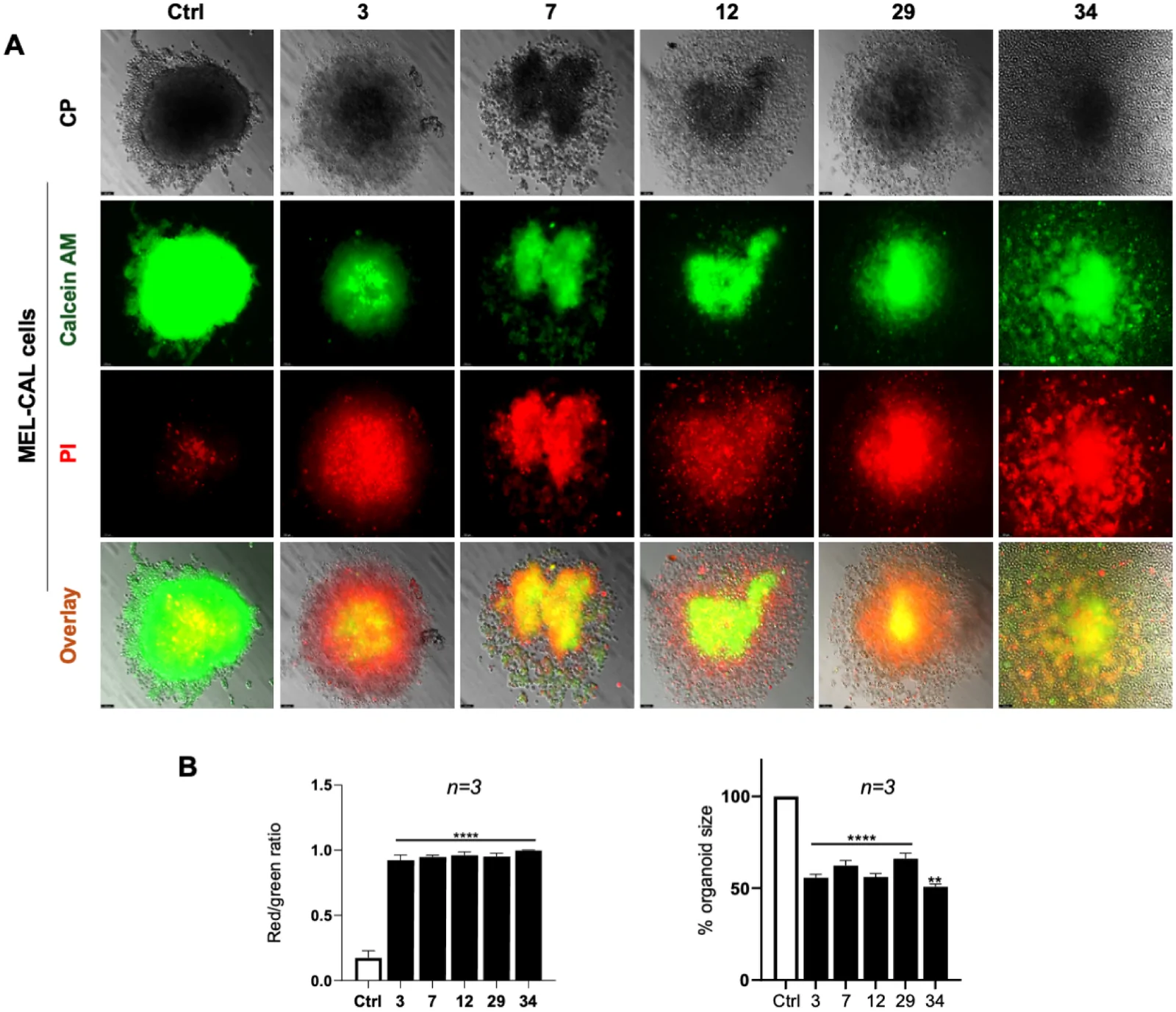
Effects of TRPM8 antagonists on size and viability of MEL-CAL-derived 3D spheroids. (A) Representative images of ECM-embedded spheroids derived from melanoma, MEL-CAL cells, either untreated (Ctrl) or treated with the indicated TRPM8 modulators for 21 days. The modulators were used at 10 μM for compounds **12** and **29** and 20 μM for compounds **3**, **7**, and **34**. Spheroids were stained with Calcein AM (green, live cells) and propidium iodide (PI, red, dead cells) and imaged by fluorescence microscopy. Top row: brightfield images; middle rows: fluorescence channels; bottom row: merged overlays. Overlay images show the spatial distribution of live and dead cells within the spheroids. Scale bar: 100 μm. (B) Quantification of spheroid viability and size. Left panel shows the *ratio* between mean fluorescence intensity of PI-positive (dead) cells/Calcein AM–positive (live) *ratio* under each treatment condition. Right panel reports spheroid size expressed as percentage relative to untreated controls (Ctrl, set to 100%). Data are presented as mean ± SD from at least three independent experiments. Statistical significance was determined by one-way ANOVA followed by Holm–Šídák’s multiple comparisons test, comparing each treatment group with the untreated control (****p < 0.0001).

**Figure 5:**
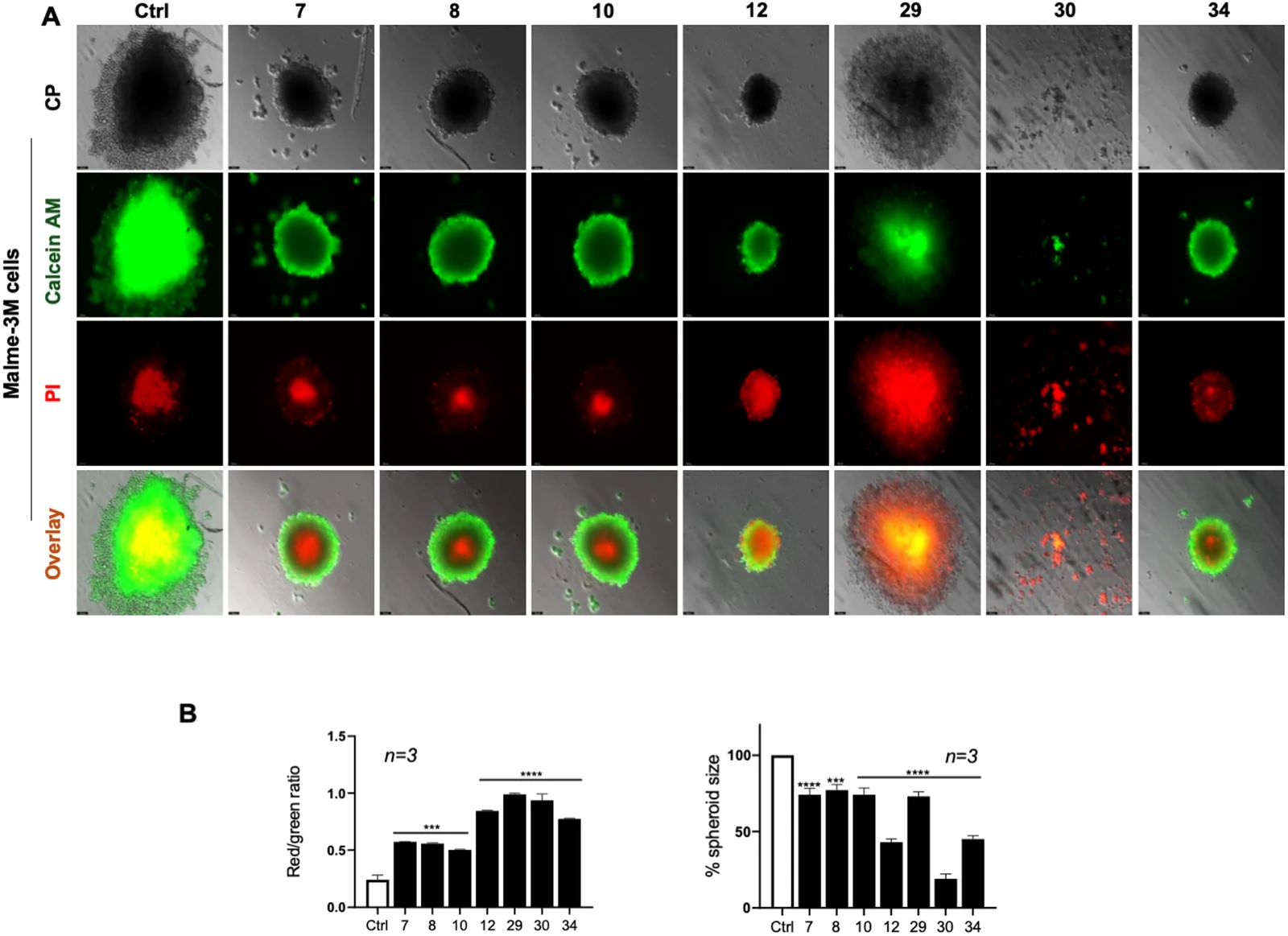
Effects of TRPM8 antagonists on size and viability of Malme-3M derived-spheroids. (A) Representative images of ECM-embedded spheroids derived from melanoma, Malme-3M, cells either untreated (Ctrl) or treated with the indicated TRPM8 modulators for 21 days. The modulators were used at 5 μM for compound **30**, 10 μM, for compounds **7**, **8**, **10**, **12**, and **34**, and 20 μM for compound **29**. Spheroids were stained with Calcein AM (green, live cells) and propidium iodide (PI, red, dead cells) and imaged by fluorescence microscopy. Top row: brightfield images; middle rows: fluorescence channels; bottom row: merged overlays. Overlay images show the spatial distribution of live and dead cells within the spheroids. Scale bar: 100 μm. (B) Quantification of spheroid viability and size. Left panel shows the *ratio* between mean fluorescence intensity of PI-positive (dead) cells/Calcein AM–positive (live) *ratio* under each treatment condition. Right panel reports spheroid size expressed as percentage relative to untreated controls (Ctrl, set to 100%). Data are presented as mean ± SD from at least three independent experiments. Statistical significance was determined by one-way ANOVA followed by Holm–Šídák’s multiple comparisons test, comparing each treatment group with the untreated control (***p < 0.001, ****p < 0.0001).

**Figure 6:**
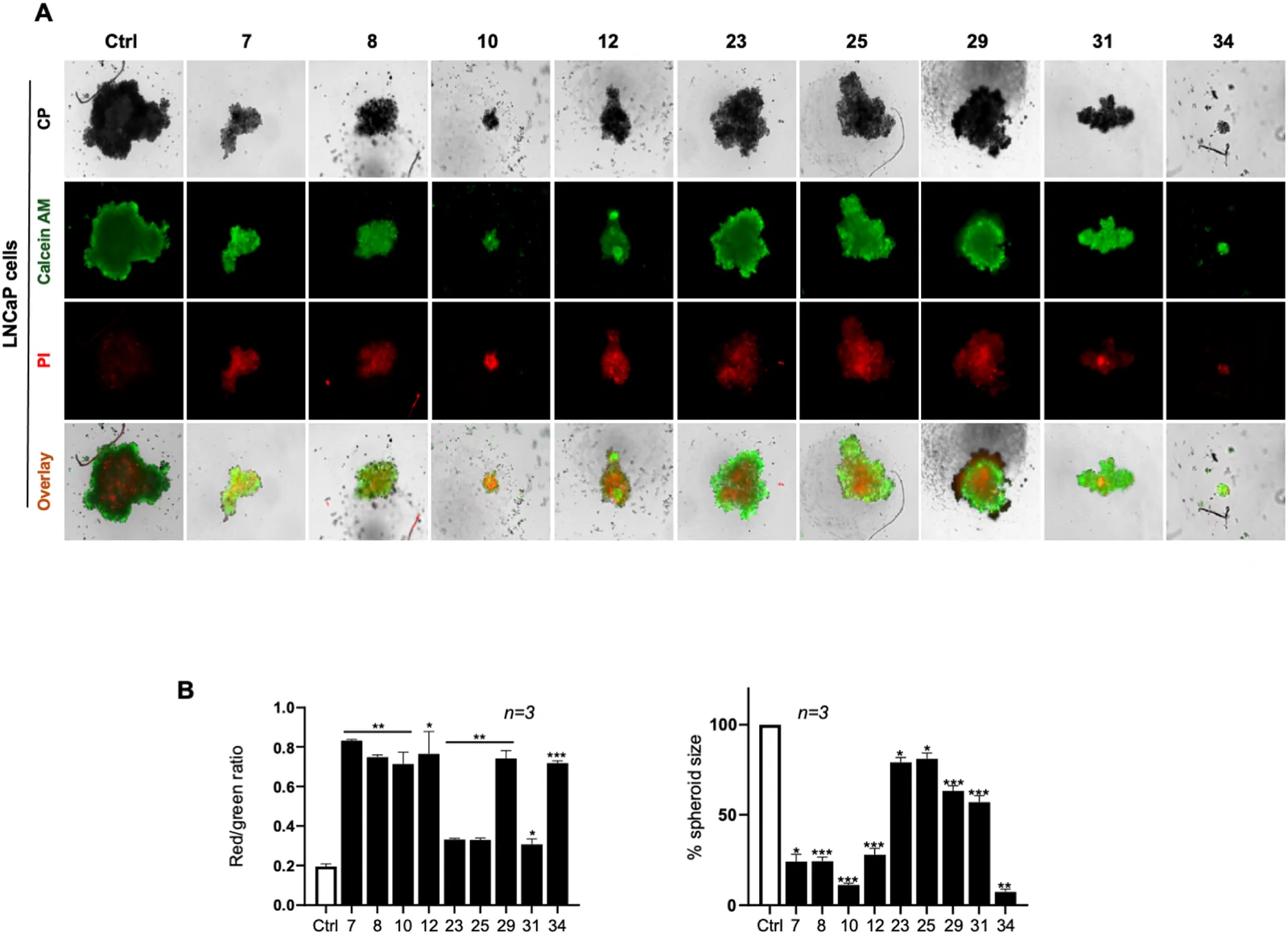
Effects of TRPM8 antagonists on size and viability of LNCaP-derived spheroids (A) Representative images of ECM-embedded spheroids derived from prostate cancer LNCaP cells, either untreated (Ctrl) or treated with the indicated TRPM8 modulators for 21 days. The compounds were used at the following concentrations: 5 μM (compound **10**), 10 μM (compounds **7**, **8**, **12**, **23**, **25**, **29**, and **34**), and 20 μM (compound **31**). Spheroids were stained with Calcein AM (green, live cells) and propidium iodide (PI, red, dead cells) and analyzed by fluorescence microscopy. Top row: brightfield images; middle rows: fluorescence channels; bottom row: merged overlays. Overlay images show the spatial distribution of live and dead cells within the spheroids. Scale bar: 100 μm. (B) Quantification of spheroid viability and size. In left panel, mean fluorescence intensity of Calcein AM–positive (live) and PI-positive (dead) cells under each treatment condition expressed as red/green *ratio* is shown. Right panel reports spheroid size expressed as percentage relative to untreated controls (Ctrl, set to 100%). Data are presented as mean ± SD from at least three independent experiments. Statistical significance was determined by one-way ANOVA followed by Holm–Šídák’s multiple comparisons test, comparing each treatment group with the untreated control (*p < 0.05, **p < 0.01, ***p < 0.001).

**Figure 7:**
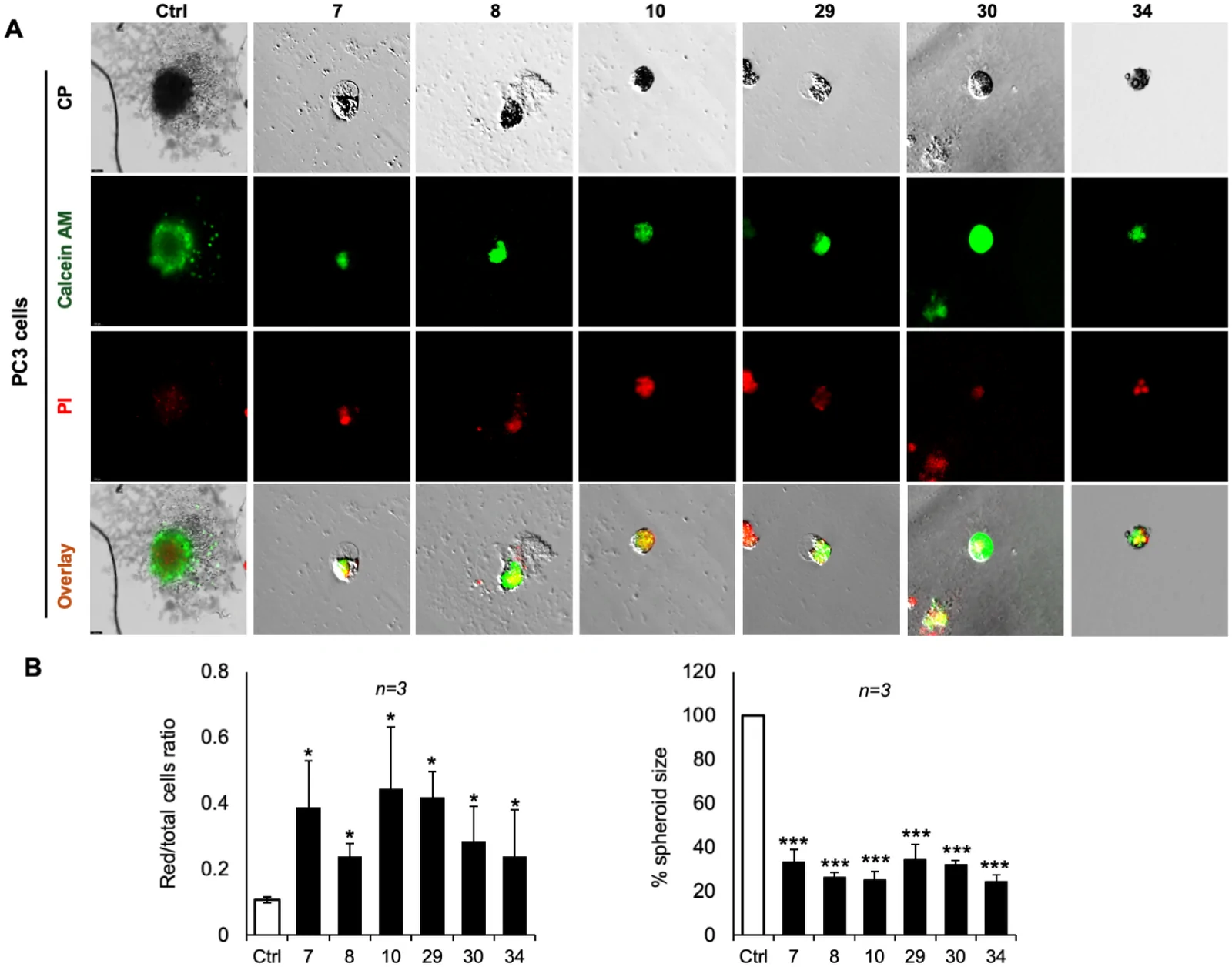
Effects of TRPM8 antagonists on size and viability of PC3–derived 3D spheroids. (A) Representative images of ECM-embedded spheroids derived from prostate cancer PC3 cells, either untreated (Ctrl) or treated with the indicated TRPM8 modulators for 21 days (all compounds were used at a concentration of 10 μM). Spheroids were stained with Calcein AM (green, live cells) and propidium iodide (PI, red, dead cells) and analyzed by fluorescence microscopy. Top row: brightfield images; middle rows: fluorescence channels; bottom row: merged overlays. Overlay images show the spatial distribution of live and dead cells within the spheroids. Scale bar: 100 μm. (B) Quantification of spheroid viability and size. In left panel, mean fluorescence intensity of Calcein AM–positive (live) and PI-positive (dead) cells under each treatment condition expressed as red/total cells (calcein AM + PI positive cells) *ratio* is shown. Right panel reports spheroid size expressed as percentage relative to untreated controls (Ctrl, set to 100%). Data are presented as mean ± SD from at least three independent experiments. Statistical significance was determined by one-way ANOVA followed by Holm–Šídák’s multiple comparisons test, comparing each treatment group with the untreated control (*p < 0.05, **p < 0.01, ***p < 0.001).

In LNCaP-derived spheroids (Figure 6), treatments induced heterogeneous but significant reductions in growth and viability. Compounds **7**, **8**, **10**, **12,** and **34** strongly reduced spheroid size and increased PI staining, resulting in an elevated red/green fluorescence *ratio*. Compound **29** also decreased viability, with a more moderate effect on spheroid size. Compound **31** (tested at 20 μM) induced partial spheroid disaggregation accompanied by increased PI positivity. Interestingly, compound **10** was already active at 5 μM, reducing organoid size and increasing PI staining to levels comparable to those observed with higher-dose treatments. Overall, quantification identified compounds **7**, **10**, **12** and **34** as the most effective in this model.

Finally, in PC3-derived spheroids (Figure 7), we report only the compounds showing the most robust antitumor activity in the initial screening. This model is technically more challenging, as PC3 cells grown in Matrigel form compact, lumen-containing, ampulla-like structures that are difficult to establish and maintain, as previously described.^46^

For this reason, only compounds showing the most pronounced biological effects were selected for further analysis and shown in the figure. Treatment with these compounds (**7**, **8**, **10**, **29**, **30**, **34**) markedly disrupted spheroid architecture, leading to reduced spheroid size and increased cell death, as indicated by enhanced PI positivity. Overall, all the reported compounds significantly impaired spheroid growth and viability in this 3D model.

### Patch-Clamp Assays

To further validate the target interaction, we continued our investigation of the most promising derivatives (**7**, **8**, **10**, **12**, **29**, **30**, **34**) subjecting them to electrophysiological assay using patch-clamp experiments. All compounds proved to dose-dependently abolish the menthol-induced calcium currents in HEK293 cells stably transfected with human TRPM8, corroborating our design strategy and the mechanism of action. Results are reported in Table 3.

**Table 3:** Q-Patch Results Compared with Ca^2+^ Imaging Data for the Most Promising Derivatives^a^

Compound	7	8	10	12	29	30	34
**Q-Patch IC**_**50**_ **(μM)**	0.87 ± 0.24	0.17 ± 0.1	1.14 ± 0.13	2.04 ± 0.54	0.76 ± 0.27	0.54 ± 0.11	7.94 ± 2.01
**Ca** ^**2+**^ **imaging IC**_**50**_ **(μM)**	0.69 ± 1.29	0.90 ± 1.29	0.90 ± 1.48	1.40 ± 1.29	0.15 ± 0.05	0.68 ± 0.08	1.13 ± 0.60

a Data are reported as mean of replicates *n* = 3. The potential antagonistic effect was evaluated after application of the agonist (menthol, 300 μM) alone and in the presence of the compounds under investigation at increasing concentrations.

As shown in Table 3, Q-Patch analysis confirmed the TRPM8 antagonism shown by the compounds in the calcium fluorimetric assay. The most promising results were for derivatives **7**, **8**, **29** and **30** which were characterized by a submicromolar inhibitory activity against the TRPM8 channel. Based on the results obtained, compounds were prioritized according to an optimal balance between potency, selectivity and structural representativeness, enabling a coherent and informative characterization of this new class of amino acid–based modulators. Derivatives **7**, **29** and **34** were selected as the most promising candidates for molecular dynamics simulations and *in vitro* pharmacokinetic evaluations.

Compounds **7**, **29** and **34** showed consistent and robust antiproliferative activity in all tumor-derived cell lines, with IC_50_ values at 72 h below 15.5 μM across melanoma and prostate models (compound **7**: MEL-CAL 15.0 μM, Malme-3M 10.4 μM, LNCaP 7.9 μM, PC3 15 μM; compound **29**: MEL-CAL 12.4 μM, Malme-3M 15.4 μM, LNCaP 12.4 μM and PC3 14.3 μM; compound **34**: MEL-CAL 14.8 μM, Malme-3M 10.4 μM, LNCaP 6.6 μM and PC3 15 μM). At the same time, the compounds displayed substantially reduced effects on nontumor cells, with markedly higher IC_50_ values (e.g., 28–46 μM) or no significant cytotoxicity, thereby providing a clear therapeutic window. Thus, the three selected compounds combine low micromolar potency on cancer cells with a clear safety window relative to the normal cell models used.

Beyond 2D viability assays, the 3D spheroid experiments further reinforced these findings. All three compounds induced strong cytotoxic responses, disrupting spheroid architecture and reducing spheroid size. Importantly, however, the selected set is not limited to melanoma: compound **34** also demonstrated strong activity in prostate cancer models, where it reduces spheroid size and viability at 10 μM in both PC-derived spheroid models and shows one of the lowest 2D IC_50_ values among the series in LNCaP cells. Thus, trio **7**, **29**, **34** offers both broad antitumor potency and complementary selectivity profiles: **7** provides reproducible efficacy across all tumor models, whereas **29** and **34** bridge melanoma and prostate efficacy while not exhibiting toxicity on normal cells.

In addition to their biological performance, these three molecules collectively display the structural diversity intentionally built into the series. Each represents a distinct chemotype arising from the major modifications introduced during design, ensuring that downstream molecular dynamics and pharmacokinetic analyses interrogate meaningful differences in the chemical space characterizing this new series of TRPM8 antagonists.

Before further characterizing the selected compounds, we also evaluated their selectivity against two of the most relevant TRP channels: TRPV1 and TRPA1. Derivatives **7**, **29** and **34** were tested at concentrations that fully inhibited TRPM8 in our assays, using heterologous expression systems for TRPA1 and TRPV1. At the highest concentration tested (50 μM), the maximal inhibition observed for TRPA1 and TRPV1 mediated Ca^2+^ responses was ≤5%, confirming the absence of relevant off target modulation on these channels under conditions that fully block TRPM8 (Figure S71).

### Molecular Dynamics

With the aim of further rationalizing, at the molecular level, the structural features crucial for protein-site recognition and binding over time, molecular dynamics (MD) simulations were performed for compounds **7**, **29**, and **34**, selected as the most promising derivatives based on their high activity in biological assays (Tables 1, 2, and Figure S78, Supporting Information). Each protein–ligand system was subjected to energy minimization, followed by equilibration steps, and a long MD simulation of 1 μs (see Experimental Section).

The analysis of the trajectories revealed that compounds **7** (Figure 8A), **29** (Figure 8B) and **34** (Figure 8C) maintained stable interactions with the TRPM8 binding cavity throughout the simulation. In all cases, the protein root-mean-square deviation (RMSD) values remained stable over the time (∼4–5 Å), confirming that the overall conformation of the channel and the ligand-protein binding were preserved. The RMSD trend of compound **34** (Figure 8C) showed an initial increase during the first 100 ns, followed by stabilization around a constant value (∼2 Å), indicating that the ligand rapidly adapted to the binding pocket and remained tightly associated with the protein over time. Although a transient increase in RMSD was observed at the beginning of the simulation, the values subsequently remained constant, reflecting the attainment of a stable equilibrium conformation within the site. This behavior was similarly observed for the other active compounds, confirming that all three ligands achieve stable binding pose and maintain persistent interactions within the TRPM8 channel throughout the entire simulation.

**Figure 8:**
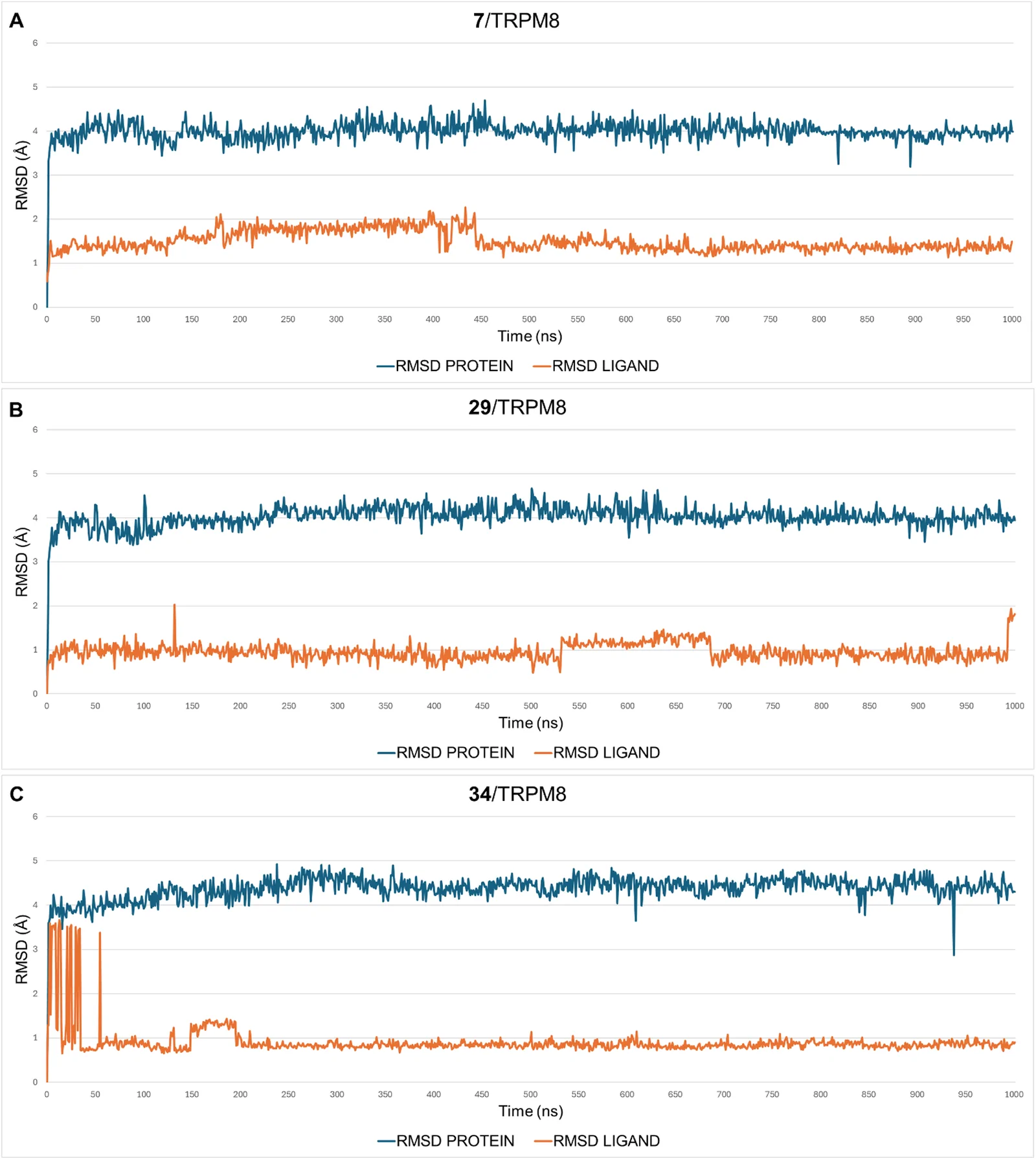
RMSD plots of the protein in complex with compounds **7** (**A**), **29** (**B**), and **34** (**C**). Each trajectory frame was extracted every 2000 ps and superimposed onto the initial structure (frame 0). RMSD was calculated for backbone atoms of the protein and all heavy atoms of the ligand.

### Genetic Silencing of TRPM8 Confirms On-Target Cytotoxicity of Lead Compounds

To validate the on-target mechanism underlying the cytotoxic activity of the selected compounds, TRPM8 expression was silenced in MEL-CAL cells using siRNA-mediated knockdown. Western blot analysis confirmed efficient TRPM8 knockdown in TRPM8-targeting siRNA–transfected cells compared with control siRNA (Figure 9A). Cells were then treated with three of the most active compounds identified in previous assays (**7**, **29**, and **34**), used at 40 μM to ensure robust biological effect and facilitate discrimination between TRPM8-dependent and potential nonspecific cytotoxic effects. Cell death was evaluated after 12 h by propidium iodide (PI) staining. As shown in Figure 9 (B–E), all compounds significantly increased PI-positive cells under control siRNA-transfected conditions. In contrast, no significant increase in PI positivity was observed in TRPM8-silenced cells, indicating that the cytotoxic effects are largely dependent on TRPM8 expression.

**Figure 9:**
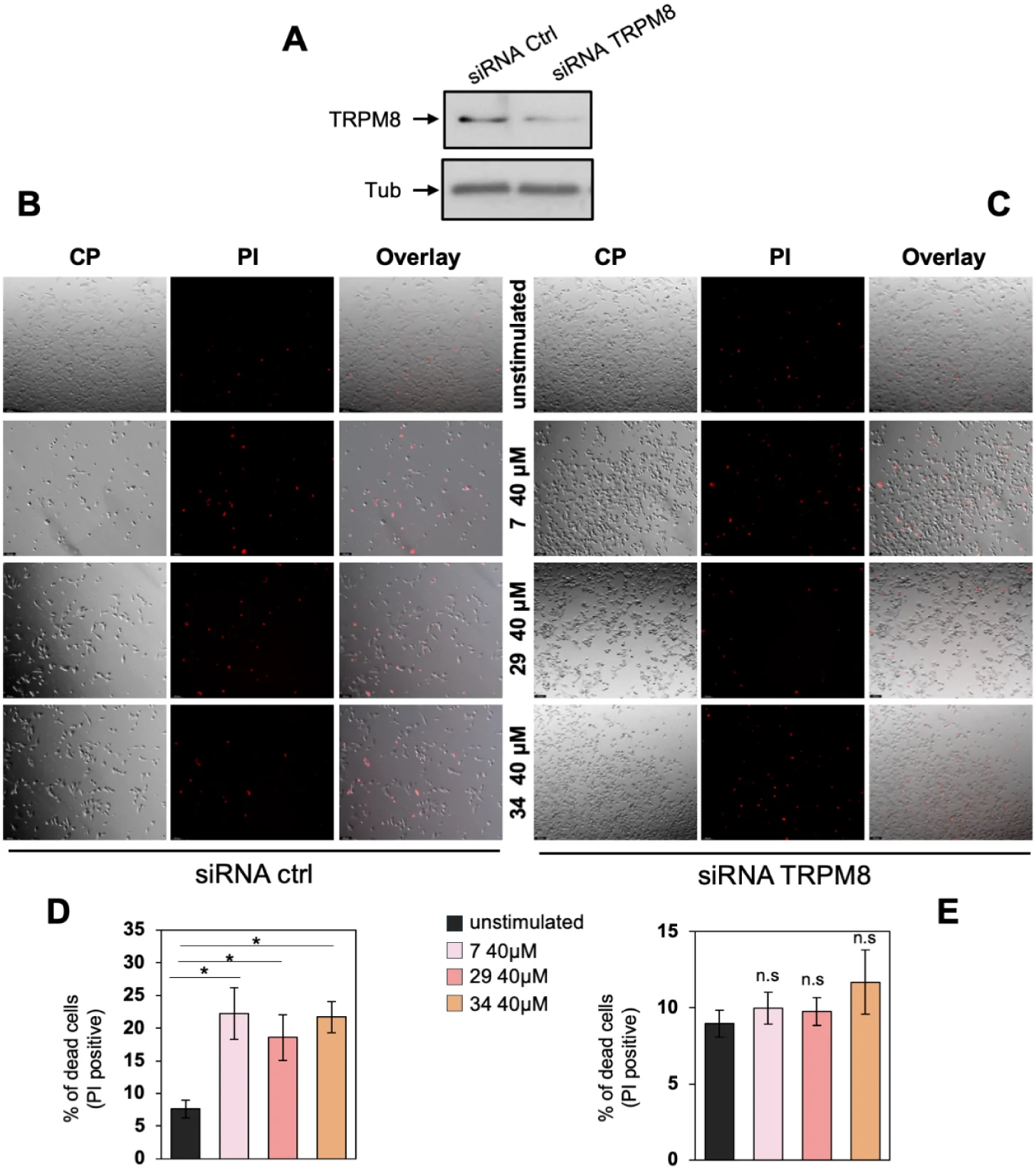
(A) Western blot (WB) analysis of TRPM8 expression levels. Cells were transfected with either control siRNA (siRNA Ctrl) or TRPM8-targeting siRNA (siRNA TRPM8) to confirm efficient gene silencing. Tubulin (Tub) was used as a loading control. (B, C) Representative phase-contrast (PC) and fluorescence images of (B) control siRNA- and (C) TRPM8 siRNA-transfected MEL-CAL cells. Following transfection, cells were treated with 40 μM of compounds **7**, **29**, or **34**, or left untreated, as described in the Methods. Cell death was assessed by propidium iodide (PI; red fluorescence) incorporation. Merged images show overlay of phase-contrast and fluorescence signals. Scale bars: 100 μm. (D, E) Quantification of PI-positive cells in (D) control siRNA and (E) TRPM8 siRNA conditions quantified as reported in Methods Section. Data are presented as mean ± SD (*n* = 3). Statistical significance was determined using Student’s *t*-test (*p < 0.05). n.s not significant.

These findings demonstrate that the cytotoxic activity of compounds **7**, **29**, and **34** is largely dependent on TRPM8 expression, supporting an on-target mechanism of action and confirming TRPM8 as a key mediator of their antiproliferative effects.

### *In Vitro* Drug-Likeness Evaluation

Microsomes, subcellular fractions of the endoplasmic reticulum enriched in major drug-metabolizing enzymes, provide a reliable and cost-effective *in vitro* model for assessing drug metabolism.^47^ Owing to their enzymatic composition, stability, and suitability for high-throughput screening, the *in vitro* metabolic stability of compounds **7**, **29**, and **34** was assessed using human liver microsomes (HLMs) in the presence of NADPH.

Each compound was incubated in potassium phosphate buffer (pH 7.4) at 37 °C for up to 60 min, and samples were quenched with ice-cold organic solvent at predefined time points. The remaining parent compound was quantified by LC-MS/MS using a triple-quadrupole platform. First-order decay kinetics were observed, and elimination rate constants (k) were determined from the slope of the natural logarithm of the percentage of remaining compound versus incubation time (Figure S72). The corresponding half-lives (*t*_1/2_) were 19.4, 2.6, and 10.3 min for compounds **7** (CL_int,scaled_: 57.9 mL min^–1^ kg^–1^), **29** (CL_int,scaled_: 431.6 mL min^–1^ kg^–1^), and **34** (CL_int,scaled_: 109.5 mL min^–1^ kg^–1^), respectively, indicating markedly different metabolic stabilities under identical experimental conditions.

The progressive disappearance of the parent compound over time was accompanied by the time-dependent formation of several metabolites (Figure S73). Metabolite identification was performed using high-resolution MS/MS data processed with Compound Discoverer 3.3, following a customized workflow designed for the detection and structural elucidation of both predicted and unexpected metabolites (Figure S73D). This approach enabled tentative structural assignments based on accurate mass measurements and diagnostic fragmentation patterns.

LC-MS/MS analysis showed that the major metabolites of compound **7** originated from dealkylation pathways (M1–7 and M3–7; Figure S74). Interpretation of the MS/MS spectra revealed that M1–7 (C_14_H_13_F_2_N, Figure S74A) was generated through the loss of a C_12_H_11_NO_2_ fragment, whereas M3–7 (C_25_H_22_F_2_N_2_O_2_, Figure S74C) arose from O-demethylation, involving cleavage of a methyl group from a methoxy moiety.

Additional minor oxidative metabolites (M2–7 and M4–7, C_26_H_24_F_2_N_2_O_3_) were identified. The corresponding biotransformations were localized on the indole ring, as indicated by the diagnostic fragment ion observed at *m*/*z* 218.0811 (Figure S74B,D).

Analogous metabolites were detected for compounds **29** and **34** (Figure S73B,C). The major metabolites, M2–29 (C_22_H_22_N_2_O_2_) and M2–34 (C_20_H_21_N_3_O_2_), were both generated through O-demethylation of a methoxy substituent (Figures S76B and S75B).

Given that compound **7** exhibited the highest hepatic stability among the tested compounds, additional analyses were conducted to further investigate its *in vitro* NADPH-dependent and -independent metabolic profile.

To this end, parallel incubation in the absence of the cofactor was included as a negative control to evaluate NADPH-independent metabolism and possible nonenzymatic degradation. After 60 min of incubation, the percentage of remaining parent compound in the NADPH-free control dropped below 50%, indicating that compound **7** also underwent significant NADPH-independent degradation. To exclude the possibility of nonenzymatic degradation, the chemical stability of the test compounds was further assessed in PBS buffer (pH 7.4) at 37 °C for up to 60 min, revealing that they remained substantially more stable under these experimental conditions (Figure S77).

Given their ester functionalities, compound **7** exhibited NADPH-independent hepatic degradation, which was attributed to microsomal carboxylesterase (CES) activity. This hypothesis was corroborated by HLM incubations performed in the presence of a CES inhibitor^48,49^ under which no significant degradation was observed (Figure S77).

Overall, the data indicate that NADPH-independent, esterase-driven hydrolysis plays a predominant role in the metabolic instability of compound **7**, highlighting the involvement of carboxylesterase-mediated pathways to its hepatic clearance.

During drug metabolism, enzymatic reactions may generate reactive metabolites with enhanced pharmacological activity or toxicity compared with the parent compound. Among detoxification pathways, glutathione (GSH) conjugation represents a major protective mechanism. To evaluate the potential formation of reactive intermediates, a GSH trapping assay was performed by incubating compound **7** with HLMs in the presence of NADPH and GSH. In this assay, reactive intermediates are trapped as stable GSH conjugates, which are subsequently identified by LC-MS.^50,51^ A negligible amount of a metabolite of compound **7** formed a GSH conjugate (C_29_H_33_F_2_N_5_O_8_S, RT: 0.51 min, 650.2062 *m*/*z*), indicating a very limited extent of bioactivation and supporting its favorable safety profile.

Finally, the stability of compound **7** in human plasma was assessed. Remarkably, the compound remained unchanged after incubation for up to 240 min, indicating excellent plasma stability, consistent with limited hydrolysis by plasma esterases (Figure S77). Collectively, these results highlight a suitable drug-likeness profile for compound **7**.

## Conclusion

Prostate cancer and melanoma represent two of the most prevalent malignancies worldwide, with steadily increasing incidence and high mortality rates, particularly in advanced and therapy-resistant stages.^52,53^

Despite significant progress in chemotherapy, hormone therapy, immunotherapy, and targeted treatments,^54,55^ drug resistance remains a major limitation that severely compromises long-term patient survival. These challenges underscore the urgent need for the identification and validation of novel molecular targets involved in tumor development and aggressiveness.

TRPM8 is a well-recognized therapeutic target in androgen-dependent prostate cancer, whereas its blockade by TRPM8 antagonists has emerged as a valuable strategy for the development of new drug candidates.^42,43^ Conversely, the involvement of this channel in melanoma progression and the molecular mechanism underlying it, have only recently been elucidated by our group.^44^ In the present study, we put in place all our consolidate expertise in the development of TRPM8 antagonists to explore the therapeutic potential of this target in both prostate and melanoma cancer models. Based on our previously identified tryptophan derived lead compound (compound **14**)^40^ we designed a new series of amino acid–based derivatives, in the attempt to increase the molecular diversity of the arsenal of TRPM8 blockers. Their TRPM8 antagonist activity was assessed by calcium fluorimetric experiments and rationalized through molecular docking studies, adding new insights about the channel binding pocket and the ligand/target crucial interactions. The most active compounds were subsequently evaluated for their cytotoxic activity in prostate and melanoma cancer cell lines and compared with nontransformed control cells to obtain a preliminary assessment of tumor selectivity. The antitumor effectiveness of this series was further ascertained in 3D prostate and melanoma spheroid models, where several compounds demonstrated a strong ability to disrupt spheroid architecture and reduce cell viability. From this exhaustive *in vitro* antitumoral characterization, we selected 3 compounds for their most promising activity and their molecular diversity, to trace their drug likeness profile and selectivity profile against TRPA1 and TRPV1. Among them, we were able to identify compound **7** as the most promising derivative whose antitumoral characterization will be further expanded in the future.

As next step, a broader selectivity and safety profiling for compound **7** will be a priority in future preclinical work. Nonetheless, the lack of detectable activity on TRPA1 and TRPV1, together with the robust TRPM8 antagonism, the tumor-selective cytotoxicity profile, and the favorable *in vitro* ADME properties, supports the view that our lead compound behaves as TRPM8-directed anticancer candidate with an encouraging preliminary selectivity profile. Although additional pharmacokinetic, toxicological and *in vivo* studies will be required to formally define the therapeutic window of these compounds, the preferential activity observed in tumor cells supports further development of TRPM8 antagonists as anticancer agents.

Overall, our findings further support the suitability of amino acid–based scaffolds for the design of TRPM8 modulators; moreover, our results represent a further validation of TRPM8 as a relevant therapeutic target involved in tumor progression. Notably, to the best of our knowledge, this study represents the first systematic effort to develop TRPM8 antagonists as potential antimelanoma agents. We exploited our previously acquired insights in this field, which provided a molecular understanding of molecular mechanisms underlying the antitumor activity of known TRPM8 blockers,^44^ thereby opening new avenues for targeted therapeutic intervention in this highly aggressive cancer.

## Experimental Section

### General Methods

All reagents and solvents used were purchased from Sigma-Aldrich (Milan, Italy) or Fluorochem Limited (Unit 14, Graphite Way, Rossington Park, Hadfield, Derbyshire England) unless otherwise stated. Reactions were performed under magnetic stirring in round-bottom flasks or under microwave irradiation assisted (Biotage, Initiator EU). TLC analysis of reaction mixtures was performed on precoated glass silica gel plates (F254, 0.25 mm, VWR International). The crude products were purified by the Isolera Spektra One automated flash chromatography system (Biotage, Uppsala, Sweden), using commercial silica gel cartridges (SNAP KP-Sil, Biotage). NMR spectra were recorded on a Bruker Avance 400 MHz apparatus, at room temperature. Chemical shifts were reported in δ values (ppm) relative to internal Me_4_Si for ^1^H and ^13^C NMR. J values were reported in hertz (Hz). C–F J couplings were determined through the analysis of the ^13^C DEPT spectra multiplets. ^1^H NMR peaks were described using the following abbreviations: s (singlet), bs (broad singlet), d (doublet), t (triplet), and m (multiplet). HR-MS and IT-MS spectra were recorded by LTQ-Orbitrap-XL-ETD mass spectrometer (Thermo Scientific, Bremen, Germany), equipped with electrospray ionization. The purity of final compounds was assessed by ultrahigh-performance liquid-chromatography (UHPLC) analyses, performed on a Jasco Extrema LC 4000 (Jasco, Japan) consisting of an LC-Net CG cable controller, quaternary flow pump system PU-4285, a DG-4000–04 degasser, a UV-4075 detector, and an AS-4250 autosampler. Purity assessment UHPLC runs were carried out on an EVO C18 150 mm × 2.1 mm × 2.6 μm (100 Å) column (Phenomenex, Bologna, Italy). The optimal mobile phase consisted of 0.1% HCOOH/H2O v/v (A) and 0.1% HCOOH/ACN v/v (B). Analysis was performed in gradient elution as follows: 0–10.00 min, 5–95% B; 10–12.00 min, 95–95% B; 12–15.00 min, isocratic to 5% B. Flow rate was 0.5 mL min^‑1^. The injection volume was set at 5 μL. Data acquisition was set in the range 190–800 nm and chromatograms were monitored at 254 nm. All the final compounds showed a purity ≥95% by HPLC analysis. Optical rotations were measured on an Atago Polax 2-L polarimeter at a concentration of 0.1 g/100 mL.

**General procedure A: reductive amination (1–4, 33).** The starting amino acid (1.0 equiv) was dissolved in dry methanol (15.0 mL) under positive nitrogen flow, and added with benzaldehyde or 4-ethoxybenzaldehyde, (1.2 equiv), and stirred overnight under nitrogen atmosphere. Then, NaBH_4_ (3.0 equiv) was added and the mixture was reacted for further 1 h at room temperature. Afterward, the reaction was evaporated under vacuum, diluted with ethyl acetate, washed with a 2 M solution of NaOH (2 × 20.0 mL) and extracted. The organic layer was dried over Na_2_SO_4_, filtered, and evaporated. The crude products were purified by flash chromatography using mixtures of DCM/ethyl acetate as eluent affording the corresponding derivatives in 61–75% of yield.

**General procedure B: N-alkylation (5–10, 23–29, 34, 35).** The proper amino intermediate was dissolved in tetrahydrofuran, added with DIPEA (3.0 equiv) and benzyl bromide, 4-phenylbenzyl bromide, 2-(bromomethyl)­naphthalene or 2,4-difluorobenzyl bromide (3.0 equiv) and heated at 120 °C for 45 min in a 20 mL tube under microwave irradiation (150 W). Afterward, the reaction mixture was diluted with dichloromethane and washed twice with a 2 M solution of NaOH (2 × 20.0 mL). The organic layer was extracted, dried over Na_2_SO_4_, filtered, evaporated and purified by flash chromatography using mixtures of *n*-hexane/ethyl acetate as mobile phase furnishing the alkylated intermediates in 23–77% of yield.

**General procedure C: hydrolysis reaction (11, 30–32).** 1.0 mmol of the ester intermediate **8**, **23**, **26** or **28** was dissolved in methanol (5.0 mL), added with a 2 M solution of NaOH and refluxed overnight. The reaction was evaporated *in vacuo*, quenched by a 2 M aqueous solution of HCl, and the mixture was extracted with ethyl acetate (3 × 20.0 mL), dried over Na_2_SO_4_, and concentrated. The obtained derivatives were used in the next step with no further purification.

**General procedure D: coupling reaction (12, 13, 30–32).** 1.0 mmol of the proper carboxylic acid was dissolved in DCM and added with HOBt (1.2 equiv), HBTU (1.2 equiv), DIPEA (2.4 equiv), and methylamine or butylamine (1.2 equiv) and stirred at −15 °C or at 55 °C for 12 h. Then, the solvent was evaporated in vacuum, and the residue was dissolved in dichloromethane and washed with water (3 × 20.0 mL), a saturated solution of NaHCO_3_ (3 × 20.0 mL), and a solution of citric acid (10% w:w, 3 × 20.0 mL). The organic phase was extracted, dried over Na_2_SO_4_, filtered, and concentrated under vacuum. The crude products were purified by flash chromatography using mixtures of *n*-hexane/ethyl acetate as mobile phase (46–66% yield).

**General procedure E: Negishi coupling (16–22).** Iodine (0.15 equiv) was added to a stirred suspension of zinc dust (3.0 equiv) in dry DMF and the mixture was stirred at 50 °C for 30 min. Then, intermediate **14** (1.0 equiv), solubilized in dry DMF, was added to the reaction mixture and, then, stirred at 100 °C until the completion of the reaction, monitored by TLC. To the stirring solution of methyl (S)-2-((tert-butoxycarbonyl)­amino)-3-(4-chlorophenyl)­propanoate (**15a**) or (R)-2-((tert-butoxycarbonyl)­amino)-3-(4-chlorophenyl)­propanoate (**15b**) the proper aryl halide (1.0 equiv), Pd_2_(dba)_3_ (0.013 equiv), and XPhos (0.025 equiv) were added. The reaction mixture was stirred at 120 °C for 4 h. Then, water was added and the mixture was extracted with ethyl acetate (2 × 50.0 mL), dried over Na_2_SO_4_, and concentrated *in vacuo*. The crude products were purified by flash chromatography using mixtures of *n*-hexane/ethyl acetate as eluent (43–54% yield).

**General procedure F: Boc removal (23.1–29.1).** The proper protected intermediate was dissolved in a mixture of dichloromethane/trifluoroacetic acid (*ratio* 3/1, v/v) and added with triisopropylsilane. The reaction mixture was stirred for 2 h at room temperature, then quenched with a 4 M solution of NaOH and extracted with ethyl acetate. Then, the organic layer was dried over Na_2_SO_4_, evaporated, precipitated from dichloromethane/diethyl ether and filtered off. The obtained solid was used in the next reaction step without further purification.

**Methyl benzyl-** *L* **-tryptophanate (1).** Obtained from *L*-tryptophan methyl ester and benzaldehyde according to the general procedure A. FC *n*-hexane/ethyl acetate (*ratio* 7/3, v/v). R*f*: 0.46. Whitish powder (75% yield). ^1^H NMR (400 MHz, CDCl_3_); δ: 2.24 (bs, 1H, N*H*); 3.21–3.31 (m, 2H, C*H*_2_); 3.69 (s, 3H, C*H*_3_); 3.72–3.79 (m, 2H, C*H*_2_); 3.91 (d, 1H, C*H*, *J* = 13.0 Hz); 6.97 (s, 1H, aryl); 7.17 (t, 1H, aryl, *J* = 9.9 Hz); 7.24 (t, 1H, aryl, *J* = 7.6 Hz); 7.31–7.35 (m, 5H, aryl); 7.41 (s, 1H, aryl); 7.65 (d, 1H, aryl, *J* = 7.7 Hz); 8.45 (bs, 1H, N*H*). HR-MS *m*/*z* calcd for C_19_H_20_N_2_O_2_ [(M + H)^+^]: 309.1598; found 309.1546.

**Ethyl benzyl-** *L* **-tryptophanate (2).** Synthesized from *L*-tryptophan ethyl ester and benzaldehyde according to the general procedure A. FC *n*-hexane/ethyl acetate (*ratio* 7/3, v/v). R*f*: 0.45. Whitish powder (72% yield). ^1^H NMR (400 MHz, CDCl_3_); δ: 1.35 (t, 3H, C*H*_3_, *J* = 7.2 Hz); 3.19–3.27 (m, 2H, C*H*_2_); 3.70–3.76 (m, 2H, C*H*_2_); 3.90 (d, 1H, C*H*, *J* = 13.0 Hz); 4.22 (dd, 2H, C*H*_2_, *J*′ = 5.2 Hz, *J*″ = 11.1 Hz); 6.94 (s, 1H, aryl); 7.14 (t, 1H, aryl, *J* = 9.6 Hz); 7.20 (t, 1H, aryl, *J* = 7.5 Hz); 7.30–7.35 (m, 5H, aryl); 7.38 (s, 1H, aryl); 7.65 (d, 1H, aryl, *J* = 7.7 Hz); 8.48 (bs, 1H, N*H*). HR-MS *m*/*z* calcd for C_20_H_22_N_2_O_2_ [(M + H)^+^]: 323.1754; found 323.1778.

**Benzyl benzyl-** *L* **-tryptophanate (3).** Synthesized from *L*-tryptophan benzyl ester and benzaldehyde according to the general procedure A. FC in *n*-hexane/ethyl acetate 8/2, R*f*: 0.48. Yellowish oil (65% yield). [α]^25^_D_: −8.00 ± 0.51 (c = 0.10, MeOH). ^1^H NMR (400 MHz, CD_3_OD): δ: 3.17 (d, 2H, C*H*_2_, *J* = 7.0 Hz); 3.64 (d, 1H, C*H*_2a_, *J* = 12.9 Hz); 3.69 (t, 1H, C*H*, *J* = 6.9 Hz); 3.76 (d, 1H, C*H*_2b_, *J* = 12.9 Hz); 4.98 (s, 2H, C*H*_2_); 6.97 (s, 1H, aryl); 7.00 (d, 1H, aryl, *J* = 7.8 Hz); 7.08–7.13 (m, 3H, aryl); 7.17–7.19 (m, 3H, aryl); 7.19–7.25 (m, 3H, aryl); 7.29 (t, 2H, aryl, *J* = 3.7 Hz); 7.36 (d, 1H, aryl, *J* = 7.9 Hz); 7.47 (d, 1H, aryl, *J* = 7.9 Hz). ^13^C NMR (100 MHz, CD_3_OD) δ: 32.6, 55.3, 65.1, 70.1, 113.3, 114.9, 121.9, 122.3, 125.0, 127.0, 130.8, 131.3, 131.7, 131.9, 131.96, 131.97, 132.0, 139.7, 140.7, 142.8, 178.3. IT-MS *m*/*z* calcd for C_25_H_24_N_2_O_2_ [(M + H)]^+^: 385.1911; found 385.1898.

**Methyl (4-ethoxybenzyl)­tryptophanate (4).** Obtained from *L*-tryptophan methyl ester and 4-ethoxybenzaldehyde according to the general procedure A. FC *n*-hexane/ethyl acetate (*ratio* 7/3, v/v). R*f*: 0.44. Whitish powder (69% yield). ^1^H NMR (400 MHz, CD_3_OD); δ: 1.38 (t, 3H, C*H*_3_, *J* = 7.0 Hz); 3.10–3.21 (m, 2H, C*H*_2_); 3.57–3.60 (m, 4H, C*H*_2_ and C*H*); 3.72 (d, 1H, C*H*, *J* = 6.7 Hz); 4.01 (dd, 2H, C*H*_2_, *J*′ = 7.0 Hz, *J″* = 13.9 Hz); 6.80 (d, 2H, aryl, *J* = 8.6 Hz); 6.99 (t, 1H, aryl, *J* = 7.2 Hz); 7.05 (s, 1H, aryl); 7.09–7.12 (m, 3H, aryl); 7.35 (d, 1H, aryl, *J* = 8.2 Hz); 7.45 (d, 1H, aryl, *J* = 8.2 Hz). HR-MS *m*/*z* calcd for C_21_H_24_N_2_O_3_ [(M + H)^+^]: 353.1860; found 353.1888.

**Methyl N** ^**α**^ **-([1,1′-biphenyl]-4-ylmethyl)-N** ^**α**^ **-benzyl-** *L* **-tryptophanate (5).** Final derivative **5** was synthesized from **1** and 4-phenylbenzyl bromide according to the general procedure B. FC in *n*-hexane/ethyl acetate 1/1, R*f*: 0.48. Yellow oil (62% yield). [α]^25^_D_: + 4.07 ± 0.16 (c = 0.10, MeOH). ^1^H NMR (400 MHz, CDCl_3_): 3.04 (dd, 1H, C*H*_*2a*_, *J*′ = 5.9; *J*″ = 14.0 Hz); 3.31 (dd, 1H, C*H*_*2b*_, *J′* = 9.0; *J*″ = 14.4 Hz); 3.51 (dd, 2H, C*H*_2_, *J*′ = 11.7; *J*″ = 13.8 Hz); 3.63 (s, 3H, C*H*_3_); 3.76 (dd, 1H, C*H*, *J*′ = 5.9; *J*″ = 8.4 Hz); 3.97 (dd, 2H, C*H*_2_, *J*′ = 8.5; *J*″ = 9.6 Hz); 6.82–6.85 (m, 2H, aryl); 7.02–7.06 (m, 2H, aryl); 7.14–7.23 (m, 4H, aryl); 7.27 (t, 5H, aryl, *J* = 8.2 Hz); 7.35–7.42 (m, 4H, aryl); 7.51 (d, 2H, aryl, *J* = 7.2 Hz); 7.84 (bs, 1H, N*H*). ^13^C NMR (100 MHz, CDCl_3_) δ: 26.3, 29.7, 51.0, 54.4, 61.4, 112.2, 118.7, 119.2, 121.8, 122.7, 126.96, 127.06, 127.4, 128.2, 128.7, 128.8, 129.2, 136.1, 138.7, 139.6, 139.9, 141.1, 172.9. HR-MS *m*/*z* calcd for C_32_H_30_N_2_O_2_ [(M + H)]^+^: 475.2380; found 475.2389.

**Methyl N** ^**α**^ **-benzyl-N** ^**α**^ **-(naphthalen-2-ylmethyl)-** *L* **-tryptophanate (6).** Compound **6** was obtained from **1** and 2-(bromomethyl)­naphthalene according to the general procedure B. FC in *n*-hexane/ethyl acetate 8/2, R*f*: 0.48. Yellow oil (48% yield). [α]^25^_D_: −24.05 ± 0.15 (c = 0.10, MeOH) ^1^H NMR (400 MHz, CDCl_3_): 3.16 (dd, 1H, C*H*_*2a*_, *J*′ = 6.0; J″ = 14.4 Hz); 3.42 (dd, 1H, C*H*_*2b*_, *J*′ = 9.0; J″ = 14.4 Hz); 3.63 (d, 1H, C*H*_*2a*_, *J* = 13.8 Hz); 3.73–3.76 (m, 4H, C*H*_3_ and C*H*_*2b*_); 3.90 (dd, 1H, C*H*, *J*′ = 6.0; J″ = 8.8 Hz); 4.08 (d, 1H, C*H*_2a_, *J* = 13.8 Hz); 4.20 (d, 1H, C*H*_2b_, *J* = 13.8 Hz); 6.82 (t, 1H, aryl, *J* = 7.4 Hz); 6.91 (s, 1H, aryl); 7.07–7.14 (m, 2H, aryl); 7.24–7.32 (m, 4H, aryl); 7.34–7.37 (m, 2H, aryl); 7.45–7.48 (m, 3H, aryl); 7.73–7.78 (m, 3H, aryl); 7.82–7.85 (m, 1H, aryl); 7.91 (bs, 1H, N*H*). ^13^C NMR (100 MHz, CDCl_3_) δ: 26.3, 51.0, 54.7, 54.9, 61.4, 110.9, 112.2, 118.7, 119.2, 121.8, 122.7, 125.5, 128.8, 127.0, 127.1, 127.5, 127.6, 127.7, 127.9, 128.2, 128.9, 132.8, 133.3, 136.1, 137.1, 139.5, 173.0. HR-MS *m*/*z* calcd for C_30_H_28_N_2_O_2_ [(M + H)]^+^: 449.2224; found 449.2257.

**Methyl N** ^**α**^ **-benzyl-N** ^**α**^ **-(2,4-difluorobenzyl)-** *L* **-tryptophanate (7).** Derivative **7** was obtained starting from **1** and 2,4-difluorobenzyl bromide in accordance with the procedure B. FC in *n*-hexane/ethyl acetate 7/3, R*f*: 0.46. Yellow oil (59% yield). [α]^25^_D_: −6.08 ± 0.35 (c = 0.10, MeOH). ^1^H NMR (400 MHz, CDCl_3_): δ: 3.13 (dd, 1H, C*H*_*2a*_, *J*′ = 6.2; *J″* = 14.4 Hz); 3.41 (dd, 1H, C*H*_*2b*_, *J*′ = 8.6; *J*″ = 14.4 Hz); 3.65 (d, 1H, C*H*_*2a*_, *J* = 13.8 Hz); 3.73 (s, 3H, C*H*_3_); 3.78–3.84 (m, 2H, C*H* and C*H*_*2b*_); 3.90 (d, 1H, C*H*_*2a*_, *J* = 6.6 Hz); 4.04 (d, 1H, C*H*_*2b*_, *J* = 13.9 Hz); 6.71–6.76 (m, 2H, aryl); 6.94 (s, 1H, aryl); 7.01 (t, 1H, aryl, *J* = 7.5 Hz); 7.16–7.22 (m, 2H, aryl); 7.25–7.31 (m, 6H, aryl); 7.35 (d, 1H, aryl, *J* = 7.5 Hz); 8.00 (bs, 1H, N*H*). ^13^C NMR (100 MHz, CDCl_3_) δ: 47.1, 51.1, 54.8, 61.8, 103.4 (t, ^2^ *J*_*CF*_ = 25.0 Hz); 111.0, 112.1, 118.6, 119.3, 121.9, 122.8, 127.1, 127.4, 128.2, 128.7, 131.6 (dd, ^3^ *J*′_*CF*_ = 9.3 Hz; ^3^ *J*″_*CF*_ = 6.1 Hz); 136.2; 139.2; 161.3 (dd, ^1^ *J*_*CF*_ = 247.6 Hz; ^3^ *J*_*CF*_ = 11.7 Hz); 162.0 (dd, ^1^ *J*_*CF*_ = 247.6 Hz; ^3^ *J*_*CF*_ = 11.7 Hz); 172.9. HR-MS *m*/*z* calcd for C_26_H_24_F_2_N_2_O_2_ [(M + H)]^+^: 435.1879; found 435.1853.

**Ethyl N** ^**α**^,**N** ^**α**^ **-dibenzyl-** *L* **-tryptophanate (8).** Compound **8** was obtained starting from **2** and benzyl bromide in agreement with the procedure B. FC in *n*-hexane/ethyl acetate 1/1, R*f*: 0.47. Yellow oil (77% yield). [α]^25^_D_: + 3.36 ± 0.49 (c = 0.10, MeOH). ^1^H NMR (400 MHz, CDCl_3_): δ: 1.32 (t, 3H, C*H*_3_, *J* = 7.1 Hz); 3.14 (dd, 1H, C*H*_*2a*_, *J*′ = 5.6; *J*″ = 14.3 Hz); 3.44 (dd, 1H, C*H*_*2b*_, *J*′ = 6.7; J″ = 14.2 Hz); 3.64 (d, 2H, C*H*_2_, *J* = 13.8 Hz); 3.84 (dd, 1H, C*H*, *J*′ = 5.8; J″ = 8.9 Hz); 4.08 (d, 2H, C*H*_2_, *J* = 13.8 Hz); 4.13–4.19 (m, 1H, C*H*_*2a*_); 4.25–4.33 (m, 1H, C*H*_*2b*_); 6.92 (s, 1H, aryl); 7.01 (t, 1H, aryl, *J* = 7.5 Hz); 7.18 (t, 2H, aryl, *J* = 7.7 Hz); 7.28–7.39 (m, 11H, aryl); 7.94 (bs, 1H, N*H*). ^13^C NMR (100 MHz, CDCl_3_) δ: 14.6, 26.2, 54.7, 60.1, 61.4, 110.9, 112.5, 118.8, 119.2, 121.8, 122.8, 127.0, 127.5, 128.5, 128.9, 139.7, 172.5. HR-MS *m*/*z* calcd for C_27_H_28_N_2_O_2_ [(M + H)]^+^: 413.2224; found 413.2206.

**Benzyl N** ^**α**^,**N** ^**α**^ **-dibenzyl-** *L* **-tryptophanate (9).** Final derivative **9** was synthesized from **3** and benzyl bromide according to the general procedure B. FC in *n*-hexane/ethyl acetate 8/2, R*f*: 0.47. Yellowish oil (74% yield). [α]^25^_D_: + 1.08 ± 0.01 (c = 0.10, MeOH) ^1^H NMR (400 MHz, CDCl_3_): 3.17 (dd, 1H, C*H*_*2a*_, *J*′ = 5.5; J″ = 14.3 Hz); 3.48 (dd, 1H, C*H*_*2b*_, *J*′ = 6.8; J″ = 14.4 Hz); 3.63 (d, 2H, C*H*_2_, *J* = 13.9 Hz); 3.93 (dd, 1H, C*H*, *J*′ = 5.7; J″ = 8.8 Hz); 4.07 (d, 2H, C*H*_2_, *J* = 13.9 Hz); 5.13 (d, 1H, C*H*_*2a*_, *J* = 12.3 Hz); 5.29 (d, 1H, C*H*_*2b*_, *J* = 12.3 Hz); 6.87 (s, 1H, aryl); 7.03 (t, 1H, aryl, *J* = 7.5 Hz); 7.19–7.23 (m, 2H, aryl); 7.27–7.41 (m, 15H, aryl); 7.89 (bs, 1H, N*H*). ^13^C NMR (100 MHz, CDCl_3_) δ: 26.1, 54.7, 61.5, 66.0, 111.0, 112.0, 118.8, 119.3, 121.9, 122.8, 127.0, 127.5, 128.3, 128.47, 128.54, 128.9, 136.2, 139.6, 172.4. HR-MS *m*/*z* calcd for C_32_H_30_N_2_O_2_ [(M + H)]^+^: 475.2380; found 475.2397.

**Methyl N** ^**α**^ **-(2,4-difluorobenzyl)-N** ^**α**^ **-(4-ethoxybenzyl)-** *L* **-tryptophanate (10).** Compound **10** was obtained from **4** and 2,4-difluorobenzyl bromide applying the general procedure A. FC in *n*-hexane/ethyl acetate 7/3, R*f*: 0.45. Yellowish oil (72% yield). [α]^25^_D_: −74.00 ± 0.07 (c = 0.10, MeOH). ^1^H NMR (400 MHz, CDCl_3_): δ: 1.44 (t, 3H, C*H*_3_, *J* = 7.0 Hz); 3.10 (dd, 1H, C*H*_*2a*_, *J*′ = 6.2; J″ = 14.4 Hz); 3.38 (dd, 1H, C*H*_*2b*_, *J*′ = 8.6; J″ = 14.3 Hz); 3.54 (d, 1H, C*H*_*2a*_, *J* = 13.6 Hz); 3.72 (t, 4H, C*H*_3_ and C*H*_*2b*_, *J* = 6.0 Hz); 3.80 (dd, 1H, C*H*, *J*′ = 6.4; J″ = 8.4 Hz); 3.86–3.95 (m, 2H, C*H*_2_); 4.02 (d, 1H, C*H*_*2a*_, *J* = 6.9 Hz); 4.05 (d, 1H, C*H*_*2b*_, *J* = 6.9 Hz); 6.70–6.75 (m, 2H, aryl); 6.80 (d, 2H, aryl, *J* = 8.5 Hz); 6.93 (s, 1H, aryl); 6.99 (t, 1H, aryl, *J* = 7.4 Hz); 7.16–7.21 (m, 4H, aryl); 7.23–7.28 (m, 1H, aryl); 7.35 (d, 1H, aryl, *J* = 6.9 Hz); 7.94 (bs, 1H, N*H*). ^13^C NMR (100 MHz, CDCl_3_) δ: 14.9, 26.0, 46.9, 51.1, 54.2, 61.5, 63.4, 103.4 (t, ^2^ *J*_*CF*_ = 25.5 Hz); 111.0, 112.2, 114.2, 118.6, 119.2, 121.9, 131.9, 122.7, 127.4, 129.9, 131.0, 131.6 (dd, ^3^ *J*′_*CF*_ = 9.3 Hz, ^3^ *J*″_*CF*_ = 6.1 Hz); 136.1, 158.1, 161.2 (dd, ^1^ *J*_*CF*_ = 247.6 Hz; ^3^ *J*_*CF*_ = 11.7 Hz); 161.9 (dd, ^1^ *J*_*CF*_ = 247.6; ^3^ *J*_*CF*_ = 11.7 Hz); 172.9. HR-MS *m*/*z* calcd for C_28_H_28_F_2_N_2_O_3_ [(M + H)]^+^: 479.2141; found 479.2120.

**(S)-2-(Dibenzylamino)-3-(1H-indol-3-yl)­propanoic acid (11).** Obtained from **8** in accordance with the general procedure C. Precipitated from dichloromethane/diethyl ether. White solid (92% yield). ^1^H NMR (400 MHz, CD_3_OD) δ: 3.19 (dd, 1H, C*H*_*2a*_, *J*′ = 4.0, *J*″ = 12.0 Hz); 3.45 (dd, 1H, C*H*_*2b*_, *J*′ = 8.0, *J*″ = 16.0 Hz); 3.79 (d, 2H, C*H*_2_, *J* = 12.0 Hz); 3.86 (t, 1H, C*H*, *J* = 8.2 Hz); 3.98 (d, 2H, C*H*_2_, *J* = 12.0); 6.89 (t, 1H, aryl, *J* = 8.0 Hz); 7.04 (s, 1H, aryl); 7.09 (t, 1H, aryl, *J* = 8.0 Hz); 7.19 (d, 1H, aryl, *J* = 8.0 Hz); 7.25–7.30 (m, 10H, aryl); 7.36 (d, 1H, aryl, *J* = 8.0 Hz). HR-MS *m*/*z* calcd for C_25_H_25_N_2_O_2_ [(M + H)^+^]: 385.1911; found 385.1920.

**(S)-2-(Dibenzylamino)-3-(1H-indol-3-yl)-** *N* **-methylpropanamide (12).** Final compound **12** was synthesized from **11** and methylamine according to the general procedure D. FC in *n*-hexane/ethyl acetate 2/8, R*f*: 0.43. Yellowish solid (46% yield). [α]^25^_D_: + 12.02 ± 0.04 (c = 0.10, MeOH). ^1^H NMR (400 MHz, CDCl_3_): δ: 2.78 (d, 3H, C*H*_3_, *J* = 4.0 Hz); 3.25 (dd, 1H, C*H*_*2a*_, *J*′ = 3.8; J″ = 13.5 Hz); 3.53–3.63 (m, 2H, C*H* and C*H*_*2b*_); 3.79 (d, 2H, C*H*_2_, *J* = 13.8 Hz); 3.88 (d, 2H, C*H*_2_, *J* = 13.8 Hz); 6.20 (bs, 1H, N*H*); 7.06 (s, 1H, aryl); 7.09 (t, 1H, aryl, *J* = 7.2 Hz); 7.20 (t, 1H, aryl, *J* = 7.3 Hz); 7.27–7.37 (m, 11H, aryl); 7.51 (d, 1H, aryl, *J* = 7.9 Hz); 8.27 (bs, 1H, N*H*). ^13^C NMR (100 MHz, CDCl_3_) δ: 22.6, 26.0, 54.7, 63.0, 111.2, 113.2, 118.8, 119.2, 121.8, 123.3, 127.2, 127.3, 128.5, 128.7, 136.3, 139.4, 173.3. HR-MS *m*/*z* calcd for C_26_H_27_N_3_O [(M + H)]^+^: 398.2227; found 398.2209.

**(** *S* **)-N-Butyl-2-(dibenzylamino)-3-(1H-indol-3-yl)­propanamide (13).** Derivative **13** was obtained from **11** and butylamine in accordance with the general procedure D. FC in *n*-hexane/ethyl acetate 3/7, R*f*: 0.48. Yellowish powder (66% yield). [α]^25^_D_: −15.07 ± 0.15 (c = 0.10, MeOH). ^1^H NMR (400 MHz, CDCl_3_): δ: 0.8 (t, 3H, C*H*_3,_ *J* = 7.3 Hz); 1.12–1.20 (m, 2H, C*H*_2_); 1.27–1.35 (m, 2H, C*H*_2_); 3.01–3.20 (m, 3H, C*H*_2_ and C*H*_*2a*_); 3.45–3.49 (m, 2H, C*H* and C*H*_*2b*_); 3.68 (d, 2H, C*H*_2,_ *J* = 14.0 Hz); 3.74 (d, 2H, C*H*_2_, *J* = 14.0 Hz); 6.14 (bs, 1H, N*H*); 6.97–7.00 (m, 2H, aryl); 7.09 (t, 1H, aryl, *J* = 7.4 Hz); 7.11–7.22 (m, 10H, aryl); 7.26 (d, 1H, aryl, *J* = 8.0 Hz); 7.41 (d, 1H, aryl, *J* = 7.8 Hz); 7.99 (bs, 1H,N*H*). ^13^C NMR (100 MHz, CDCl_3_) δ: 13.7, 20.1, 22.5, 31.7, 38.9, 54.6, 62.8, 111.1, 113.4, 118.9, 119.2, 121.9, 123.2, 127.1, 127.3, 128.4, 128.6, 136.2, 139.6, 172.5. HR-MS *m*/*z* calcd for C_29_H_33_N_3_O [(M + H)]^+^: 440.2696; found 440.2666.

**Synthesis of methyl (S)-2-((tert-butoxycarbonyl)­amino)-3-iodopropanoate and methyl (R)-2-((tert-butoxycarbonyl)­amino)-3-iodopropanoate (14a, 14b).** Iodine (1.5 equiv) was added to a solution of P­(Ph)_3_ (1.5 equiv) in CH_2_Cl_2_ (10.0 mL) and the mixture was stirred at room temperature for 60 min, then 1.5 eq of imidazole were added. After 60 min a solution of Boc-*L*-Ser-OMe or Boc-*D*-Ser-OMe (1.0 equiv) in DCM (5.0 mL) was added and the mixture was further stirred at room temperature for 12 h. Afterward, the organic phase was added with 15 mL of 5% NaHSO_3_ aqueous solution, diluted with CH_2_Cl_2_, extracted dried over anhydrous Na_2_SO_4_, filtered, and concentrated. The crude was purified by flash chromatography using *n*-hexane and ethyl acetate (9/1) as mobile phase. Rf: 0.40. Yellowish oil (89, 92% yield). ^1^H NMR (400 MHz, CDCl_3_): δ: 1.49 (s, 9H, C*H*_3_); 3.56–3.72 (m, 2H, C*H*_2_); 3.83 (s, 3H, C*H*_3_); 4.55 (t, 1H, C*H*, *J* = 4.0 Hz); 5.37 (d, 2H, C*H*_2,_ *J* = 5.8 Hz); HR-MS *m*/*z* calcd for C_9_H_16_INO_4_ [(M + H)]^+^: 330.0197; found 330.0150.

**tert-Butyl (S)-5-(2-((tert-butoxycarbonyl)­amino)-3-methoxy-3-oxopropyl)-1H-indole-1-carboxylate (16).** Obtained from **15** and *tert*-butyl 5-bromoindole-1-carboxylate in accordance with the general procedure E. FC *n*-hexane/ethyl acetate (*ratio* 8/2, v/v). R*f*: 0.46. Whitish oil (46% yield). ^1^H NMR (400 MHz, CDCl_3_): δ: 1.41 (s, 9H, C*H*_3_); 1.62 (s, 9H, C*H*_3_); 3.07–3.20 (m, 2H, C*H*_2_); 3.65 (s, 3H, C*H*_3_); 4.56 (t, 1H, C*H, J* = 6.5 Hz); 5.16 (d, N*H*, *J* = 7.9 Hz); 6.45 (d, 1H, aryl, *J* = 3.7 Hz); 7.04 (d, 1H, aryl, *J* = 8.4 Hz); 7.28 (s, 1H, aryl); 7.54 (d, 1H, aryl, *J* = 4.4 Hz); 8.03 (d, 1H, aryl, *J* = 8.1 Hz). HR-MS *m*/*z* calcd for C_22_H_30_N_2_O_6_ [(M + H)]^+^: 419.2177; found 419.2189.

**tert-Butyl (R)-5-(2-((tert-butoxycarbonyl)­amino)-3-methoxy-3-oxopropyl)-1H-indole-1-carboxylate (17).** Obtained from **15** and *tert*-butyl 5-bromoindole-1-carboxylate in accordance with the general procedure E. FC *n*-hexane/ethyl acetate (*ratio* 8/2, v/v). R*f*: 0.46. Whitish oil (48% yield). ^1^H NMR (400 MHz, CDCl_3_): δ: 1.41 (s, 9H, C*H*_3_); 1.62 (s, 9H, C*H*_3_); 3.07–3.20 (m, 2H, C*H*_2_); 3.65 (s, 3H, C*H*_3_); 4.55 (t, 1H, C*H, J* = 6.5 Hz); 5.16 (d, N*H*, *J* = 7.9 Hz); 6.45 (d, 1H, aryl, *J* = 3.7 Hz); 7.03 (d, 1H, aryl, *J* = 8.4 Hz); 7.28 (s, 1H, aryl); 7.52 (d, 1H, aryl, *J* = 4.4 Hz); 8.03 (d, 1H, aryl, *J* = 8.1 Hz). HR-MS *m*/*z* calcd for C_22_H_30_N_2_O_6_ [(M + H)]^+^: 419.2177; found 419.2196.

**Methyl (S)-2-((tert-butoxycarbonyl)­amino)-3-(thiophen-3-yl)­propanoate (18).** Obtained from **15** and 3-bromothiophene according to the general procedure E. FC *n*-hexane/ethyl acetate (*ratio* 8/2, v/v). R*f*: 0.48. White powder (54% yield). ^1^H NMR (400 MHz, CDCl_3_): δ: 1.48 (s, 9H, C*H*_3_); 3.19–3.26 (m, 2H, C*H*_2_); 3.66 (s, 3H, C*H*_3_); 4.28 (t, 1H, C*H*, *J* = 6.5 Hz); 6.97 (d, 1H, aryl, *J* = 5.5 Hz); 7.22 (s, 1H, aryl); 7.40 (d, 1H, aryl, *J* = 3.5 Hz); HR-MS *m*/*z* calcd for C_13_H_19_NO_4_S [(M + H)]^+^: 286.1108; found 286.1133.

**Methyl (S)-2-((tert-butoxycarbonyl)­amino)-3-(thiophen-2-yl)­propanoate (19).** Synthesized from 15 and 2-bromothiophene according to the general procedure E. FC *n*-hexane/ethyl acetate (*ratio* 8/2, v/v). R*f*: 0.44. Whitish oil (53% yield). ^1^H NMR (400 MHz, CDCl_3_): δ: 1.46 (s, 9H, C*H*_3_); 3.32–3.43 (m, 2H, C*H*_2_); 3.76 (s, 3H, C*H*_3_); 4.60 (bs, 1H, C*H*); 5.16 (bs, N*H*); 6.81 (d, 1H, aryl, *J* = 3.9 Hz); 6.95 (d, 1H, aryl, *J* = 5.1 Hz); 7.18 (d, 1H, aryl, *J* = 5.8 Hz). HR-MS *m*/*z* calcd for C_13_H_19_NO_4_S [(M + H)]^+^: 285.1035; found 285.1055.

**tert-Butyl (S)-2-(2-((tert-butoxycarbonyl)­amino)-3-methoxy-3-oxopropyl)-1H-indole-1-carboxylate (20).** Intermediate **20** was obtained from **15** and *tert*-butyl 2-bromo-1H-indole-1-carboxylate in agreement with the general procedure E. FC *n*-hexane/ethyl acetate (*ratio* 7/3, v/v). R*f*: 0.45. Whitish oil (43% yield). ^1^H NMR (400 MHz, CD_3_OD): δ: 1.43 (s, 9H, C*H*_3_); 1.60 (s, 9H, C*H*_3_); 3.16 (dd, 1H, C*H*_*2a,*_ *J*′ = 7.4 Hz and *J*″ = 13.6 Hz); 3.32 (dd, 1H, C*H*_*2b,*_ *J*′ = 7.4 Hz and *J*″ = 13.6 Hz); 3.65 (s, 3H, C*H*_3_); 4.47 (t, 1H, C*H*, *J =* 8.2 Hz); 6.32 (s, 1H, aryl); 6.95 (t, 1H, aryl, *J* = 8.7 Hz); 7.10 (t, 1H, aryl, *J* = 8.6 Hz); 7.45 (d, 1H, aryl, *J* = 8.6 Hz); 7.57 (d, 1H, aryl, *J* = 8.6 Hz). HR-MS *m*/*z* calcd for C_22_H_30_N_2_O_6_ [(M + H)]^+^: 419.2177; found 419.2198.

**Methyl (S)-2-((tert-butoxycarbonyl)­amino)-3-(pyridin-2-yl)­propanoate (21).** Obtained from **15** and 2-bromopyridine according to the general procedure E. FC *n*-hexane/ethyl acetate (*ratio* 8/2, v/v). R*f*: 0.46. Whitish oil (50% yield). ^1^H NMR (400 MHz, CDCl_3_): δ: 1.44 (s, 9H, C*H*_3_); 3.27–3.37 (m, 2H, C*H*_2_); 3.71 (s, 3H, C*H*_3_); 4.70 (t, 1H, C*H, J =* 5.4 Hz); 5.87 (bs, N*H*); 7.15–7.18 (m, 2H, aryl); 7.62 (t, 1H, aryl, *J* = 9.5 Hz); 8.52 (d, 1H, aryl, *J* = 4.4 Hz). HR-MS *m*/*z* calcd for C_14_H_20_N_2_O_4_ [(M + H)]^+^: 281.1496; found 281.1408.

**Methyl (R)-2-((tert-butoxycarbonyl)­amino)-3-(pyridin-2-yl)­propanoate (22).** Obtained from **15** and 2-bromopyridine according to the general procedure E. FC *n*-hexane/ethyl acetate (*ratio* 7/3, v/v). R*f*: 0.44. Whitish oil (52% yield). ^1^H NMR (400 MHz, CDCl_3_): δ: 1.44 (s, 9H, C*H*_3_); 3.25–3.37 (m, 2H, C*H*_2_); 3.70 (s, 3H, C*H*_3_); 4.70 (t, 1H, C*H, J =* 5.4 Hz); 5.88 (bs, N*H*); 7.15–7.20 (m, 2H, aryl); 7.62 (t, 1H, aryl, *J* = 9.5 Hz); 8.51 (d, 1H, aryl, *J* = 4.4 Hz). HR-MS *m*/*z* calcd for C_14_H_20_N_2_O_4_ [(M + H)]^+^: 281.1496; found 281.1411.

**Methyl (S)-2-amino-3-(1H-indol-5-yl)­propanoate (23.1).** Obtained from **16** according to the general procedure F. Precipitated from dichloromethane/diethyl ether. Whitish powder (92% yield). ^1^H NMR (400 MHz, CDCl_3_): δ: 3.14–3.24 (m, 2H, C*H*_2_); 3.73 (s, 3H, C*H*_3_); 4.61 (bs, 1H, C*H*); 4.99 (bs, N*H*); 6.53 (d, 1H, aryl, *J* = 3.6 Hz); 7.08 (d, 1H, aryl, *J* = 8.5 Hz); 7.33 (s, 1H, aryl); 7.59 (d, 1H, aryl, J= 3.6 Hz); 8.07 (d, 1H, aryl, *J* = 8.1 Hz). HR-MS *m*/*z* calcd for C_12_H_14_N_2_O_2_ [(M + H)]^+^: 219.1128; found 219.1137.

**Methyl (** *S* **)-2-(dibenzylamino)-3-(1H-indol-5-yl)­propanoate (23).** Final derivative **23** was synthesized from **23.1** and benzyl bromide according to the general procedure B. FC in dichloromethane/ethyl acetate 9/1, R*f*: 0.48. Colorless oil (65% yield). [α]^25^_D_: −5.04 ± 0.07 (c = 0.10, MeOH). ^1^H NMR (400 MHz, CD_3_OD): δ: 3.07 (dd, 1H, C*H*_*2a*_, *J*′ = 7.8; *J*″ = 13.8 Hz); 3.18 (dd, 1H, C*H*_*2b*_, *J*′ = 7.9; *J*″ = 13.7 Hz); 3.54 (d, 2H, C*H*_2_, *J* = 13.9 Hz); 3.65–3.71 (m, 4H, C*H*_3_ and C*H*); 3.96 (d, 2H, C*H*_3_, *J* = 13.8 Hz); 6.34 (d, 1H, aryl, *J* = 304 Hz); 6.77 (d, 1H, aryl, *J* = 8.3 Hz); 7.20–7.26 (m, 13H, aryl). ^13^C NMR (100 MHz, CD_3_OD) δ: 35.5, 50.1, 54.1, 63.2, 100.6, 110.4, 120.3, 122.6, 124.3, 126.5, 127.7, 128.1, 128.5, 139.4, 173.2. HR-MS *m*/*z* calcd for C_26_H_26_N_2_O_2_ [(M + H)]^+^: 399.2067; found 399.2058.

**Methyl (R)-2-amino-3-(1H-indol-5-yl)­propanoate (24.1).** Synthesized from **17** according to the general procedure F. Precipitated from dichloromethane/diethyl ether. Whitish powder (92% yield). ^1^H NMR (400 MHz, CDCl_3_): δ: 3.14–3.24 (m, 2H, C*H*_2_); 3.73 (s, 3H, C*H*_3_); 4.61 (bs, 1H, C*H*); 4.99 (bs, N*H*); 6.53 (d, 1H, aryl, *J* = 3.6 Hz); 7.08 (d, 1H, aryl, *J* = 8.5 Hz); 7.33 (s, 1H, aryl); 7.59 (d, 1H, aryl, *J* = 3.6 Hz); 8.07 (d, 1H, aryl, *J* = 8.1 Hz). HR-MS *m*/*z* calcd for C_12_H_14_N_2_O_2_ [(M + H)]^+^: 219.1128; found 219.1137.

**Methyl (** *R* **)-2-(dibenzylamino)-3-(1H-indol-5-yl)­propanoate (24).** Final derivative **24** was synthesized from **24.1** and benzyl bromide according to the general procedure B. FC in dichloromethane/ethyl acetate 9/1, R*f*: 0.44. Yellowish oil (52% yield). [α]^25^_D_: + 9.00 ± 0.18 (c = 0.10, MeOH). ^1^H NMR (400 MHz, CD_3_OD): δ: 3.07 (dd, 1H, C*H*_*2a*_, *J*′ = 7.8; *J*″ = 13.8 Hz); 3.18 (dd, 1H, C*H*_*2b*_, *J*′ = 7.9; *J*″ = 13.7 Hz); 3.54 (d, 2H, C*H*_2_, *J* = 13.9 Hz); 3.67–3.71 (m, 4H, C*H*_3_ and C*H*); 3.96 (d, 2H, C*H*_2_, *J* = 13.8 Hz); 6.34 (d, 1H, aryl, *J* = 304 Hz); 6.77 (d, 1H, aryl, *J* = 8.3 Hz); 7.18–7.26 (m, 13H, aryl). ^13^C NMR (100 MHz, CD_3_OD) δ: 35.5, 50.1, 54.1, 63.2, 100.6, 110.4, 120.3, 122.6, 124.3, 126.5, 127.7, 128.1, 128.5, 139.4, 173.2. HR-MS *m*/*z* calcd for C_26_H_26_N_2_O_2_ [(M + H)]^+^: 399.2067; found 399.2047.

**Methyl (S)-2-amino-3-(thiophen-3-yl)­propanoate (25.1).** Synthesized from **18** according to the general procedure F. Precipitated from methanol/diethyl ether. R*f*: 0.46. White powder (93% yield). ^1^H NMR (400 MHz, CDCl_3_): δ: 3.28–3.33 (m, 2H, C*H*_2_); 3.86 (s, 3H, C*H*_3_); 4.34 (t, 1H, C*H*, *J* = 6.7 Hz); 7.01 (d, 1H, aryl, *J* = 4.8 Hz); 7.28 (s, 1H, aryl); 7.47 (d, 1H, aryl, *J* = 3.0 Hz). HR-MS *m*/*z* calcd for C_8_H_11_NO_2_S [(M + H)]^+^: 186.0583; found 186.0552.

**Methyl (** *S* **)-2-(dibenzylamino)-3-(thiophen-3-yl)­propanoate (25).** Compound **25** was obtained from **25.1** and benzyl bromide applying the general procedure B. FC in dichloromethane/ethyl acetate 9.5/0.5, R*f*: 0.32. Whitish oil (60% yield). [α]^25^_D_: + 16.07 ± 0.38 (c = 0.10, MeOH). ^1^H NMR (400 MHz, CDCl_3_); 2.94–3.08 (m, 2H, C*H*_2_); 3.47 (d, 2H, C*H*_2_, *J* = 13.8 Hz); 3.59 (t, 1H, C*H*, *J* = 7.6 Hz); 3.67 (s, 3H, C*H*_3_); 3.86 (d, 2H, C*H*_2_, *J* = 13.8 Hz); 6.64 (d, 1H, aryl, *J* = 4.8 Hz); 6.78 (s, 1H, aryl); 7.11–7.22 (m, 11H, aryl). ^13^C NMR (100 MHz, CDCl_3_) δ: 30.3, 51.2, 54.5, 61.8, 122.0, 125.0, 127.0, 128.2, 128.7, 128.8, 139.3, 172.7. HR-MS *m*/*z* calcd for C_22_H_23_NO_2_S [(M + H)]^+^: 366.1522; found 366.1508.

**Methyl (S)-2-amino-3-(thiophen-2-yl)­propanoate (26.1).** Synthesized from **19** according to the general procedure F. Precipitated from dichloromethane/diethyl ether. Whitish powder (87% yield). ^1^H NMR (400 MHz, CD_3_OD): δ: 3.51 (d, 2H, C*H*_2,_ *J* = 6.1 Hz); 3.88 (s, 3H, C*H*_3_); 4.37 (t, 1H, C*H, J* = 6.0 Hz); 7.01 (d, 1H, aryl, *J* = 3.0 Hz); 7.04 (t, 1H, aryl, *J* = 5.1 Hz); 7.39 (d, 1H, aryl, *J* = 5.1 Hz). HR-MS *m*/*z* calcd for C_8_H_11_NO_2_S [(M + H)]^+^: 186.0583; found 186.0577.

**Methyl (S)-2-(dibenzylamino)-3-(thiophen-2-yl)­propanoate (26).** Compound **26** was obtained from **26.1** and benzyl bromide applied the general procedure B. FC in *n*-hexane/diethyl ether 8/2, R*f*: 0.42. Whitish oil (64% yield). [α]^25^_D_: + 44.00 ± 0.73 (c = 0.10, MeOH). ^1^H NMR (400 MHz, CDCl_3_); δ: 3.21 (dd, 1H, C*H*_*2a*_, *J*′ = 7.4, *J*″ = 9.6 Hz); 3.37 (dd, 1H, C*H*_*2b*_, *J*′ = 7.4, *J*″ = 9.6 Hz); 3.57 (d, 2H, C*H*_2_, *J* = 13.9 Hz); 3.69 (t, 1H, C*H*, *J* = 13.6 Hz); 3.78 (s, 3H, C*H*_3_); 3.96 (d, 2H, C*H*_2_, *J* = 13.9 Hz); 6.70 (d, 1H, aryl, *J* = 3.0 Hz); 6.91 (t, 1H, aryl, *J* = 4.9 Hz); 7.14 (d, 1H, aryl, *J* = 5.6 Hz); 7.22–7.30 (m, 10H, aryl). ^13^C NMR (100 MHz, CDCl_3_) δ: 30.1, 51.3, 54.7, 63.0, 123.7, 125.9, 126.6, 127.0, 128.2, 128.8, 139.1, 140.5. HR-MS *m*/*z* calcd for C_22_H_23_NO_2_S [(M + H)]^+^: 366.1522; found 366.1521.

**Methyl** *L* **-tryptophanate (27.1).** Obtained from **20** according to the general procedure F. Precipitated from methanol/diethyl ether. White powder (95% yield). ^1^H NMR (400 MHz, CD_3_OD): δ: 3.38 (dd, 1H, C*H*_*2a,*_ *J*′ = 7.6 Hz and *J*″ = 13.5 Hz); 3.32 (dd, 1H, C*H*_*2b,*_ *J*′ = 7.6 Hz and *J*″ = 13.5 Hz); 3.87 (s, 3H, C*H*_3_); 4.42 (t, 1H, C*H*, *J =* 7.9 Hz); 6.37 (s, 1H, aryl); 7.01 (t, 1H, aryl, *J* = 8.7 Hz);); 7.11 (t, 1H, aryl, *J* = 8.4 Hz); 7.35 (d, 1H, aryl, *J* = 7.8 Hz); 7.50 (d, 1H, aryl, *J* = 8.4 Hz). HR-MS *m*/*z* calcd for C_12_H_14_N_2_O_2_ [(M + H)]^+^: 219.1128; found 219.1146.

**Methyl Nα,Nα-dibenzyl-** *L* **-tryptophanate (27).** Compound **27** was obtained from **27.1** and benzyl bromide according to the general procedure B. FC dichloromethane/ethyl acetate (*ratio* 9.5/0.5, v/v). R*f*: 0.46. Whitish oil (63% yield). [α]^25^_D_: −37.06 ± 0.09 (c = 0.10, MeOH). ^1^H NMR (400 MHz, CD_3_OD): δ: 3.09 (d, 2H, C*H*_2_, *J* = 6.6 Hz); 3.44 (d, 2H, C*H*_2_, *J* = 13.8 Hz); 3.65 (s, 3H, C*H*_3_); 3.71 (t, 1H, C*H*, *J* = 7.9 Hz); 3.81 (d, 2H, C*H*_2_, *J* = 13.8 Hz); 6.86 (t, 1H, aryl, *J* = 7.1 Hz); 6.93 (t, 1H, aryl, *J* = 7.7 Hz); 7.07–7.13 (m, 11H, aryl); 7.32 (d, 1H, aryl, *J* = 7.7 Hz). ^13^C NMR (100 MHz, CD_3_OD) δ: 28.0, 50.3, 54.1, 61.2, 100.0, 110.2, 118.5, 119.0, 120.1, 126.6, 127.8, 128.5, 135.7, 136.3, 139.2, 172.7. HR-MS *m*/*z* calcd for C_26_H_26_N_2_O_2_ [(M + H)]^+^: 399.2067; found 399.2079.

**Methyl (S)-2-amino-3-(pyridin-2-yl)­propanoate (28.1).** Intermediate **28.1** was synthesized from **21** according to the general procedure F. Precipitated from dichloromethane/diethyl ether. Whitish powder (95% yield). ^1^H NMR (400 MHz, CD_3_OD): δ: 3.48–3.56 (m, 2H, C*H*_2_); 3.81 (s, 3H, C*H*_3_); 4.59 (t, 1H, C*H, J =* 5.7 Hz); 7.51–7.58 (m, 2H, aryl); 8.03 (t, 1H, aryl, *J* = 9.5 Hz); 8.62 (d, 1H, aryl, *J* = 5.2 Hz). HR-MS *m*/*z* calcd for C_9_H_12_N_2_O_2_ [(M + H)]^+^: 181.0972; found 181.0998.

**Methyl (S)-2-(dibenzylamino)-3-(pyridin-2-yl)­propanoate (28).** Derivative **28** was obtained from **28.1** and benzyl bromide in accordance with the general procedure B. FC in dichloromethane/ethyl acetate 8/2, R*f*: 0.42. White oil (60% yield). [α]^25^_D_: −14.08 ± 0.17 (c = 0.10, MeOH). ^1^H NMR (400 MHz, CDCl_3_); δ 3.20 (dd, 1H, C*H*_*2a*_, *J*′ = 9.0, *J*″ = 14.2 Hz); 3.28 (dd, 1H, C*H*_*2b*_ *J*′ = 8.2, *J*″ = 14.3 Hz); 3.61 (d, 2H, C*H*_2_, *J* = 14.4 Hz); 3.79 (s, 3H, C*H*_3_); 3.96 (d, 2H, C*H*_2_, *J* = 13.9 Hz); 4.01 (dd, 1H, C*H*, *J*′ = 6.4, *J*″ = 8.8 Hz); 7.04 (d, 1H, aryl, *J* = 7.0 Hz); 7.13–7.19 (m, 5H, aryl); 7.21–7.29 (m, 6H, aryl); 7.57 (t, 1H, *J* = 7.6 Hz); 8.44 (d, 1H, aryl, *J* = 4.7 Hz). ^13^C NMR (100 MHz, CDCl_3_) δ: 38.1, 51.2, 54.6, 61.3, 121.2, 123.9, 126.9, 128.1, 128.8, 135.9, 139.3, 149.1, 158.6, 172.9. MS *m*/*z* calcd for C_23_H_24_N_2_O_2_ [(M + H)] ^+^: 361.1911; found 361.1898.

**Methyl (R)-2-amino-3-(pyridin-2-yl)­propanoate (29.1).** Obtained from **22** according to the procedure F. Precipitated from dichloromethane/diethyl ether. Whitish powder (97% yield). ^1^H NMR (400 MHz, CD_3_OD): δ: 3.47–3.59 (m, 2H, C*H*_2_); 3.80 (s, 3H, C*H*_3_); 4.60 (t, 1H, C*H, J =* 5.7 Hz); 7.51–7.59 (m, 2H, aryl); 8.03 (t, 1H, aryl, *J* = 9.5 Hz); 8.61 (d, 1H, aryl, *J* = 5.2 Hz). HR-MS *m*/*z* calcd for C_9_H_12_N_2_O_2_ [(M + H)]^+^: 181.0972; found 181.0978.

**Methyl (R)-2-(dibenzylamino)-3-(pyridin-2-yl)­propanoate (29).** Compound **29** was synthesized from **29.1** and benzyl bromide applying the general procedure B. FC in dichloromethane/ethyl acetate 1/1, R*f*: 0.44. Whitish oil (58% yield). [α]^25^_D_: + 18.20 ± 0.67 (c = 0.10, MeOH). ^1^H NMR (400 MHz, CDCl_3_); δ 3.21 (dd, 1H, C*H*_*2a*_, *J*′ = 9.0, *J*″ = 14.2 Hz); 3.29 (dd, 1H, C*H*_*2b*_ *J*′ = 8.2, *J*″ = 14.3 Hz); 3.61 (d, 2H, C*H*_2_, *J* = 14.4 Hz); 3.79 (s, 3H, C*H*_3_); 3.97 (d, 2H, C*H*_2_, *J* = 13.9 Hz); 4.01 (dd, 1H, C*H*, *J*′ = 6.4, *J*″ = 8.8 Hz); 7.04 (d, 1H, aryl, *J* = 7.0 Hz); 7.13–7.19 (m, 5H, aryl); 7.21–7.29 (m, 6H, aryl); 7.57 (t, 1H, *J* = 7.6 Hz); 8.44 (d, 1H, aryl, *J* = 4.7 Hz). ^13^C NMR (100 MHz, CDCl_3_) δ: 38.1, 51.2, 54.6, 61.3, 121.2, 123.9, 126.9, 128.1, 128.8, 135.9, 139.3, 149.1, 158.6, 172.9. HR-MS *m*/*z* calcd for C_23_H_24_N_2_O_2_ [(M + H)] ^+^: 361.1911; found 361.1903.

**(S)-2-(Dibenzylamino)-3-(pyridin-2-yl)­propanoic acid (30.1) (S)-2-(dibenzylamino)-3-(1H-indol-5-yl)-** *N* **-methylpropanamide (30).** Final amidic derivative **30** was synthesized from **30.1** and methylamine according to the general procedure D. FC in dichloromethane/ethyl acetate 8/2, R*f*: 0.47. Yellowish oil (51% yield). [α]^25^_D_: + 24.00 ± 0.12 (c = 0.10, MeOH) ^1^H NMR (400 MHz, CD_3_OD): δ: 2.68 (s, 3H, C*H*_3_); 3.10–3.15 (m, 1H, C*H*_*2a*_); 3.21–3.26 (m, 1H, C*H*_*2b*_); 3.55 (t, 1H, C*H*, *J* = 7.3 Hz); 3.66 (d, 2H, C*H*_2_, *J* = 14.4 Hz); 3.90 (d, 2H, C*H*_2_, *J* = 14.4 Hz); 6.36 (d, 1H, aryl, *J* = 3.1 Hz); 6.90 (d, 1H, aryl, *J* = 3.8 Hz); 7.17–7.25 (m, aryl, 10H); 7.28 (d, 1H, aryl, *J* = 4.0 Hz); 7.33 (s, 1H, aryl). ^13^C NMR (100 MHz, CD_3_OD) δ: 24.5, 34.5, 54.3, 64.9, 100.6, 110.4, 120.3, 122.6, 124.3, 126.5, 127.8, 128.2, 128.4, 128.9, 134.1, 135.9, 139.8, 174.2. HR-MS *m*/*z* calcd for C_26_H_26_N_2_O_2_ [(M + H)]^+^: 398.1994; found 398.1984

**(S)-2-(Dibenzylamino)-3-(1H-indol-5-yl)­propanoic acid (31.1).** Obtained from **26** in accordance to the general procedure C. Precipitated from dichloromethane/diethyl ether. White powder (94% yield). ^1^H NMR (400 MHz, CD_3_OD): δ: 3.02 (dd, 1H, C*H*_*2a,*_ *J*′ = 7.4 Hz and *J*″ = 13.3 Hz); 3.16 (dd, 1H, C*H*_*2b,*_ *J*′ = 7.4 Hz and *J*″ = 13.3 Hz); 3.57 (t, 1H, C*H*, *J =* 8.2 Hz); 3.72 (d, 2H, C*H*_2_, *J =* 14.1 Hz); 3.93 (d, 2H, C*H*_2_, *J =* 14.1 Hz); 6.83 (d, 1H, aryl, *J* = 8.6 Hz); 7.05–7.10 (m, 13H, aryl); 7.20 (d, 1H, aryl, *J* = 7.7 Hz); 7.25 (s, 1H, aryl). HR-MS *m*/*z* calcd for C_26_H_26_N_2_O_2_ [(M + H)]^+^: 398.1994; found 398.1978.

**(** *S* **)-2-(Dibenzylamino)-3-(thiophen-2-yl)­propanoic acid (31).** Final compound **31** was synthesized from **31.1** and methylamine applying the general procedure D. FC in dichloromethane/ethyl acetate 8/2, R*f*: 0.45. Yellow oil (48% yield). [α]^25^_D_: −8.00 ± 0.07 (c = 0.10, MeOH). ^1^H NMR (400 MHz, CD_3_OD): δ: 2.74 (s, 3H, C*H*_3_); 3.25–3.30 (m, 1H, C*H*_*2a*_); 3.36–3.40 (m, 1H, C*H*_*2b*_); 3.48 (dd, 1H, C*H*, *J*′ = 6.1, *J*″ = 9.1 Hz); 3.67 (d, 2H, C*H*_2_, *J* = 13.9 Hz); 3.84 (d, 2H, C*H*_2_, *J* = 13.9 Hz); 6.80 (d, 1H, aryl, *J* = 2.8 Hz); 6.90 (dd, 1H, aryl, *J*′ = 3.4, *J*″ = 5.1 Hz); 7.20 (dd, 1H, aryl, *J*′ = 2.0, *J*″ = 5.2 Hz); 7.21–7.24 (m, 2H, aryl); 7.27–7.33 (m, 8H, aryl). ^13^C NMR (100 MHz, CD_3_OD) δ: 24.7, 27.6, 54.3, 64.7, 123.3, 125.5, 126.2, 126.7, 127.9, 128.5, 139.3, 141.2, 173.3. HR-MS *m*/*z* calcd for C_22_H_24_N_2_OS [(M + H)]^+^: 365.1682; found 365.1674.

**(S)-2-(Dibenzylamino)-3-(thiophen-2-yl)­propanoic acid (32.1).** Obtained from **28** applying the general procedure C. Precipitated from dichloromethane/diethyl ether. White powder (94% yield). ^1^H NMR (400 MHz, CD_3_OD): δ: 3.25 (dd, 1H, C*H*_*2a*_, *J*′ = 7.6 Hz and *J*″ = 14.5 Hz); 3.39 (dd, 1H, C*H*_*2b*_, *J*′ = 7.6 Hz and *J*″ = 14.5 Hz); 3.73 (d, 2H, C*H*_2_, *J* = 13.6 Hz); 3.82 (t, 1H, C*H, J* = 7.0 Hz); 3.98 (d, 2H, C*H*_2_, *J* = 13.8 Hz); 7.23 (d, 1H, aryl, *J* = 3.6 Hz); 7.45 (t, 1H, aryl, *J* = 5.0 Hz); 7.55 (d, 1H, aryl, *J* = 5.0 Hz); 7.58–7.67 (m, 9H, aryl); 7.73 (t, 1H, aryl, *J* = 8.4 Hz). HR-MS *m*/*z* calcd for C_22_H_23_NO_2_S [(M + H)]^+^: 351.1293; found 351.1209.

**(** *S* **)-2-(Dibenzylamino)-** *N* **-methyl-3-(pyridin-2-yl)­propanamide (32).** Compound **32** was synthesized from **32.1** according to the general procedure D. FC in dichloromethane/ethyl acetate 8/2, R*f*: 0.47. Yellowish oil (47% yield). [α]^25^_D_: −27.05 ± 0.42 (c = 0.10, MeOH). ^1^H NMR (400 MHz, CD_3_OD): δ: 2.75 (s, 3H, C*H*_3_); 3.20–3.32 (m, 2H, C*H*_2_); 3.65 (d, 2H, C*H*_2_, *J* = 14.0 Hz); 3.76 (t, 1H, C*H*, *J* = 6.8 Hz); 3.87 (d, 2H, C*H*_2_, *J* = 14.0 Hz); 7.16–7.30 (m, 12H, aryl); 7.73 (t, 1H, aryl, *J* = 7.7 Hz); 8.42 (d, 1H, aryl, *J* = 4.9 Hz). ^13^C NMR (100 MHz, CD_3_OD) δ: 24.7, 35.8, 54.3, 62.7, 121.6, 124.5, 126.7, 127.9, 128.4, 136.8, 139.3, 148.2, 159.2, 173.4). HR-MS *m*/*z* calcd for C_23_H_25_N_3_O [(M + H)]^+^: 360.2070; found 360.2061.

**Methyl benzyl-** *L* **-histidinate (33).** Obtained from *L*-Histidine methyl ester dihydrochloride and benzaldehyde applying the general procedure A. FC *n*-hexane/ethyl acetate (*ratio* 2/8, v/v). R*f*: 0.46. White powder (61% yield). ^1^H NMR (400 MHz, CD_3_OD); δ: 2.93 (d, 2H, C*H*_2_, *J* = 5.8 Hz); 3.64 (s, 3H, C*H*_3_); 3.83 (t, 1H, C*H*, *J* = 6.1 Hz); 6.74 (s, 1H, aryl); 7.17 (s, 1H, aryl). HR-MS *m*/*z* calcd for C_14_H_17_N_3_O_2_ [(M + H)^+^]: 260.1394; found 260.1356.

**Methyl N** ^**α**^,**N** ^**α**^ **-dibenzyl-** *L* **-histidinate (34).** Final compound **34** was synthesized from **33** and benzyl bromide according to the general procedure B. FC in *n*-hexane/ethyl acetate 1/1, R*f*: 0.42. Yellowish oil (23% yield). [α]^25^_D_: + 7.04 ± 0.22 (c = 0.10, MeOH). ^1^H NMR (400 MHz, CDCl_3_): δ: 2.93 (dd, 1H, C*H*_*2a*_, *J*′ = 7.5; *J*″ = 15.1 Hz); 3.03 (dd, 1H, C*H*_*2b*_, *J*′ = 6.9; *J*″ = 14.7 Hz); 3.49 (d, 2H, C*H*_2,_ *J* = 13.8 Hz); 3.59 (t, 1H, C*H*, *J* = 7.3 Hz); 3.70 (s, 3H, C*H*_3_); 3.84 (d, 2H, C*H*_2_, *J* = 13.8 Hz); 5.70 (bs, 1H, N*H*); 6.61 (s, 1H, aryl); 7.15–7.23 (m, 10H, aryl); 7.36 (s, 1H, aryl). ^13^C NMR (100 MHz, CDCl_3_) δ: 26.5, 51.5, 54.8, 61.3, 120.7, 127.2, 128.4, 128.9, 131.2, 134.1, 139.0, 172.9. HR-MS *m*/*z* calcd for C_21_H_23_N_3_O_2_ [(M + H)]^+^: 350.1863; found 350.1852.

**(** *S* **)-1,3-Dibenzyl-4-(2-(dibenzylamino)-3-methoxy-3-oxopropyl)-1H-imidazol-3-ium (35)** Final compound **35** was synthesized from **33** and benzyl bromide according to the general procedure B. FC in *n*-hexane/ethyl acetate 1/1, R*f*: 0.42. Yellowish oil (28% yield). [α]^25^_D_: + 42.10 ± 0.28 (c = 0.10, MeOH). ^1^H NMR (400 MHz, CDCl_3_): δ: 2.72–2.83 (m, 2H, C*H*_2_); 3.47–3.52 (m, 3H, C*H*_2_ and C*H*); 3.74 (d, 2H, C*H*_2,_ *J* = 13.6 Hz); 3.79 (s, 3H, C*H*_3_); 5.05 (s, 2H, C*H*_2_); 5.43 (d, 1H, C*H*_*2a,*_ *J* = 14.5 Hz); 5.52 (d, 1H, C*H*_*2b,*_ *J* = 14.5 Hz); 6.43 (s, 1H, aryl); 7.09–7.12 (m, 6H, aryl); 7.28–7.30 (m, 9H, aryl); 7.45 (s, 5H, aryl); 10.98 (s, 1H, aryl). ^13^C NMR (100 MHz, CDCl_3_) δ: 24.6, 51.0, 51.9, 53.3, 54.8, 58.6, 119.7, 127.76, 127.81, 128.6, 128.8, 128.9, 129.2, 129.4, 129.47, 129.54, 132.0, 132.3, 133.1, 137.9, 138.1, 171.0. IT-MS *m*/*z* calcd for C_35_H_36_N_3_O_2_^+^ [(M + H)]^+^: 530.2802; found 530.2762.

**Calcium microfluorimetric assay and IC**_**50**_ **determination.** TRPM8 activity was monitored in HEK293 cells stably expressing rat TRPM8 using a standard Ca^2+^ microfluorimetric assay. Cells were loaded for 30–45 min at 37 °C with a Ca^2+^-sensitive fluorescent dye (e.g., Fluo-4 AM, 2–4 μM) in HBSS-based buffer supplemented with 0.02–0.04% Pluronic F-127, washed, and incubated for an additional 15–30 min to allow de-esterification. Changes in intracellular Ca^2+^ were recorded at 37 °C using an automated fluorescence plate reader equipped with 96-well optics (excitation/emission appropriate for the dye). To obtain concentration–response curves, cells were preincubated for 5–10 min with increasing concentrations of test compounds, followed by stimulation with menthol (300 μM) as TRPM8 agonist. Fluorescence signals were normalized to the basal fluorescence recorded immediately before agonist addition and expressed as percentage of menthol-induced response in the absence of antagonist (100% response). For each independent experiment, full concentration–response curves were generated from multiple wells per condition (typically 6–8 wells per concentration), and each well contained multiple cells; wells were averaged to obtain a single mean response value per concentration. These data were fitted to a four-parameter logistic equation $\left(Y=\mathrm{Bottom}+(\mathrm{Top}-\mathrm{Bottom})/(1+10^{\left(X-\mathrm{Log}{\mathrm{IC}}_{50}\right)})\right)$ using nonlinear regression (GraphPad Prism), with all parameters left free. The quality of the fits was routinely checked by inspection of residuals and by monitoring standard goodness-of-fit metrics (R^2^,Sy.x and 95% confidence intervals for Log IC_50_ and IC_50_). IC_50_ values reported in Table 1 correspond to the mean ± SD of the IC_50_ estimates obtained from at least three independent experiments performed on different days, each based on a separate concentration–response curve. Thus, the standard deviations reflect biological and day-to-day experimental variability across independent assays rather than the uncertainty of a single curve fit. The relatively large SD observed for some compounds reflects inter experiment variability inherent to imaging based functional assays, rather than poor definition of the dose–response relationship within individual experiments.

### Cell Cultures

Melanoma cells included MEL-CAL cells, derived from subcutaneous metastases obtained from surgical specimens of patients with documented diagnosis of melanoma upon informed consent and gently doned by IRCCS “Istituto Nazionale dei Tumori”, Milan (2009), Istituto Nazionale Tumori – IRCCS “Fondazione G. Pascale,” Naples (2018) and “San Raffaele” University Hospital, Milan (2013) and already employed in other settings^56^ and (Malme-3M cells, established from a lung metastatic site; ATCC, Rockville, MD, USA). MEL-CAL cells were cultured in RPMI-1640 medium supplemented with *L* -glutamine (2 mM), penicillin (100 U/mL), streptomycin (100 μg/mL), and 10% fetal bovine serum (FBS). Malme-3M were maintained in Iscove’s Modified Dulbecco’s Medium (IMDM) supplemented with *L* -glutamine (2 mM), streptomycin (100 μg/mL), and 20% fetal bovine serum (FBS).

Prostate adenocarcinoma LNCaP cells, established from a lymph node metastasis (ATCC, Rockville, MD, USA), were cultured in RPMI-1640 medium supplemented with 10% (FBS), *L* -glutamine (2 mM), sodium pyruvate (1 mM), nonessential amino acids (0.1 mM), and streptomycin (100 U/mL).

Murine fibroblasts NIH-3T3 (ATCC), were cultured in Dulbecco’s Modified Eagle Medium (DMEM) containing 10% FBS, *L* -glutamine (2 mM), penicillin (100 U/mL), and streptomycin (100 U/mL).

The immortalized normal prostate epithelial cells PNT-2 (ATCC) were grown in RPMI-1640 medium supplemented with 10% FBS, *L* -glutamine (2 mM), sodium pyruvate (1 mM), nonessential amino acids (0.1 mM), and streptomycin (100 U/mL).

Unless otherwise specified, culture media and supplements were obtained from Gibco (Thermo Fisher Scientific, Waltham, MA, USA). All cells were maintained at 37 °C in a humidified atmosphere containing 5% CO2 and were routinely monitored for Mycoplasma contamination.

### Cell Viability Assay and IC_50_ Determination

Cell viability was assessed using the Enhanced Cell Counting Kit-8 (WST-8/CCK-8; #E-CK-A362, Elabscience, Texas, USA) according to the manufacturer’s instructions. Briefly, cells (5 × 10^3^ per well) were seeded in 96-well plates and, after adhesion, were either left untreated or treated with the indicated compounds at increasing concentrations (0.1, 1, 10, and 40 μM). After 24 or 72 h of incubation, 10 μL of CCK-8 reagent (corresponding to 1/10 of the culture volume) was added to each well and incubated for 2 h at 37 °C. Absorbance was measured at 450 nm using a microplate reader (Infinite 200, Tecan Trading AG, Switzerland). Data were analyzed and plotted using GraphPad Prism software (GraphPad Software, Boston, MA, USA) and are presented as absorbance values. Half-maximal inhibitory concentrations (IC_50_) were calculated from dose–response curves by nonlinear regression analysis (log­[inhibitor] vs normalized response) using GraphPad Prism 9 software. All experiments were performed at least in triplicate.

### 3D Cultures in ECM and Live/Dead Staining of Spheroids

For 3D models, cells (8 × 10^2^) were suspended in a mixture of 25 μL phenol red-free, growth factor-reduced Matrigel (10 mg/mL; BD Biosciences, Erembodegem, Belgium) and 75 μL of spheroid plating medium per well in 96-well plates. The spheroid plating medium for melanoma cells was prepared as previously described^56^ whereas the spheroid plating medium for prostate cancer cells was prepared according to Di Donato et al.^46^ Spheroids were generated and either left untreated or treated with the indicated compounds for 21 days as indicated in Table S2. Half of the culture medium was replaced every 2 days with fresh medium containing the corresponding concentration of each compound. After 21 days, spheroids were stained using the Calcein AM/Propidium Iodide (PI) Double Staining Kit (#E-CK-A354, Elabscience), following the manufacturer’s instructions.

Compound concentrations were selected using a stepwise optimization strategy. All compounds were initially evaluated at 5 μM and, when no reproducible biological effect was observed, the concentration was increased to 10 μM and, if necessary, to 20 μM, in order to identify the minimum effective concentration under long-term 3D culture conditions.

Images were acquired with a MICA microhub microscope (Leica Microsystems) equipped with a × 10 objective, using immunofluorescence settings. The mean fluorescence intensity of the red (propidium iodide, dead cells) and green (Calcein AM, live cells) signals was quantified using NIH ImageJ software.^56^ Spheroid size was determined with Leica LAS X software and expressed as percentage relative to the basal (untreated) spheroid size measured at day 21-, which was set as 100%.

### Protein Extraction and Western Blot Analysis

Cell lysates were obtained using ice-cold lysis buffer composed of 50 mM Tris-HCl (pH 7.4), 1 mM EDTA, 1% Triton X-100, 150 mM NaCl, 5 mM MgCl_2_, and 1 mM EGTA, supplemented with protease and phosphatase inhibitors including 1 mM PMSF, protease inhibitor cocktail (LAP), 1 mM sodium orthovanadate (Na_3_VO_4_), and aprotinin (100 μg/mL). Protein concentrations were determined using the Bio-Rad Protein Assay (Bio-Rad Laboratories, Hercules, CA, USA).

Protein (20–40 μg) were resolved on 10–15% SDS-PAGE and subsequently transferred onto nitrocellulose membranes (Amersham Protran Premium 0.45 μm, GE Healthcare). Membranes were blocked with 5% BSA and incubated overnight at 4 °C with primary antibodies (listed below), followed by incubation with HRP-conjugated secondary antibodies. Protein bands were visualized using enhanced chemiluminescence (ECL; GE Healthcare, Chicago, IL, USA).

Antibodies used for Western blot: Mouse monoclonal: anti-α-tubulin (#E-AB-20036, Elabsciences) an anti-GAPDH (#E-AB-20079, Elabsciences); Rabbit polyclonal: anti-TRPM8 (#NBP1-97311, Novus Biologicals).

### siRNA-Mediated Gene Silencing, Transfection, and PI Staining

Small interfering RNA (siRNA) targeting human TRPM8 (sc-95009) and a nontargeting control siRNA (sc-44236) were purchased from Santa Cruz Biotechnology (Dallas, TX, USA). Melanoma (MEL-CAL) cells were seeded in 6-well plates at ∼60% confluence and transiently transfected with 150 pmol of either control or TRPM8-targeting siRNA using Lipofectamine 2000 (Thermo Fisher Scientific, Invitrogen), according to the manufacturer’s instructions. After 6 h, the medium was replaced, and cells were further incubated for 24 h. Following transfection, cells were either left untreated or exposed to the selected compounds as indicated in the corresponding figure. After 12 h of treatment, cells were processed for either protein analysis or cell death assessment.

For Western blot analysis, total protein extracts were collected from control wells to verify TRPM8 knockdown efficiency. Approximately 15 μg of total protein per sample were loaded, corresponding to lysates obtained from a single well of a 6-well plate. Notably, the protein amounts used in Figures S65–S68 were approximately doubled, as they were derived from 100 mm culture dishes.

In parallel, cell death was evaluated by propidium iodide (PI) staining (E-CK-A165, Elabscience). PI-positive (dead) cells were visualized by fluorescence microscopy using a MICA Leica imaging system (Leica Microsystems, Wetzlar, Germany). Phase-contrast images were acquired to determine total cell number. Images were captured and analyzed using LAS X software (Leica Microsystems).

Cell death was quantified as the percentage of PI-positive cells relative to the total cell number.

### Statistical Analysis

Unless otherwise indicated, all experiments were carried out in at least three independent replicates. Data are presented as mean ± standard deviation (SD). Statistical analysis was evaluated using one-way or two-way ANOVA followed by unpaired two-tailed Student’s *t* tests. For 3D spheroid experiments, statistical significance was determined using one-way ANOVA followed by Holm–Šídák’s multiple comparisons test. Data analysis was performed with GraphPad Prism software (version 8.0.2 or 9; GraphPad Software, San Diego, CA, USA). Differences were considered statistically significant at P-values <0.05.

### Computational Section

#### Molecular Docking

The structure of TRPM8 cocrystallized with the known inhibitor TC-I 2014 (PDB: 6O72)^35^ was selected and used for molecular docking investigations. The protein structure was prepared using Protein Preparation Workflow included in the Schrödinger Suite (v2025-2).^57^ During this step, hydrogen atoms and cap termini were added, and bond orders properly assigned. The coordinates of the cocrystallized ligand were employed to define the receptor grid used in the subsequent molecular docking calculations. The grid was built considering innerbox dimensions of 10 Å and outerbox dimensions of 27 Å (with center coordinates in Å: X = 148.52; Y = 149.40; Z = 199.12). After the grid generation step, the ligand was removed from the binding site. A redocking using the cocrystallized compound TC-I 2014 in TRPM8 protein structure (PDB code: 6O72) was performed to evaluate the performance of the docking software in resembling the experimentally determined binding mode (root-mean-square deviation (RMSD) = 0.94 Å computed between the two geometries, see Figure S80, Supporting Information).

All compounds under investigation were drawn using 2D sketcher of Maestro^58^ (v14.4) and then prepared through the LigPrep module^59^ (v74133, Schrödinger Suite). For each compound, all possible protonation states and tautomeric forms at pH 7.4 ± 1.0 were generated. The resulting geometries were then energy-minimized using the OPLS 2005 force field and molecular docking calculations were carried out with the Glide software^60^ (v10.7) at Extra Precision (XP) mode. A total of 10,000 poses were initially retained, with a ring sampling energy window of 5.0 kcal/mol (generating a maximum of 20 output poses).

#### Molecular Dynamics

Molecular dynamics (MD) simulations were performed using Desmond software,^61^ starting from the selected docking poses of compounds **7**, **29** and **34** in complex with the protein target. Each ligand-protein complex (chain A of TRPM8) was prepared using the System Setup panel, employing the TIP3P water model for solvatation and the OPLS 2005 force field. A cubic simulation box with a 10 Å solvent buffer was generated around the complex, and sodium (Na^+^) and chloride (Cl^–^) ions were introduced. The system was then subjected to a minimization step (Minimization panel of Desmond) for 1000 ps. Following this step, a stepwise relaxation protocol was applied, consisting of the following stages: a) NVT ensemble at 10 K for 100 ps, with positional restraints on all non-hydrogen solute atoms; b) NVT ensemble for 120 ps using the Berendsen thermostat at 10 K (12 ps); c) NPT ensemble for 12 ps at 10 K, employing both Berendsen thermostat and barostat; d) NPT ensemble for 12 ps at 300 K and 1 atm, under the same thermostat/barostat conditions; e) NPT ensemble for 24 ps at 300 K and 1 atm. After the equilibration steps, MD simulations of 1 μs were carried out in NPT, with a recording interval of 1000 ps and setting an NPT ensemble class (1.01 bar), with 2.0 fs as integration time step. RMSD values for both the protein and ligands were calculated by superimposing each trajectory frame, extracted every 2000 ps, onto the initial structure (frame 0), considering only the backbone atoms for the protein and all heavy atoms for the ligand.

### Patch-Clamp Experiments

#### Cell Culture Conditions

HEK-293/TRPM8 exon1 K3 cells were cultured in Minimum Essential Medium w/Earle’s Salts, w/o *L*-Glutamine (Euroclone cat. ECB2071L-500 mL) supplemented with 5 mL of 200 mM Ultraglutamine-1 in 0.85% NaCl Solution (Biowhittaker, cat. BE17-605E/U1), 5 mL of 100X Penicillin/Streptomycin (Biowhittaker, cat. DE17-602E), 0.2 mL of 10 mg/mL Puromycin (InvivoGen cat. ant-pr-5; final concentration 0.4 μg/mL) and with 50 mL of Fetal Bovine Serum (Sigma, cat. F7524).

#### Experimental Protocol

HEK-293/TRPM8 exon 1 cells were seeded 72 h before experiment, at a concentration of 5 million cells onto a T225 flask. Just before the experiments cells were washed twice with D-PBS w/o Ca^2+^/Mg^2+^ (Euroclone cat. ECB4004L) and detached from the flask with Detachin Cell Detachment Solution (Genlantis cat. T100106). Cells were then resuspended in the suspension solution: 25 mL EX-CELL ACF CHO medium (Sigma cat. C5467); 0.625 mL HEPES (BioWhittaker cat. BE17-737E); 0.25 mL of 100x Penicillin/Streptomycin (BioWhittaker cat. DE17-602E) and placed on the QPatch 48X.

#### Compound Preparation and Storage

REFERENCE AGONIST: Menthol stock solution (300 mM, 100% DMSO) was prepared the day of the experiment from the powder; the final dilution was performed in the extracellular solution to obtain a working concentration of 300 μM (1:1000, 0.1% final DMSO concentration).

COMPOUNDS: stock solutions (20 mM; 100% DMSO) were prepared the day of the experiment from the powder and the working dilutions were performed just before the experiments in the extracellular solution containing 300 μM menthol. The highest concentration tested was 10 μM, with 3 serial dilutions (1:10); therefore, the tested concentrations were: 0.01, 0.1, 1, 10 μM.

DMSO was balanced to keep it constant throughout all the solutions in the same experiment (0.2% final DMSO concentration).

#### Patch Clamp Analysis – QPatch 48X

Standard whole-cell voltage clamp experiments are performed at room temperature using the singlehole technology. For the voltage clamp experiments on human TRPM8, data are sampled at 10 kHz. After establishment of the seal and the passage in the whole cell configuration, the cells are challenged by a voltage ramp (20 ms step at −60 mV; 100 ms ramp −60/+100 mV; 20 ms step at +100 mV; return to −60 mV) every 4 s.

The potential antagonistic effect on human TRPM8 current of the compounds was evaluated after application of the agonist (menthol, 300 μM) alone and in the presence of the compounds under investigation at increasing concentrations.

Output: outward current evoked by the voltage ramp, measured in the step at +100 mV.

The intracellular solution contained (mM) 135 CsCl, 10 BAPTA, 10 HEPES, 4 Na_2_ATP (pH 7.2 with CsOH). The extracellular solution contained (mM) 145 NaCl, 4 KCl, 1 MgCl_2_, 2 BaCl_2_, 10 HEPES, 10 Glucose (pH 7.4 with NaOH).

#### Data Analysis

For data collection, the Sophion Assay Software was used, and the analysis was performed off-line using Excel and GraphPad Prism (v9.5.0). When possible, i.e., when the % of inhibition with the highest concentration tested was more than 50%, the dose–response curves data were fitted with the following equation:

$$Y=100/(1+10^{\left((\mathrm{Log}{\mathrm{IC}}_{50}-X)\times \mathrm{HillSlope}\right)})$$

X: log of concentration

Y: normalized response, 100% down to 0%, decreasing as X increases.

Log IC_50_: same log units as X

HillSlope: slope factor or hill slope, unitless

All data were expressed as mean ± SE.

### TRPA1 and TRPV1 Selectivity Assays

Functional selectivity over other TRP channels was evaluated using the same Ca^2+^ microfluorimetric protocol described for TRPM8. HEK293 cells stably expressing human TRPV1 and IRM90 cells endogenously expressing TRPA1 were loaded with a Ca^2+^-sensitive fluorescent dye under identical conditions, and changes in intracellular Ca^2+^ were recorded at 37 °C using a multiwell fluorescence reader. TRPV1 and TRPA1 were activated with capsaicin (10 μM) and allyl isothiocyanate (AITC, 10 μM), respectively, and ruthenium red (10 μM) was used as a reference antagonist for both channels. Test compounds (**7**, **29**, and **34**) were applied at concentrations ranging from 0.01 to 100 μM, following the same preincubation and recording protocol used for TRPM8. Responses were expressed as percentage of the agonist-induced signal in the absence of antagonist. Across this concentration range, none of the compounds produced detectable agonist effects, nor did they significantly inhibit capsaicin- or AITC-evoked Ca^2+^ responses, indicating an absence of measurable modulation of TRPV1 and TRPA1 under conditions that fully block TRPM8.

### Metabolic Stability Assay in Human Liver Microsomes (HLMs)

HLMs (Thermo Fisher Scientific, Bremen, Germany) were used for the microsomal stability assay. Reactions were initiated by adding NADPH and incubated at 37 °C for 20, 40, and 60 min in a Thermomixer (Eppendorf, Hamburg, Germany). The reaction was quenched with ice-cold methanol containing the IS, followed by centrifugation at 14,000 rpm for 7 min at 4 °C (Eppendorf, Hamburg, Germany). The supernatants were then analyzed by LC-MS. Further experimental details are provided in Figure S69.

Analyses were performed using a Waters ACQUITY UPLC I-Class system coupled online to a Waters Xevo TQ-XS triple quadrupole mass spectrometer (Waters Technologies Corp., Milford, MA, USA) equipped with an electrospray ionization (ESI) source operating in positive ion mode. The chromatographic separation was performed on a Kinetex 2.6 μm EVO C8 100 Å column (50 × 3 mm, Phenomenex, Bologna, Italy) employing as mobile phases: A) H_2_O and B) ACN, both acidified with 0.1% *v/v* formic acid with the following gradient: 0.00–3.00 min, 5–95% B; 3.01–4.00 min, isocratic at 95% B; followed by a 2.5 min re-equilibration step. The flow rate and column temperature were set at 0.7 mL·min^–1^ and 40 °C, respectively.

Analyte levels were determined using multiple reaction monitoring (MRM) in the positive ion mode. MRM transitions and parameters are listed in Table S3.

Calibration curves were constructed using standard solutions (0.005–1.5 μM) prepared by serial dilution of a DMSO stock in methanol containing tolbutamide (1 μM) as the internal.^62^ Each of the six calibration levels was analyzed in triplicate. Linear regression of analyte-to-IS peak area ratios produced highly linear curves (R^2^ ≥ 0.99), described by the following equations: **7**: y = 0.2963x – 0.0104; **29**: y = 0.2049x – 0.0092; **34**: y = 1.9245x – 0.0182.

Results were expressed as in vitro half-life (*t*_1/2_), in vitro microsomal intrinsic clearance (CL_int,micr_), and in vitro-in vivo scaled intrinsic hepatic clearance (CL_int,scaled_).

In vitro half-life (*t*_1/2_, min) was calculated using (eq 1):

1 $$t_{1/2}=0.693/\kappa$$

where κ is the slope found in the linear fit of the natural logarithm of the fraction remaining of the parent compound vs incubation time.

Microsomal intrinsic clearance (CL_int,micr_, μL min^–1^ mg^–1^) was determined using (eq 2):

2 $$CL_{int,micr}=1000\times \left(0.693/t_{1/2}\right)/0.5$$

where the microsomal protein concentration in the incubation mixture was 0.5 mg mL^–1^, and 1000 is the unit-scaling factor for volume (μL).

In vitro-in vivo scaled intrinsic hepatic clearance (CL_int,scaled_, mL min^–1^ kg^–1^) values were then scaled to the whole liver in vivo equivalent values using (eq 3):

3 $$CL_{int,scaled}=CL_{int,micr}\times SF$$

where SF equals the physiological scaling factors: mg of microsomal protein/g of liver, and the liver/body weight ratio.^63^

### Metabolite Identification (MetID) Following Microsomal Incubation

MetID was performed on a Vanquish UHPLC coupled to an Orbitrap Exploris 120 mass spectrometer (Thermo Fisher Scientific, Bremen, Germany) equipped with a HESI II source operating in positive ion mode. Chromatographic separation was achieved on a Kinetex 2.6 μm EVO C18 (100 × 2.1 mm) column using a water/acetonitrile gradient (0.1% formic acid, 5–95% B over 10 min) at 0.4 mL·min^–1^ and 40 °C. Full-scan MS spectra were acquired at 60,000 resolution (*m*/*z* 100–1500), with data-dependent MS/MS at 15,000 resolution, 30% HCD energy, and standard ESI source parameters.

Data processing was performed in Compound Discoverer 3.3 (Thermo Fisher Scientific) using a customized MetID workflow for the detection and structural elucidation of both expected and novel metabolites, following the procedure reported by Lamberti et al.,^64^ with minor modifications.

*In silico* biotransformation predictions were generated using GLORYx (NERDD portal, https://nerdd.univie.ac.at/gloryx), and the resulting compound list (name, formula, exact mass, SMILES) was imported into the workflow.^65^ Processing steps included retention time alignment, isotope pattern confirmation, compound grouping, FISh scoring, fragment annotation, and gap filling.

A schematic representation of the MetID workflow is provided in Figure S69D.

### Glutathione Trapping Assay

The GSH trapping assay was performed under the same experimental conditions as the liver microsomal stability assay, except for the inclusion of NADPH and GSH solutions in a 1:1 volume *ratio*. After 60 min of incubation with HLMs at 37 °C under shaking at 450 rpm, the reaction was quenched by adding 100 μL of H_2_O followed by 600 μL of ice-cold acetonitrile:methanol (9:1, v/v) containing the IS. The samples were centrifuged at 14,000 rpm for 10 min at 4 °C, and the resulting supernatant was evaporated to approximately 200 μL under a gentle nitrogen stream at 35 °C using a Concentrator Plus device (Eppendorf, Hamburg, Germany). The residues were subsequently analyzed by LC-MS analysis.^66^ Positive control samples included diclofenac and acetaminophen.

### Plasma Stability Assay

Plasma stability was assessed by incubating compound **7** in human plasma at 37 °C. At 240 min, aliquots were quenched with ice-cold organic solvent containing IS. Samples were analyzed by LC-MS to quantify the remaining parent compound, and stability was expressed as the percentage of the initial concentration (t_0_). Procaine and procainamide were used as reference compounds for low and high plasma stability, respectively. All experiments were performed in triplicate.

### *In Silico* Analysis of TRPM8 Gene Expression

The differential steady-state mRNA expression of the TRPM8 gene across multiple cancer types was evaluated utilizing the Gene Expression Profiling Interactive Analysis version 1­(GEPIA) web-based platform (http://gepia.cancer-pku.cn/). GEPIA provides customizable analysis by integrating harmonized RNA sequencing (RNA-seq) data sets from The Cancer Genome Atlas (TCGA) and the Genotype-Tissue Expression (GTEx) projects, comprising thousands of tumor and normal tissue samples. The Box Plot module was employed to generate the expression profiles. Transcript abundance was normalized and is reported as log2­(TPM + 1) (Transcripts Per Million). Statistical significance between tumor and corresponding normal tissues was determined via a one-way analysis of variance (ANOVA). The significance thresholds for differential expression were strictly defined as a p-value <0.01 and an absolute log2 fold change (|log2FC|) > 1.

## Supplementary Material















































## Notes

Compound 10 in complex with TRPM8 (PDB)
Compound 12 in complex with TRPM8 (PDB)
Compound 13 in complex with TRPM8 (PDB)
Compound 23 in complex with TRPM8 (PDB)
Compound 24 in complex with TRPM8 (PDB)
Compound 25 in complex with TRPM8 (PDB)
Compound 26 in complex with TRPM8 (PDB)
Compound 27 in complex with TRPM8 (PDB)
Compound 28 in complex with TRPM8 (PDB)
Compound 29 in complex with TRPM8 (PDB)
Compound 3 in complex with TRPM8 (PDB)
Compound 30 in complex with TRPM8 (PDB)
Compound 31 in complex with TRPM8 (PDB)
Compound 32 in complex with TRPM8 (PDB)
Compound 34 in complex with TRPM8 (PDB)
Compound 35 in complex with TRPM8 (PDB)
Compound 5 in complex with TRPM8 (PDB)
Compound 6 in complex with TRPM8 (PDB)
Compound 7 in complex with TRPM8 (PDB)
Compound 8 in complex with TRPM8 (PDB)
Compound 9 in complex with TRPM8 (PDB)
Figures S1–S80: ^1^H, ^13^C spectra, and HPLC traces of synthesized compounds; Figure S64: Ca^2+^ imaging concentration–response curves for compounds **7**, **29**, and **34**; Figure S65: analysis of TRPM8 expression and effects of TRPM8 modulators on MEL-CAL cell viability; Figure S66: analysis of TRPM8 expression and effects of TRPM8 modulators on Malme-3M cell viability; Figure S67: analysis of TRPM8 expression and effects of TRPM8 modulators on LNCaP cell viability; Figure S68: analysis of TRPM8 expression and effects of TRPM8 modulators on PC3 cell viability; Figure S69: analysis of TRPM8 expression and effects of TRPM8 modulators on PNT-2 cell viability; Figure S70: analysis of TRPM8 expression and effects of TRPM8 modulators on NIH3T3 cell viability; Table S1: IC_50_values (μM) of compound 14 in melanoma, prostate cancer, and nontransformed cell lines; Table S2: concentrations of compounds used for spheroid treatments ; Figure S71: TRPA1 and TRPV1 selectivity assays of compounds **7**, **29**, and **34** at concentrations ranging from 0.005 to 50 μM; Table S3: MRM parameters for all analytes tested; Figure S72: liver microsomal stability assessment in human liver microsomes (HLMs); Figure S73: identification of metabolites following microsomal incubation of test compounds; Figure S74: MS^2^ spectra illustrating the proposed fragmentation pattern of metabolites derived from compound **7**; Figure S75: MS^2^ spectra of metabolites derived from compound **34** after incubation with HLMs in the presence of NADPH; Figure S76: MS/MS spectra of HLM-generated metabolites of compound **29**; Figure S77: NADPH-dependent and -independent metabolic and plasma stability of compound **7**; Figure S78: differential mRNA expression of TRPM8 antagonists on size and viability of Malme-3M spheroids; Table S3: docking scores related to the selected poses of the compounds investigated through molecular docking; Figure S79: superposition of the three most promising compounds emerged from the present study: **7**, **29**, and **34** (PDF)
String network (CSV)
