# Supplementary material for: New TRPM8 Amino Acid-Based Antagonists Induce Antiproliferative Effect on 2D and 3D Melanoma and Prostate Cancer Models
Source: J Med Chem. 2026 Aug 6;69(16):19644–70. doi: 10.1021/acs.jmedchem.6c00479 (PMC13528199; doi:10.1021/acs.jmedchem.6c00479)
Supplement: Supplementary file 1 [file jm6c00479_si_022.pdf]

SUPPORTING INFORMATION

New TRPM8 amino acid-based antagonists induce antiproliferative effect on 2D and 3D melanoma and prostate cancer models

Tania Ciaglia,[a,#] Marzia Di Donato,[b,#] Veronica Di Sarno,[a] Gerardina Smaldone,[a] Sofia Pellecchia,[a] Francesca Di Matteo,[a] Valeria Napolitano,[a] Eleonora Boccia,[a] Carmela Sorrentino,[b] Giacomo Pepe,[a] Manuela Giovanna Basilicata,[c] Carmine Lauretta,[b] Giovanna Aquino,[a,d] Pia Giovannelli,[b] Isabel M. Gomez-Monterrey,[e] Vincenzo Vestuto,[a] Antimo Migliaccio,[b] Pietro Campiglia,[a] Gianluigi Lauro,[a] Carmine Ostacolo,[a] Giuseppe Bifulco,[a] Gabriella Castoria,[b] Antonio Ferrer-Montiel,[f] Asia Fernández-Carvajal,[f] Simona Musella,[a,*] Alessia Bertamino,[a,*]

[a]Department of Pharmacy, University of Salerno, Via G. Paolo II 132, 84084, Fisciano, Salerno, Italy

[b]Department of Precision Medicine, School of Medicine- University of Campania 'L. Vanvitelli', Via L. De Crecchio 7, 80138, Naples, Italy

[c]Department of Advanced Medical and Surgical Sciences, University of Campania "Luigi Vanvitelli", P.zza L. Miraglia, 2, 80138, Naples, Italy

[d]AREA Science Park, Laboratorio di Multi-omica Area Sud (LAAS), 84081 Baronissi, Salerno, Italy

[e]Department of Pharmacy, School of Medicine and Surgery, University of Naples Federico II, Via D. Montesano 49, 80131, Naples, Italy

[f]Instituto de Investigación, Desarrollo e Innovación en Biotecnología Sanitaria de Elche (IDiBE), Universidad Miguel Hernández de Elche, Avenida de la Universidad, 03202 Elche, Spain

[#] These authors contributed equally to this work.

*Correspondence should be addressed to: Alessia Bertamino (abertamino@unisa.it) and Simona Musella (smusella@unisa.it)

Table of contents:

**Figures S1-S63.** ^{1}H, qDEPT spectra and HPLC traces of compounds **3**, **5-10**, **12**, **13**, **23-32**, **34** and **35**	**S3-S65**
**Figures S64.** Ca^{2+} imaging concentration–response curves for compounds **7, 29, 34**	**S66**
**Figure S65.** Analysis of TRPM8 expression and effects of TRPM8 modulators on MEL-CAL cell viability	**S67**
**Figure S66.** Analysis of TRPM8 expression and effects of TRPM8 modulators on Malme-3M cell viability	**S68**
**Figure S67.** Analysis of TRPM8 expression and effects of TRPM8 modulators on LNCaP cell viability	**S69**
**Figure S68.** Analysis of TRPM8 expression and effects of TRPM8 modulators on PC3 cell viability	**S70**
**Figure S69.** Analysis of TRPM8 expression and effects of TRPM8 modulators on PNT-2 cell viability	**S71**

**Figure S70.** Analysis of TRPM8 expression and effects of TRPM8 modulators on NIH3T3 cell viability	**S72**
**Table S1.** IC_{50} values (μM) of the previously reported TRPM8 antagonist (compound 14) in melanoma, prostate cancer, and non-transformed cell lines after 72 h of treatment.	**S73**
**Table S2.** Concentrations of Compounds Used for Spheroid Treatments	**S73**
**Figure S71.** TRPA1 and TRPV1 selectivity assays of compounds **7**, **29** and **34** at concentrations ranging from 0.005 to 50 μM	**S74**
**Table S2.** MRM parameters for all compounds tested	**S75**
**Figure S72.** Liver microsomal stability assessment in human liver microsomes (HLMs).	**S75**
**Figure S73.** Identification of metabolites following microsomal incubation of test compounds.	**S76-77**
**Figure S74.** MS^2 spectra illustrating the proposed fragmentation pattern of metabolites derived from compound **7**	**S78**
**Figure S75.** MS^2 spectra of metabolites derived from compound **34** after incubation with HLMs in the presence of NADPH	**S79**
**Figure S76.** MS/MS spectra of HLM-generated metabolites of compound **29**	**S80**
**Figure S77.** NADPH-dependent and -independent metabolic and plasma stability of compound **7**	**S81**
**Figure S78.** Differential mRNA expression profile of TRPM8 across different cancer type	**S82**
**Table** S3**.** Docking scores related to the selected poses of the compounds investigated through molecular docking.	**S83**
**Figure S79.** Superposition of the three most promising compounds emerged from the present study: **7**, **29** and **34**	**S83**
**Figure S80.** Superposition of the co-crystallized binding pose of TC-I 2014 obtained by redocking in TRPM8 binding site	**S84**

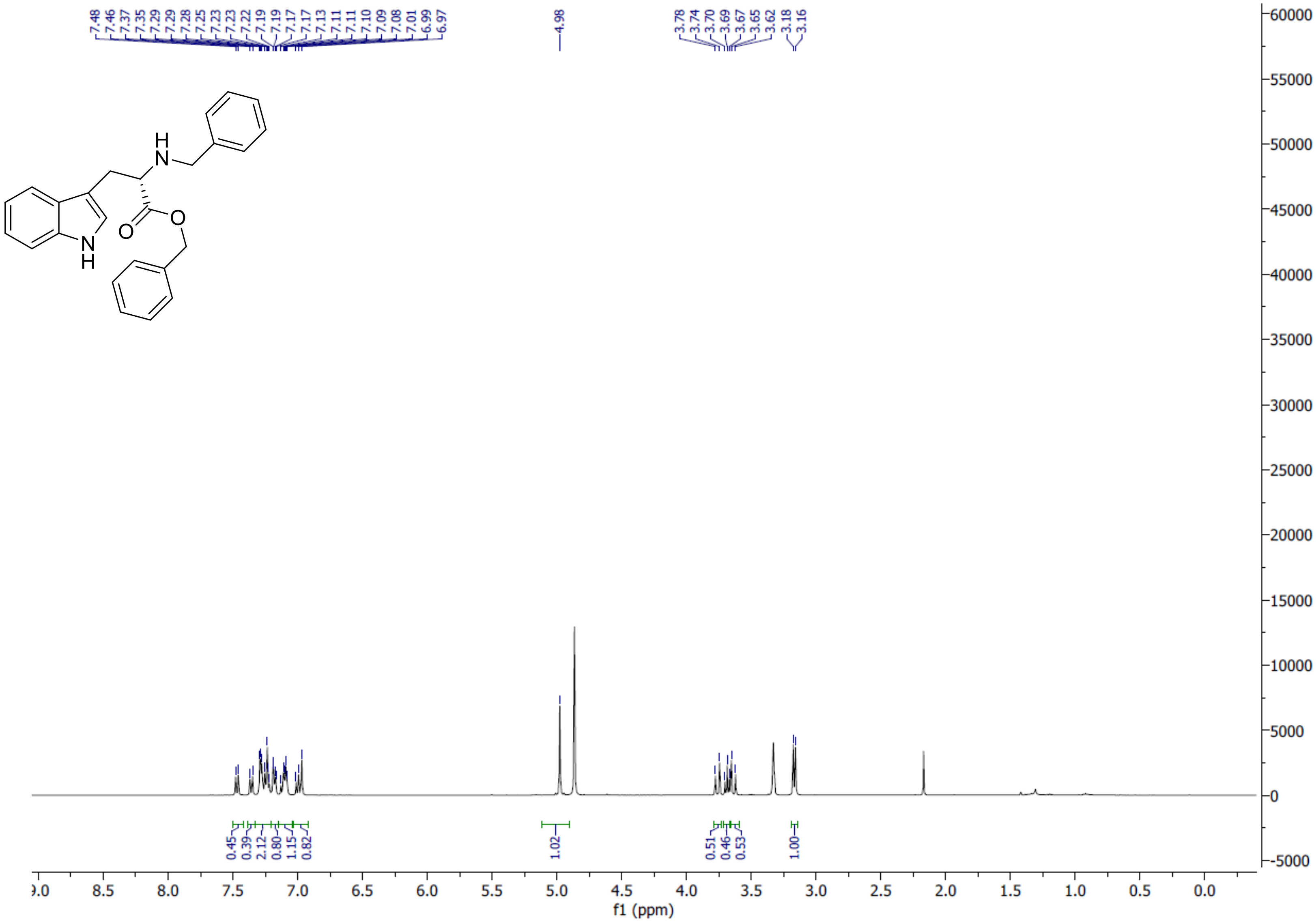

Figure S1: ^{1}H NMR spectrum of compound **3**

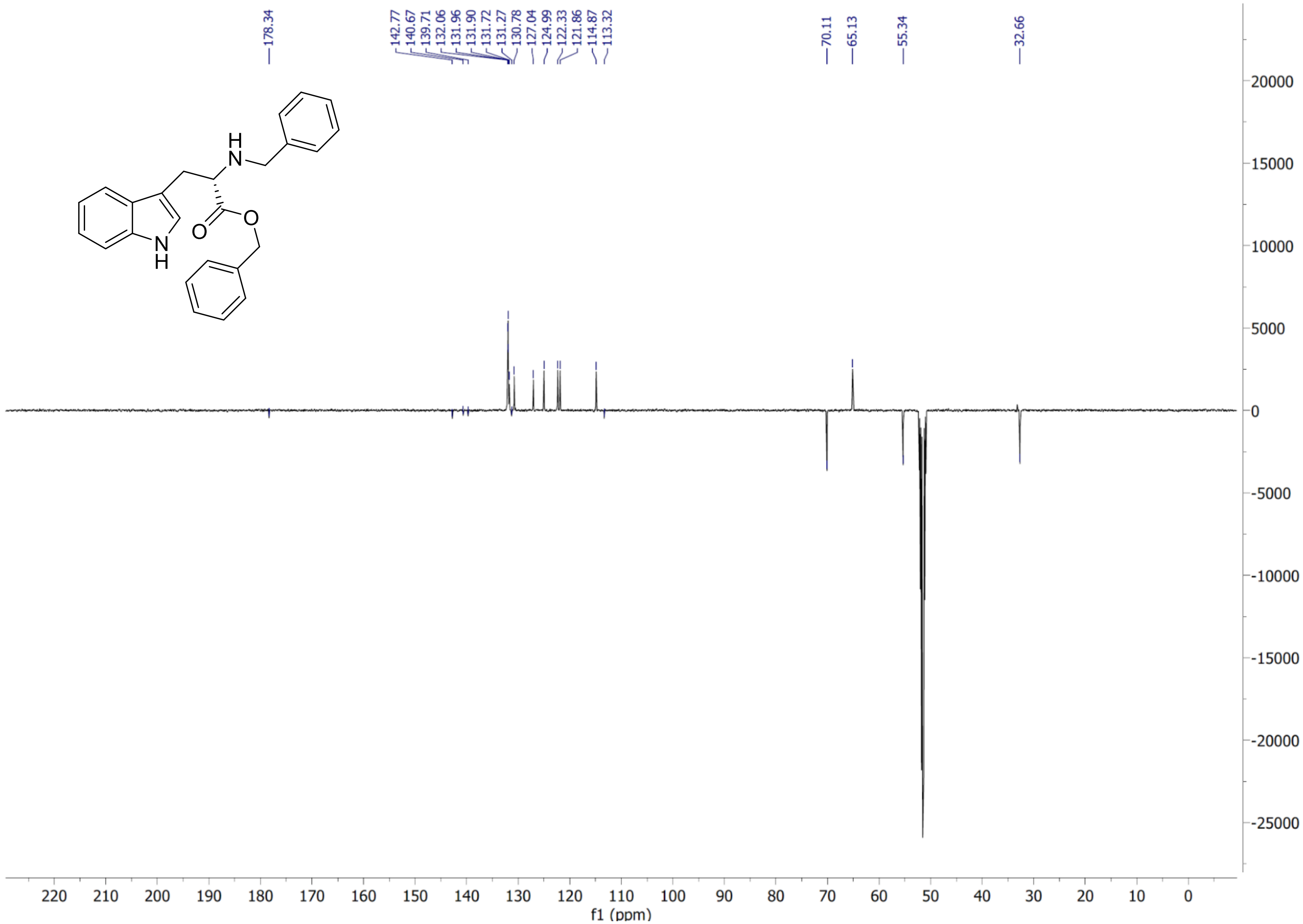

Figure S2: qDEPT spectrum of compound **3**

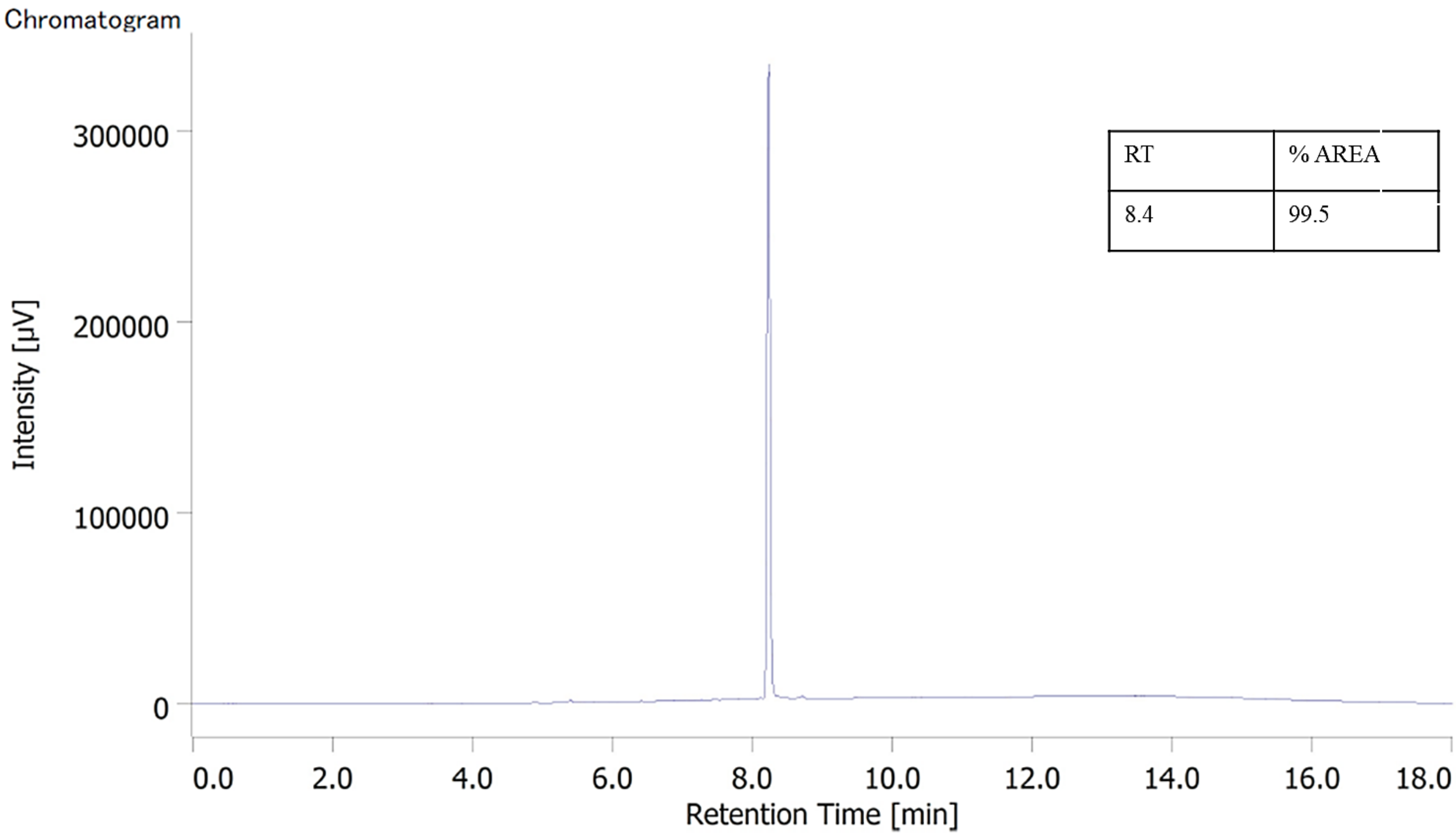

Figure S3: HPLC trace of compound **3**

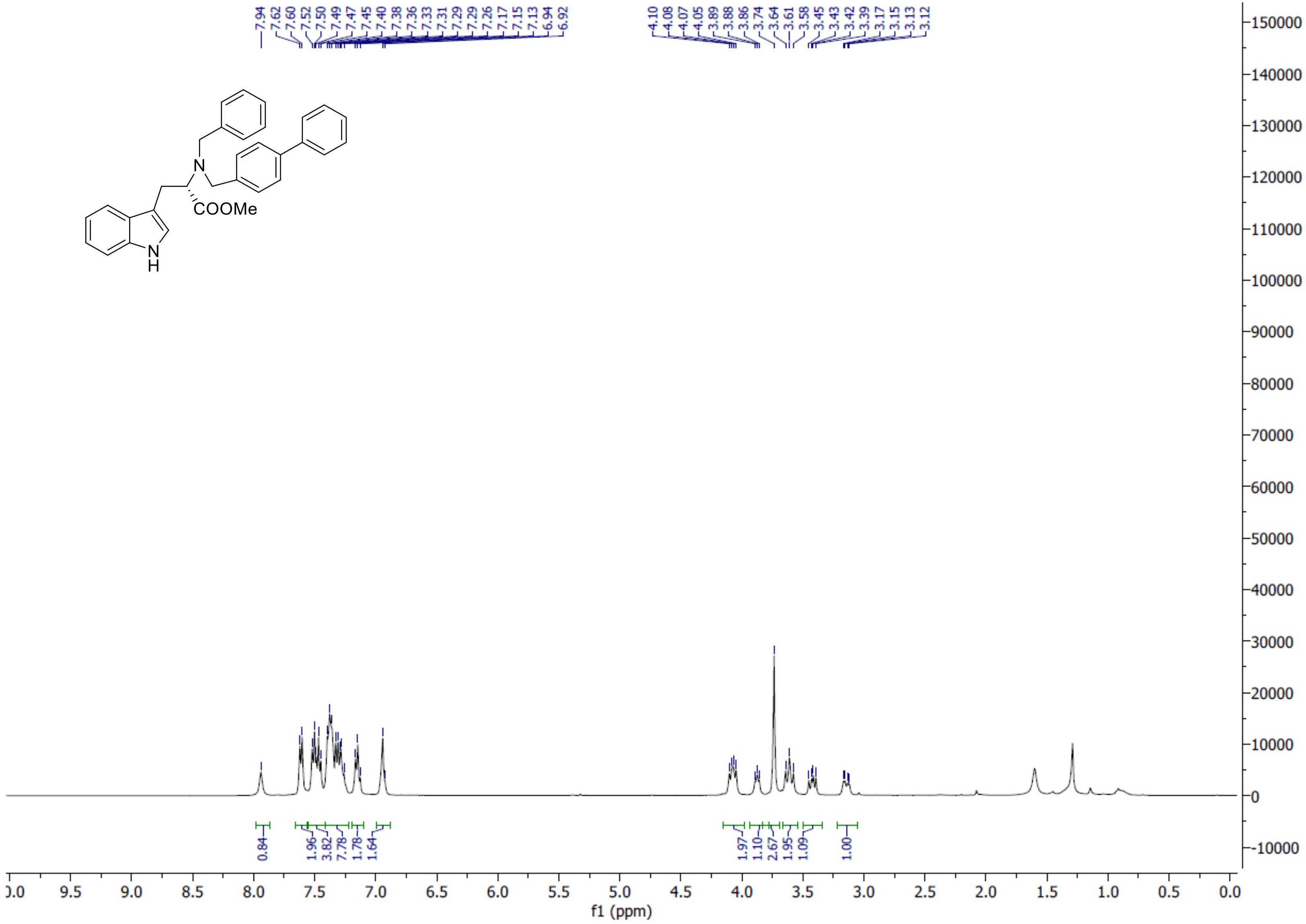

Figure S4: ^{1}H NMR spectrum of compound **5**

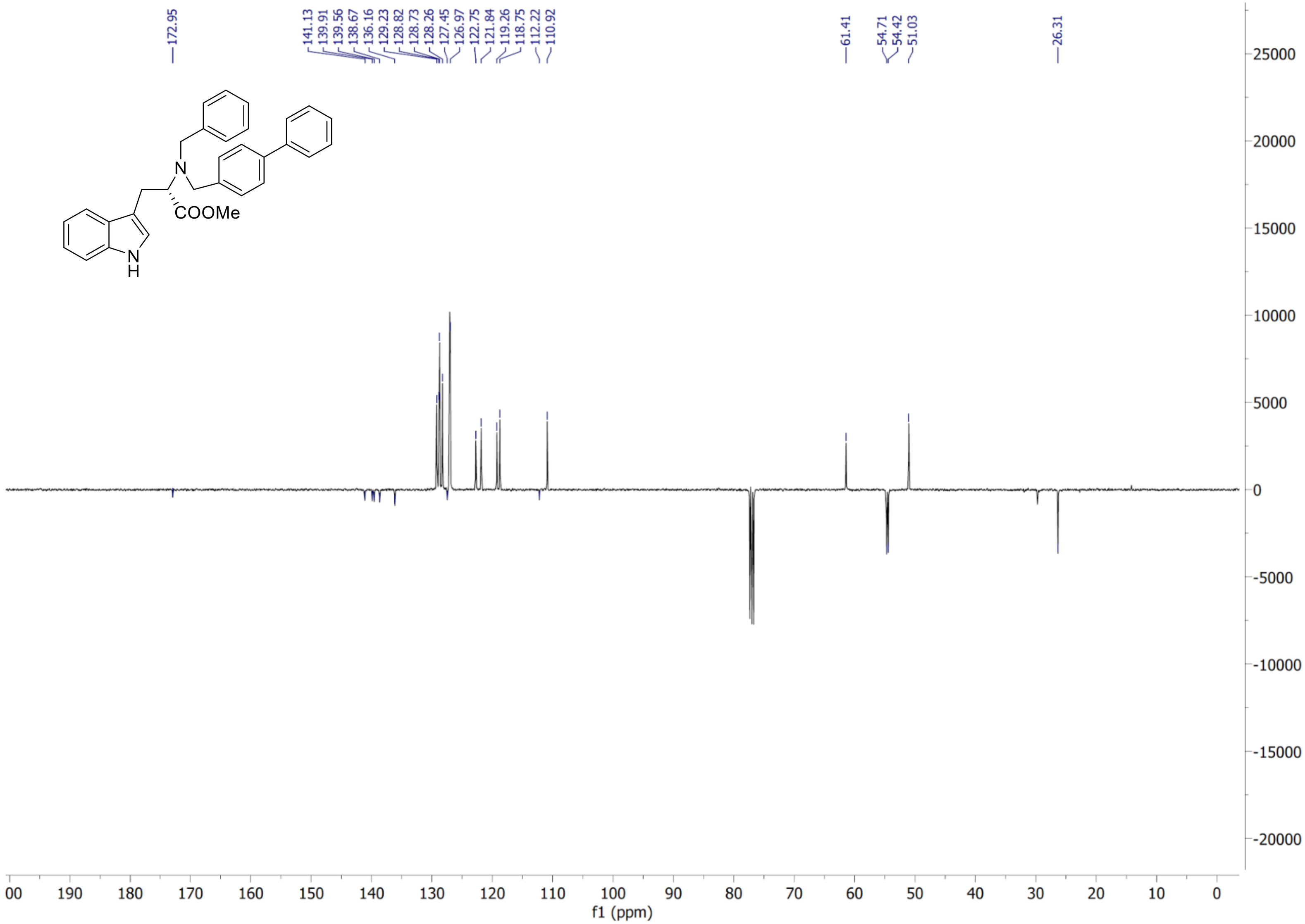

Figure S5: qDEPT spectrum of compound **5**

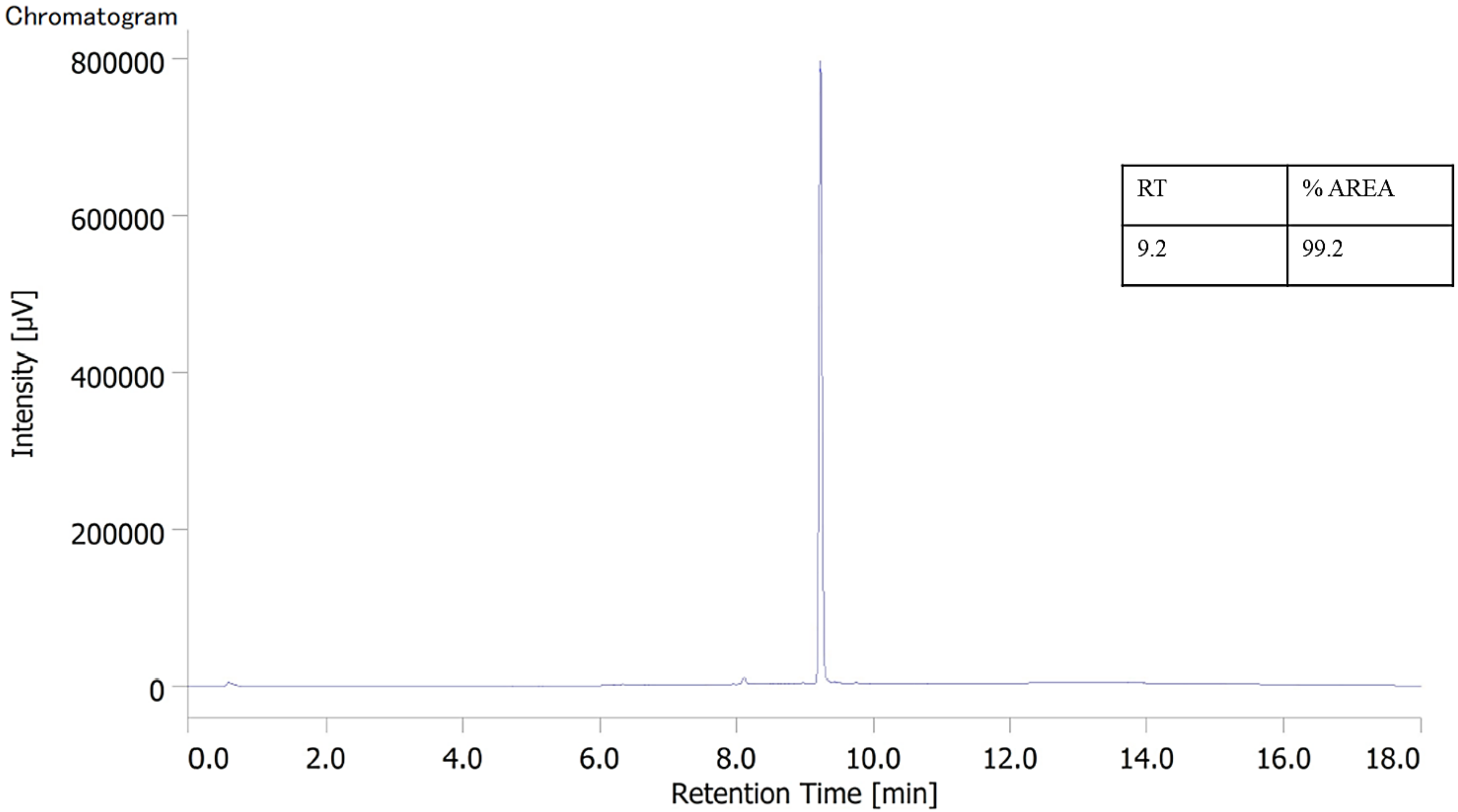

Figure S6: HPLC trace of compound **5**

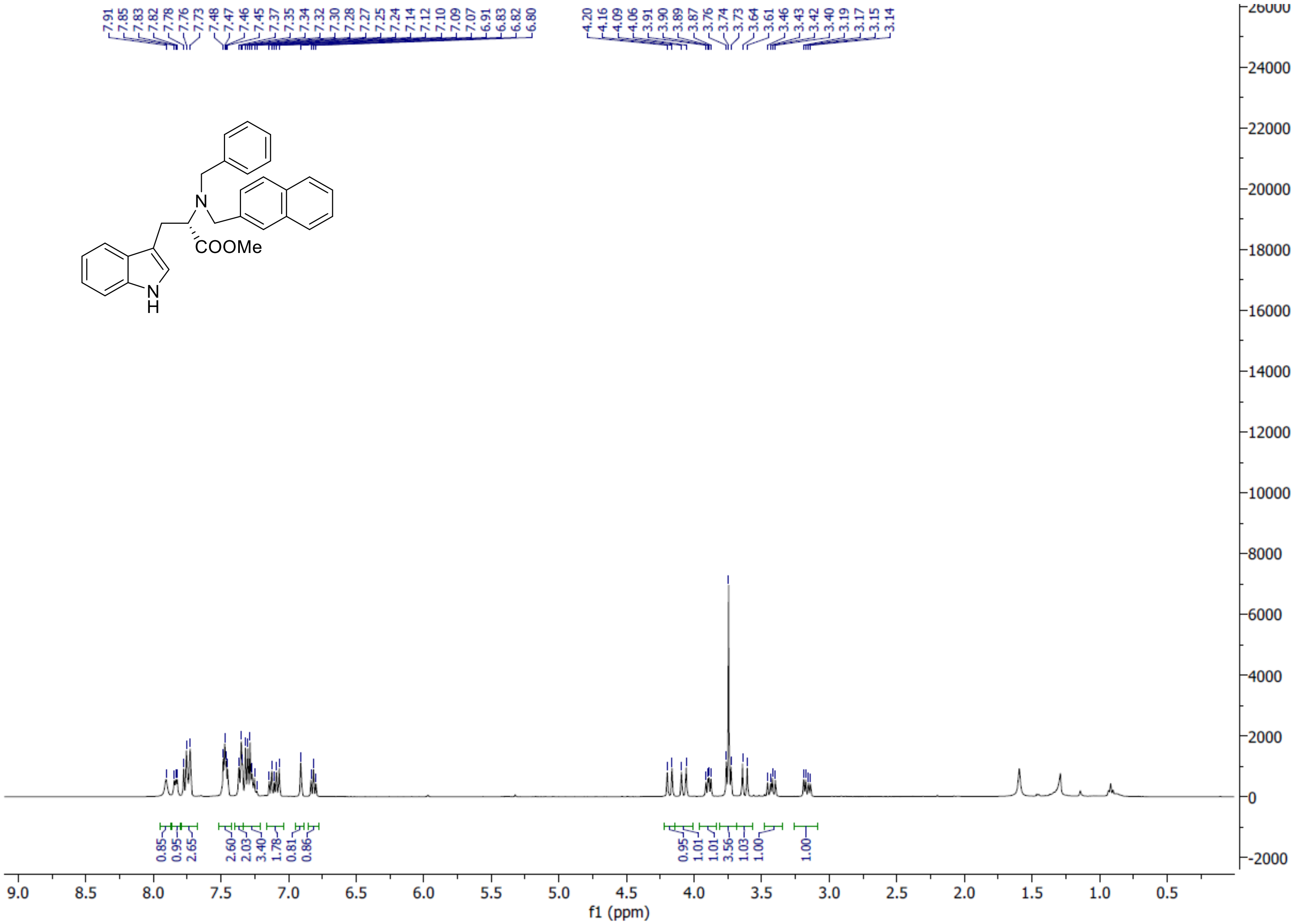

Figure S7: ^{1}H NMR spectrum of compound **6**

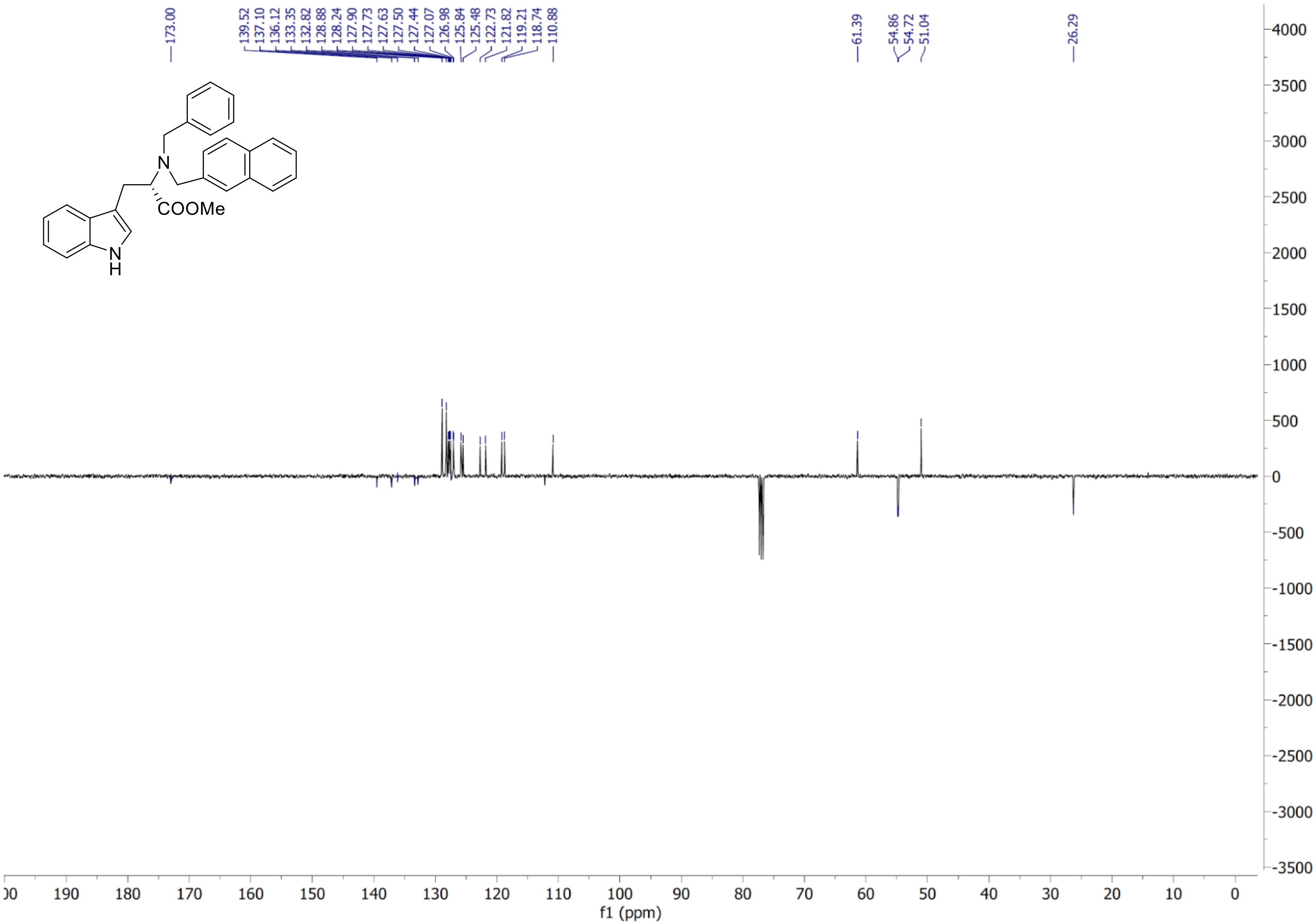

Figure S8: qDEPT spectrum of compound **6**

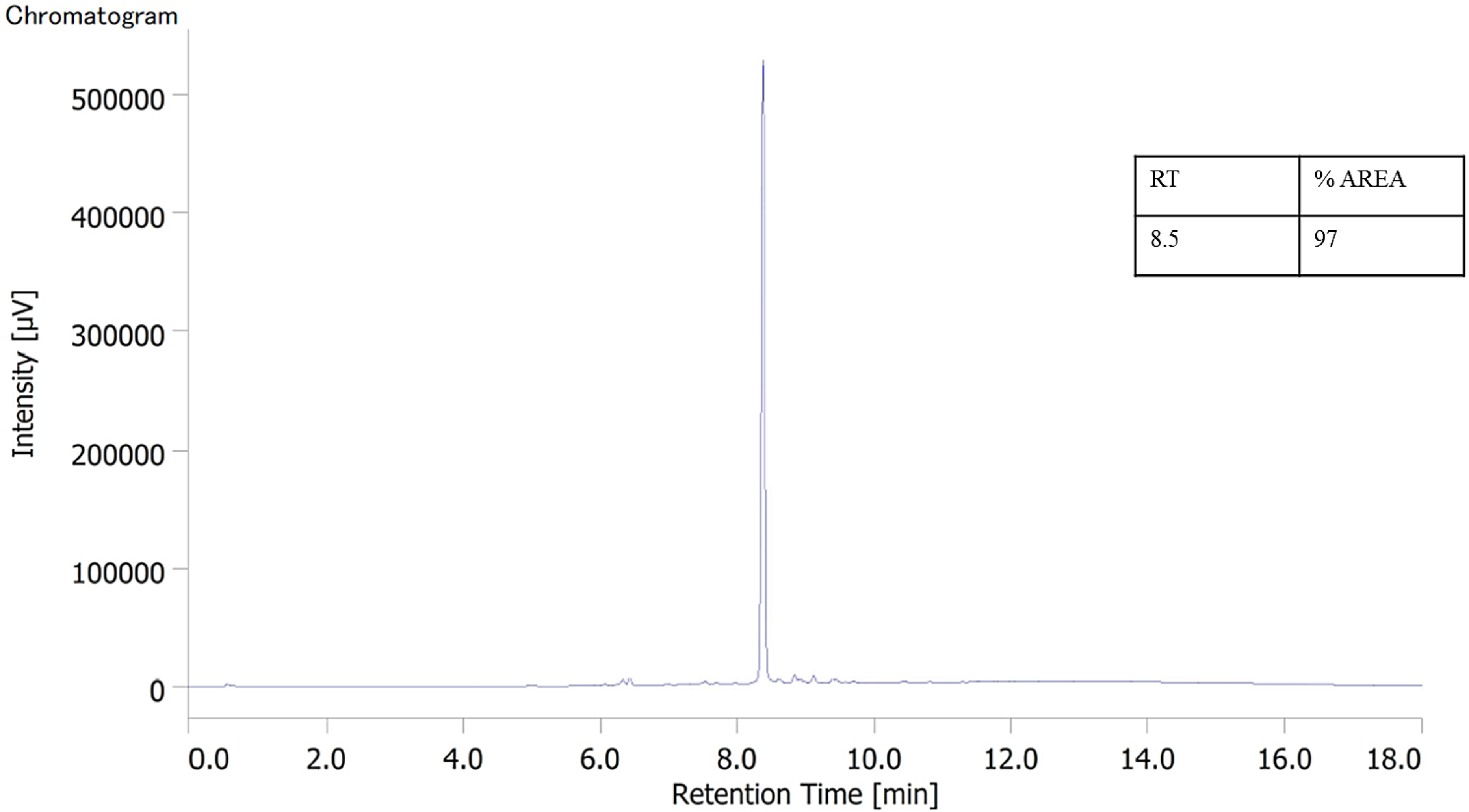

RT	% AREA
8.5	97

Figure S9: HPLC trace of compound **6**

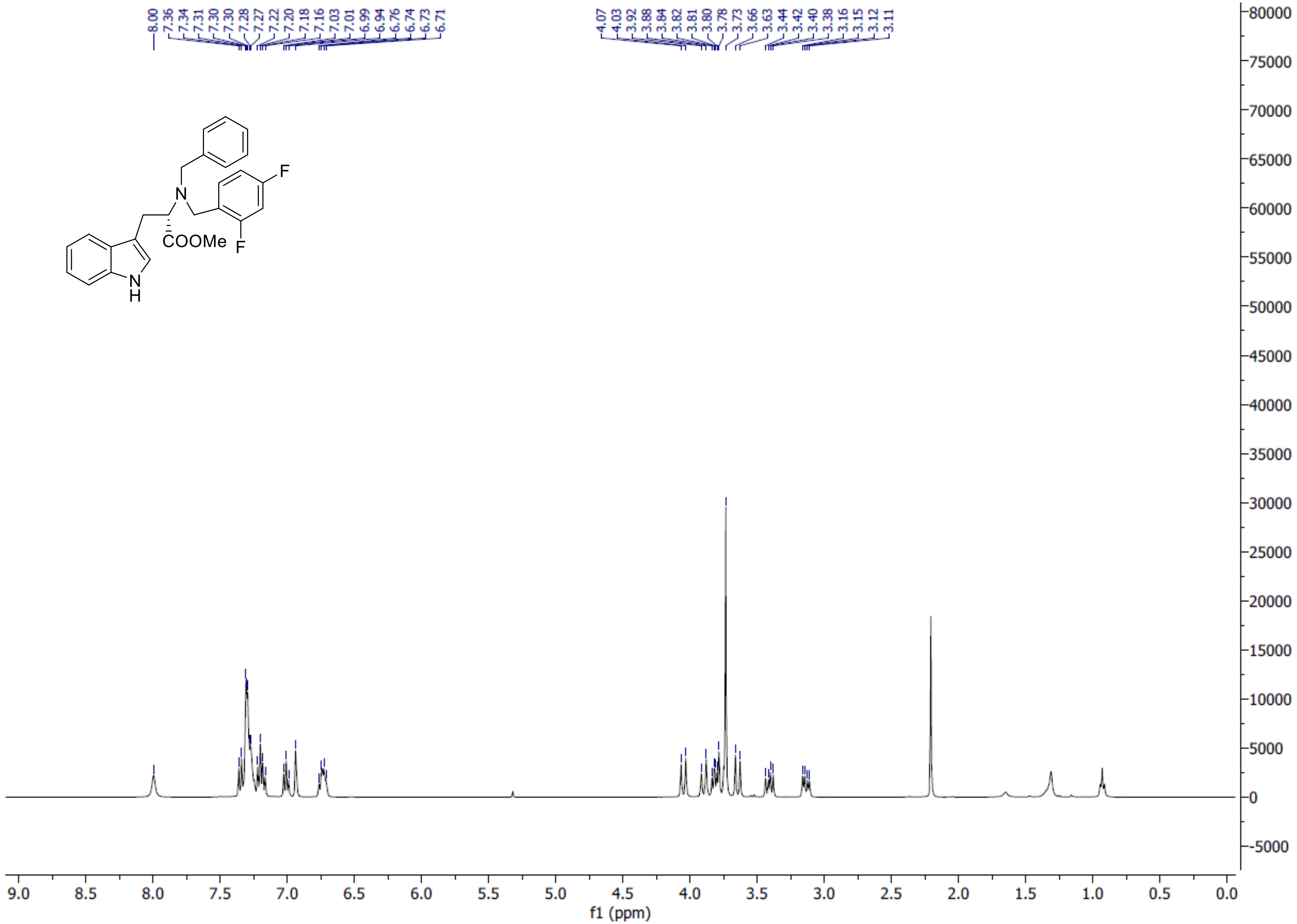

Figure S10: ¹H NMR spectrum of compound **7**

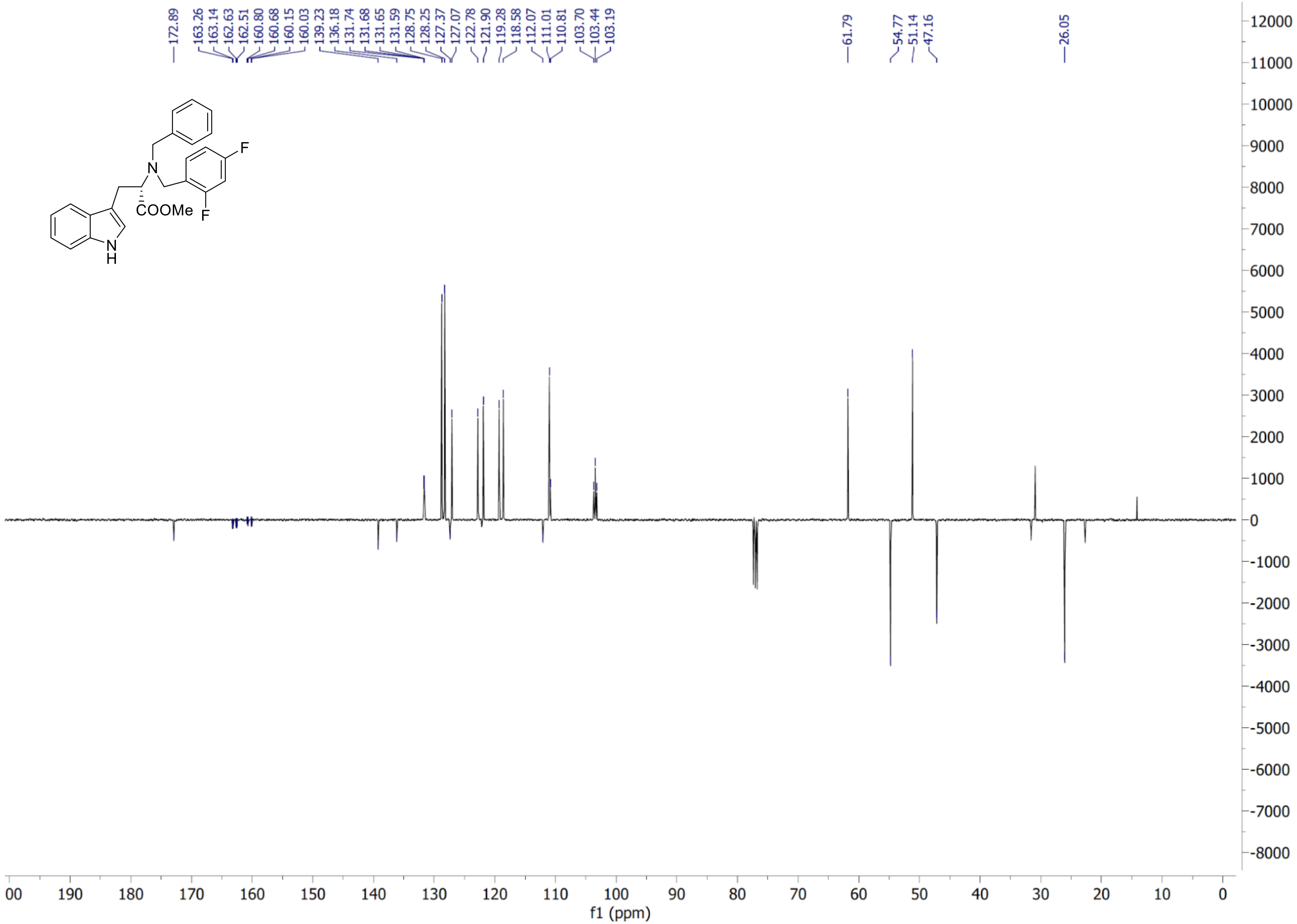

Figure S11: qDEPT spectrum of compound **7**

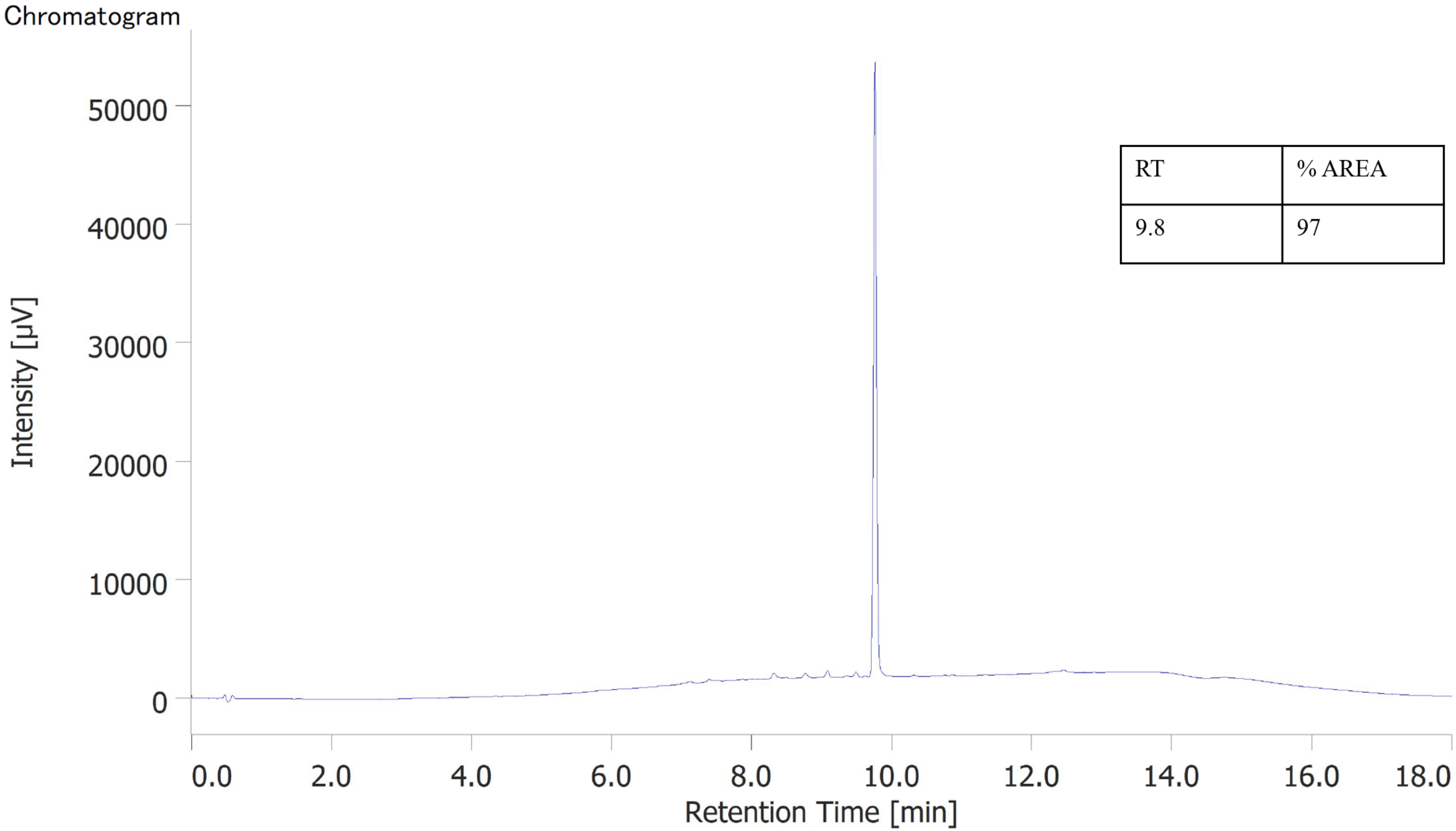

Figure S12: HPLC trace of compound **7**

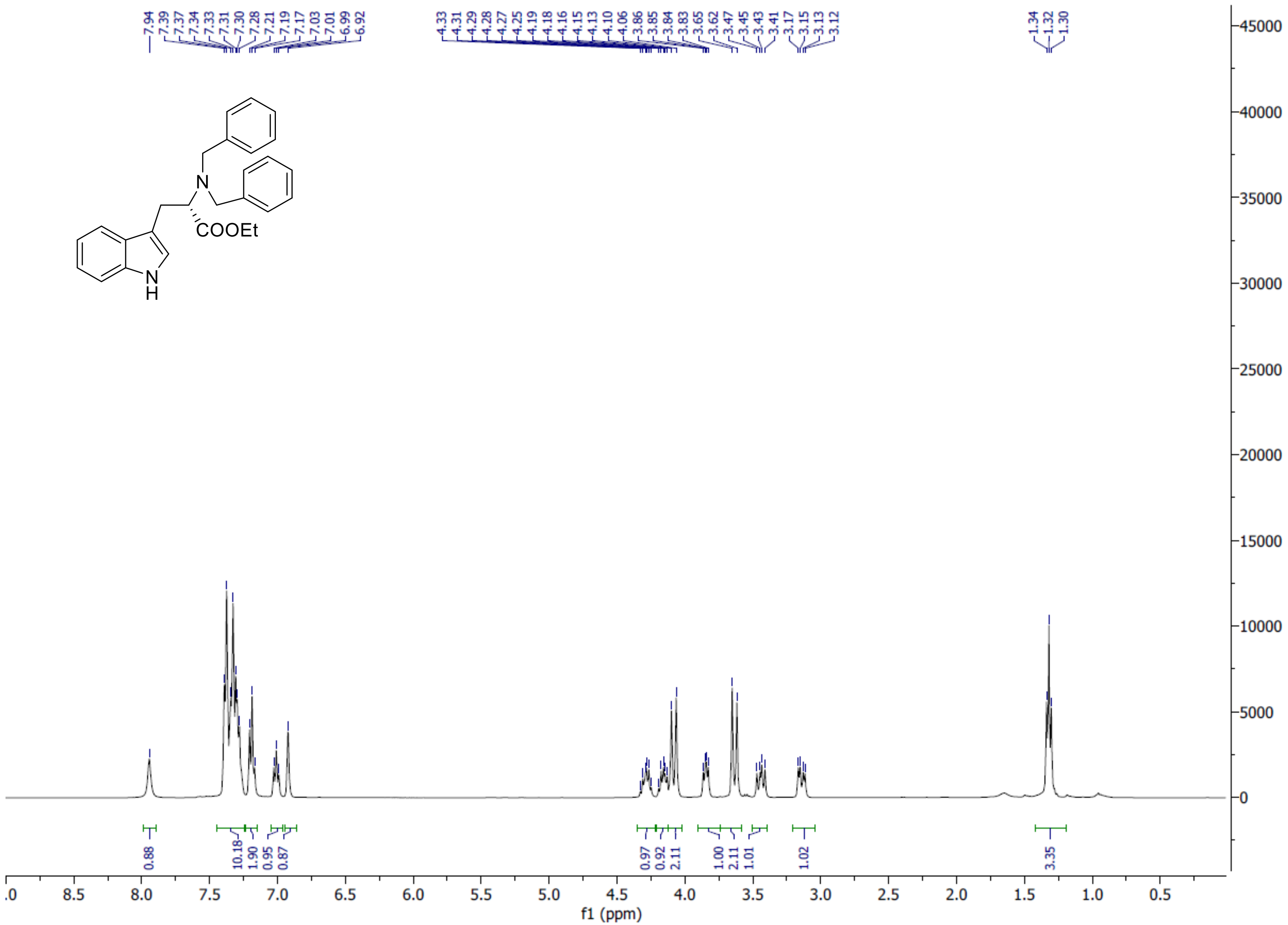

Figure S13: ^{1}H NMR spectrum of compound **8**

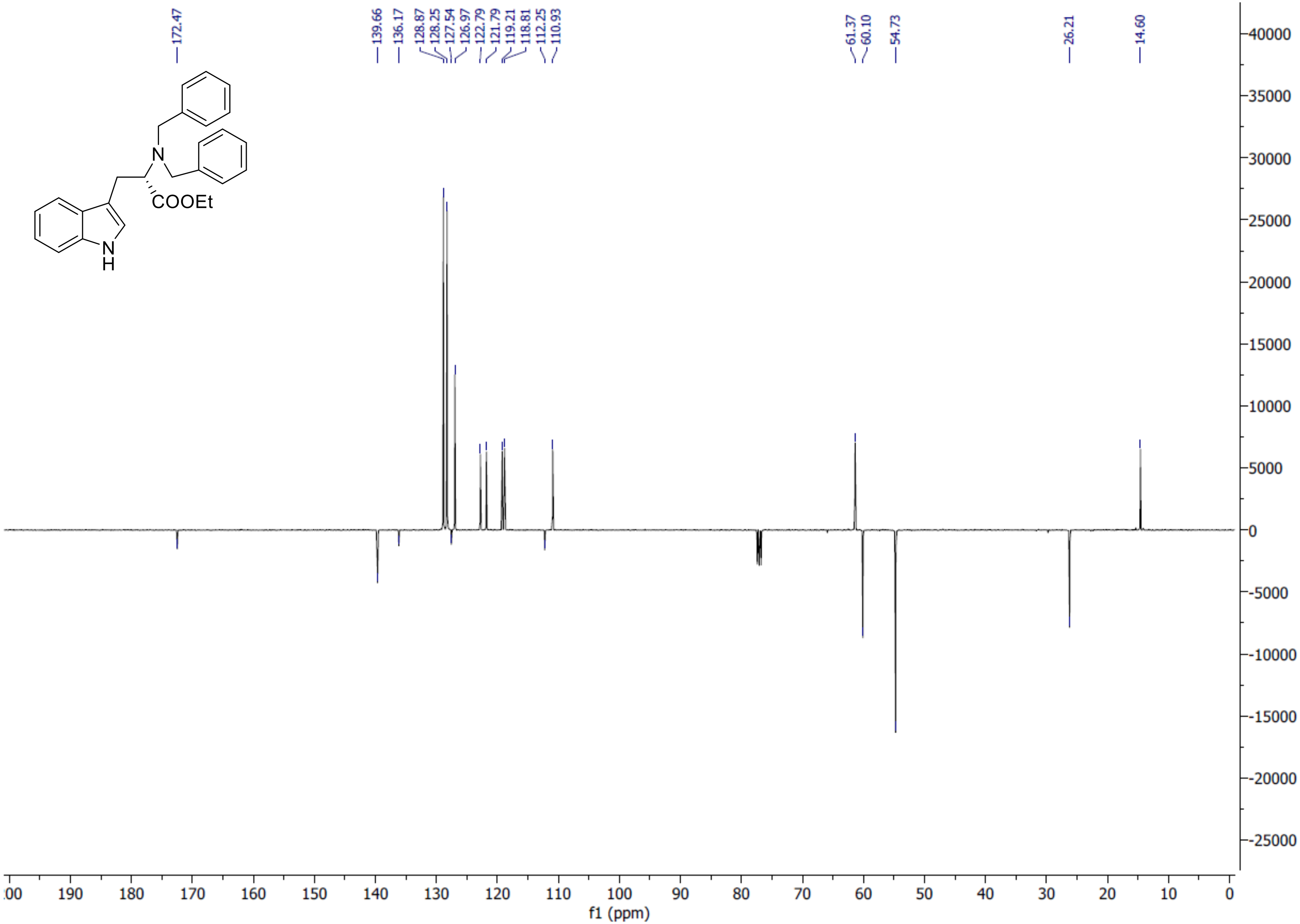

Figure S14: qDEPT spectrum of compound **8**

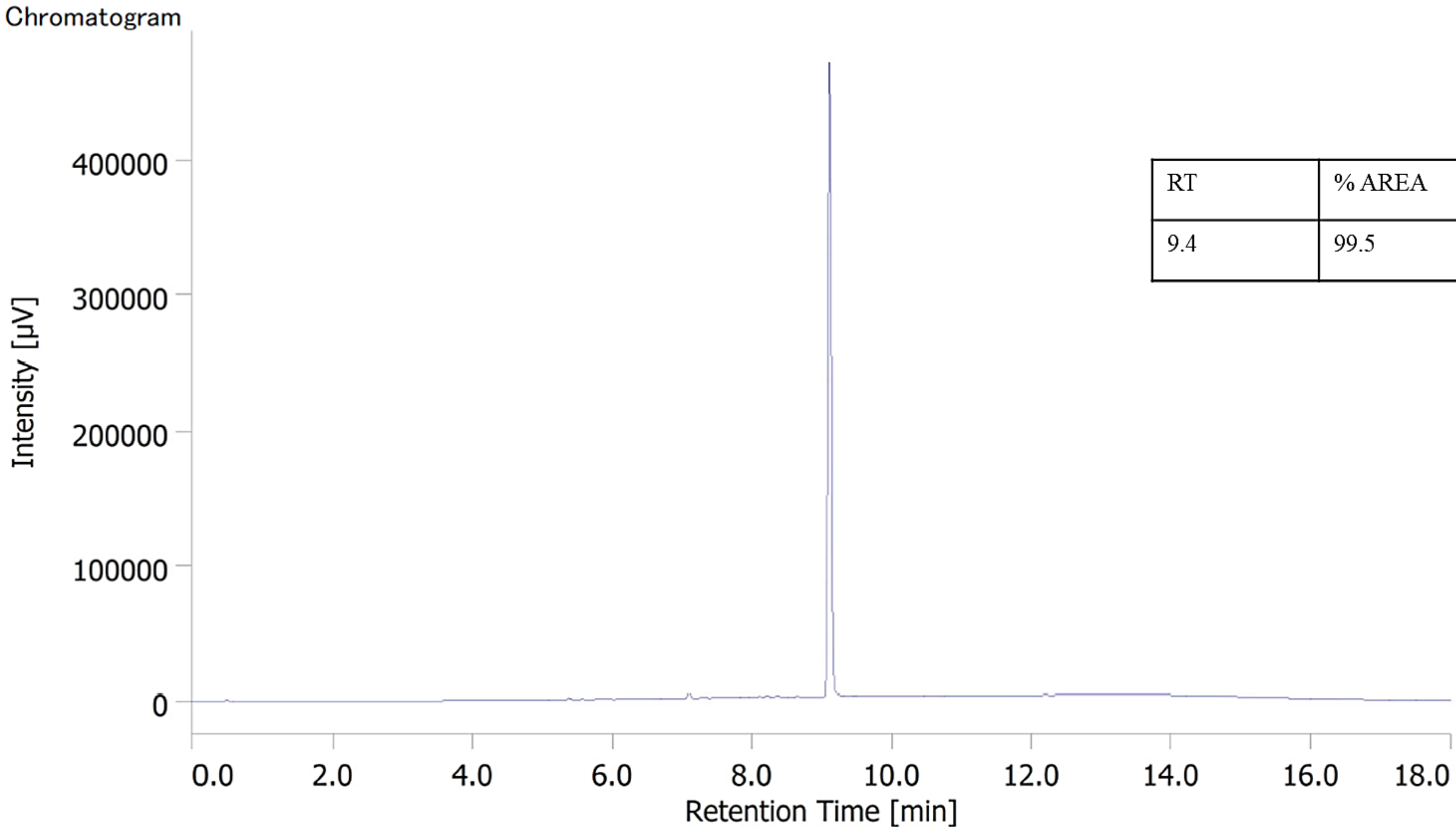

RT	% AREA
9.4	99.5

Figure S15: HPLC trace of compound **8**

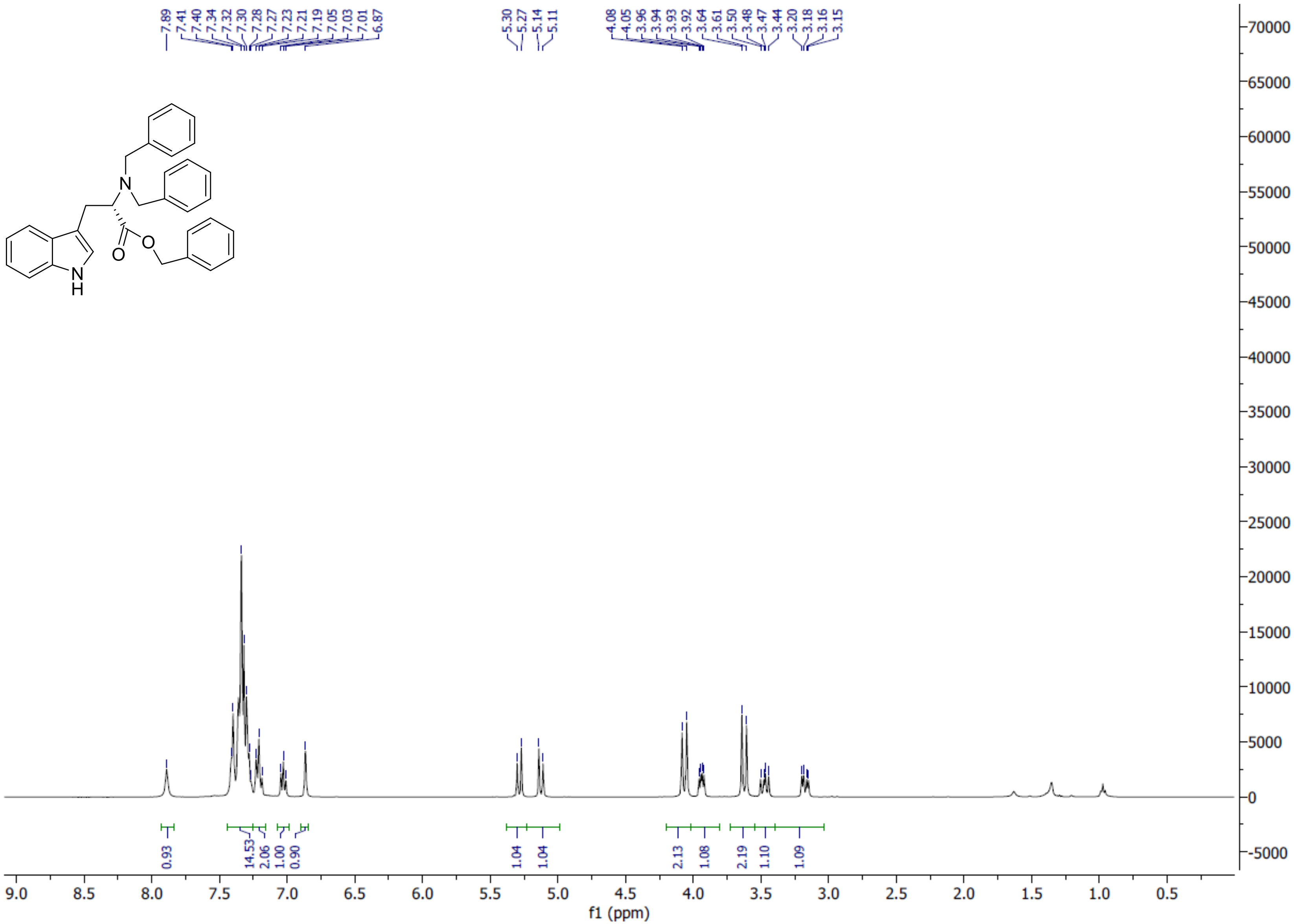

Figure S16: ^{1}H NMR spectrum of compound **9**

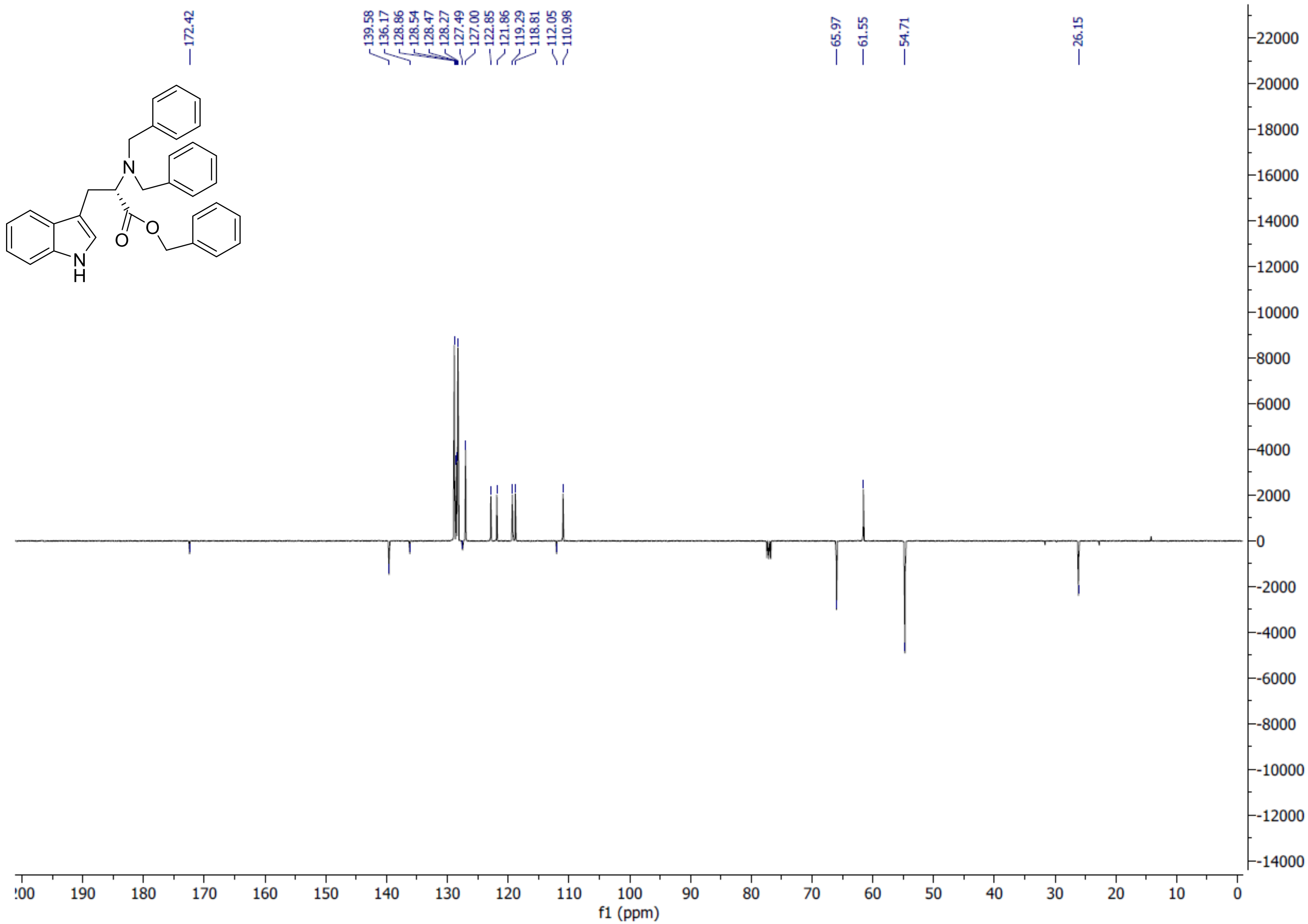

Figure S17: qDEPT spectrum of compound **9**

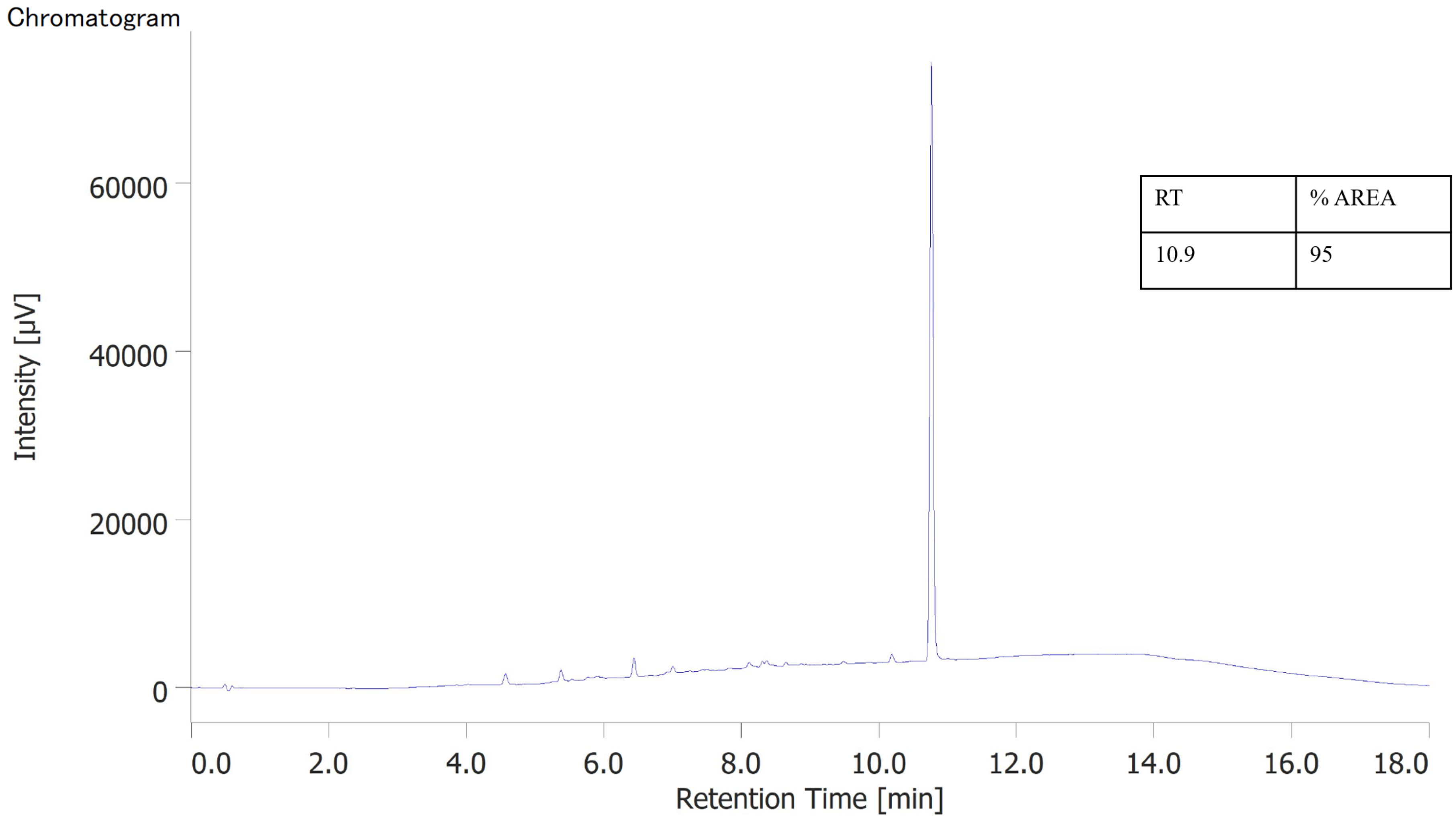

RT	% AREA
10.9	95

Figure S18: HPLC trace of compound **9**

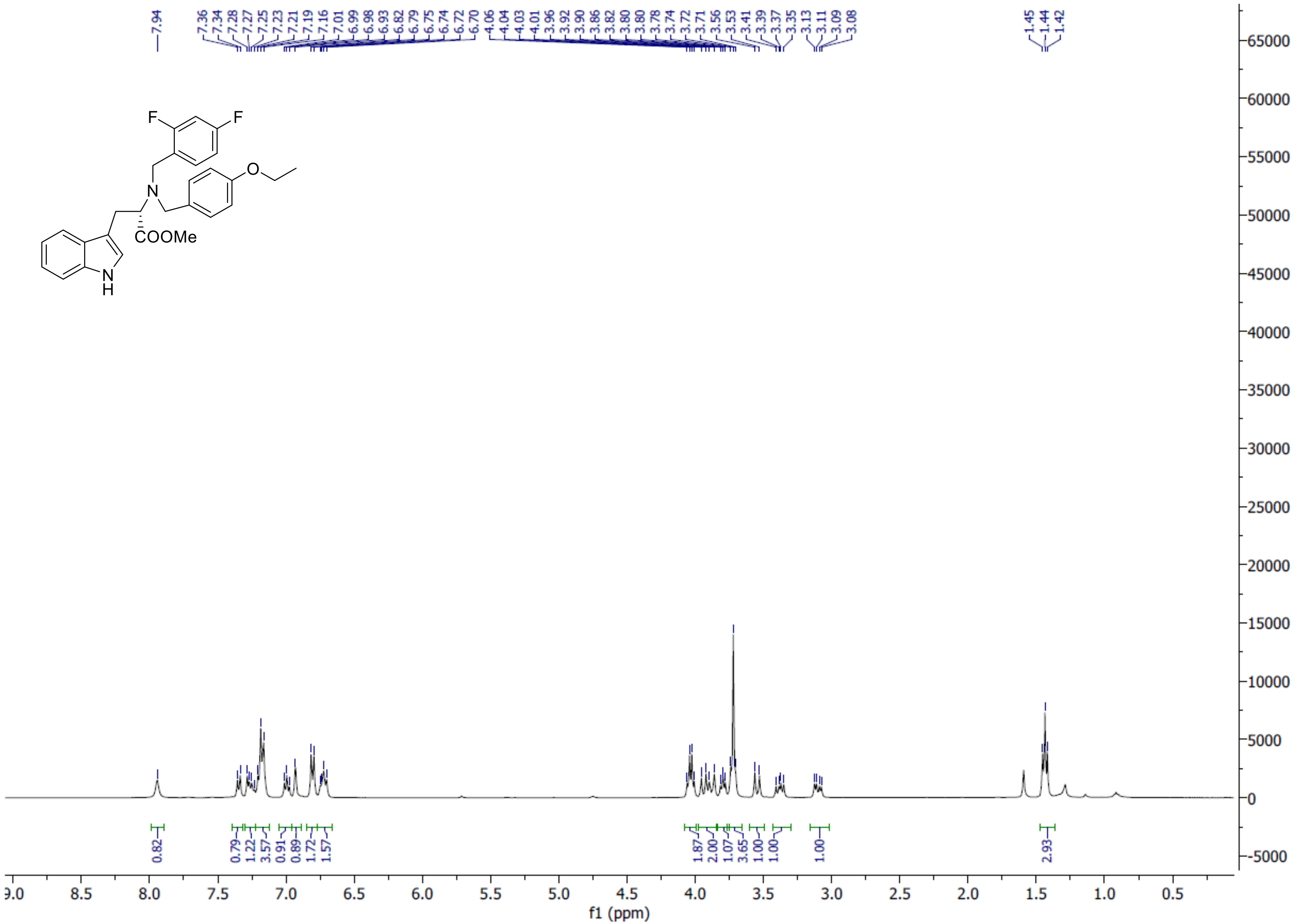

Figure S19: ^{1}H NMR spectrum of compound **10**

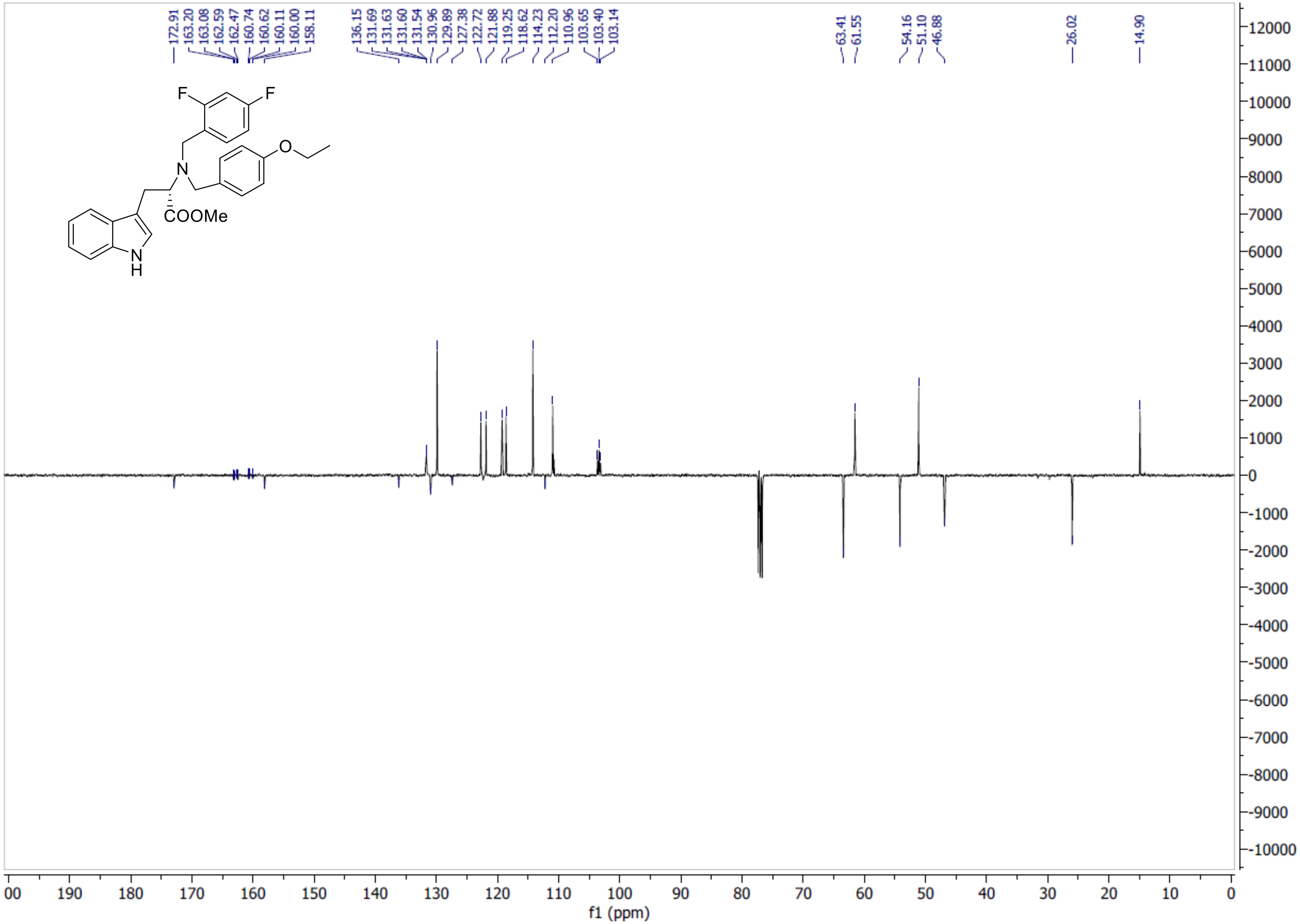

Figure S20: qDEPT spectrum of compound **10**

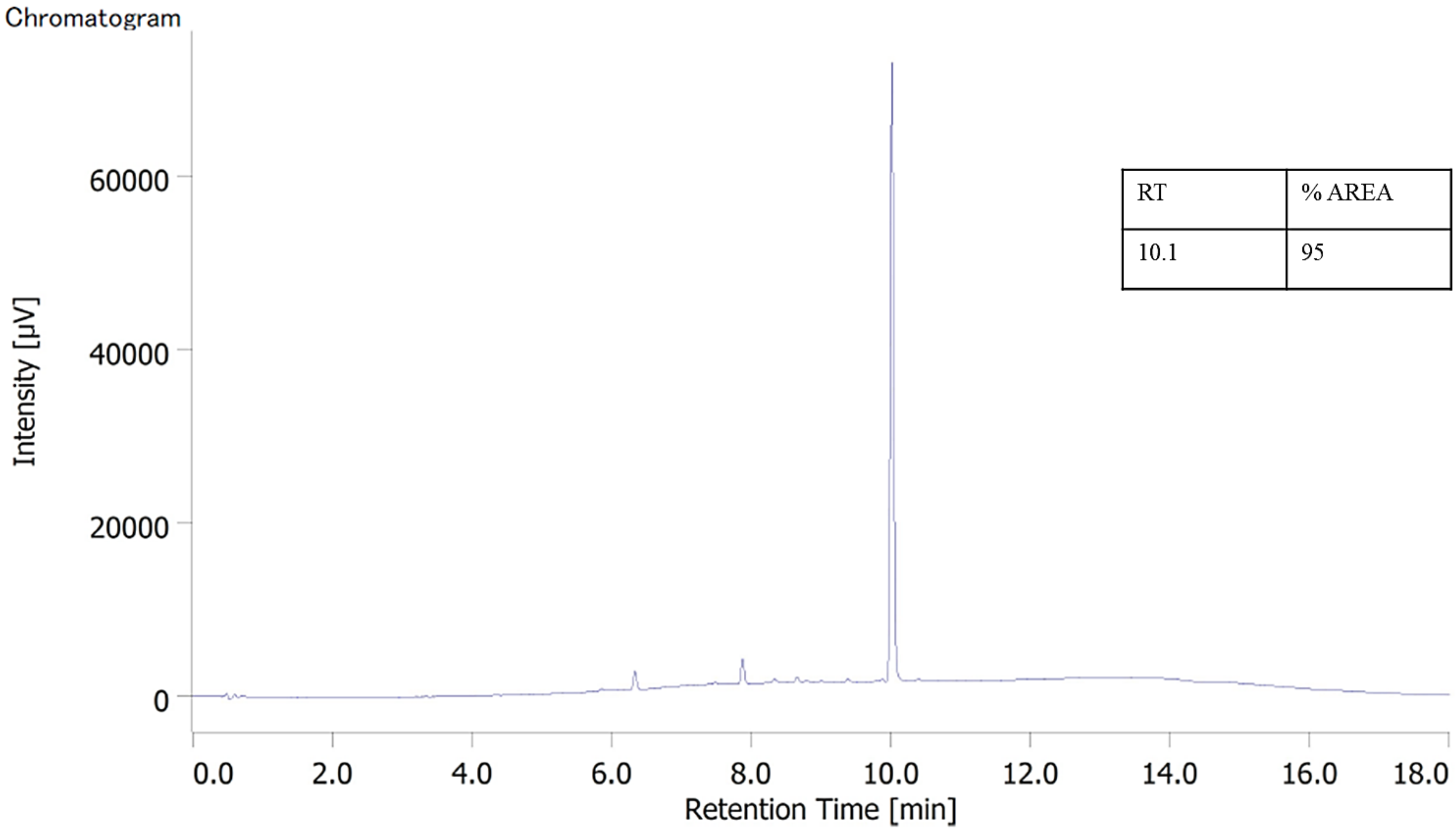

RT	% AREA
10.1	95

Figure S21: HPLC trace of compound **10**

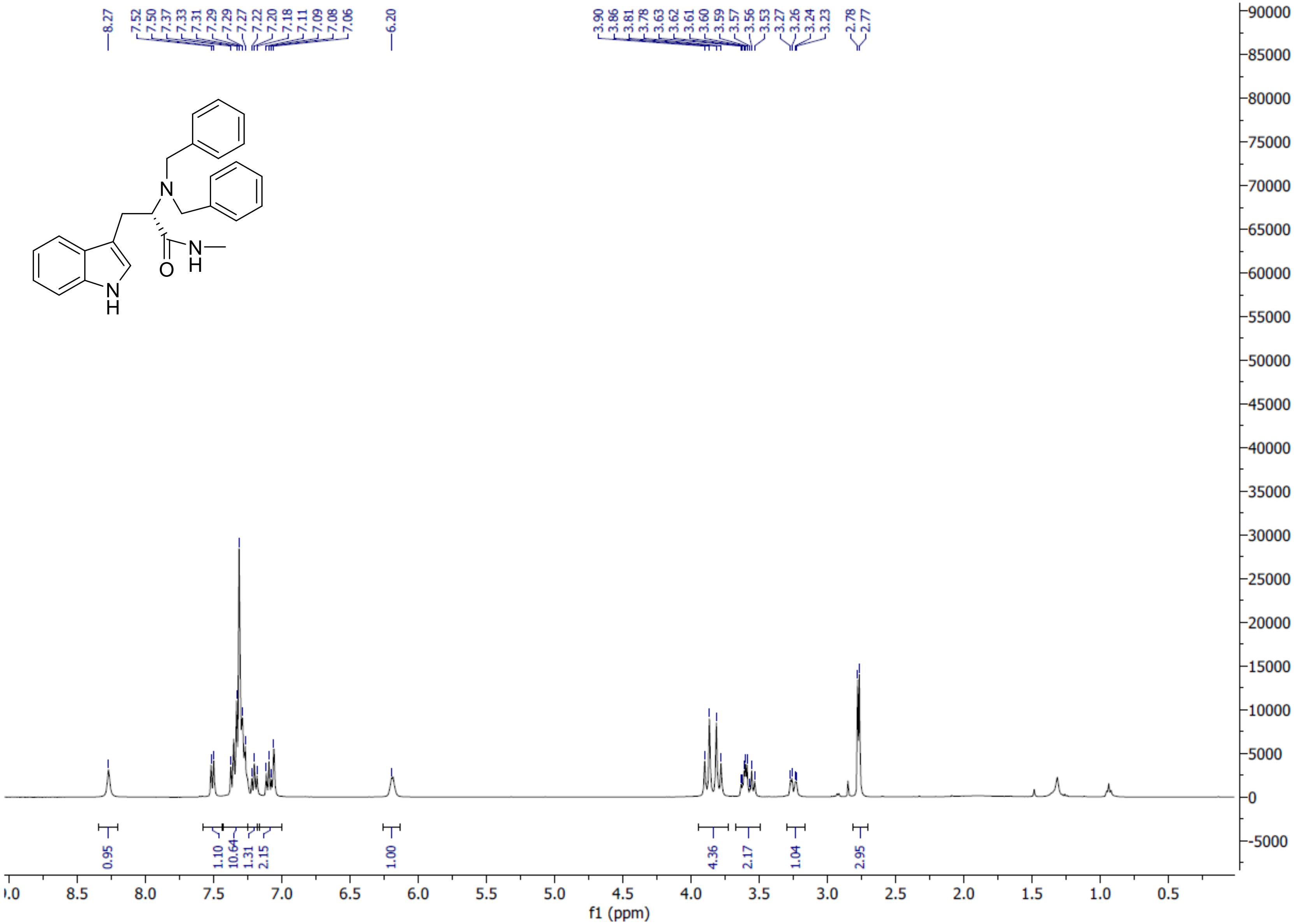

Figure S22: ^{1}H NMR spectrum of compound **12**

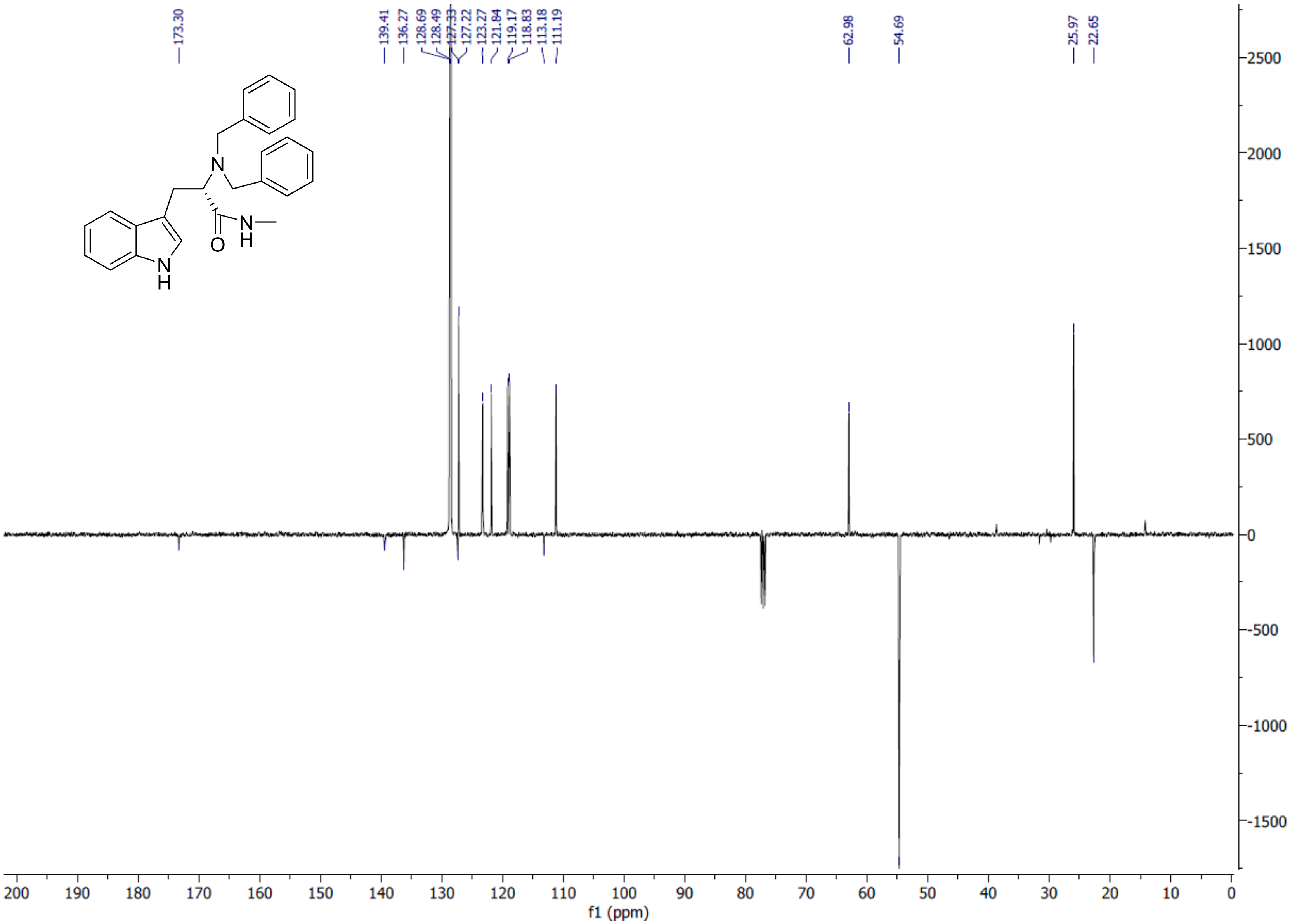

Figure S23: qDEPT spectrum of compound **12**

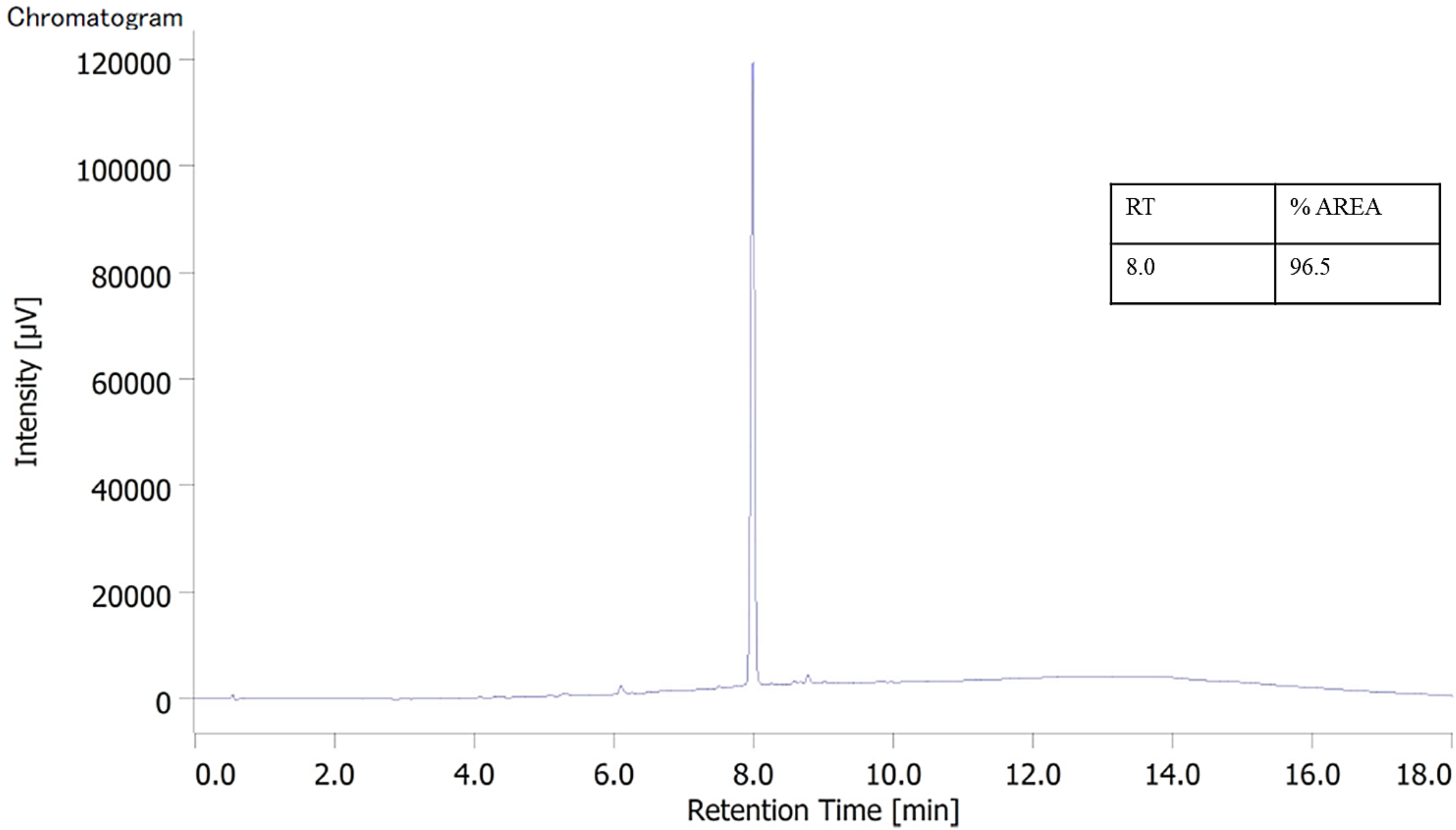

Figure S24: HPLC trace of compound **12**

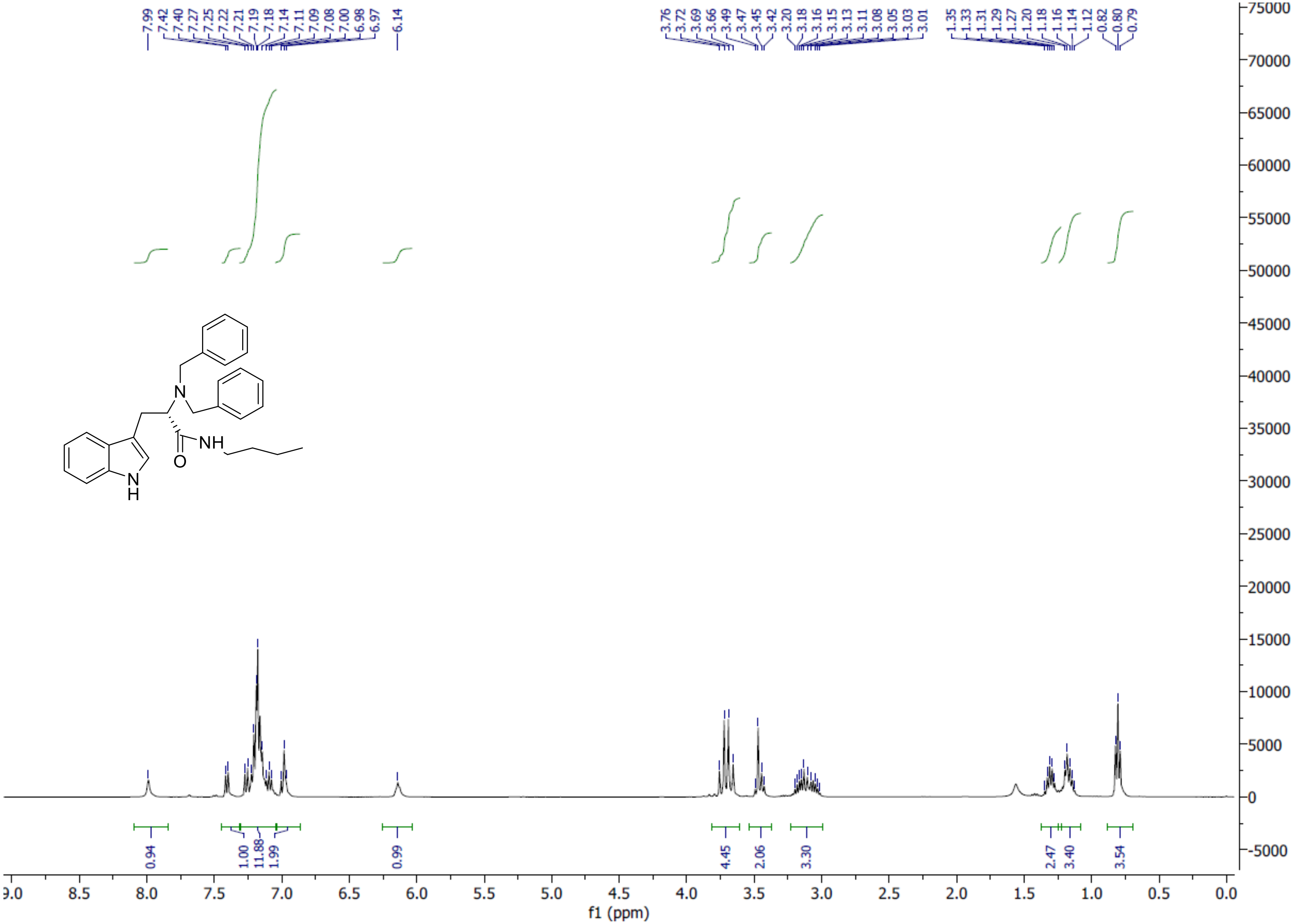

Figure S25: ^{1}H NMR spectrum of compound **13**

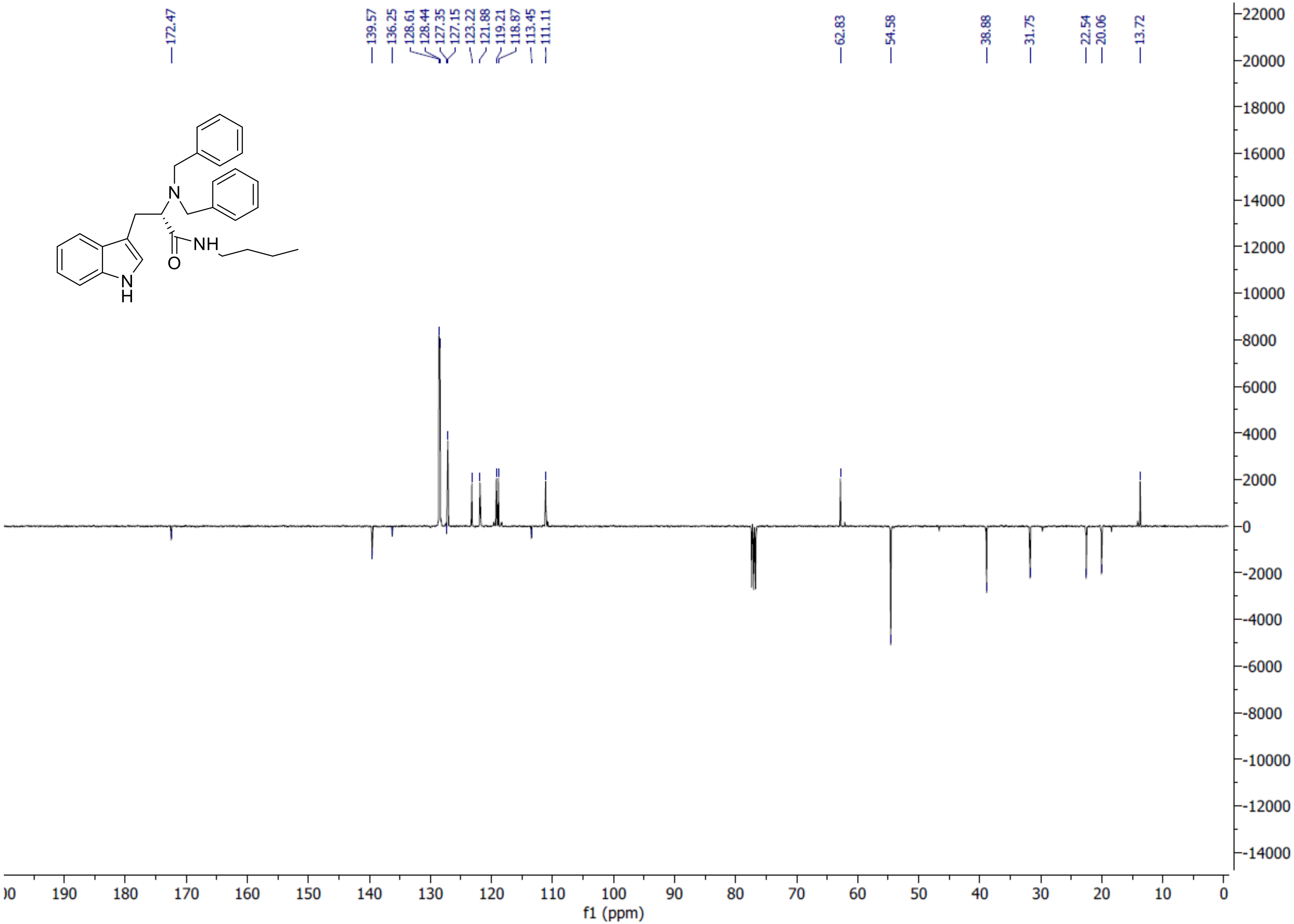

Figure S26: qDEPT spectrum of compound **13**

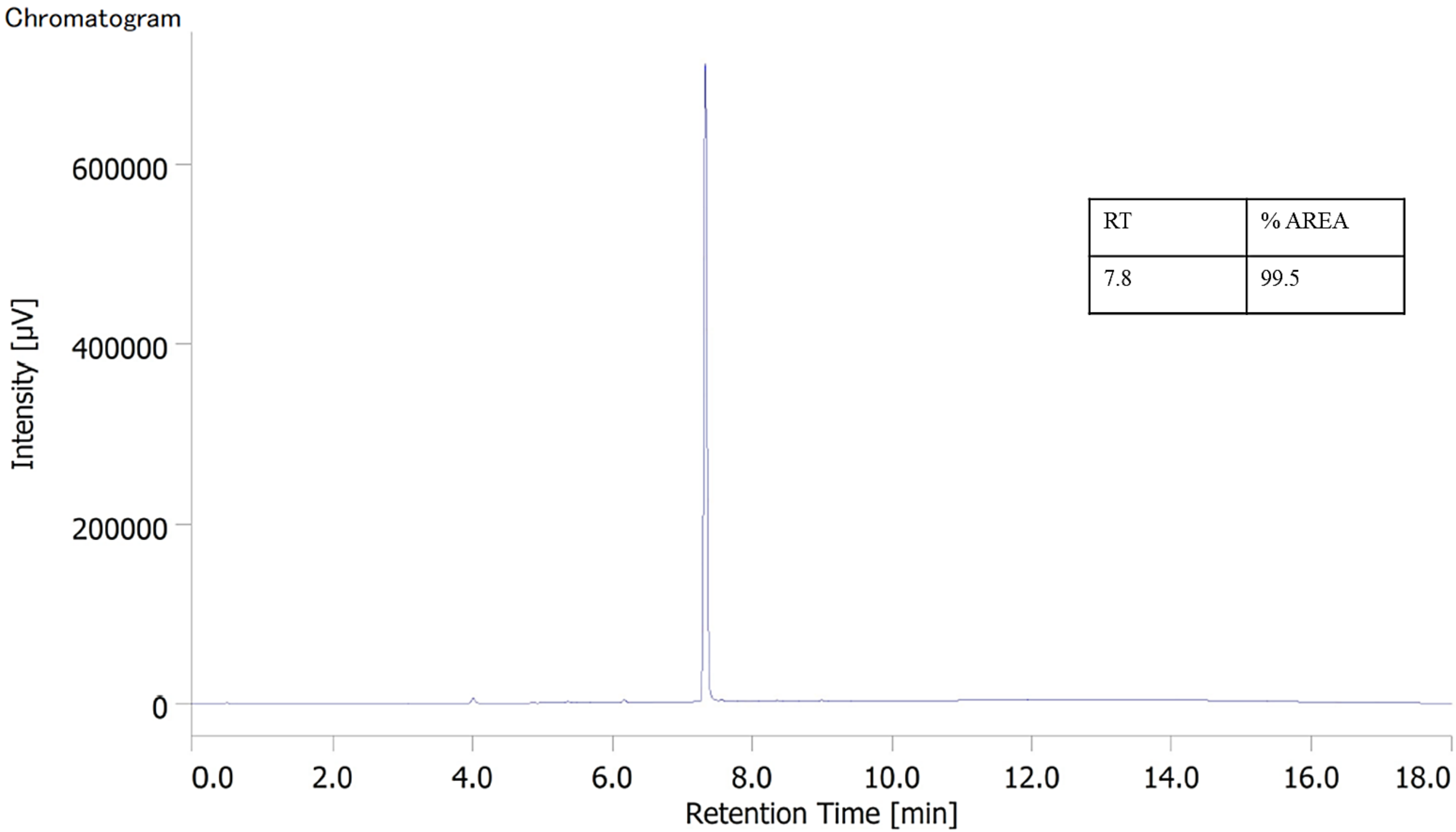

RT	% AREA
7.8	99.5

Figure S27: HPLC trace of compound **13**

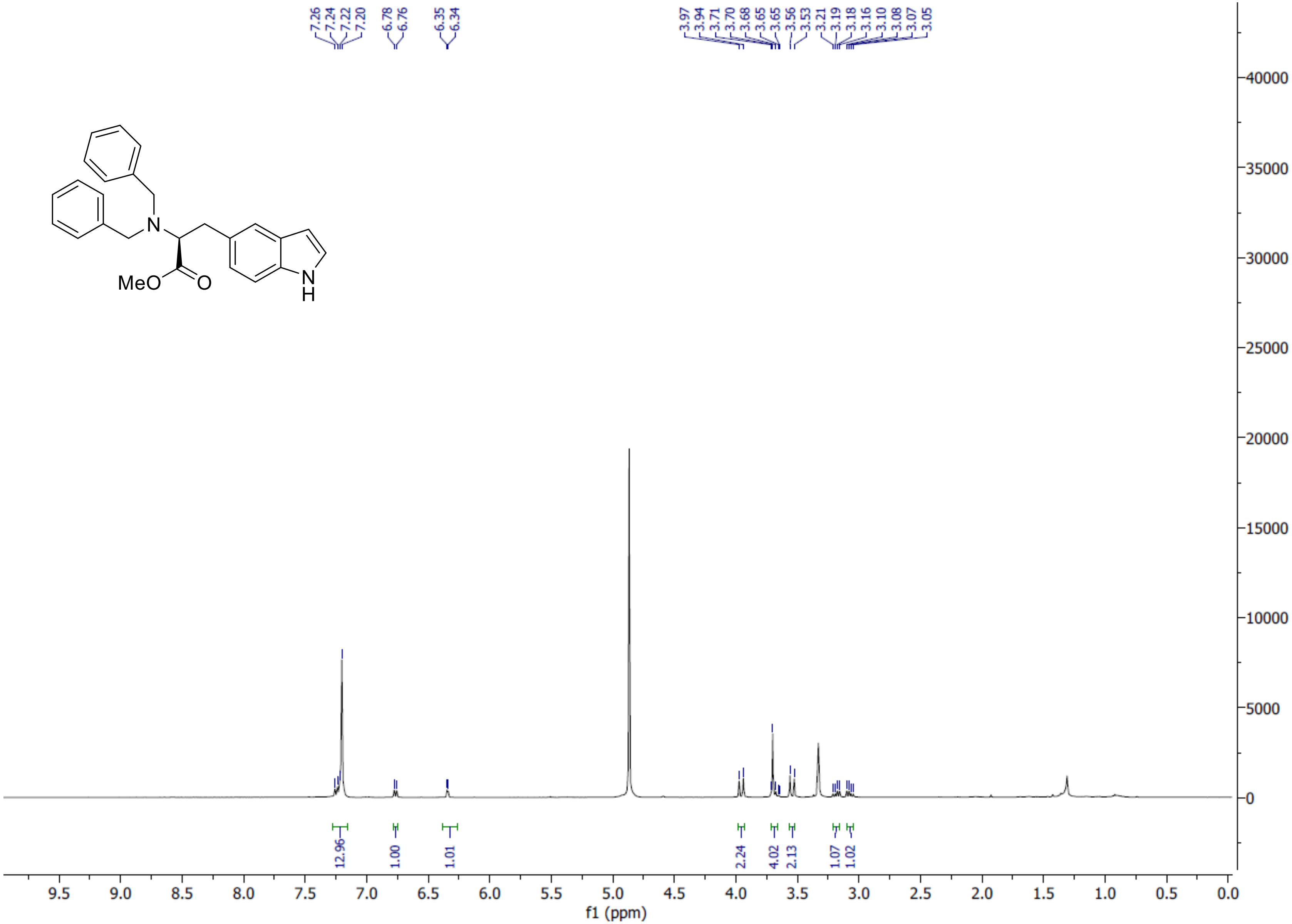

Figure S28: ^{1}H NMR spectrum of compound **23**

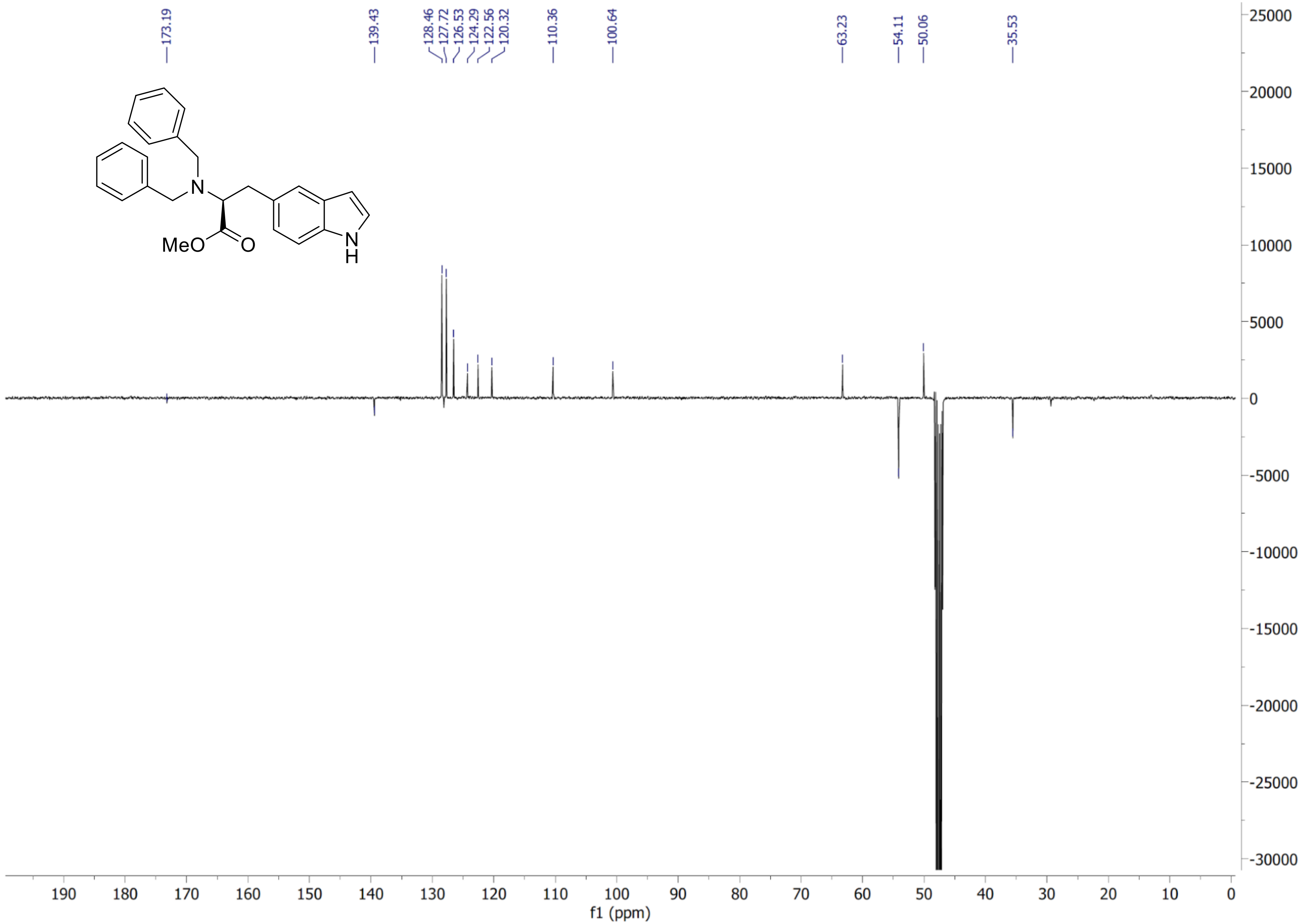

Figure S29: qDEPT spectrum of compound **23**

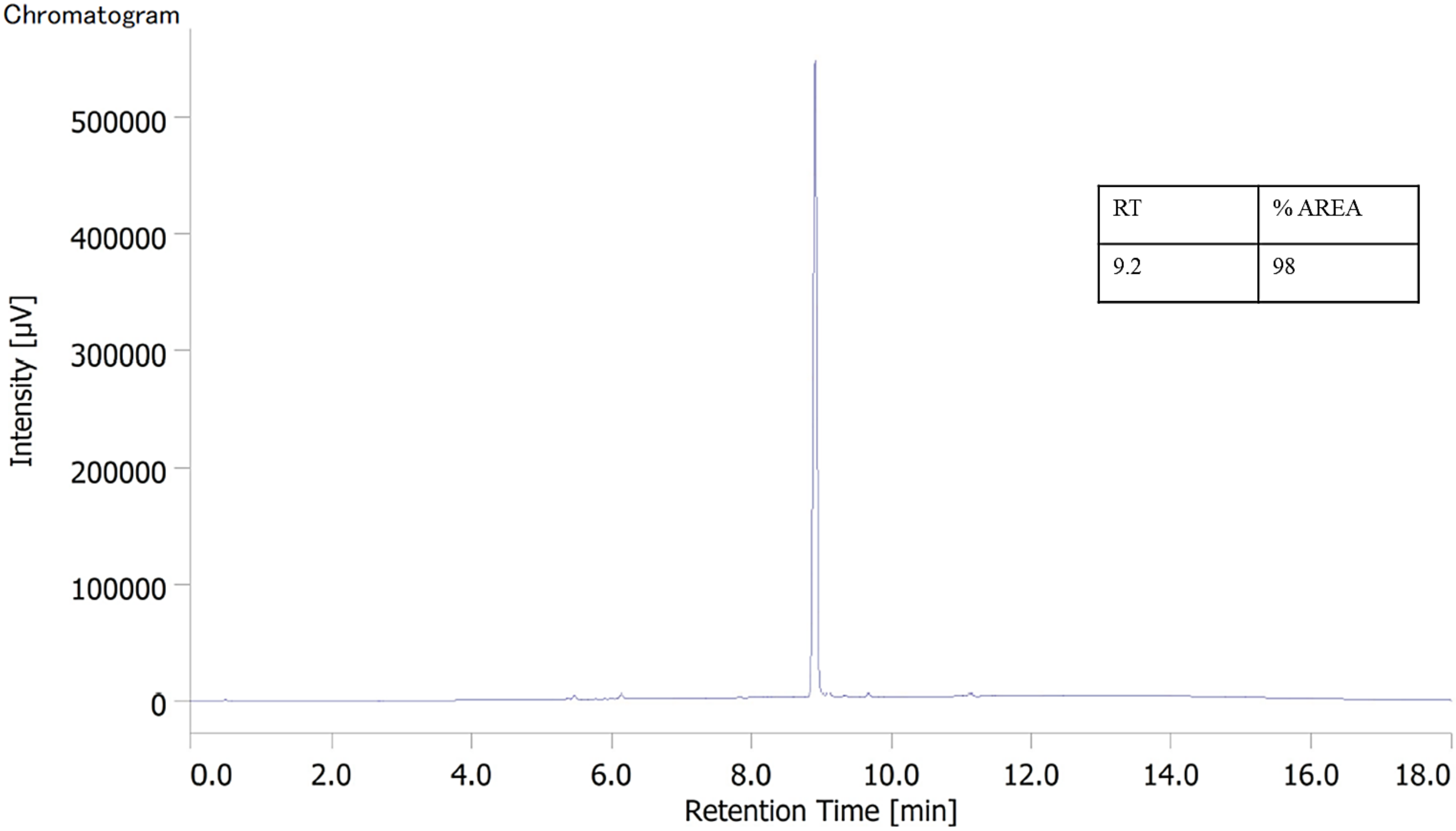

RT	% AREA
9.2	98

Figure S30: HPLC trace of compound **23**

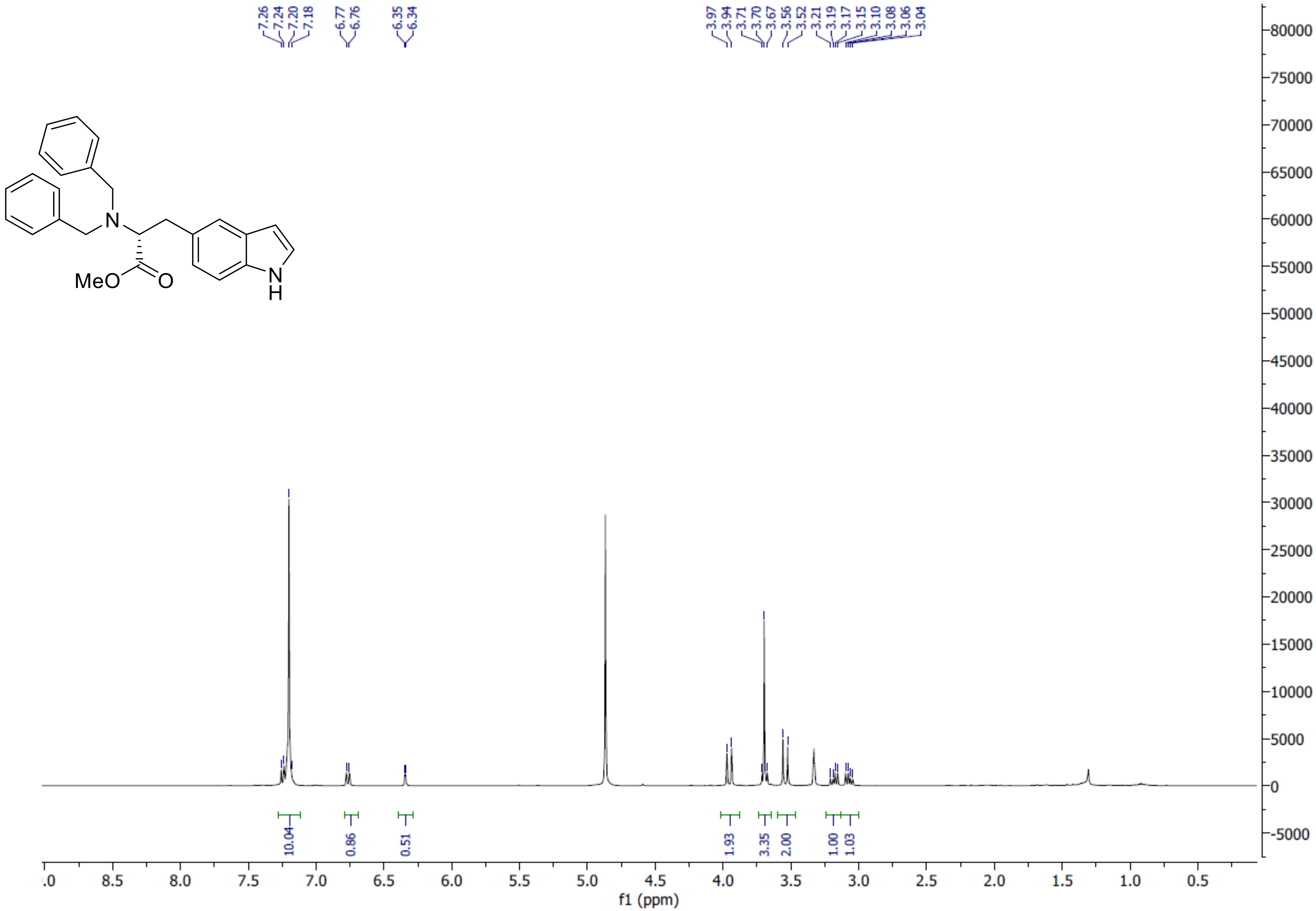

Figure S31: ^{1}H NMR spectrum of compound **24**

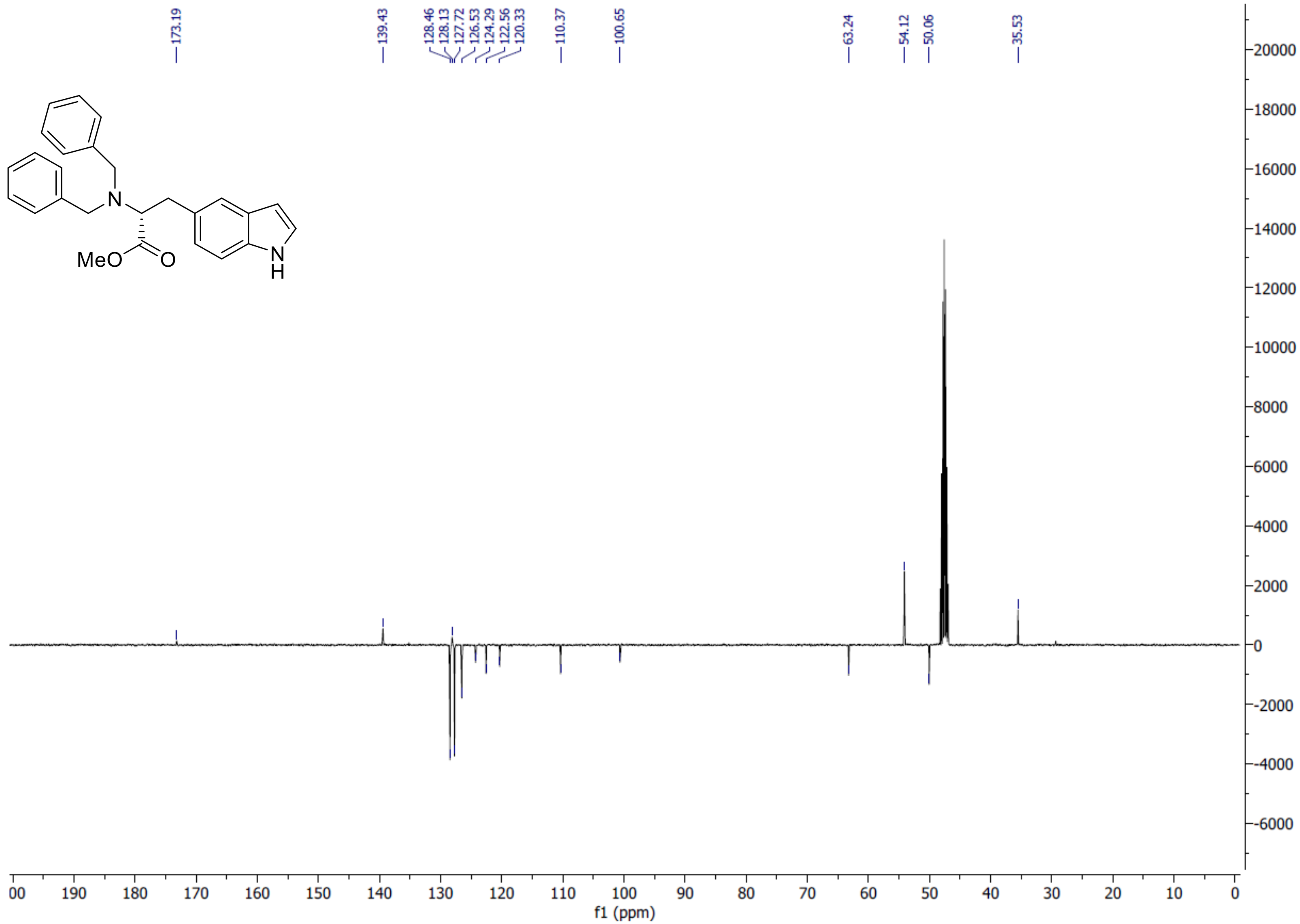

Figure S32: qDEPT spectrum of compound **24**

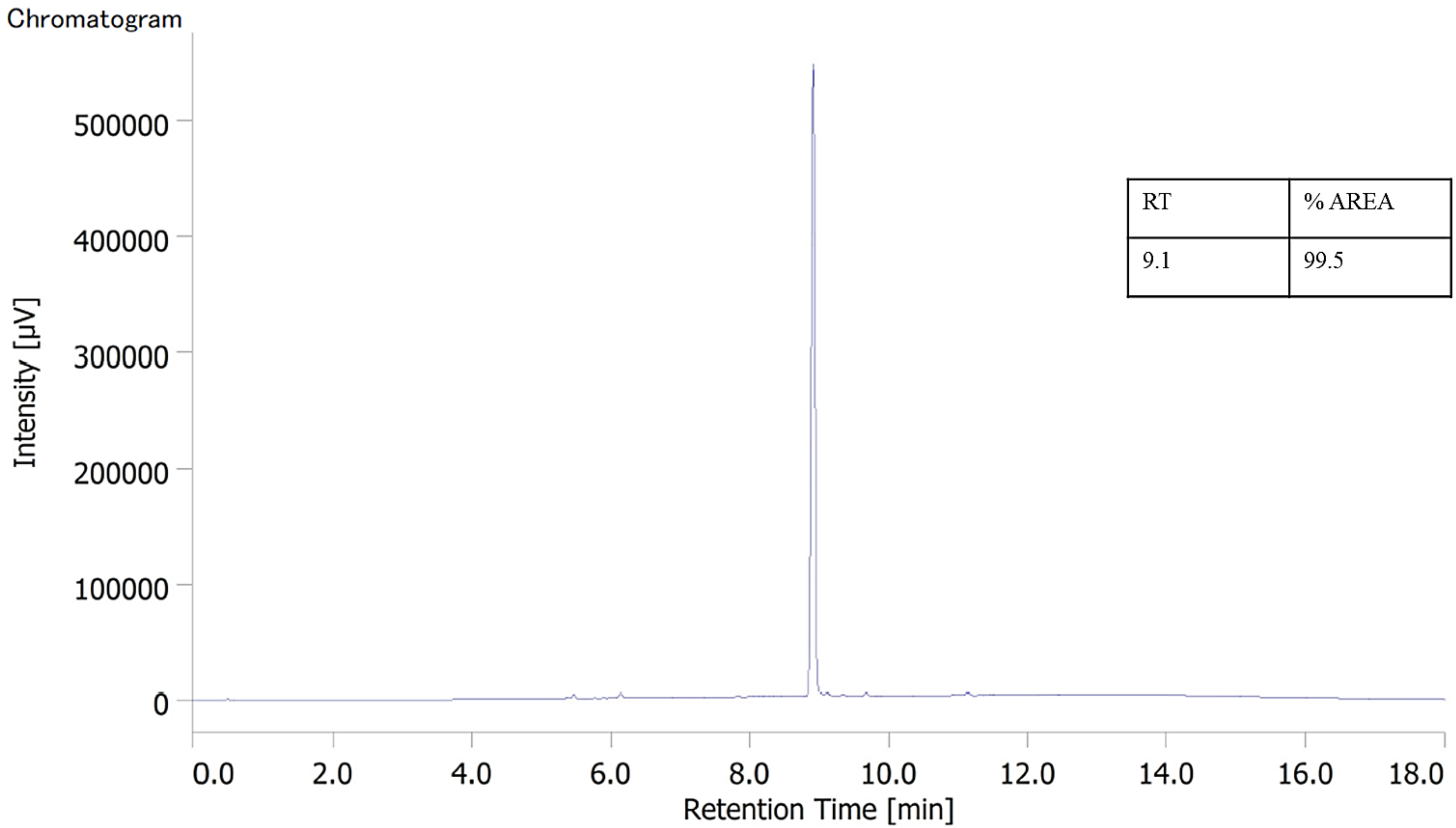

RT	% AREA
9.1	99.5

Figure S33: HPLC trace of compound **24**

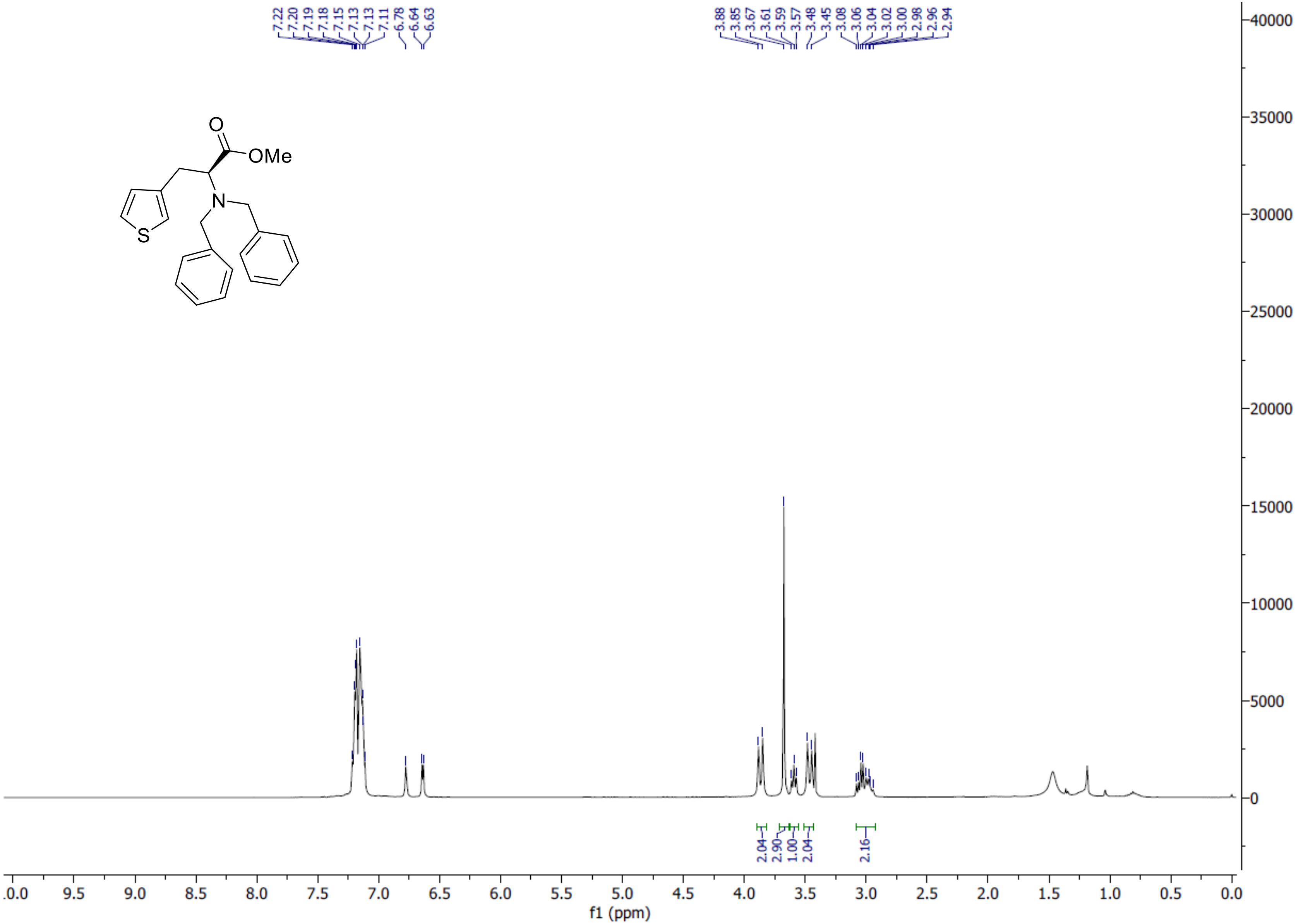

Figure S34: ^{1}H NMR spectrum of compound **25**

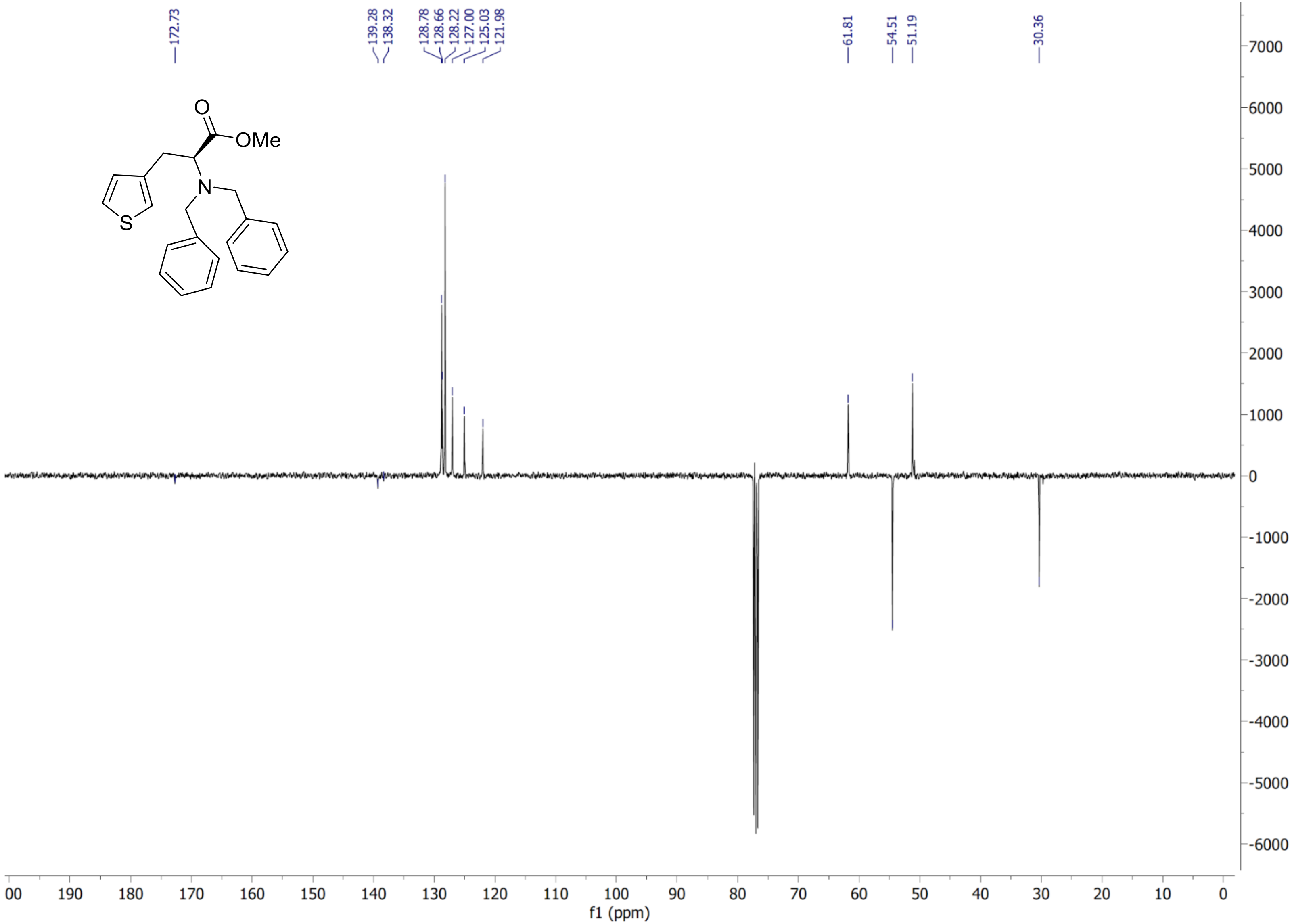

Figure S35: qDEPT spectrum of compound **25**

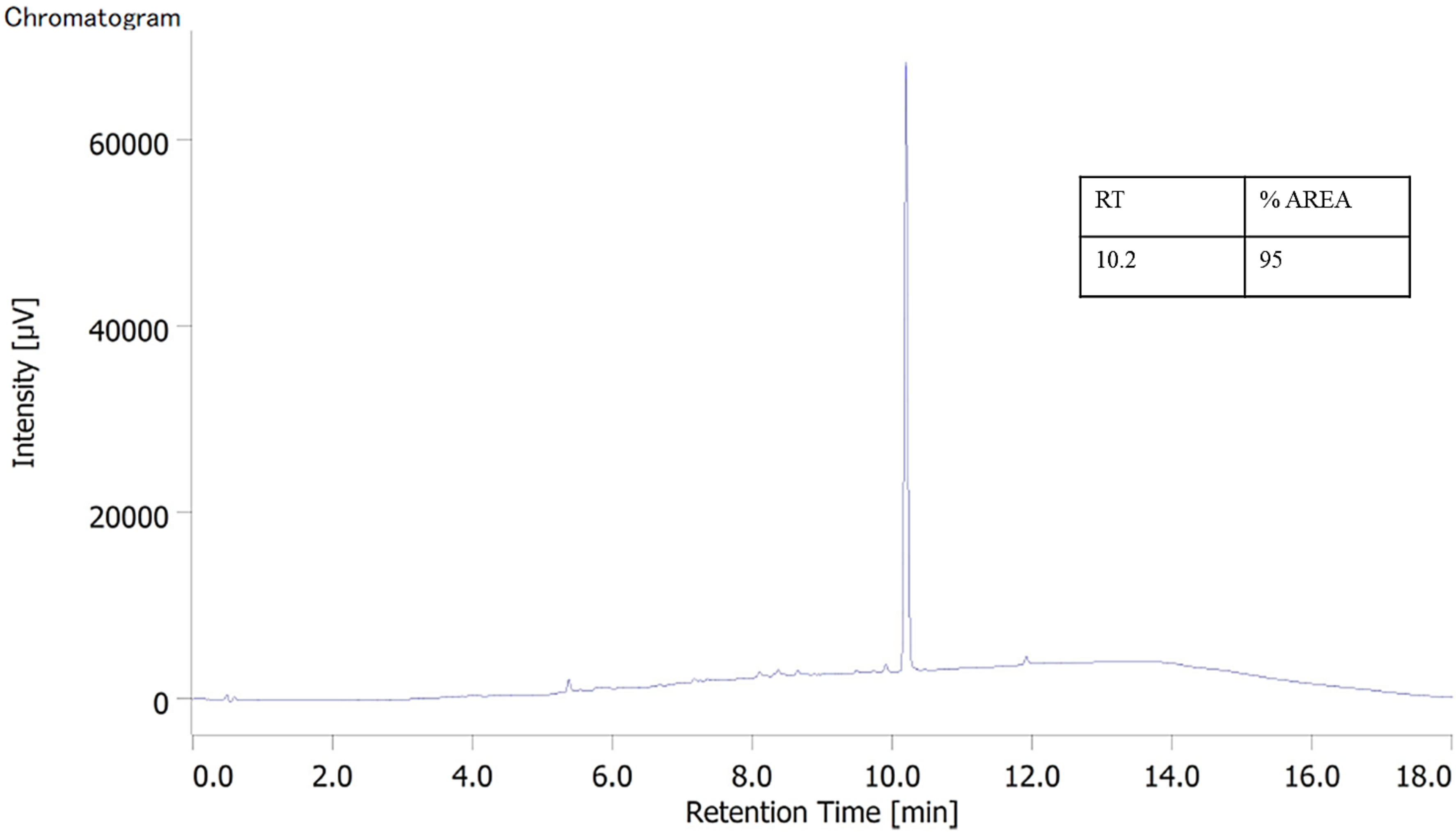

RT	% AREA
10.2	95

Figure S36: HPLC trace of compound **25**

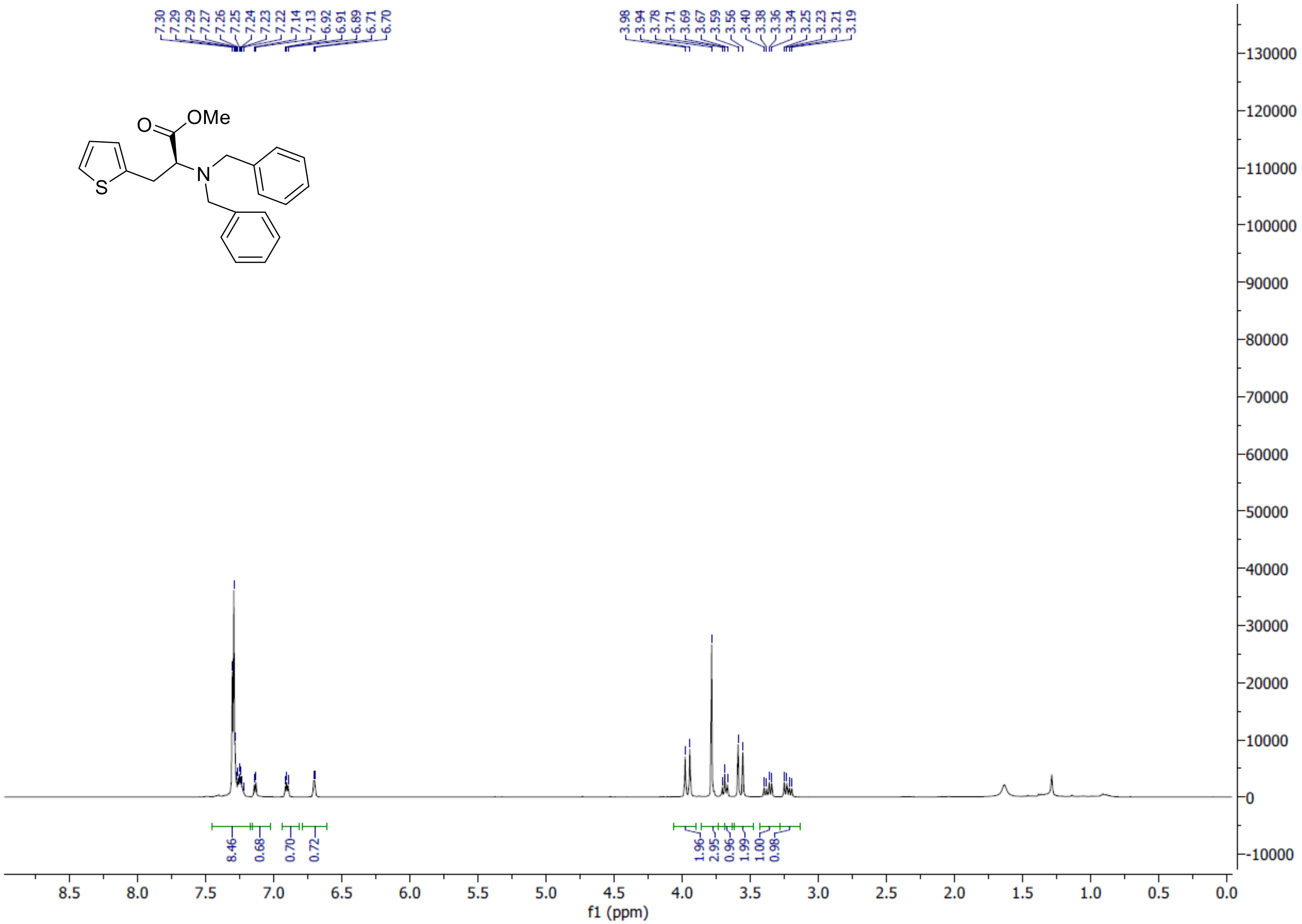

Figure S37: ^{1}H NMR spectrum of compound **26**

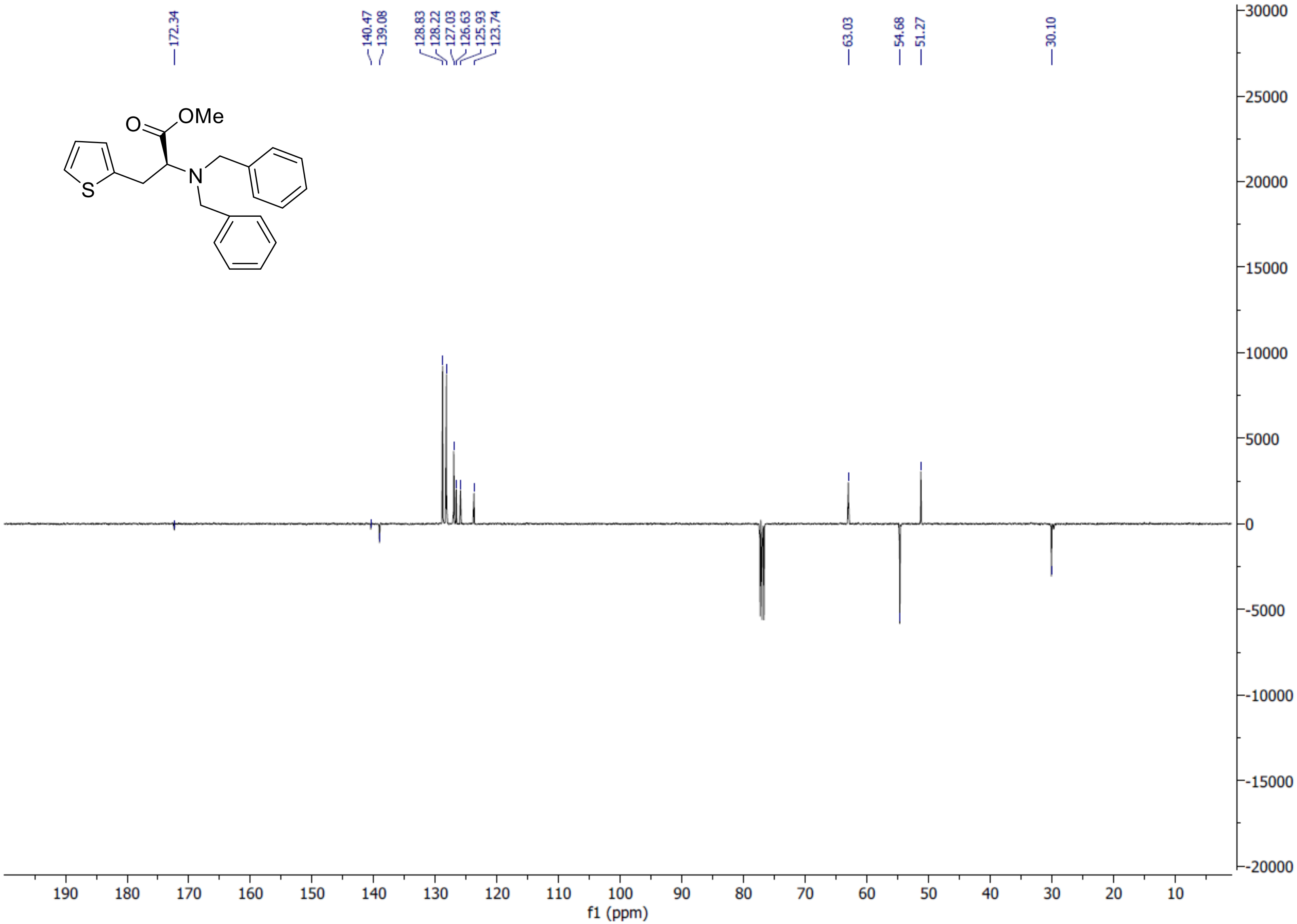

Figure S38: qDEPT spectrum of compound **26**

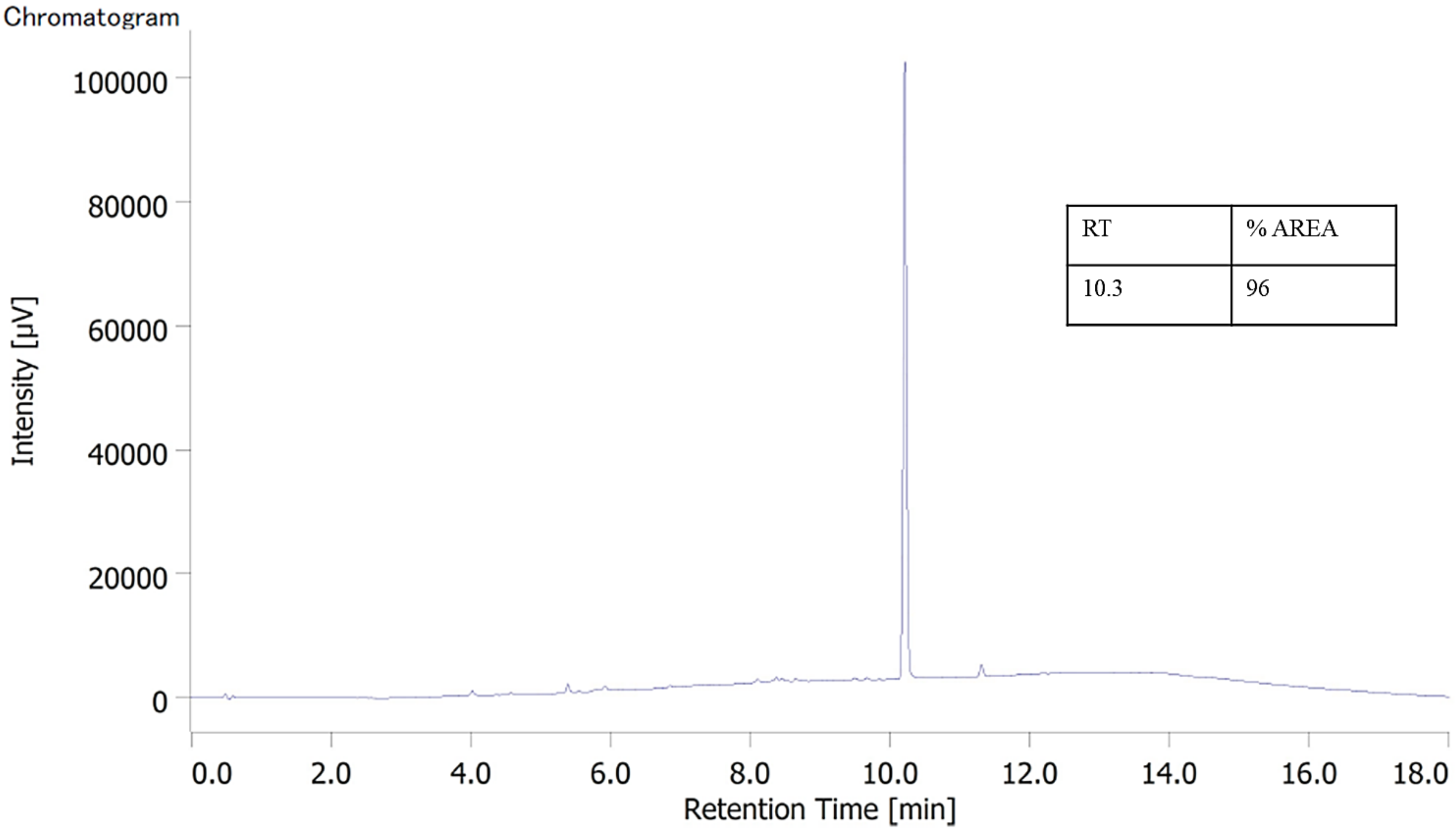

RT	% AREA
10.3	96

Figure S39: HPLC trace of compound **26**

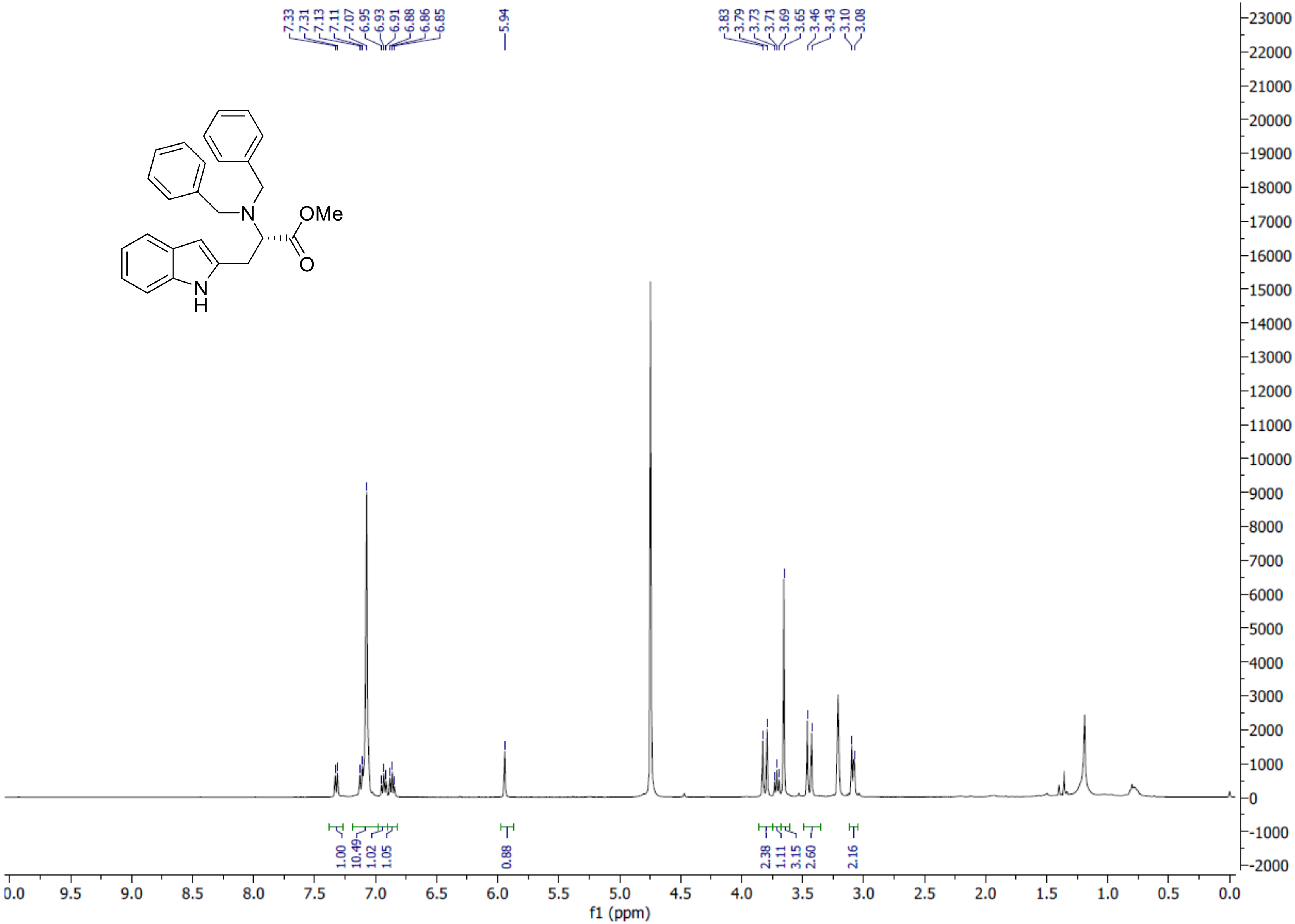

Figure S40: ^{1}H NMR spectrum of compound **27**

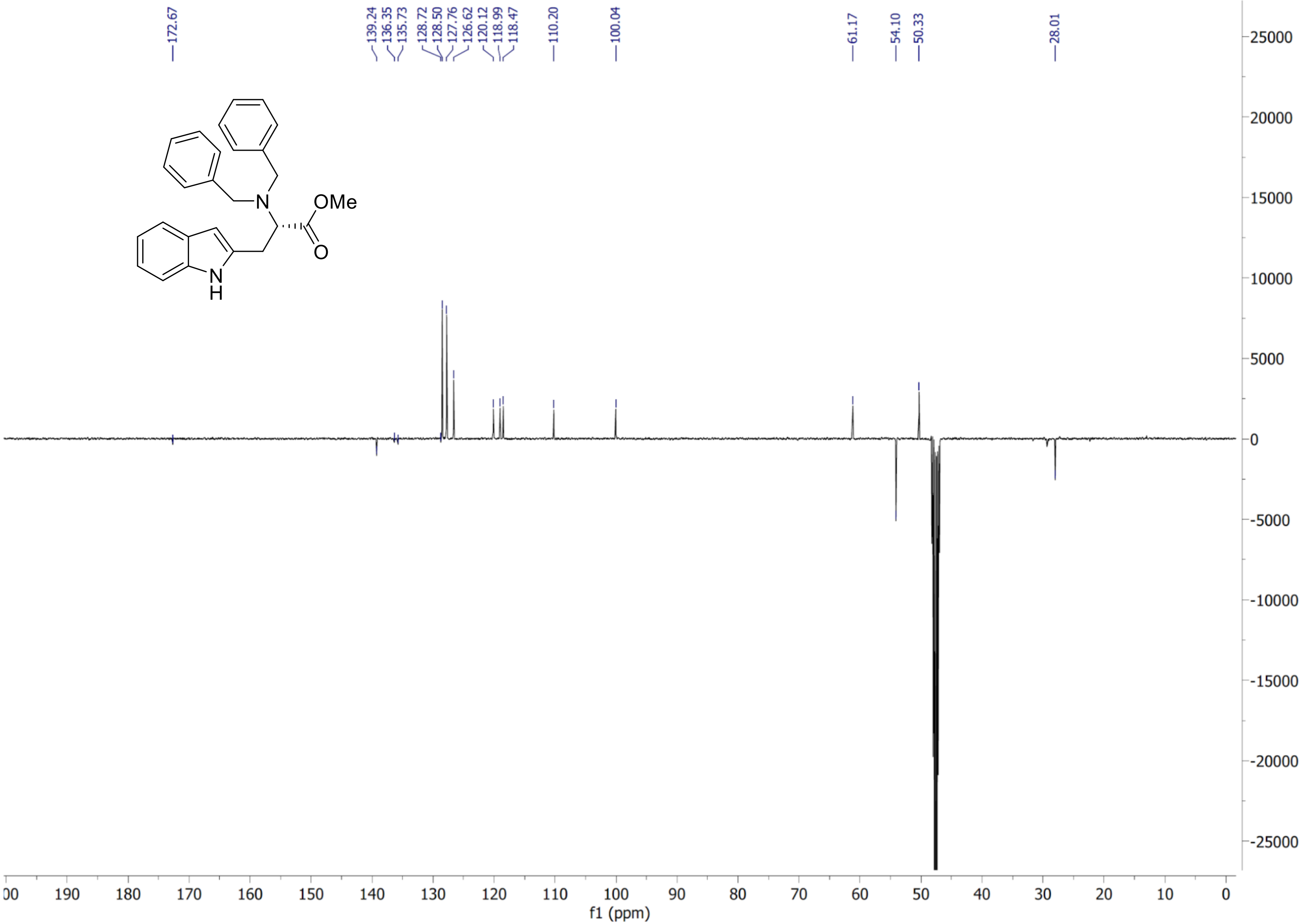

Figure S41: qDEPT spectrum of compound **27**

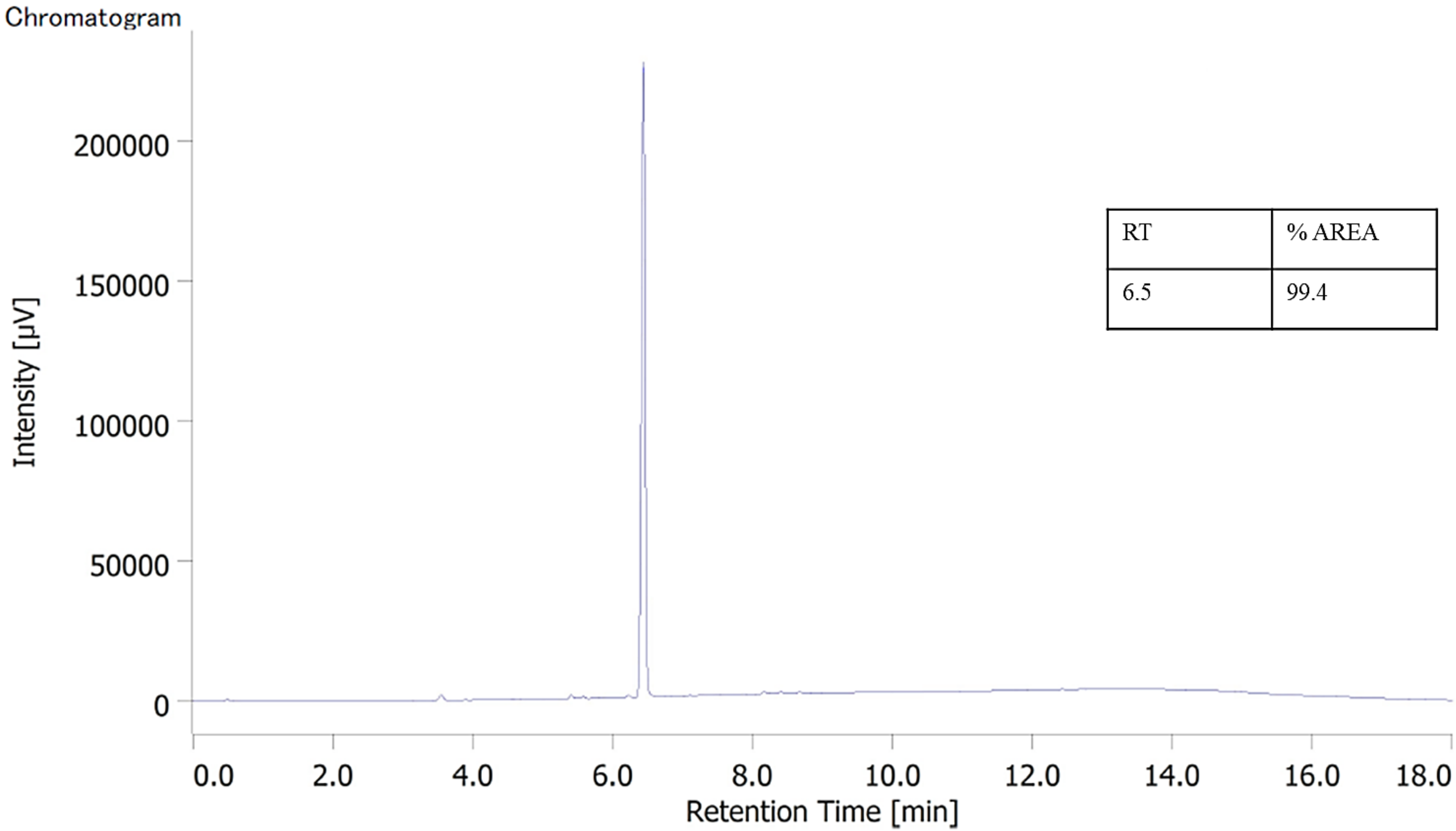

RT	% AREA
6.5	99.4

Figure S42: HPLC trace of compound **27**

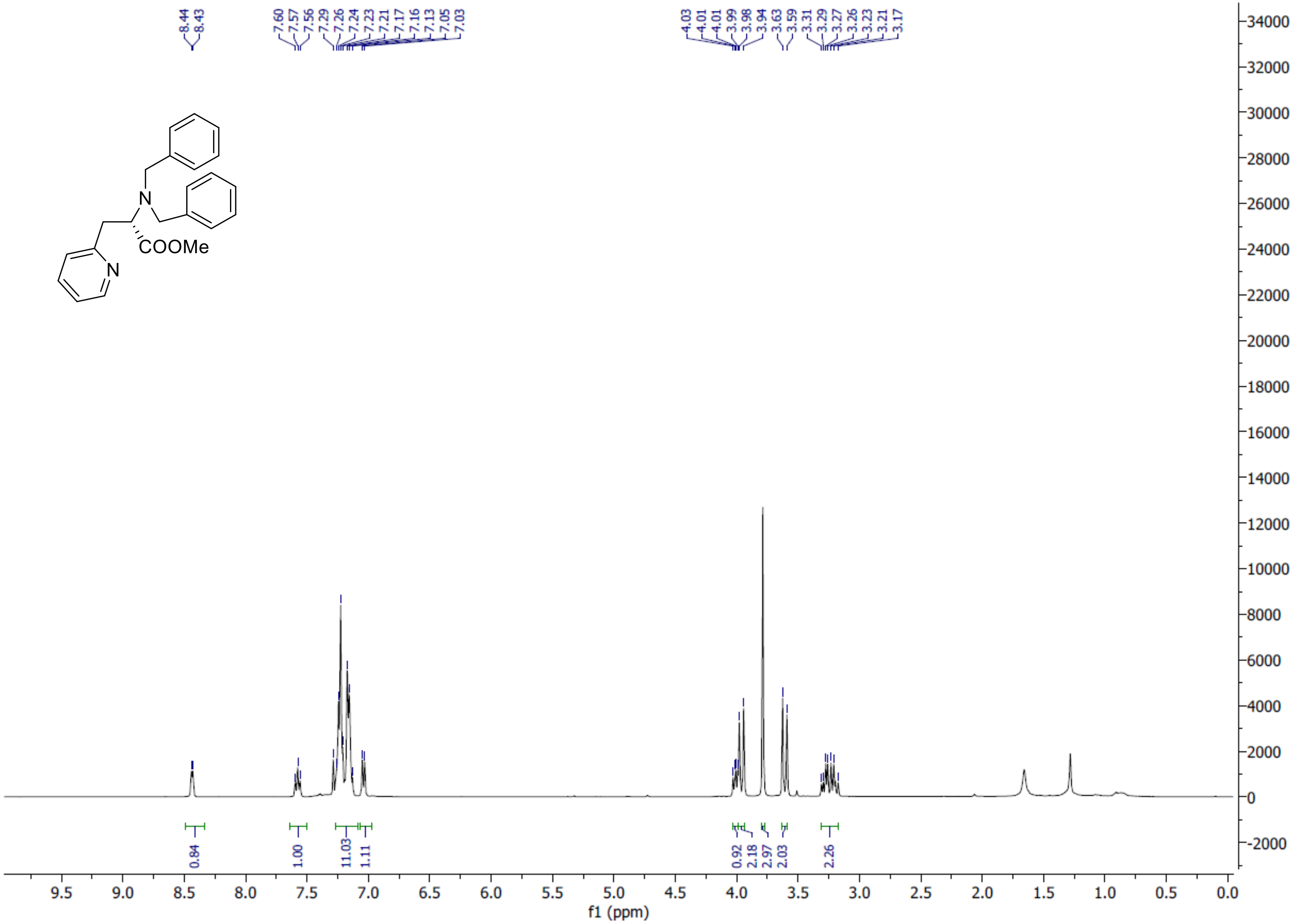

Figure S43: ^{1}H NMR spectrum of compound **28**

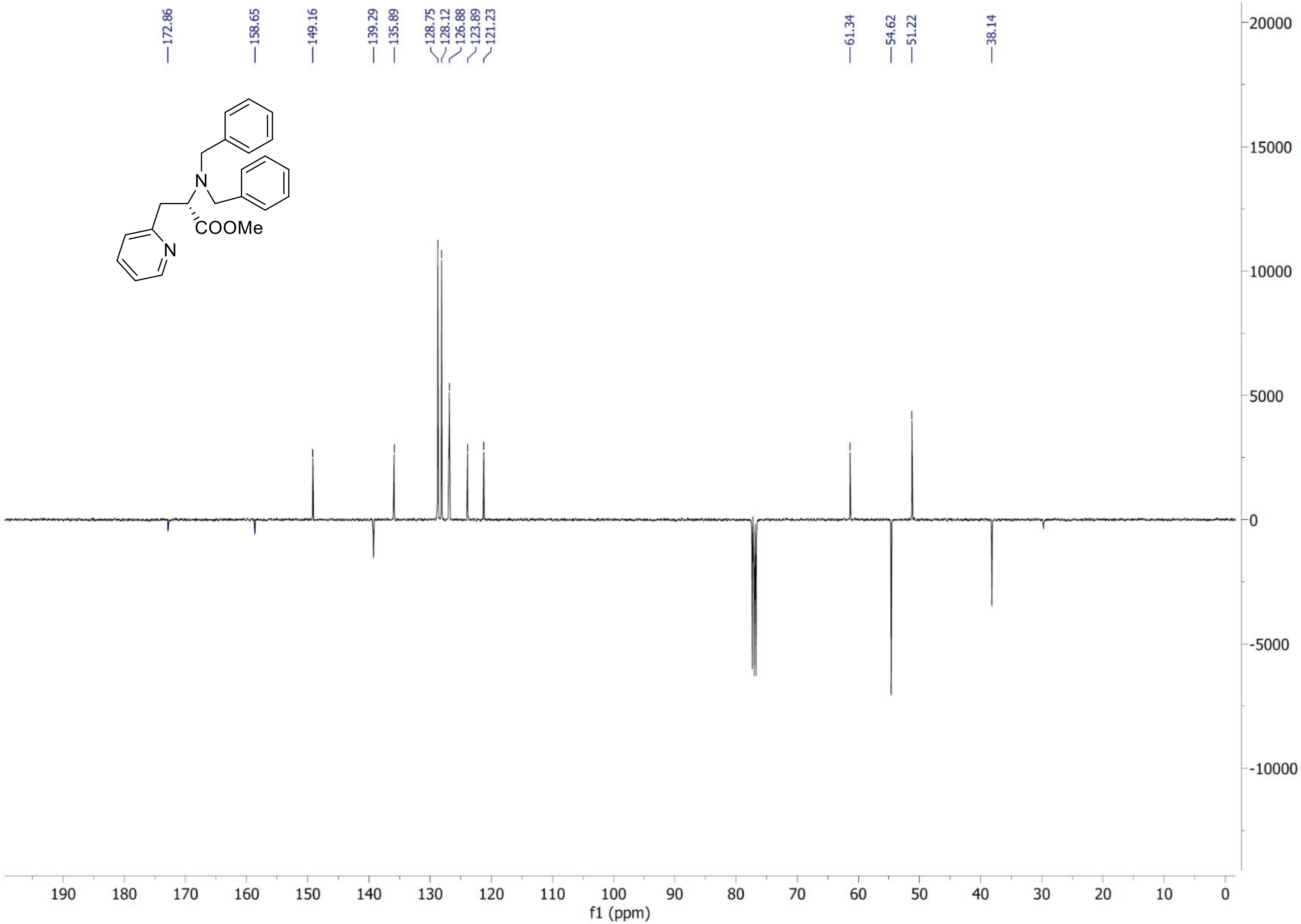

Figure S44: qDEPT spectrum of compound **28**

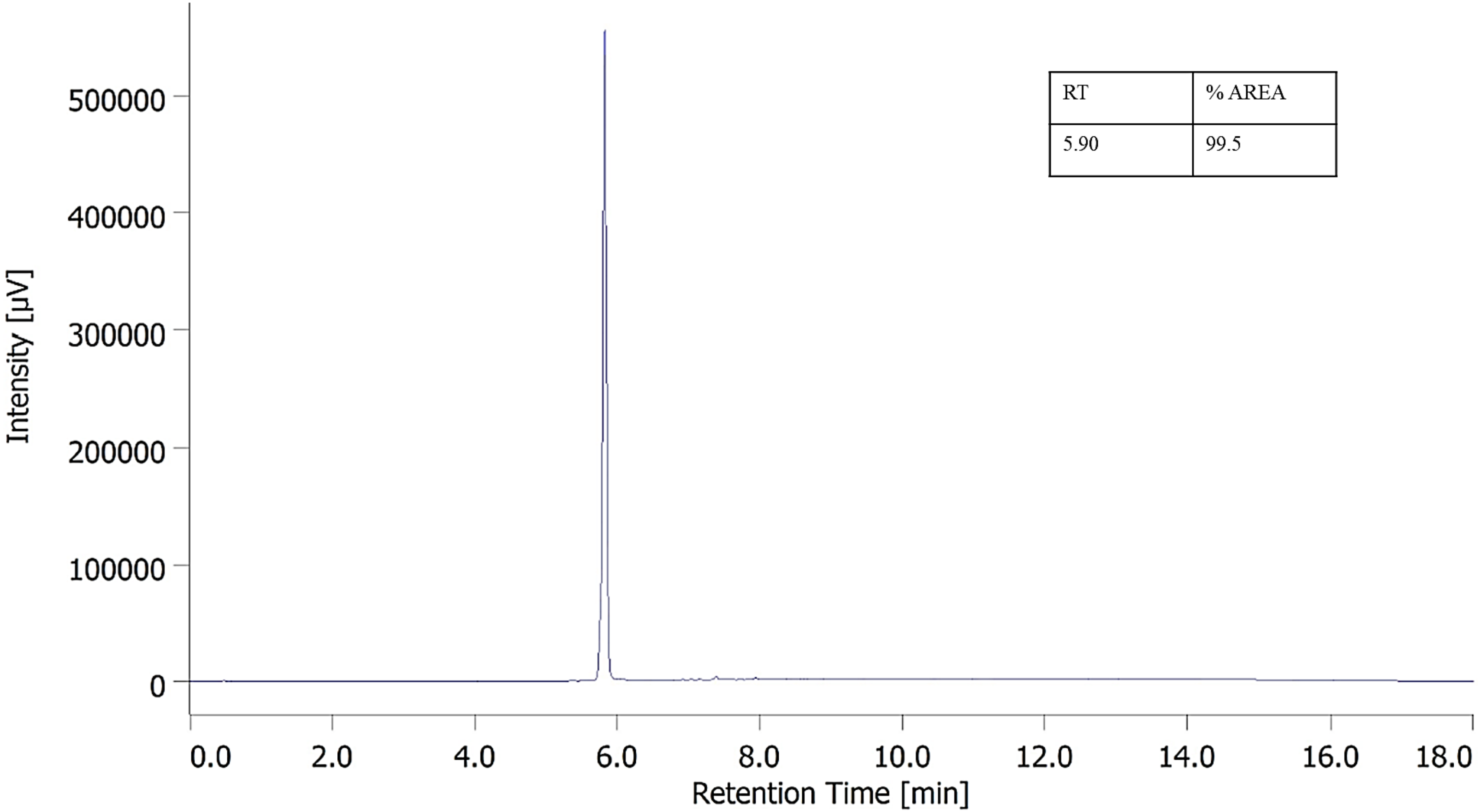

Figure S45: HPLC trace of compound **28**

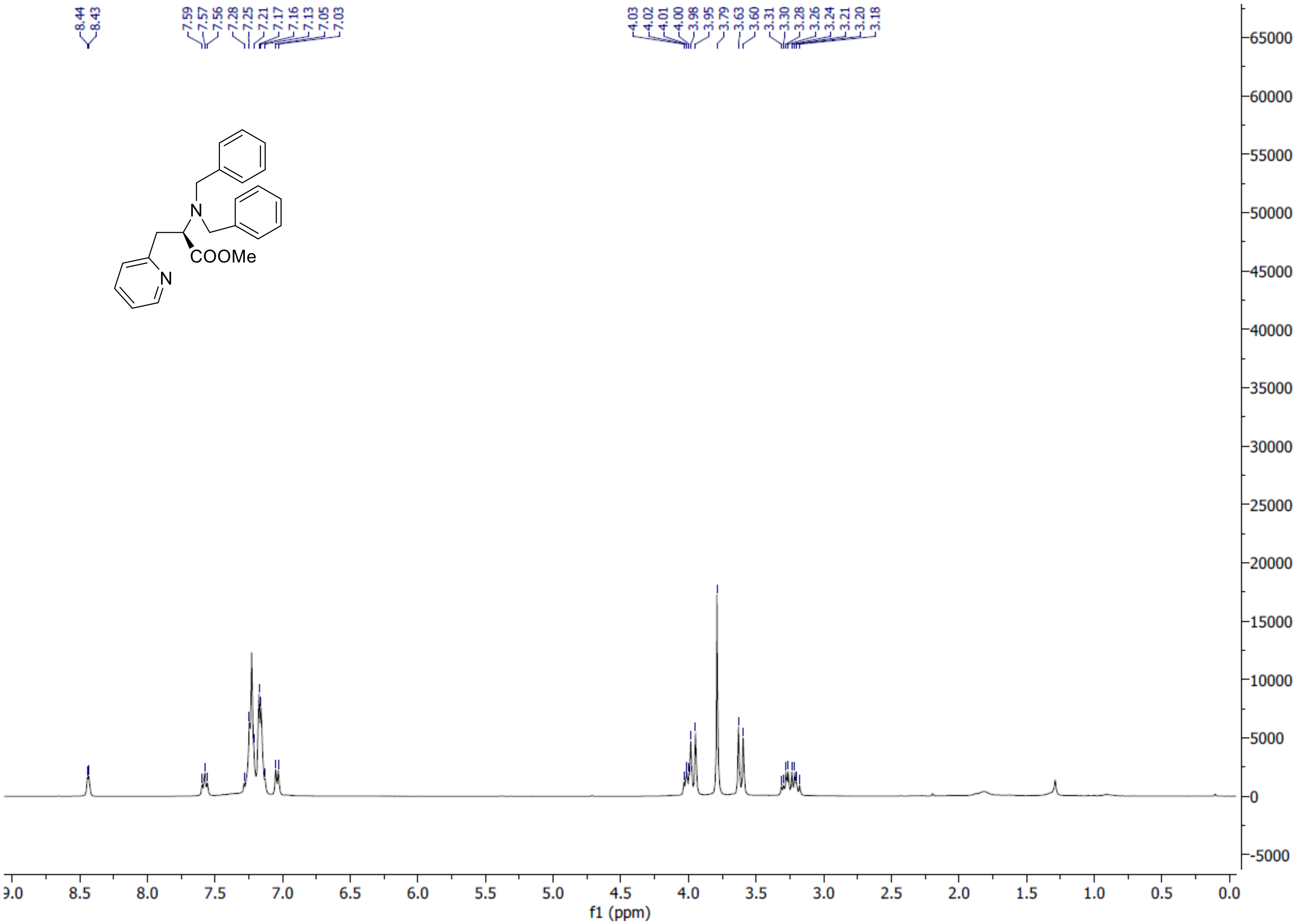

Figure S46: ^{1}H NMR spectrum of compound **29**

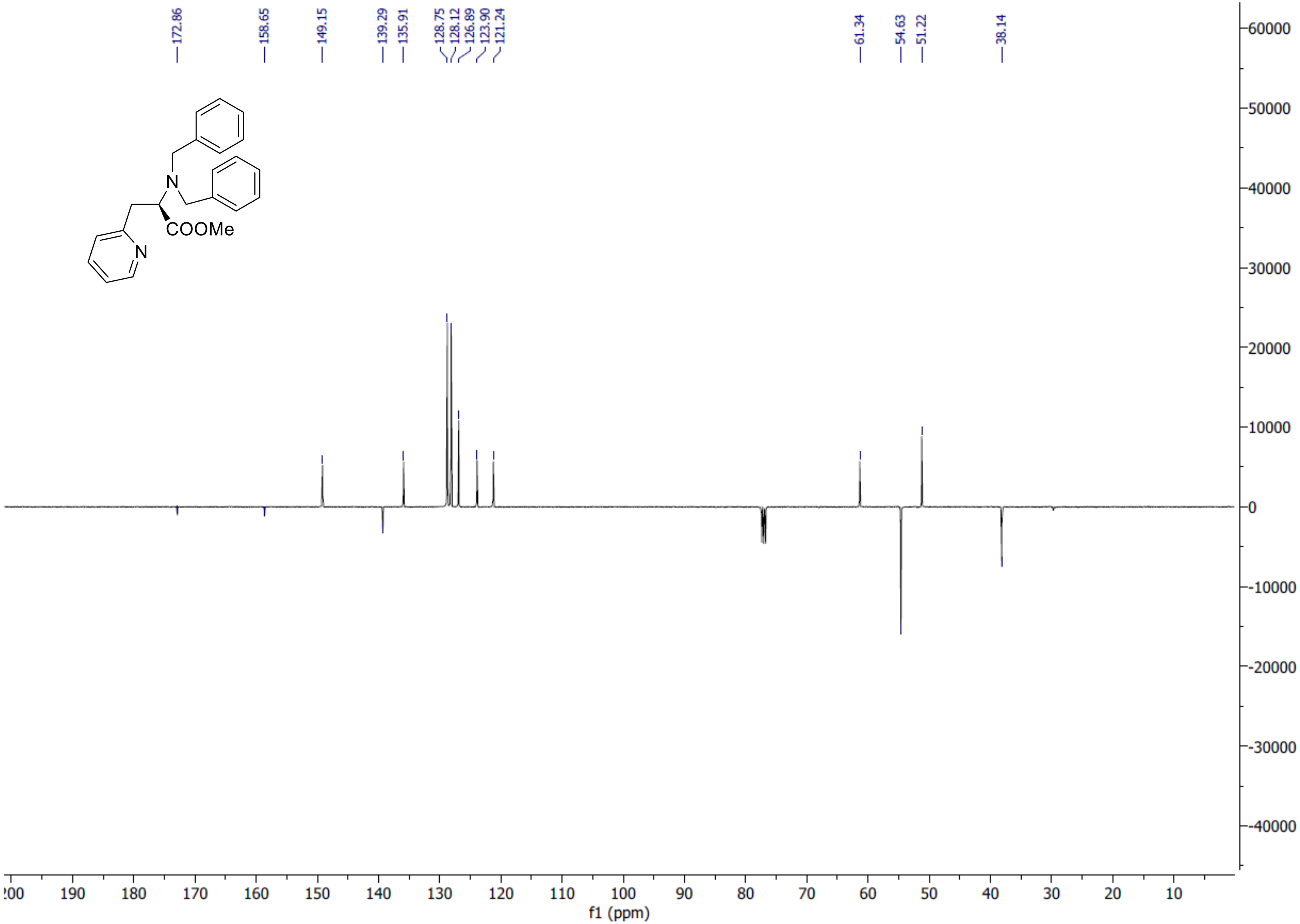

Figure S47: qDEPT spectrum of compound **29**

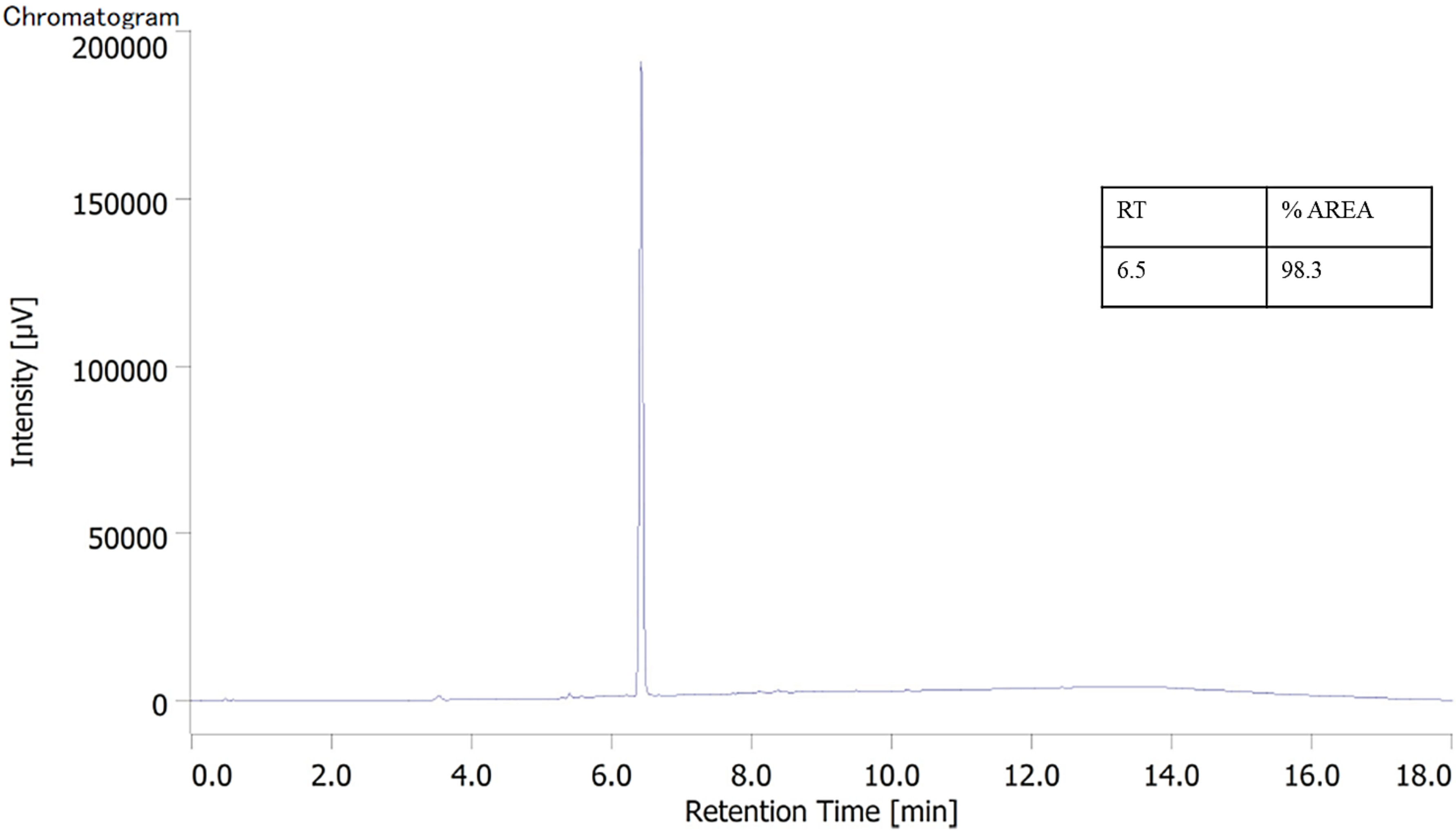

RT	% AREA
6.5	98.3

Figure S48: HPLC trace of compound **29**

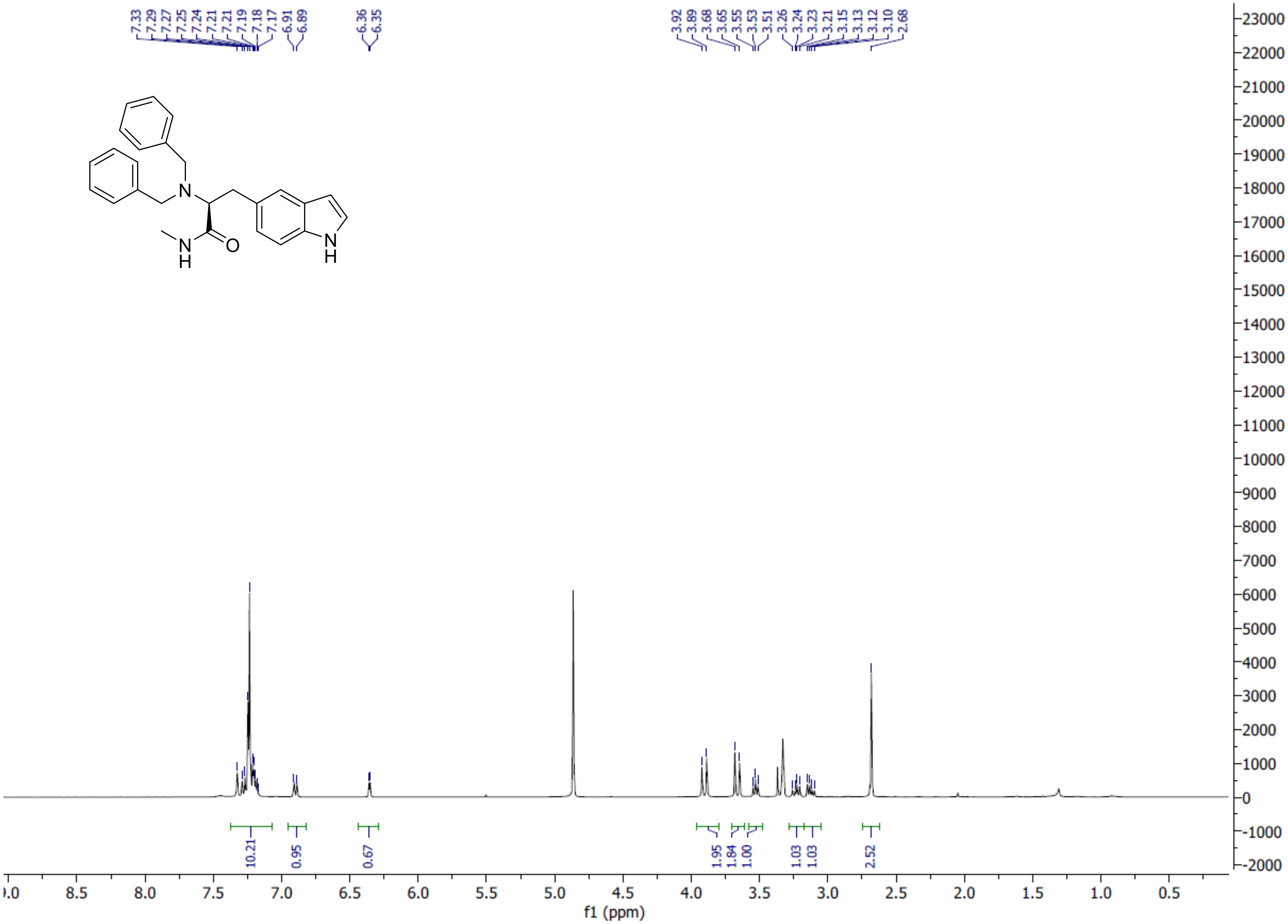

Figure S49: ^{1}H NMR spectrum of compound **30**

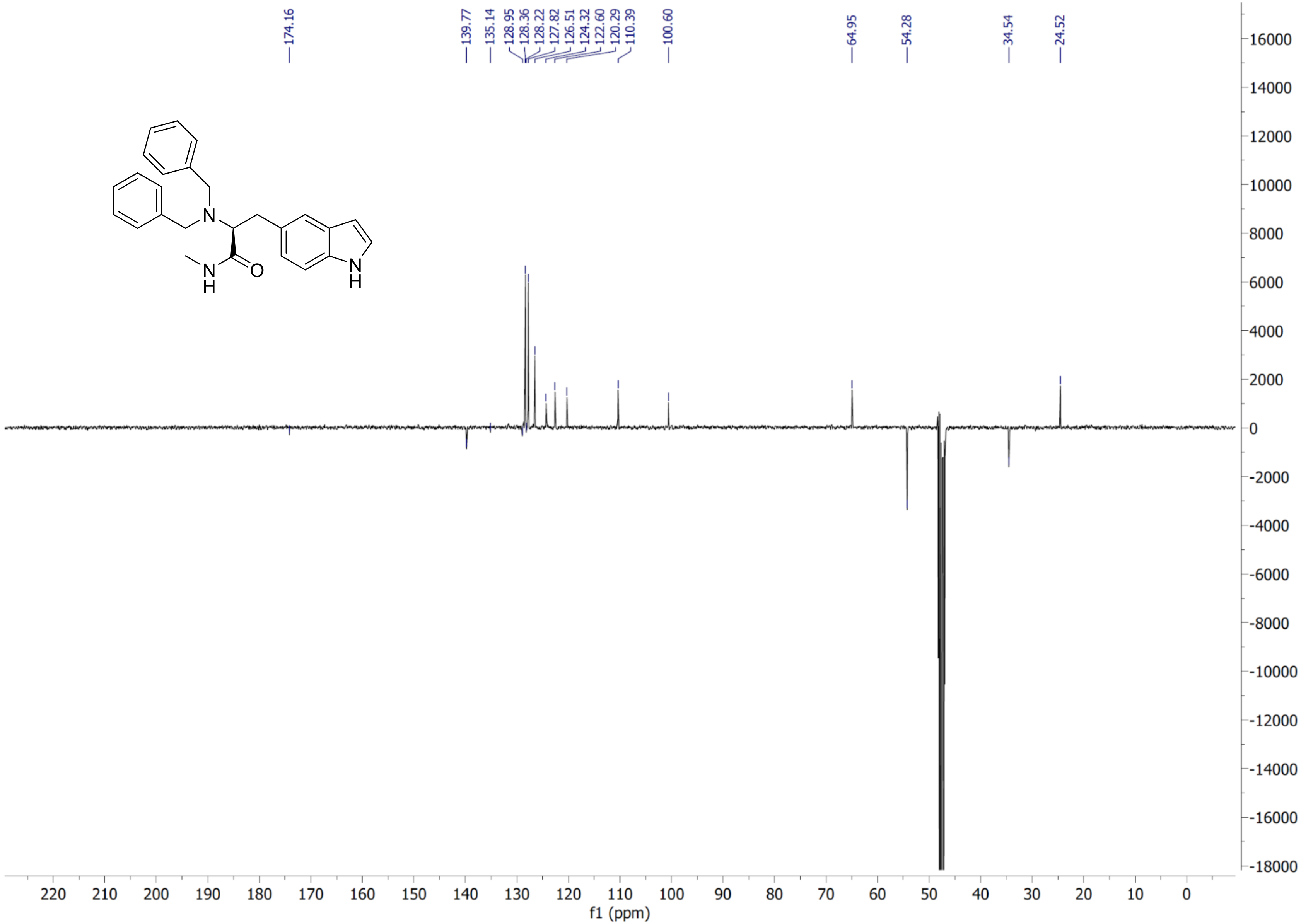

Figure S50: qDEPT spectrum of compound **30**

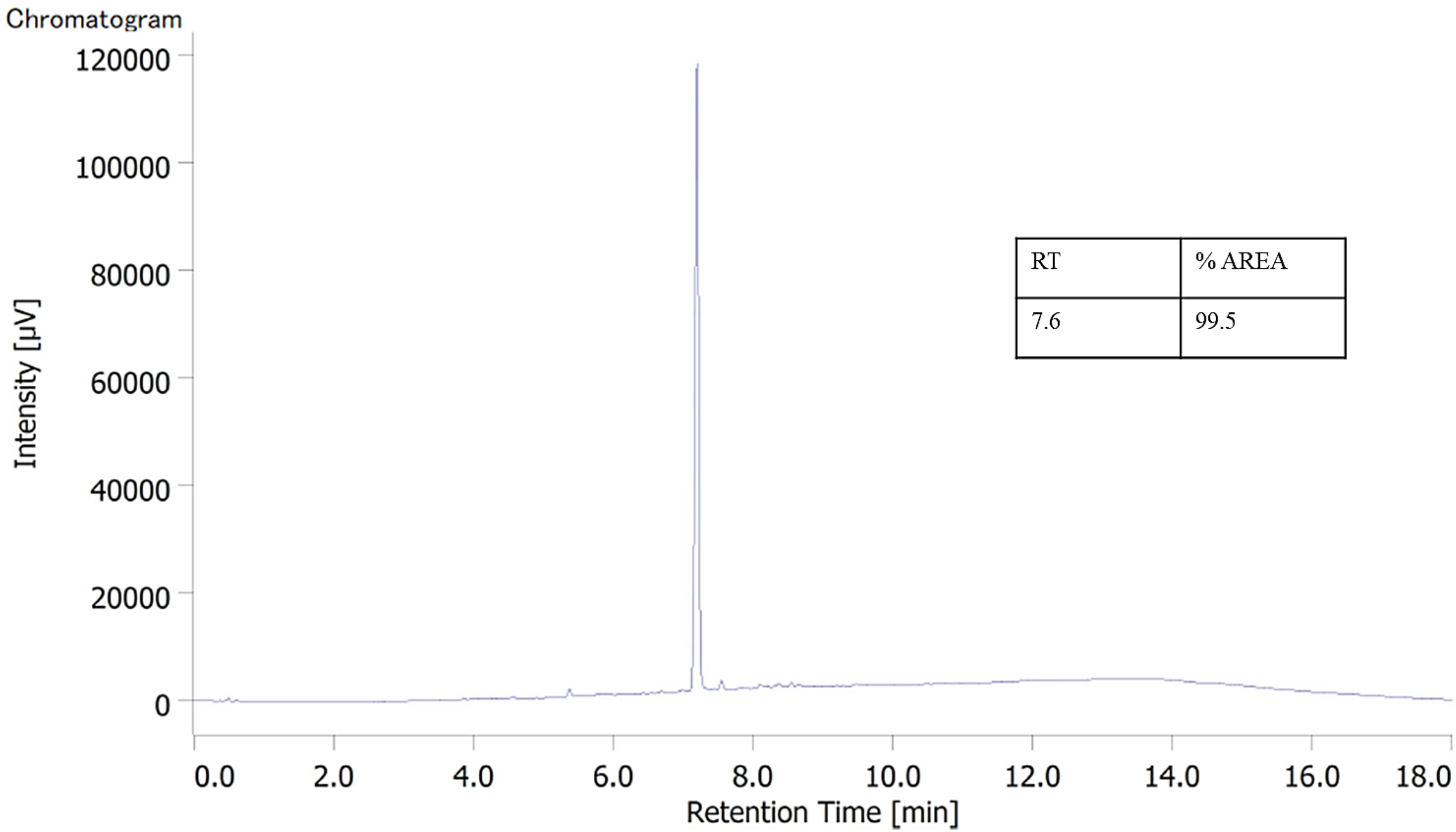

RT	% AREA
7.6	99.5

Figure S51: HPLC trace of compound **30**

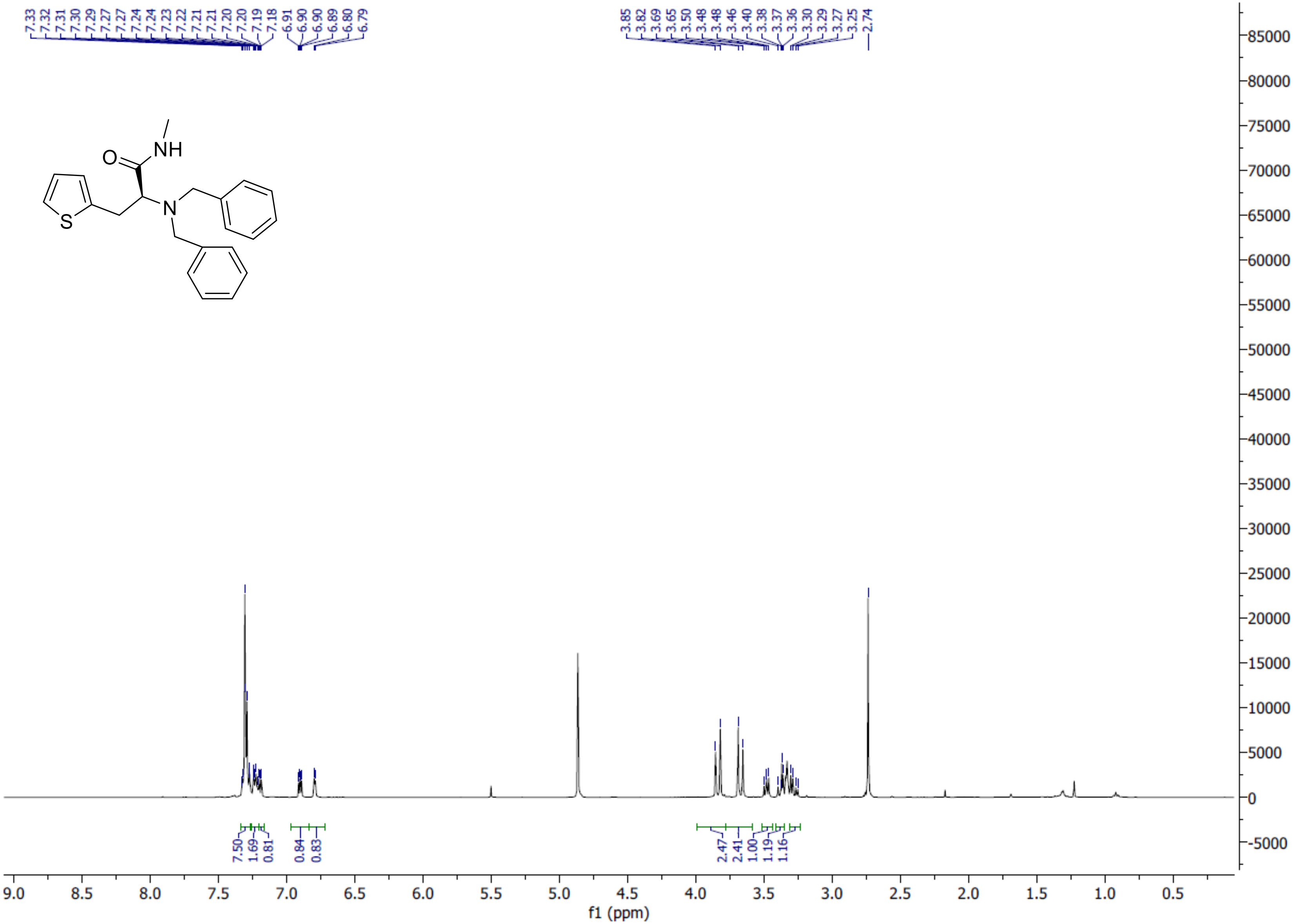

Figure S52: ^{1}H NMR spectrum of compound **31**

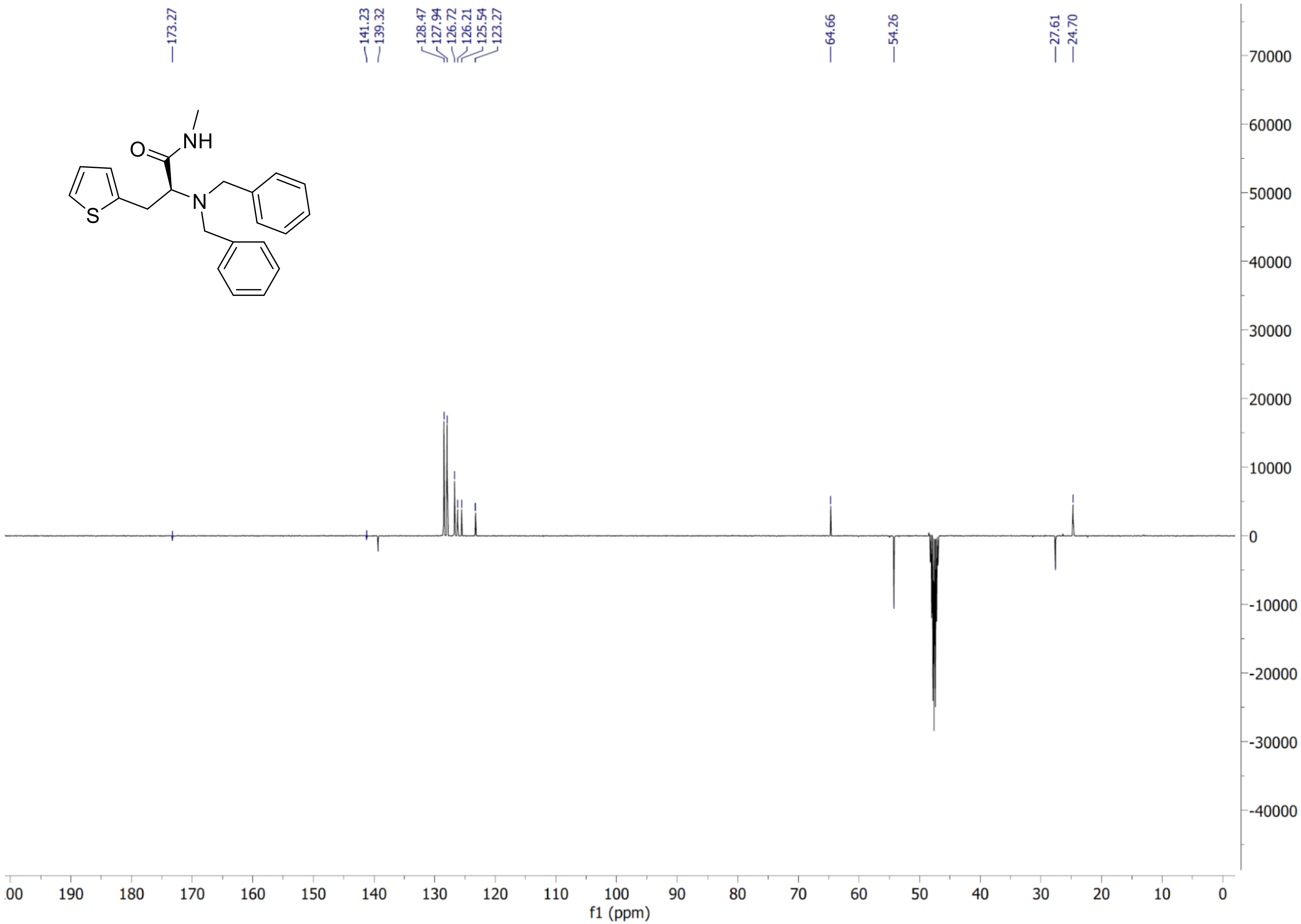

Figure S53: qDEPT spectrum of compound **31**

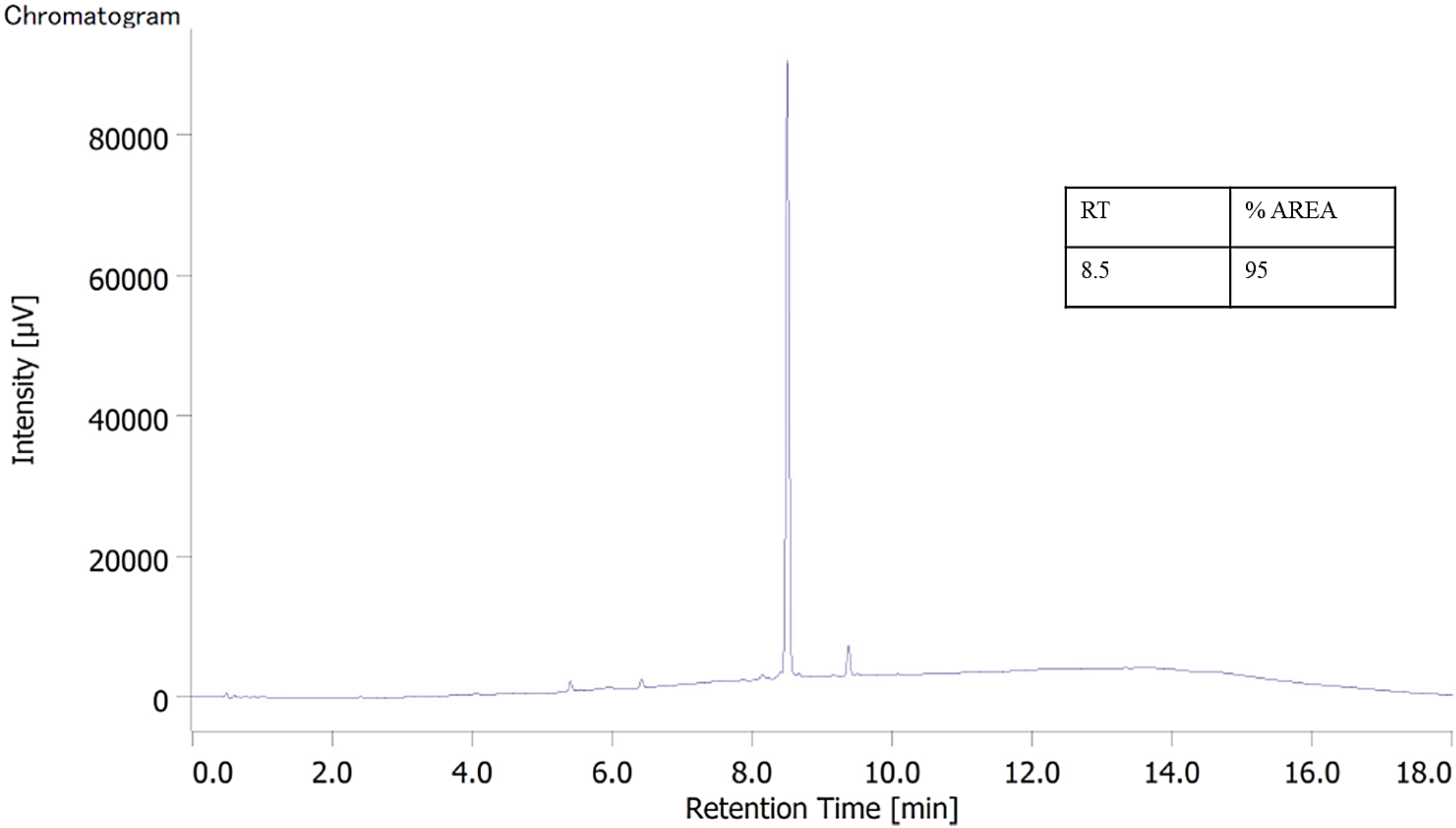

RT	% AREA
8.5	95

Figure S54: HPLC trace of compound **31**

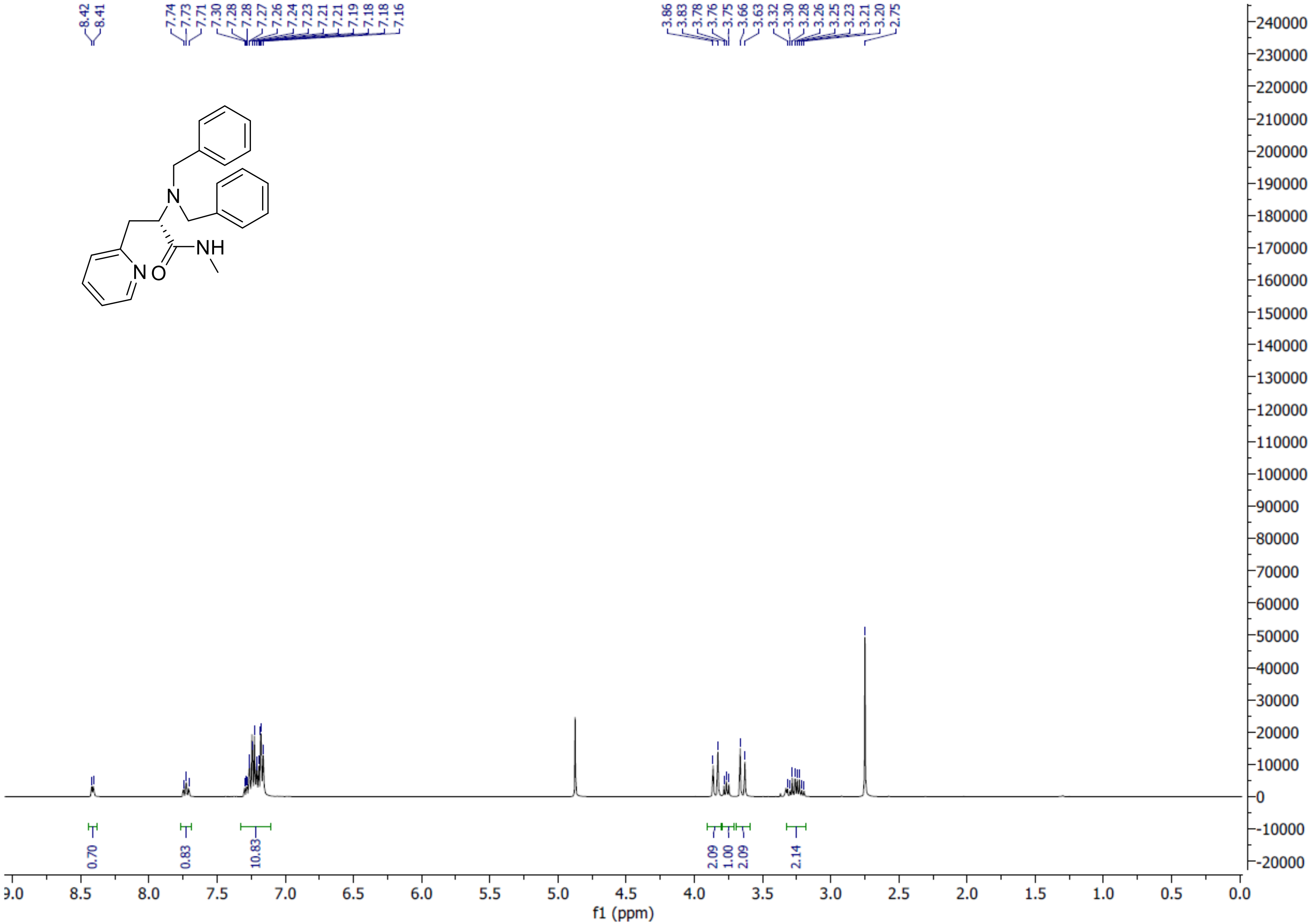

Figure S55: ^{1}H NMR spectrum of compound **32**

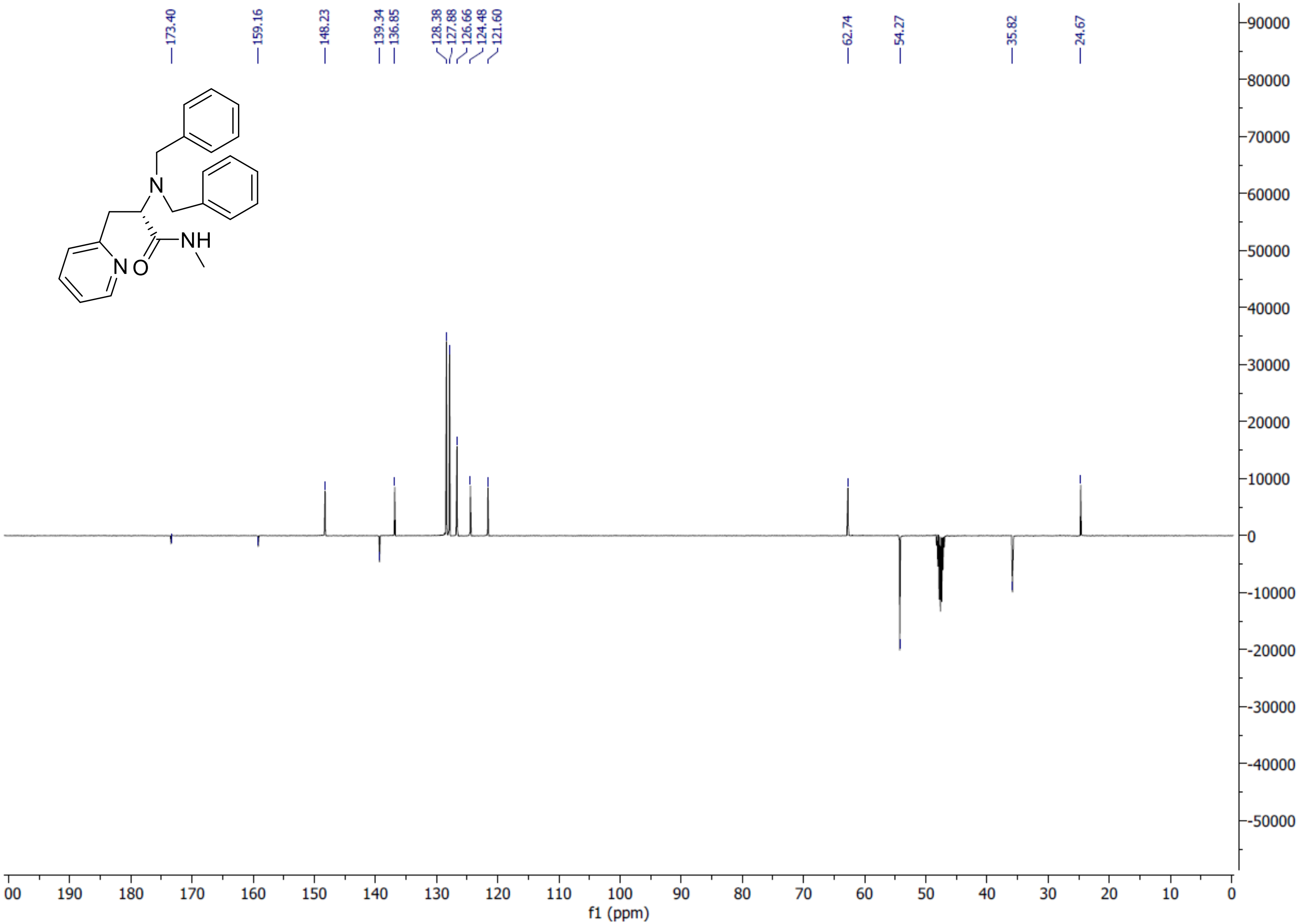

Figure S56: qDEPT spectrum of compound **32**

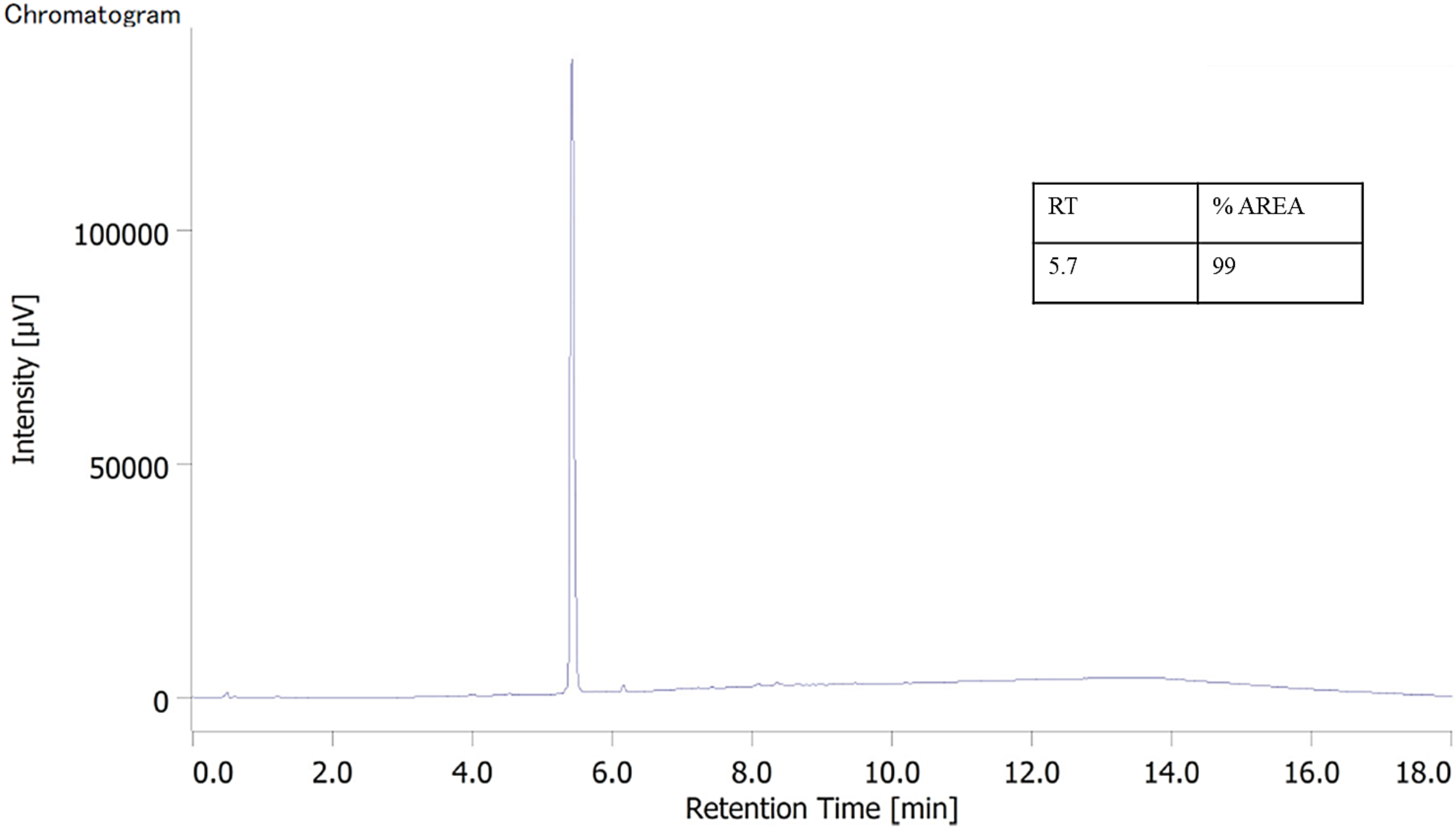

RT	% AREA
5.7	99

Figure S57: HPLC trace of compound **32**

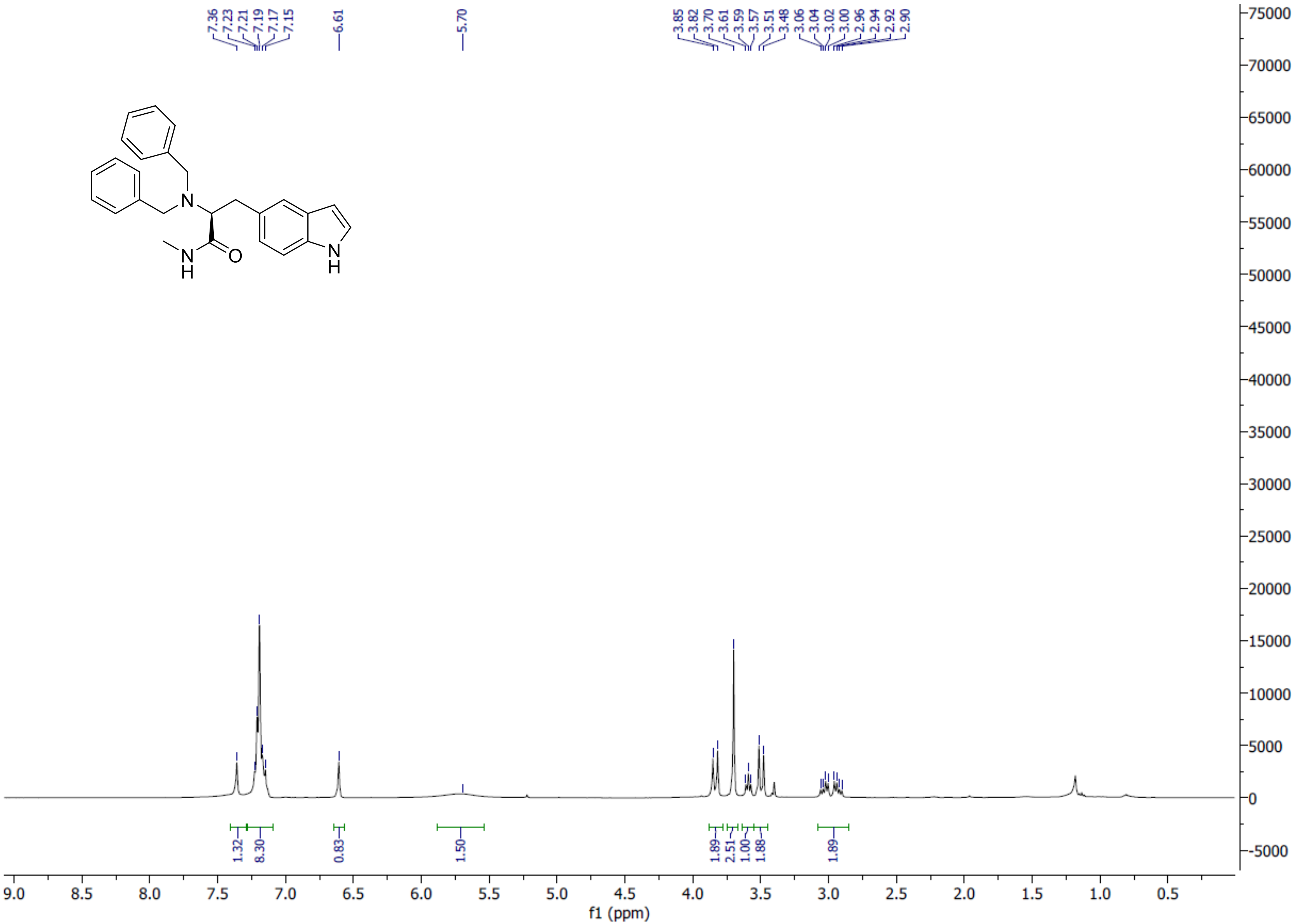

Figure S58: ^{1}H NMR spectrum of compound **34**

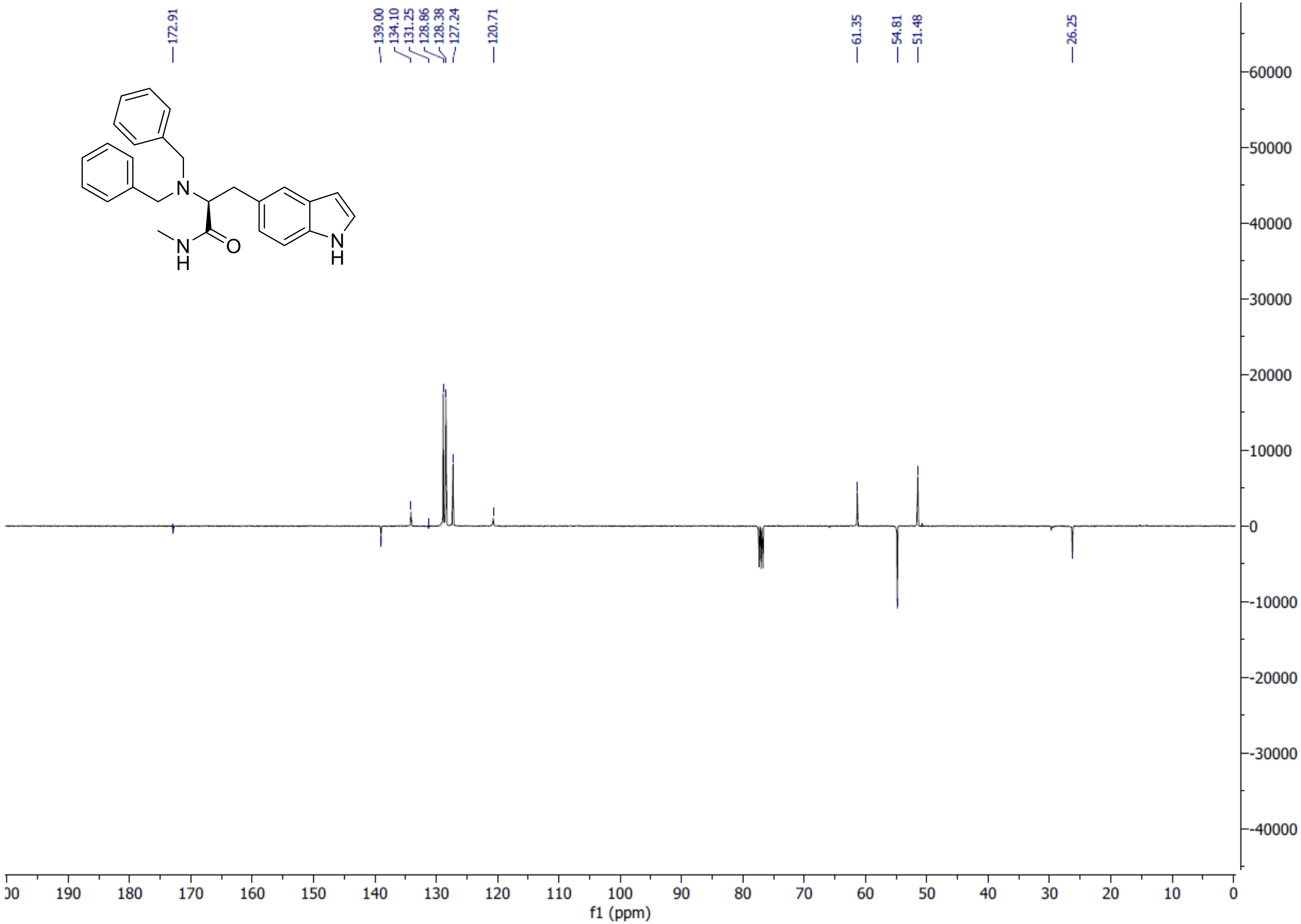

Figure S59: qDEPT spectrum of compound **34**

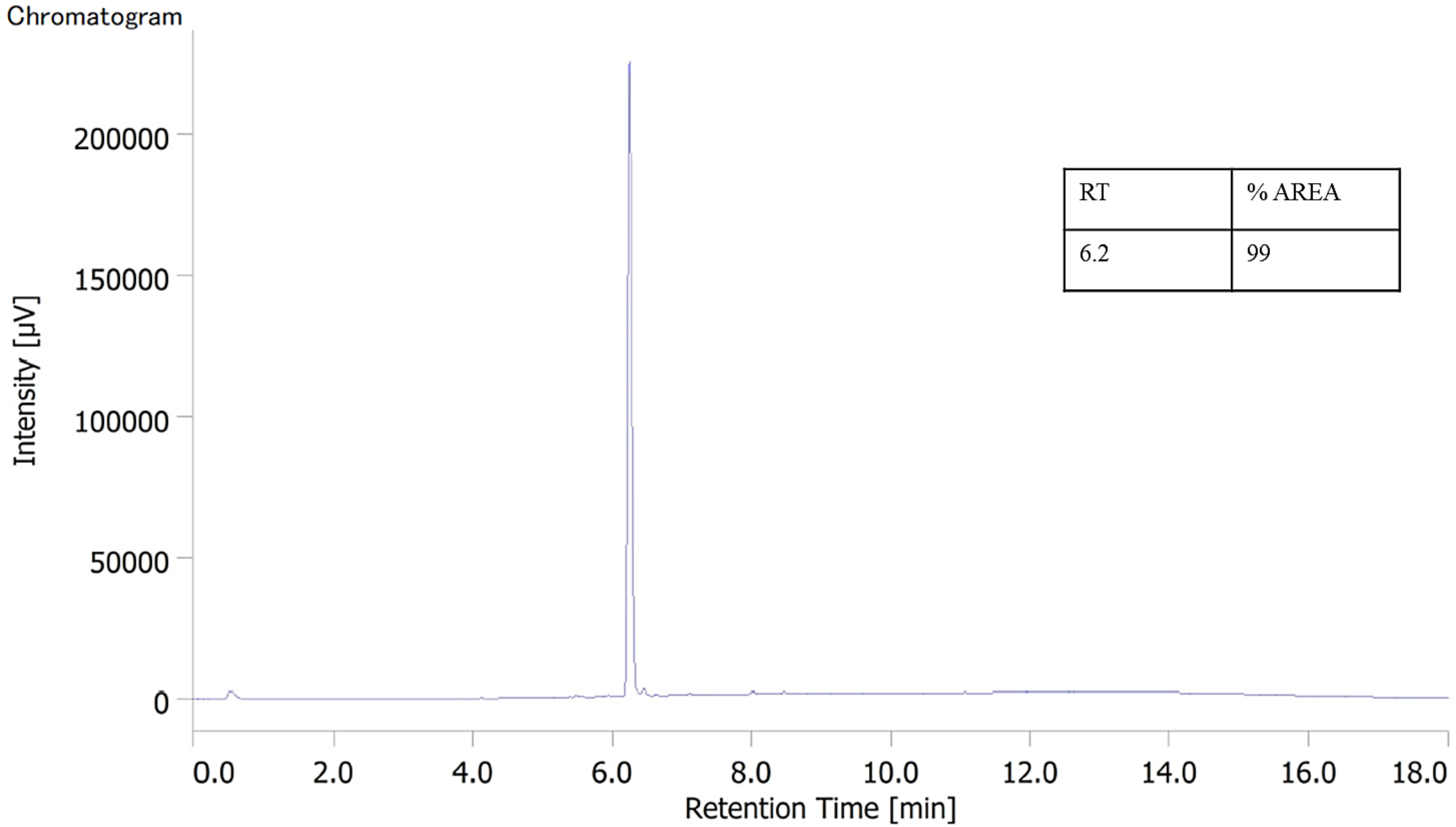

Figure S60: HPLC trace of compound **34**

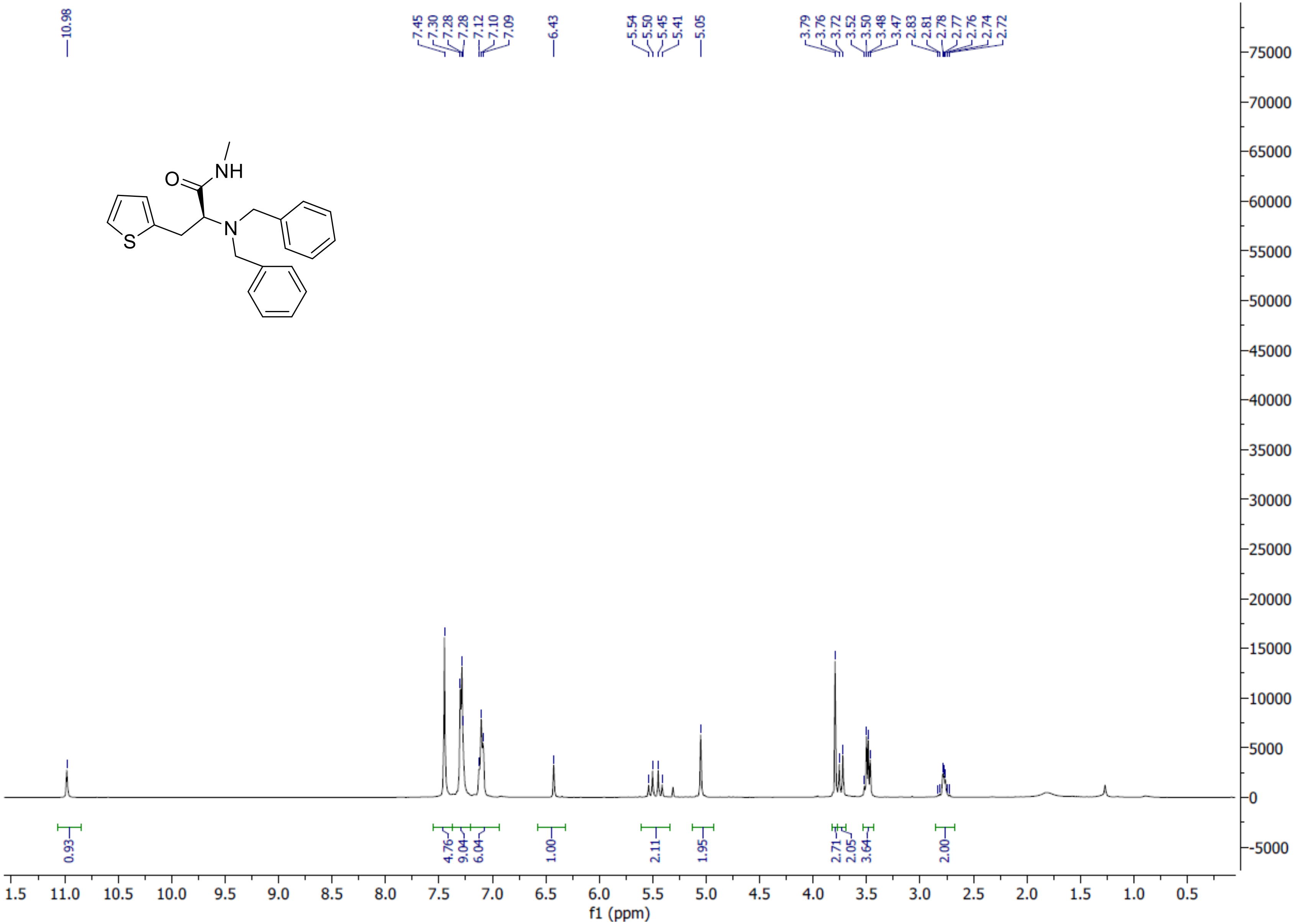

Figure S61: ^{1}H NMR spectrum of compound **35**

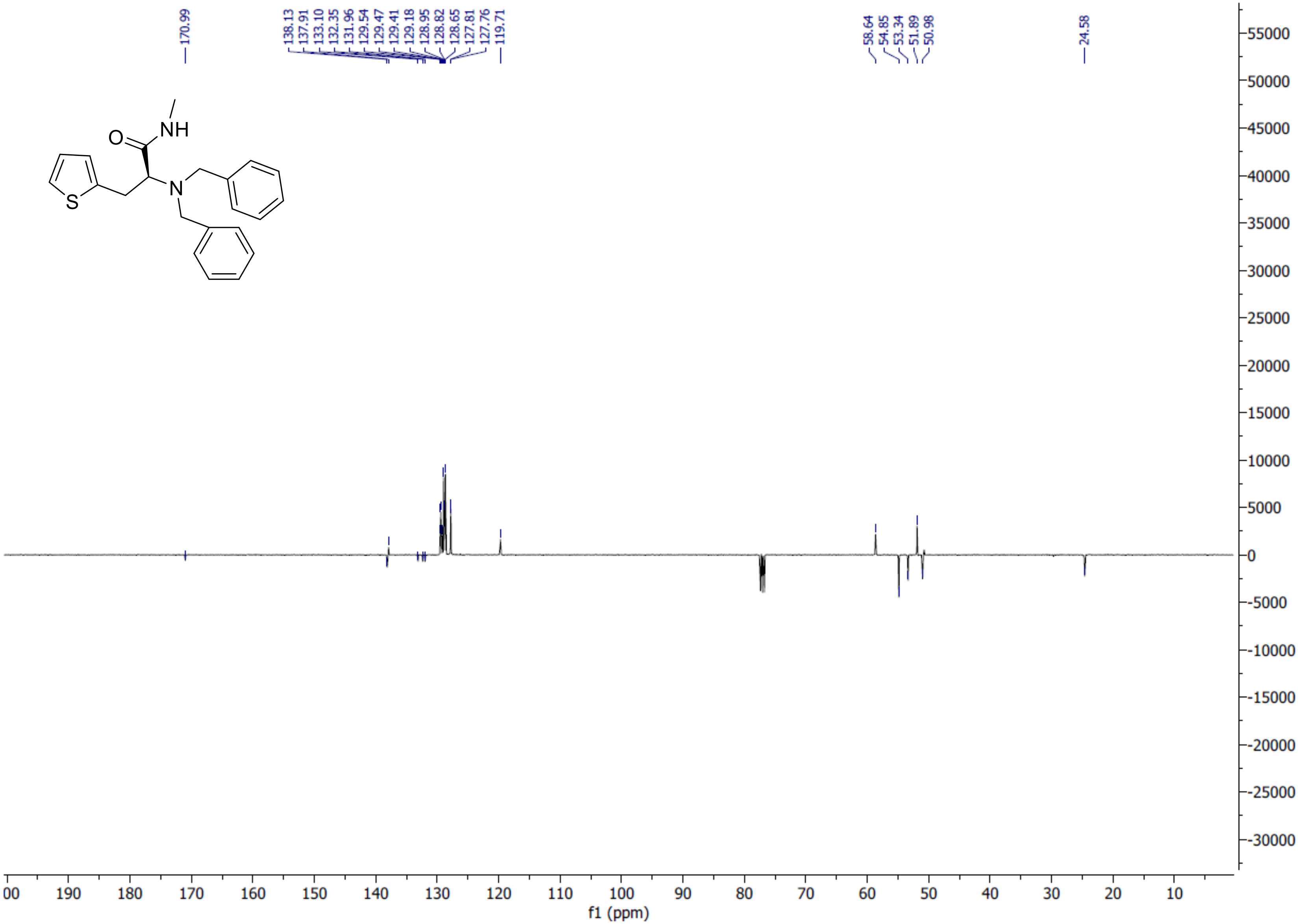

Figure S62: qDEPT spectrum of compound **35**

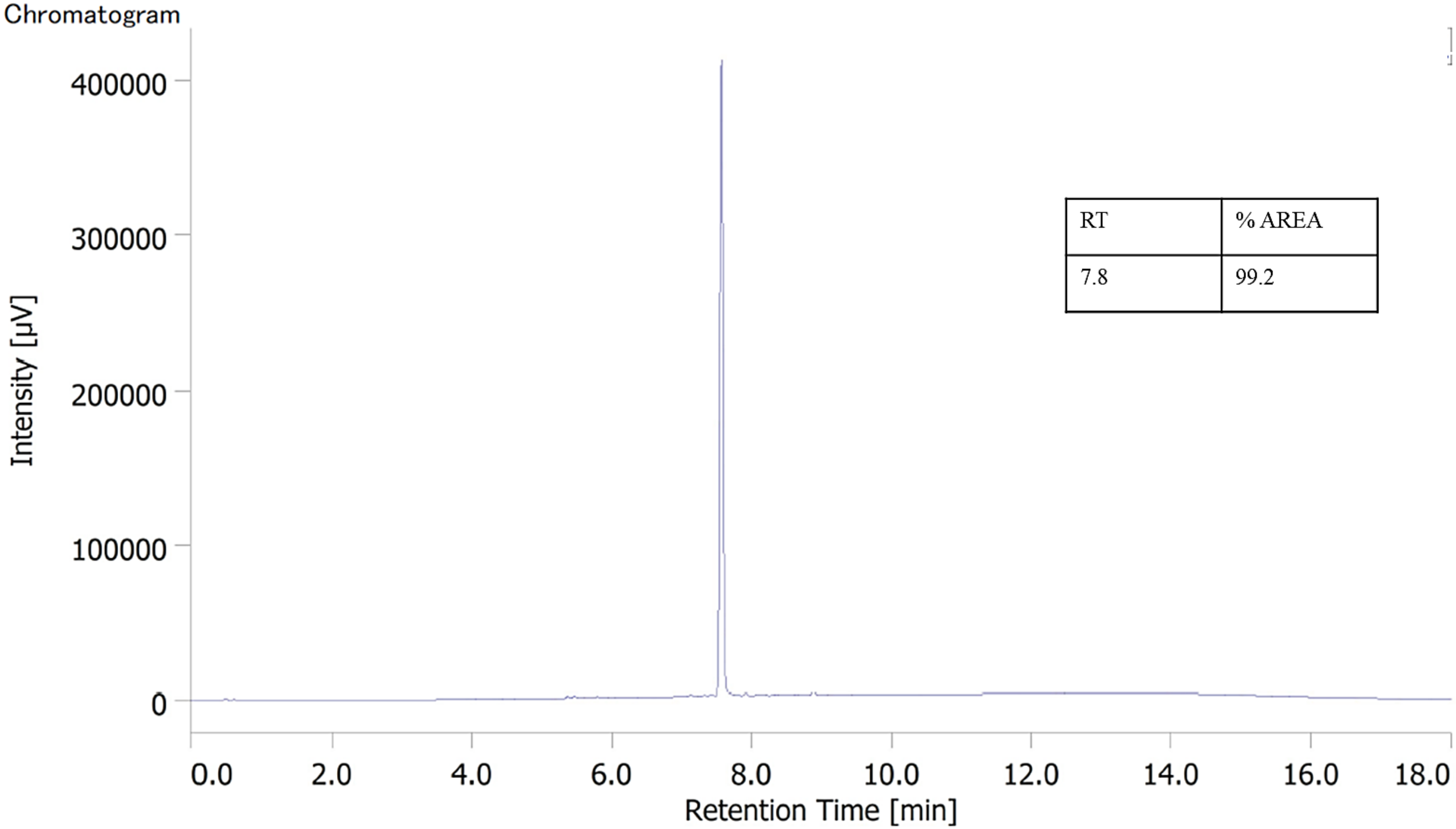

RT	% AREA
7.8	99.2

Figure S63: HPLC trace of compound **35**

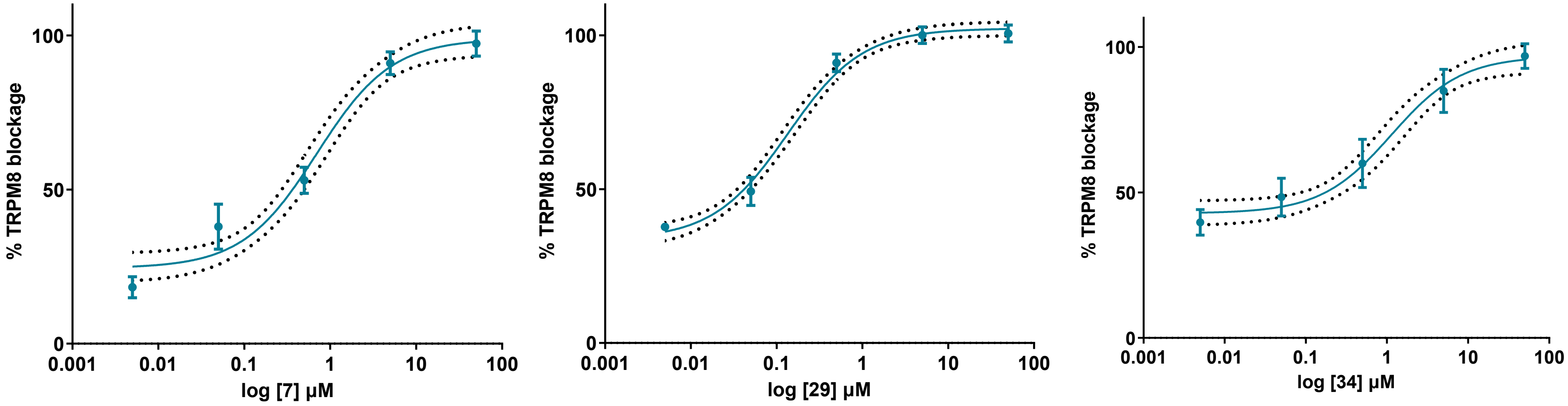

IC$_{50}$ ≈ 0.69 µM
R^2 = 0.95
95% CI for IC$_{50}$ of 0.46–1,05 µM

IC$_{50}$ ≈ 0.15 µM
R^2 = 0.98
95% CI for IC$_{50}$ of 0.10–0,17 µM

IC$_{50}$ ≈ 1,13 µM
R^2 = 0.92
95% CI for IC$_{50}$ of 0.64–2,04 µM

Figure S64: Ca^{2+} imaging concentration–response curves for compounds **7**, **29**, **34**

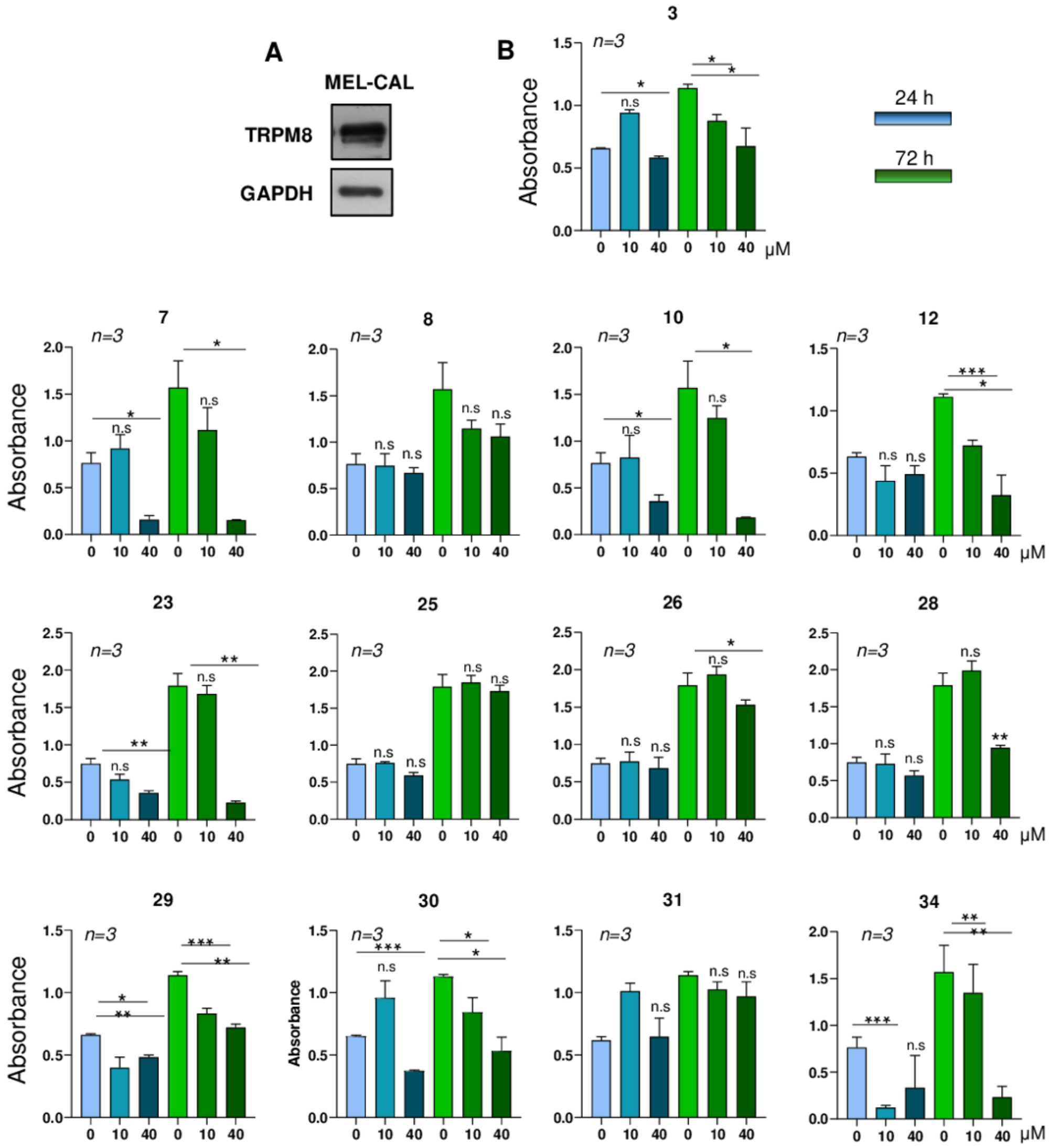

Fig. S65: Analysis of TRPM8 expression and effects of TRPM8 modulators on MEL-CAL cell viability

(A) Representative Western blot showing TRPM8 protein expression in MEL-CAL cells. GAPDH was used as a loading control.

(B) Viability of MEL-CAL cells untreated or treated with the different compounds at the concentrations indicated on the X-axis. Absorbance values from CCK-8 assays after 24 h and 72 h are reported. Data are expressed as mean ± standard deviation (SD) from n = 3 independent experiment.

*p < 0.05, **p < 0.01, ***p < 0.001 for the indicated time points vs. the corresponding untreated control

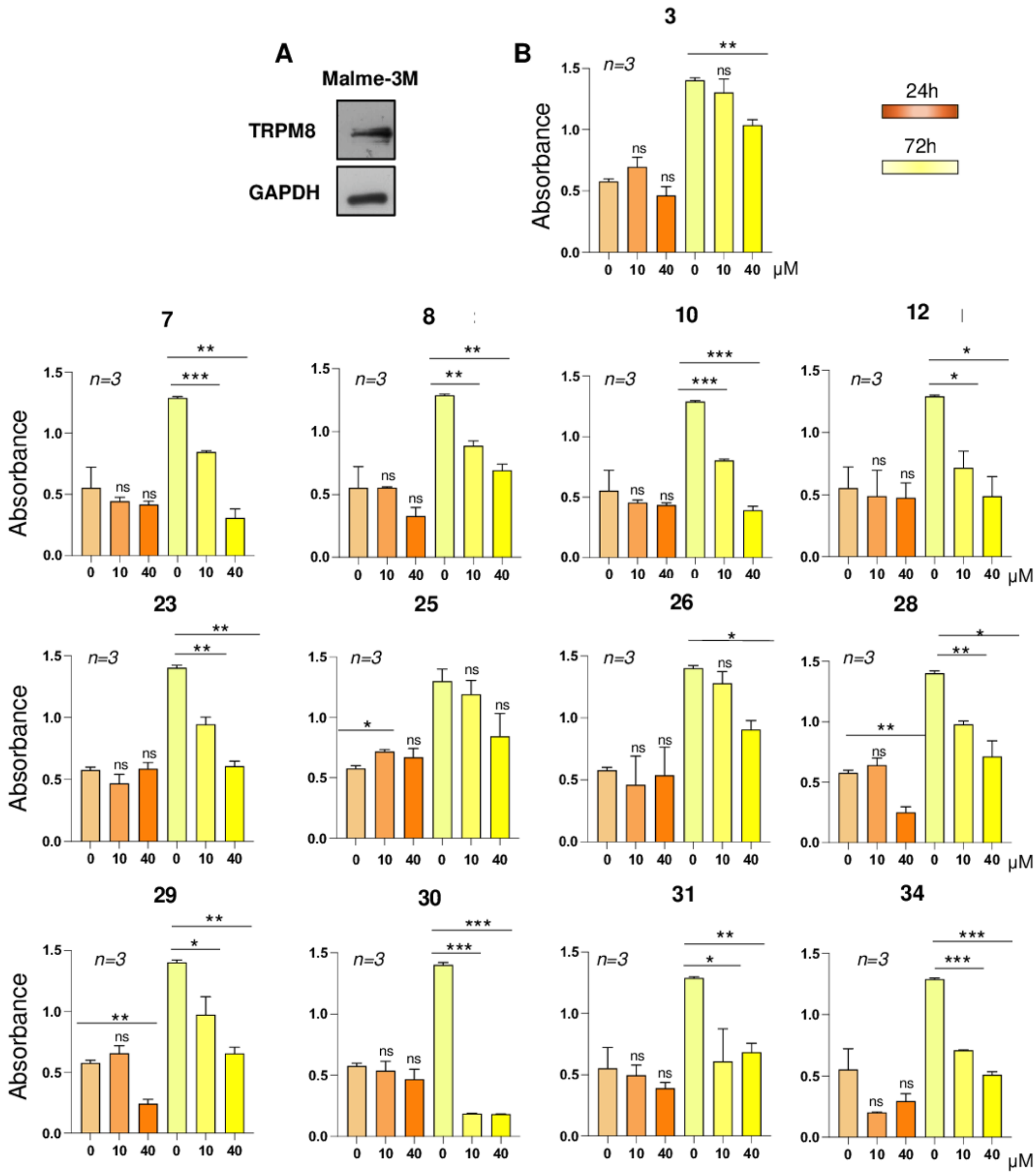

Fig. S66: Analysis of TRPM8 expression and effects of TRPM8 modulators on Malme-3M cell viability

(A) Representative Western blot showing TRPM8 protein expression in Malme-3M cells. GAPDH was used as a loading control.

(B) Viability of Malme-3M cells untreated or treated with the different compounds at the concentrations indicated on the X-axis. Absorbance values from CCK-8 assays after 24 h and 72 h are reported. Data are expressed as mean ± standard deviation (SD) from n = 3 independent experiments.

*$p < 0.05$, **$p < 0.01$, ***$p < 0.001$ for the indicated time points vs. the corresponding untreated control.

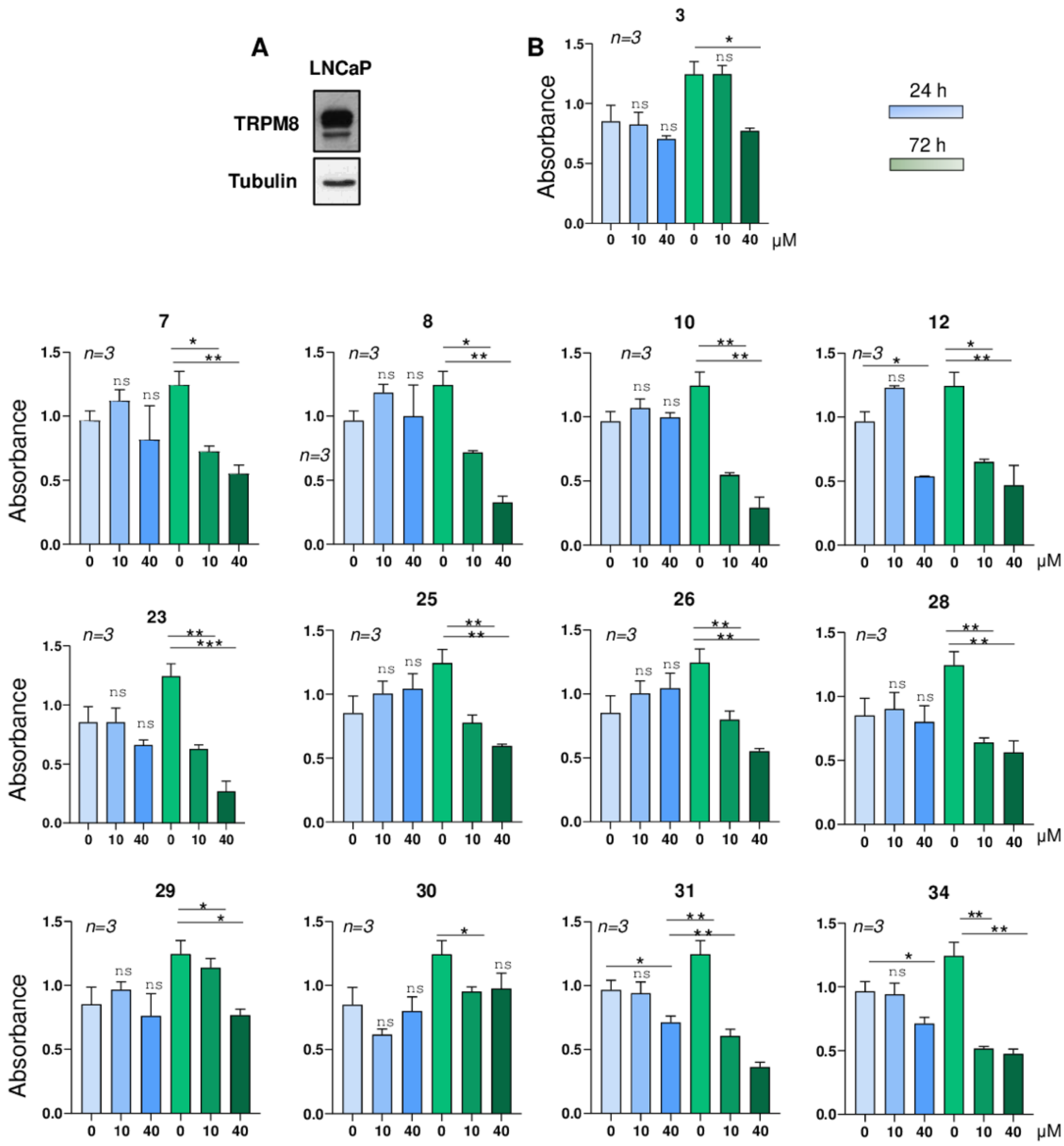

Fig. S67: Analysis of TRPM8 expression and effects of TRPM8 modulators on LNCaP cell viability

(A) Representative Western blot showing TRPM8 protein expression in LNCaP cells. Tubulin was used as a loading control.

(B) Viability of LNCaP cells untreated or treated with the different compounds at the concentrations indicated on the X-axis. Absorbance values from CCK-8 assays after 24 h and 72 h are reported. Data are expressed as mean ± standard deviation (SD) from n = 3 independent experiments.

*$p < 0.05$, ** $p < 0.01$, *** $p < 0.001$ for the indicated time points vs. the corresponding untreated control.

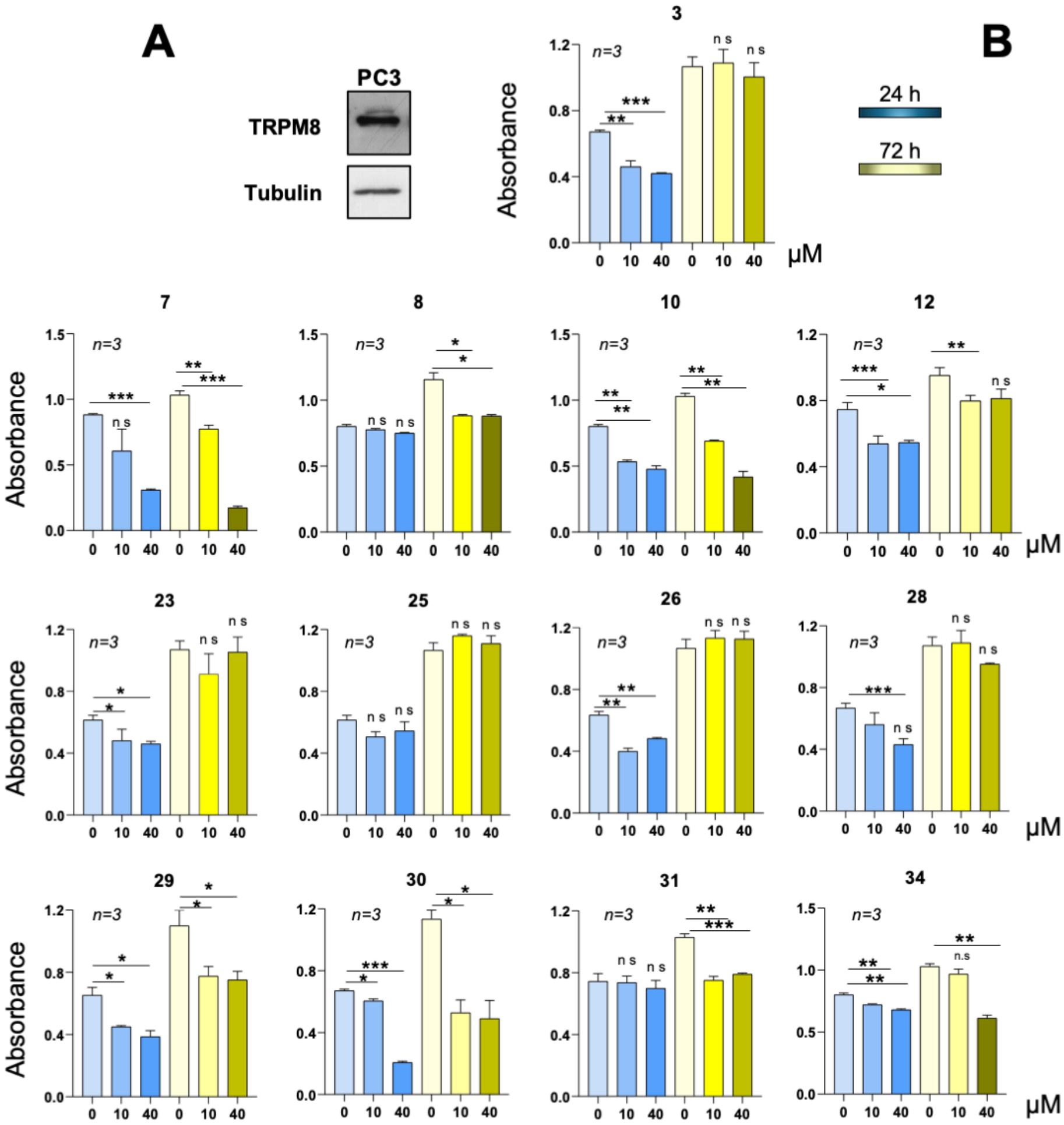

Fig. S68: Analysis of TRPM8 expression and effects of TRPM8 modulators on PC3 cell viability

(A) Representative Western blot showing TRPM8 protein expression in PC3 cells. Tubulin was used as a loading control.

(B) Viability of PC3 cells untreated or treated with the different compounds at the concentrations indicated on the X-axis. Absorbance values from CCK-8 assays after 24 h and 72 h are reported. Data are expressed as mean ± standard deviation (SD) from n = 3 independent experiments.

*$p < 0.05$, ** $p < 0.01$, *** $p < 0.001$ for the indicated time points vs. the corresponding untreated control.

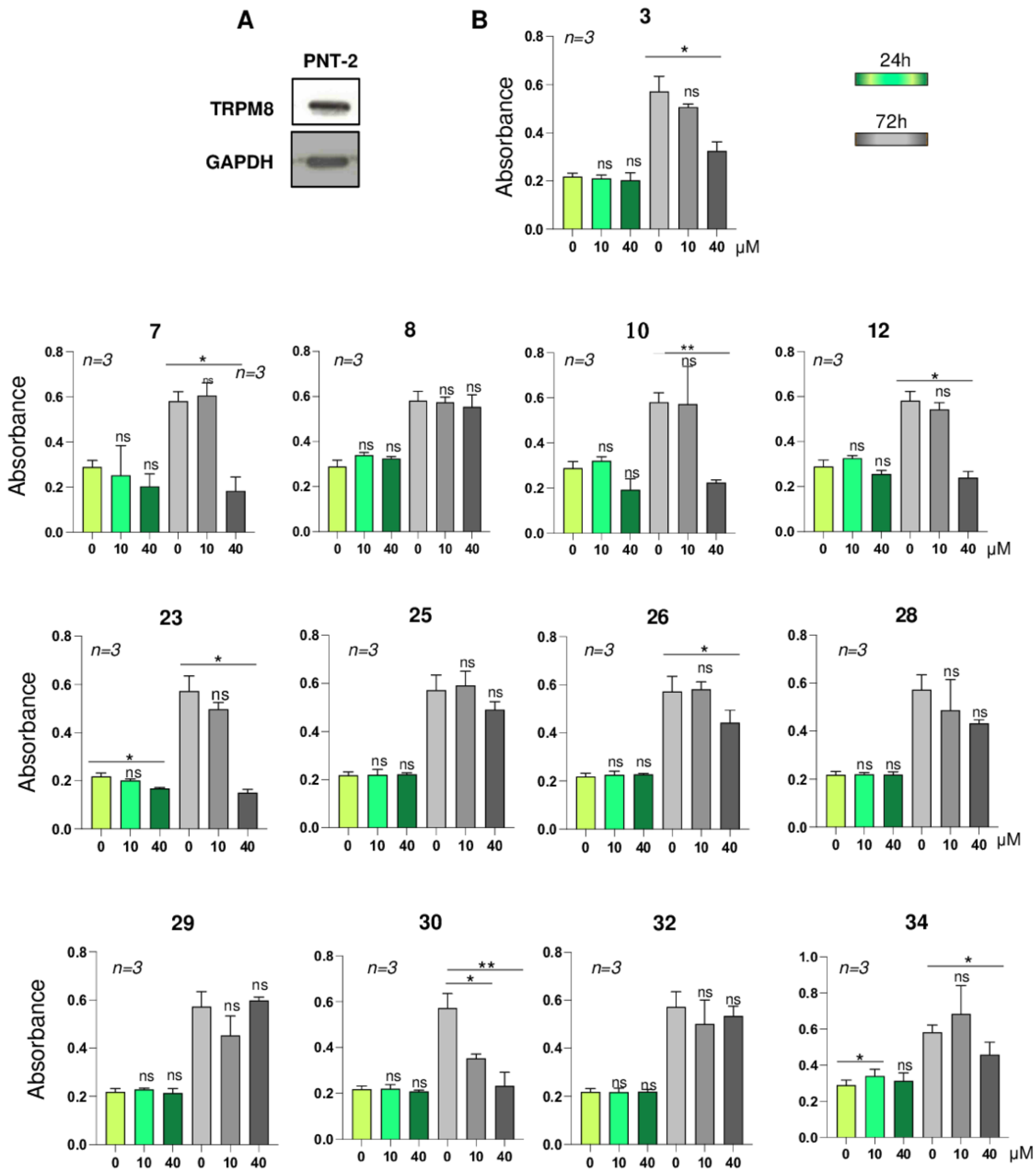

Fig. S69: Analysis of TRPM8 expression and effects of TRPM8 modulators on PNT-2 cell viability

(A) Representative Western blot showing TRPM8 protein expression in PNT-2 cells. GAPDH was used as a loading control.

(B) Viability of PNT-2 cells untreated or treated with the different compounds at the concentrations indicated on the X-axis. Absorbance values from CCK-8 assays after 24 h and 72 h are reported. Data are expressed as mean ± standard deviation (SD) from n = 3 independent experiments.

*p < 0.05, **p < 0.01, ***p < 0.001 for the indicated time points vs. the corresponding untreated control.

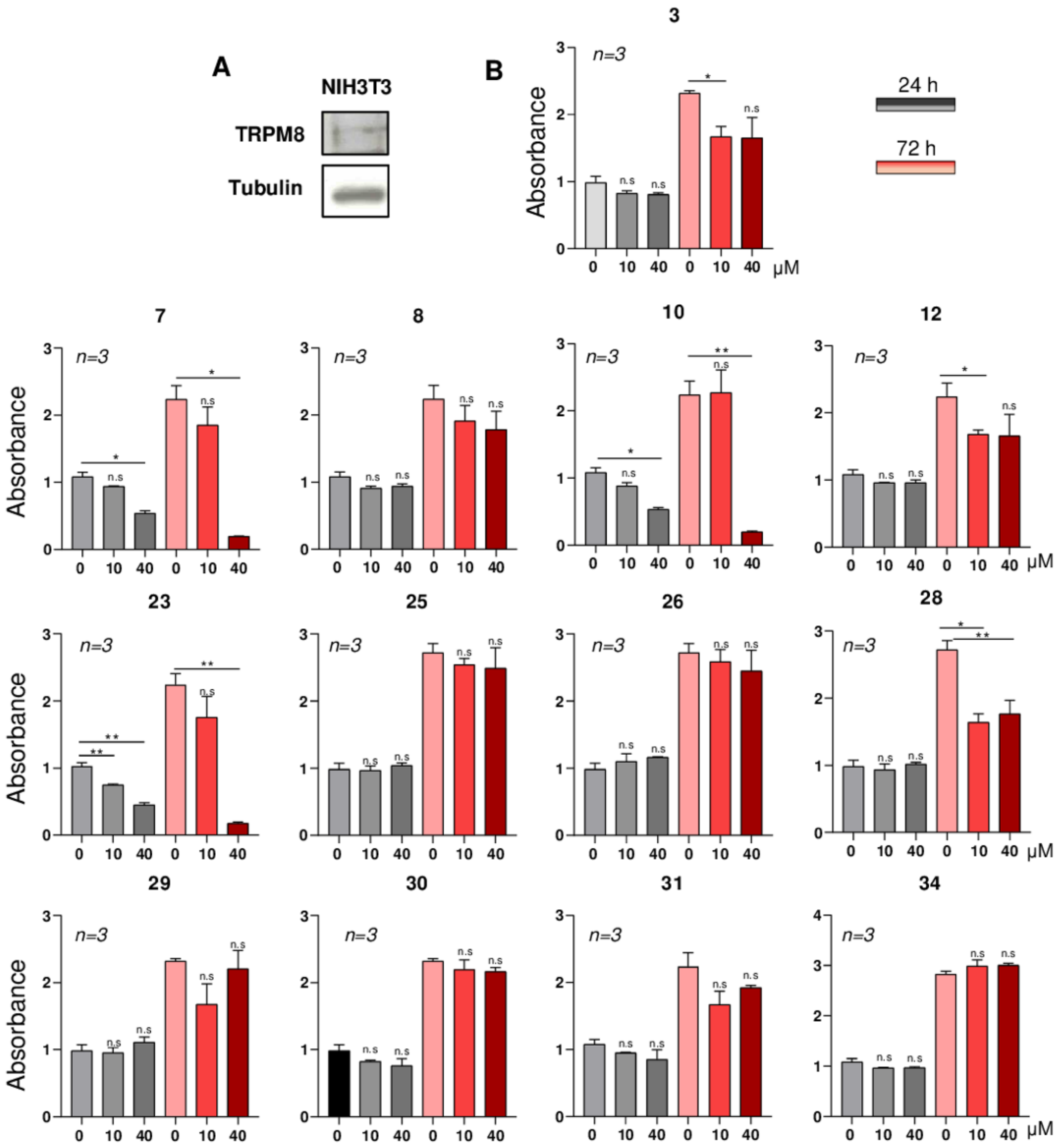

Fig. S70: Analysis of TRPM8 expression and effects of TRPM8 modulators on NIH3T3 cell viability

(A) Representative Western blot showing TRPM8 protein expression in NIH3T3 fibroblasts. Tubulin was used as a loading control.

(B) Viability of NIH3T3 cells untreated or treated with the different compounds at the concentrations indicated on the X-axis. Absorbance values from CCK-8 assays after 24 h and 72 h are reported. Data are expressed as mean ± standard deviation (SD) from n = 3 independent experiments.

$^{*}p < 0.05$, $^{**}p < 0.01$, $^{***}p < 0.001$ for the indicated time points vs. the corresponding untreated control.

Table S1: IC_{50} values (µM) of the previously reported TRPM8 antagonist (compound **14**) in melanoma, prostate cancer, and non-transformed cell lines after 72 h of treatment.

Compound	Time (h)	MEL-CAL	Malme-3M	LNCaP	PC3	PNT-2	NIH3T3
**14**	72	27.8±1.9	16.5±2.4	23.4±1.6	32.1±3.8	>40µM	24.6±2.3

Table S2: Concentrations of Compounds Used for Spheroid Treatments.

Cell line	Compound	Concentration (µM)
MEL-CAL	**3**	20
	**7**	20
	**12**	10
	**29**	10
	**34**	20
Malme-3M	**7**	10
	**8**	10
	**10**	10
	**12**	10
	**29**	20
	**30**	5
	**34**	10
LNCaP	**7**	10
	**8**	10
	**10**	5
	**12**	10
	**23**	10
	**25**	10
	**29**	10
	**31**	20
	**34**	10
PC3	**7**	10
	**8**	10
	**10**	10
	**29**	10
	**30**	10
	**34**	10

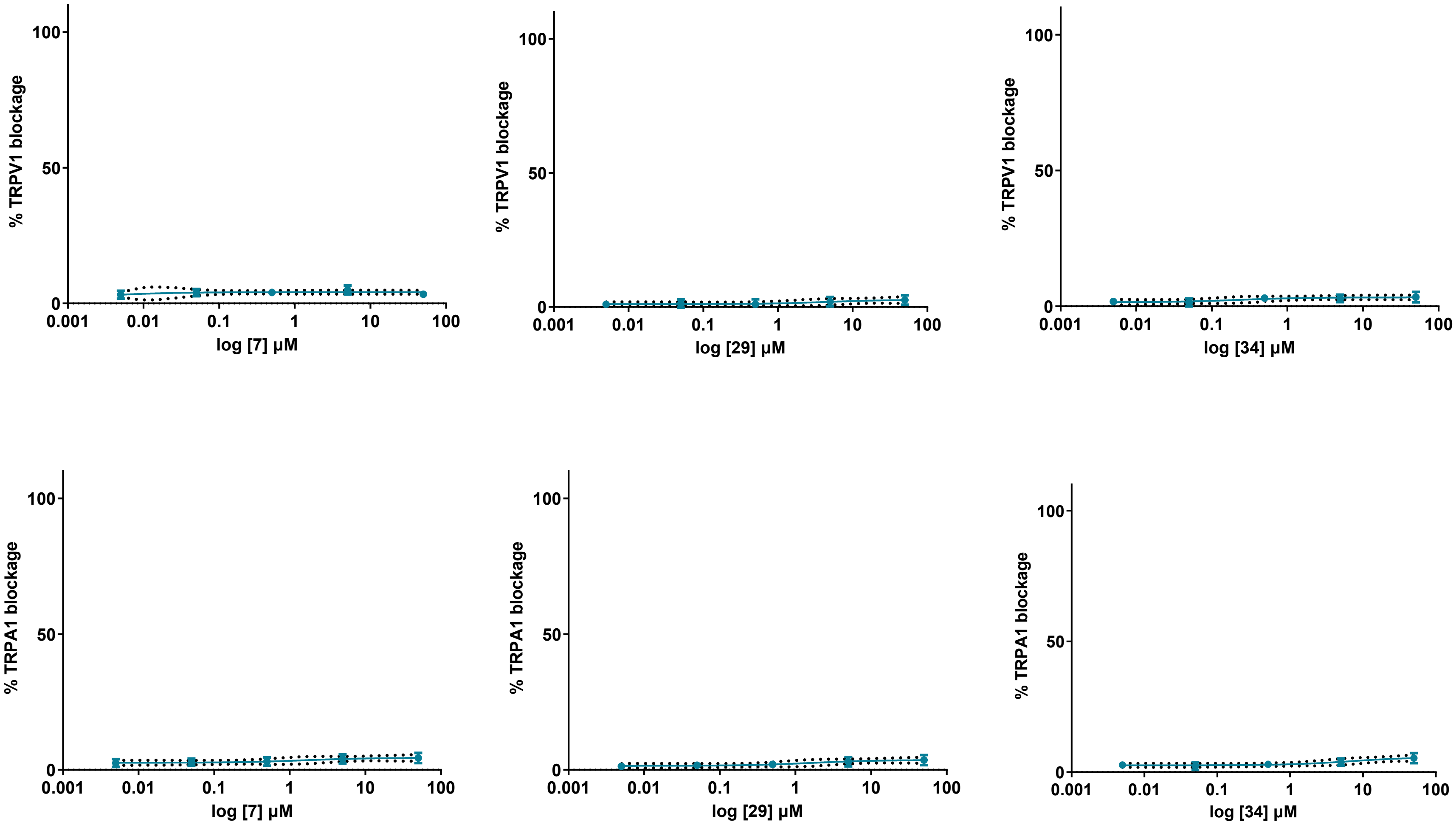

Figure S71: TRPA1 and TRPV1 selectivity assays of compounds **7**, **29** and **34** at concentrations ranging from 0.005 to 50 µM

Table S2: MRM parameters for all analytes tested.

Compound	Parent (m/z)	Daughter (m/z) qualifier	Collision (eV)	Daughter (m/z) quantifier	Collision (eV)
**7**	435.20	202.08	15	160.00	30
**29**	361.20	181.07	22	268.10	16
**34**	350.20	290.17	20	91.00	30
**Tolbutamide (IS)**	270.99	90.97	30	74.00	12

IS: internal standard; MRM: multiple reaction monitoring; Cone voltage: 30 V; Dwell time: 0.034 s.

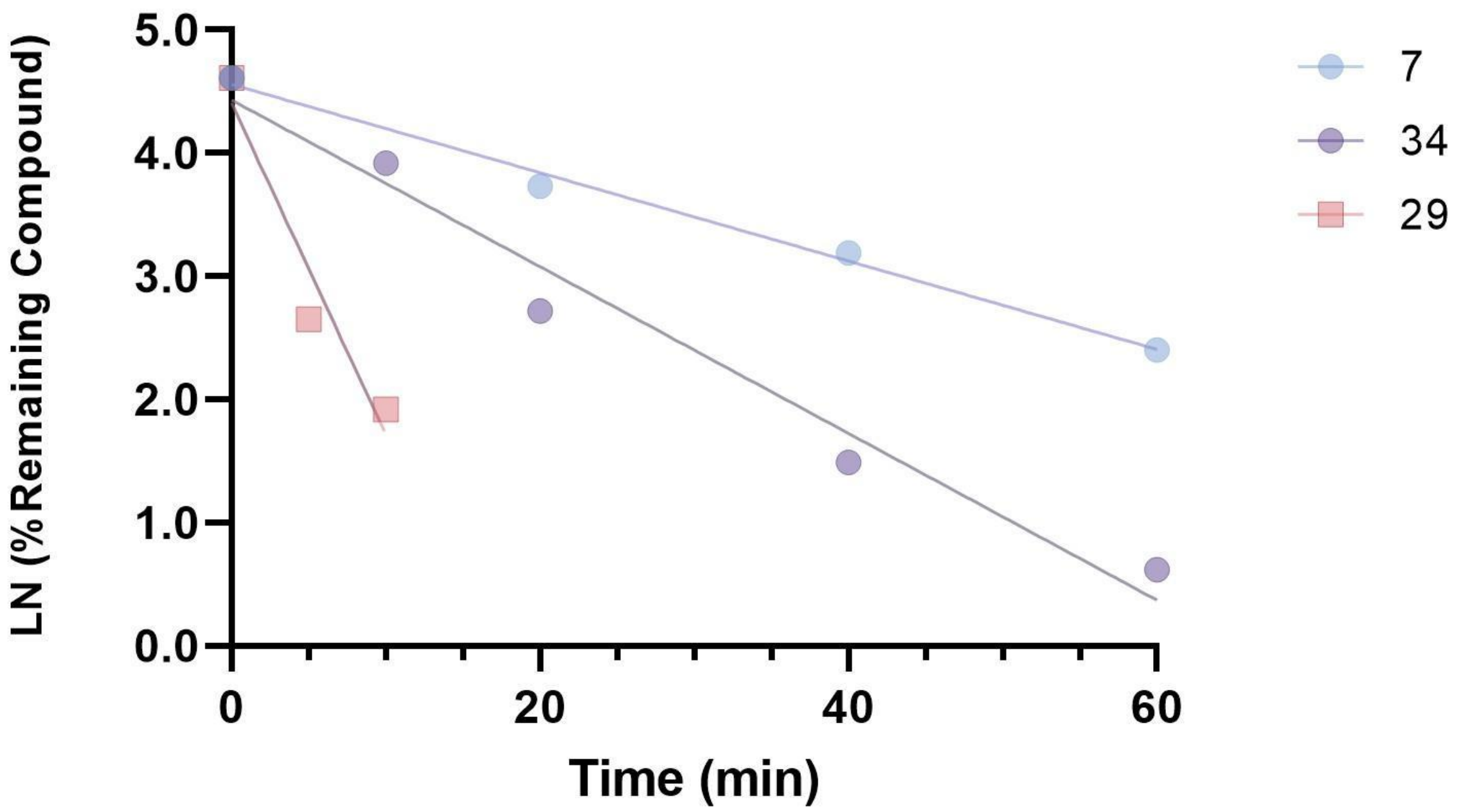

Figure S72: Liver microsomal stability assessment in human liver microsomes (HLMs). The metabolic stability of the test compounds (**7**: y = -0.0358x + 4.5535; R^2 = 0.9928, **29**: y = -0.0676x + 4.4282 R^2 = 0.9720; **34**: y = -0.2681x + 4.399; R^2 = 0.9338) was evaluated in HLMs at 37 °C in the presence of NADPH as a cofactor. Each compound (1 µM) was solubilized in an organic solvent, maintaining the final concentration below 1% (*v/v*) to prevent CYP inhibition and ensure accurate metabolic stability assessment. Low microsomal protein concentrations (0.5 mg mL^{-1}) were used to minimize nonspecific binding while preserving enzymatic activity. A "crash-in" incubation approach was applied: compounds were individually incubated with HLMs and quenched at predefined time points (20, 40, and 60 min) with ice-cold methanol containing tolbutamide as IS. For compound **29**, two additional time points (5 and 10 min) were included to obtain a more accurate estimation of in vitro pharmacokinetic parameters. All experiments were performed in triplicate. The extent of compound depletion over time was quantitatively determined by LC-MS/MS analysis. A 0-min control was prepared by immediate solvent addition, while testosterone and incubations without NADPH served as positive and negative controls, respectively.

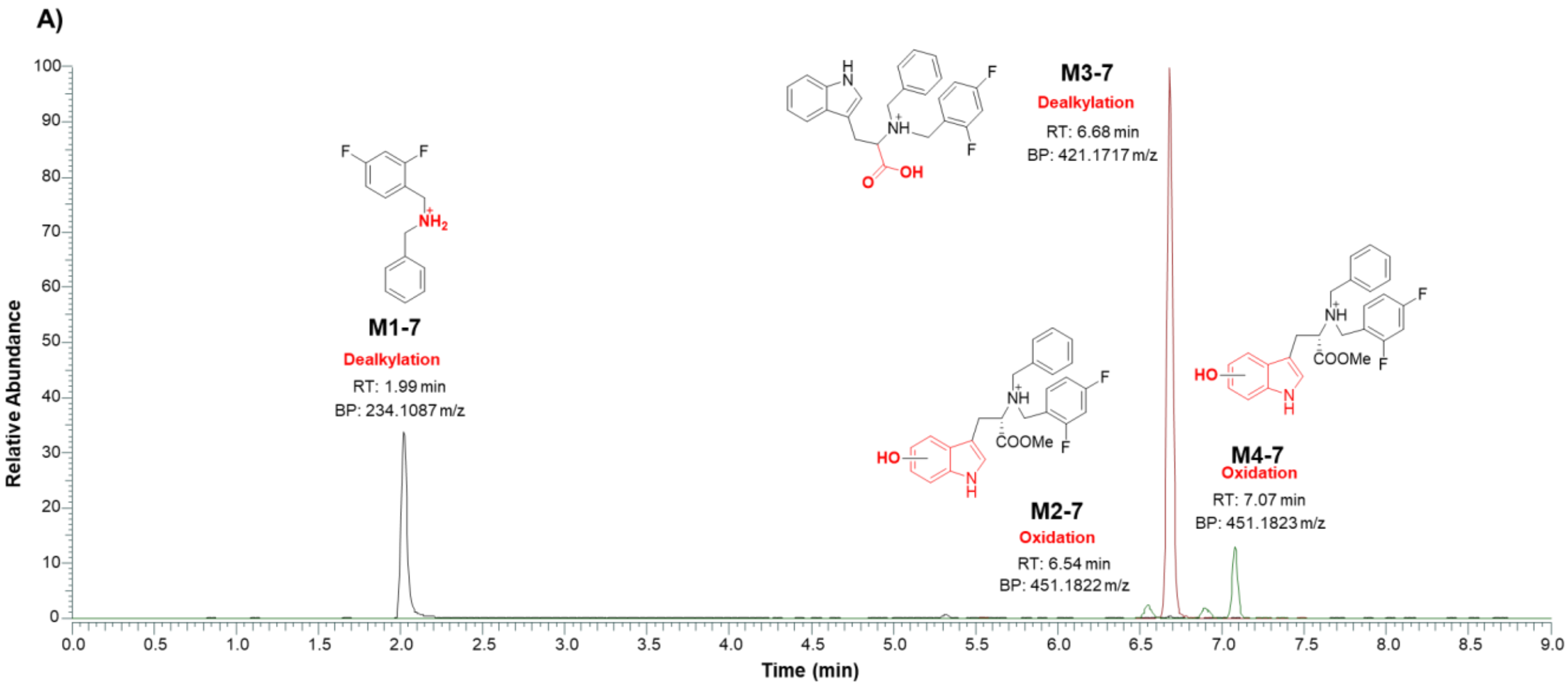
A)
M1-7
Dealkylation
RT: 1.99 min
BP: 234.1087 m/z
M2-7
Oxidation
RT: 6.54 min
BP: 451.1822 m/z
M3-7
Dealkylation
RT: 6.68 min
BP: 421.1717 m/z
M4-7
Oxidation
RT: 7.07 min
BP: 451.1823 m/z
Relative Abundance
Time (min)

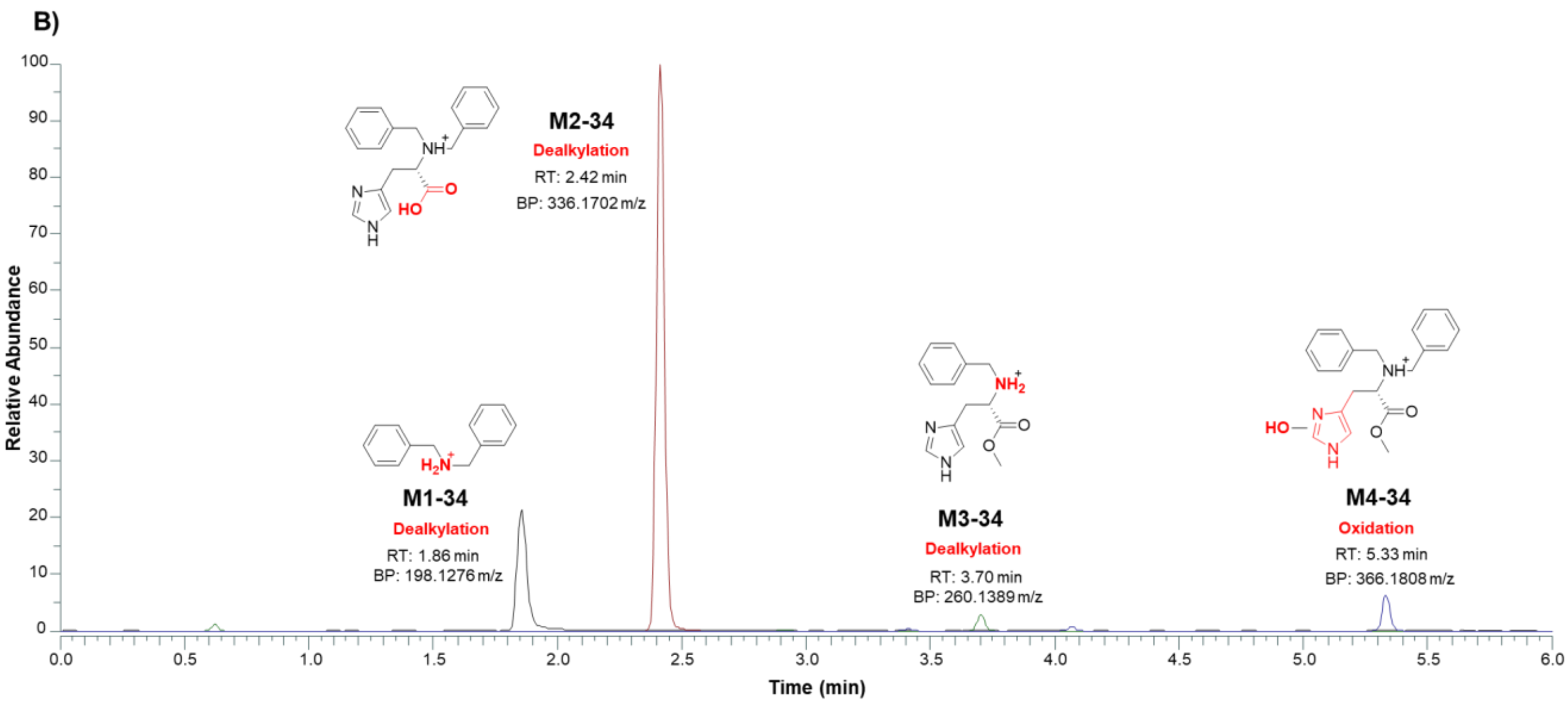
B)
M1-34
Dealkylation
RT: 1.86 min
BP: 198.1276 m/z
M2-34
Dealkylation
RT: 2.42 min
BP: 336.1702 m/z
M3-34
Dealkylation
RT: 3.70 min
BP: 260.1389 m/z
M4-34
Oxidation
RT: 5.33 min
BP: 366.1808 m/z
Relative Abundance
Time (min)

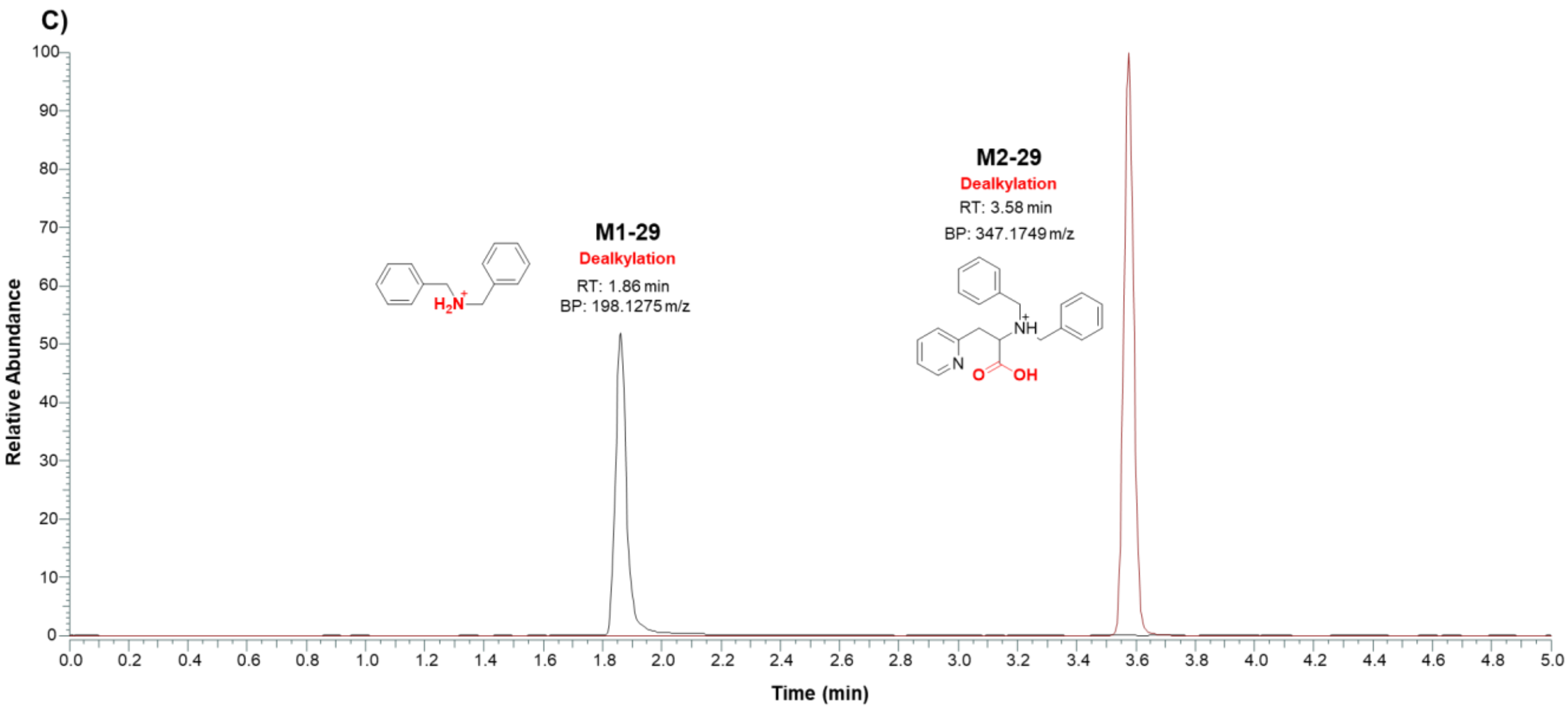
C)
M1-29
Dealkylation
RT: 1.86 min
BP: 198.1275 m/z
M2-29
Dealkylation
RT: 3.58 min
BP: 347.1749 m/z
Relative Abundance
Time (min)

D)

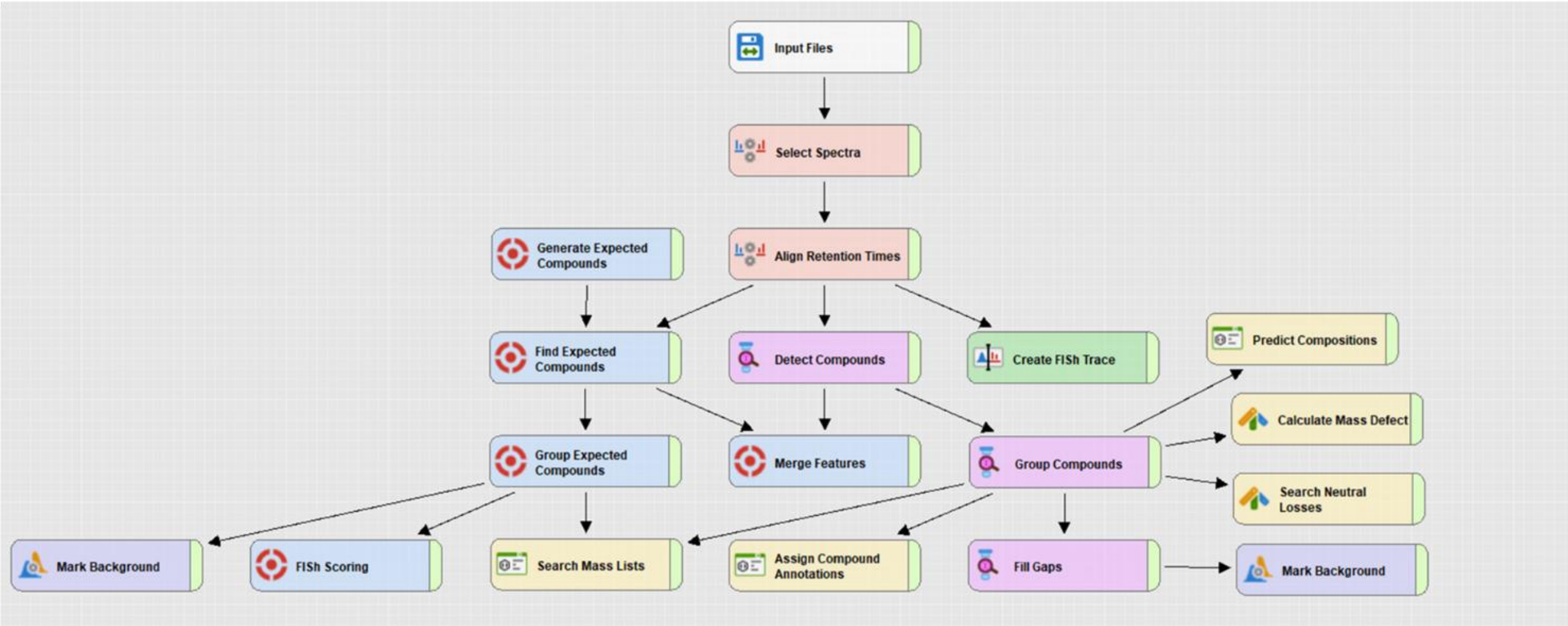

Figure S73: Identification of metabolites following microsomal incubation of test compounds. LC-HRMS/MS profiles of metabolic products from compounds **7** (**A**), **34** (**B**), and **29** (**C**). MetID was performed using a Vanquish™ UHPLC system coupled to an Orbitrap Exploris™ 120 mass spectrometer (Thermo Fisher). Chromatographic separation was achieved on a Kinetex® 2.6 µm EVO C18 column (100 × 2.1 mm) thermostated at 40 °C, using H_2O/ACN + 0.1% HCOOH (5-95% B over 10 min) at a flow rate of 0.4 mL·min^{-1}. Full MS scans (m/z 100–1500, 60000 res) and data-dependent MS/MS (15000 res, 30% HCD) were acquired in positive ion (HESI+) mode.
D) Data analysis was performed using Compound Discoverer™ 3.3 (Thermo Fisher Scientific, Waltham, MA, USA). MetID processing workflow included the following steps: i) retention time (RT) alignment across all samples; ii) Detection of expected metabolites derived from *in silico* predictions for the test compounds, with confirmation by isotope pattern matching; iii) grouping of detected compounds across samples, based on composition prediction, pattern scoring, neutral loss search, library matching, and mass list search; iv) application of FISh (Fragment Ion Search) scoring to all expected metabolites and their transformation products; v) annotation of fragmentation spectra with candidate structures and FISh scores; vi) prediction of elemental compositions for unknown compounds, gap filling across samples, and chemical background removal.

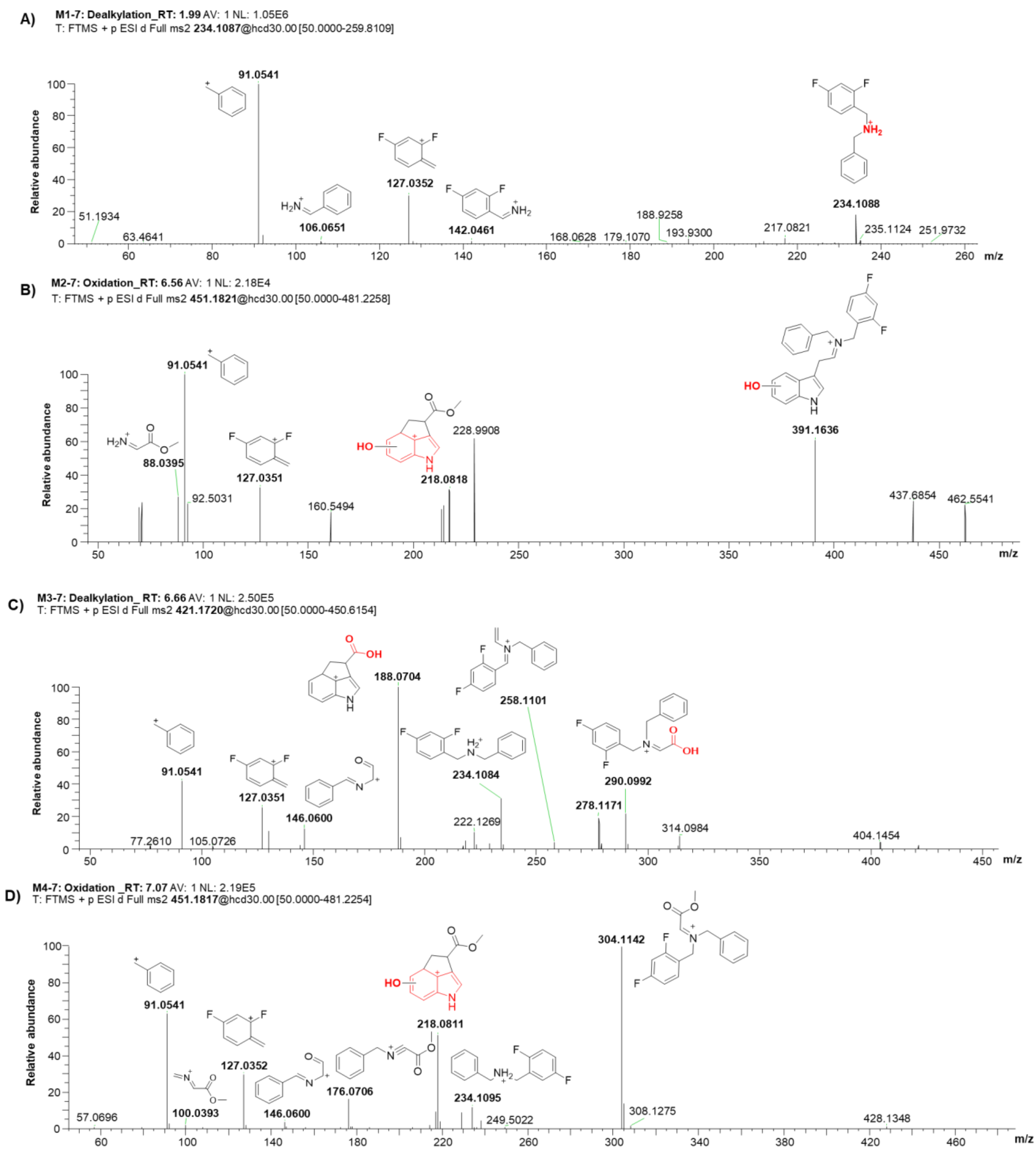

Figure S74: MS^2 spectra illustrating the proposed fragmentation pattern of metabolites derived from compound 7. M1-7 (**A**, *formula*: $C_{14}H_{13}F_2N$, *transformations*: dealkylation, *composition change*: -($C_{12}H_{11}NO_2$), *error*: -1.09 ppm); M2-7 (**B**, *formula*: $C_{26}H_{24}F_2N_2O_3$, *transformations*: oxidation, *composition change*: +(O), *error*: -1.88 ppm); M3-7 (**C**, *formula*: $C_{25}H_{22}F_2N_2O_2$, *transformations*: dealkylation, *composition change*: -(CH_2), *error*: -1.95 ppm); M4-7 (**D**, *formula*: $C_{26}H_{24}F_2N_2O_3$, *transformations*: oxidation, *composition change*: +(O), *error*: -1.57 ppm). Proposed structures and key fragmentations are highlighted in red.

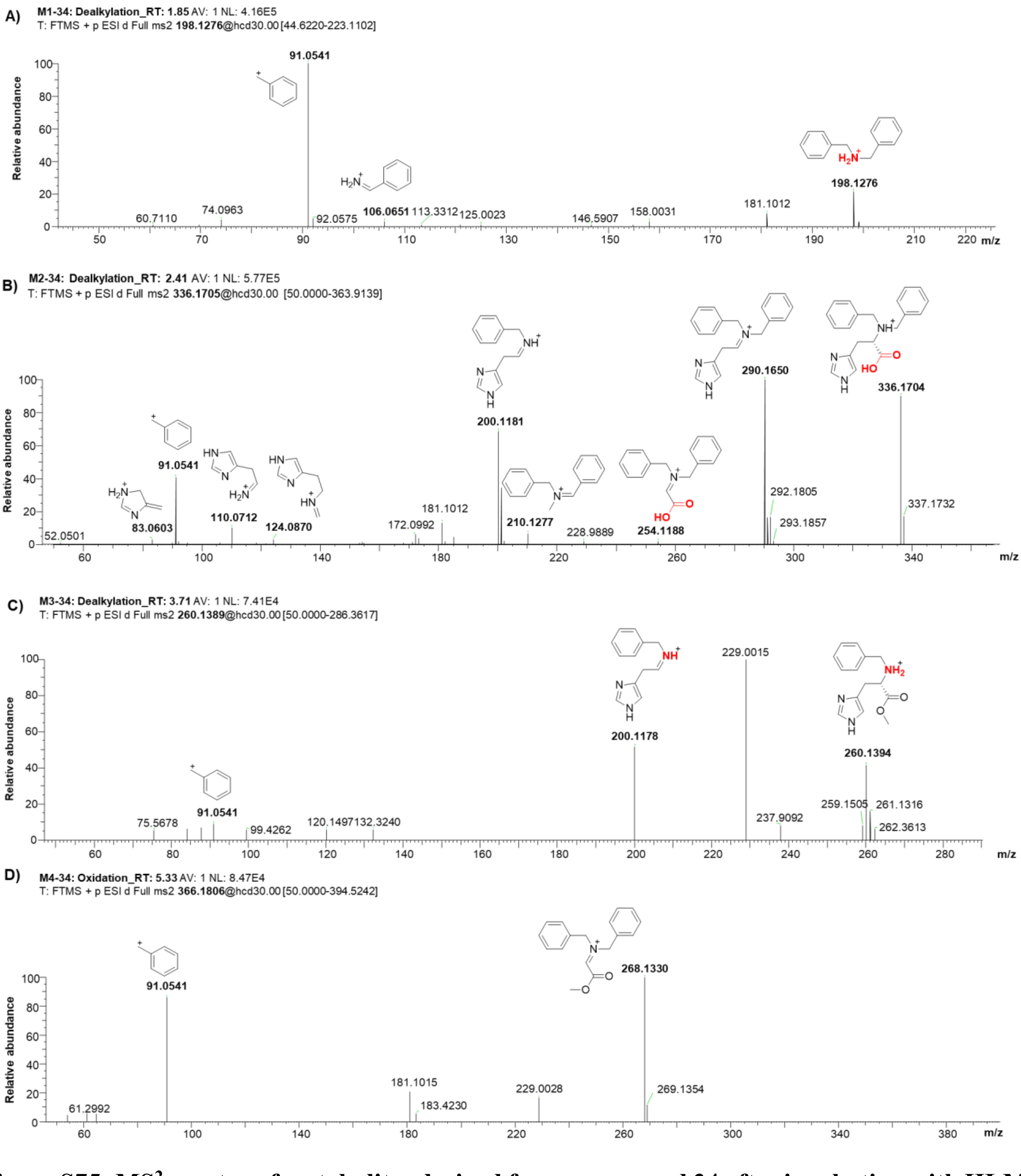

Figure S75: MS² spectra of metabolites derived from compound 34 after incubation with HLMs in the presence of NADPH. The characteristic fragment ions and the proposed fragmentation patterns are highlighted in red. M1-34 (**A**, *formula*: $C_{14}H_{15}N$, *transformations*: dealkylation, *composition change*: -($C_7H_8N_2O_2$), *error*: -0.76 ppm); M2-34 (**B**, *formula*: $C_{20}H_{21}N_3O_2$, *transformations*: dealkylation, *composition change*: -(CH_2), *error*: -1.45 ppm); M3-34 (**C**, *formula*: $C_{14}H_{17}N_3O_2$, *transformations*: dealkylation, *composition change*: -(C_7H_6), *error*: -2.06 ppm); M4-34 (**D**, *formula*: $C_{21}H_{23}N_3O_3$, *transformations*: oxidation, *composition change*: +(O), *error*: -1.36 ppm).

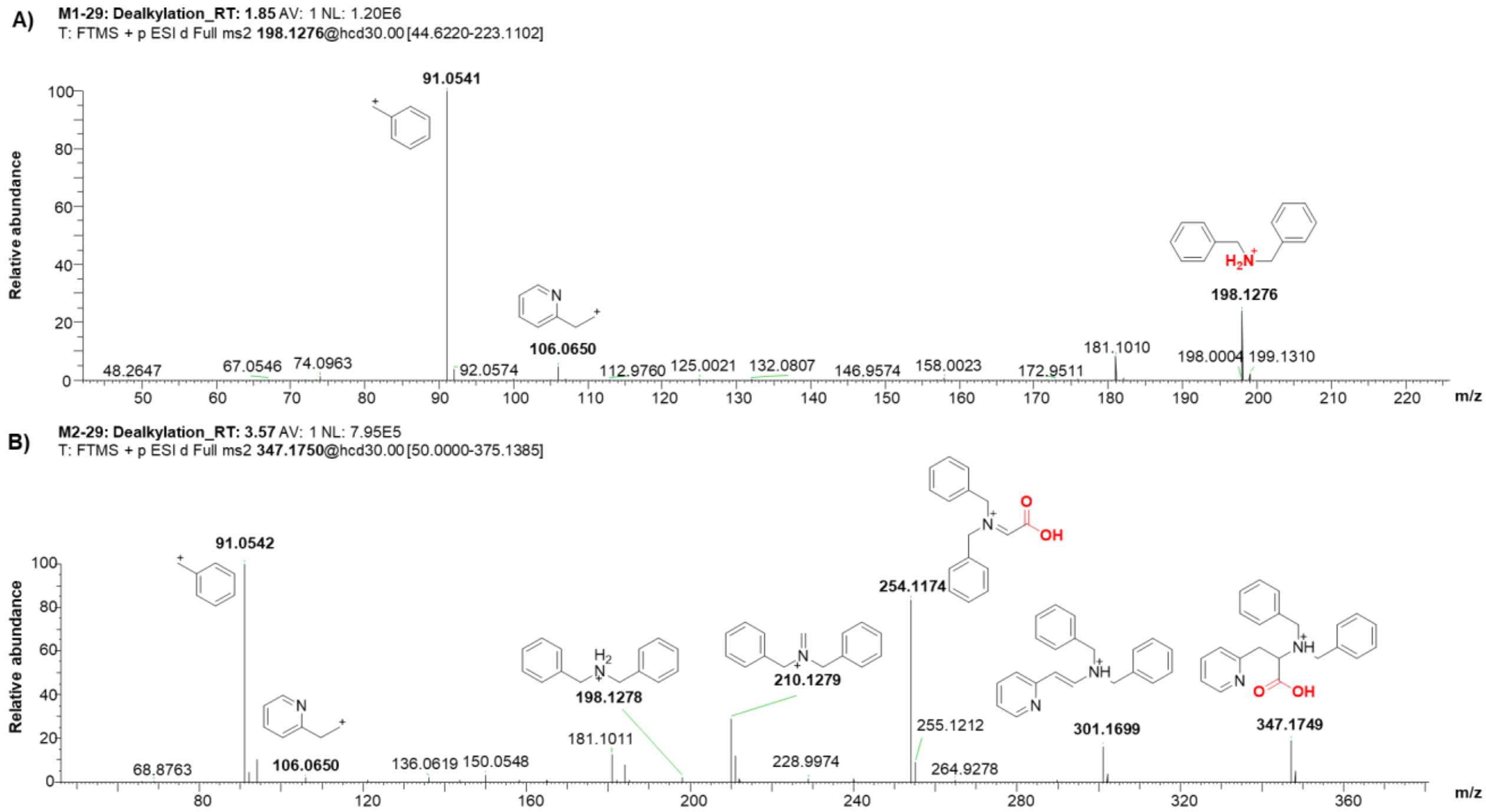

Figure S76: MS/MS spectra of HLM-generated metabolites of compound 29. M1-29 (**A**, *formula*: $C_{14}H_{15}N$, *transformations*: dealkylation, *composition change*: -($C_9H_9NO_2$), *error*: -0.81 ppm); M2-29 (**B**, *formula*: $C_{22}H_{22}N_2O_2$, *transformations*: dealkylation, *composition change*: -(CH_2), *error*: -1.89 ppm). Fragment ions and proposed fragmentation pathways highlighted in red.

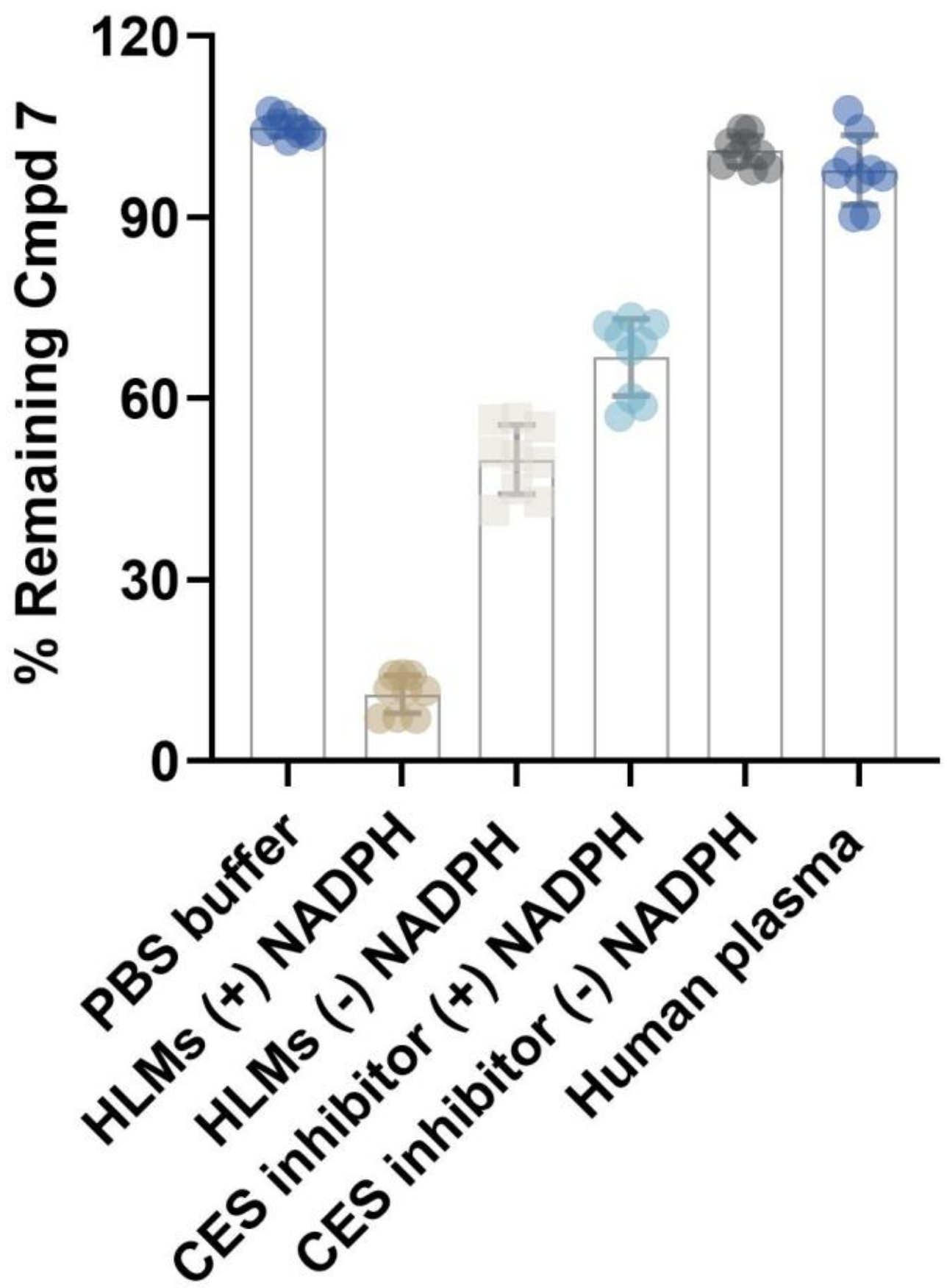

Figure S77: NADPH-dependent and -independent metabolic and plasma stability of compound 7. Percentage of parent compound remaining after 60 min incubation under different conditions: phosphate-buffered saline (PBS), human liver microsomes (HLMs) with or without NADPH, and HLMs pretreated with CES inhibitor phenylmethylsulfonyl fluoride (PMSF) in the presence or absence of NADPH. Human plasma stability was evaluated after 240 min of incubation. Data represent mean ± SD (n = 3).

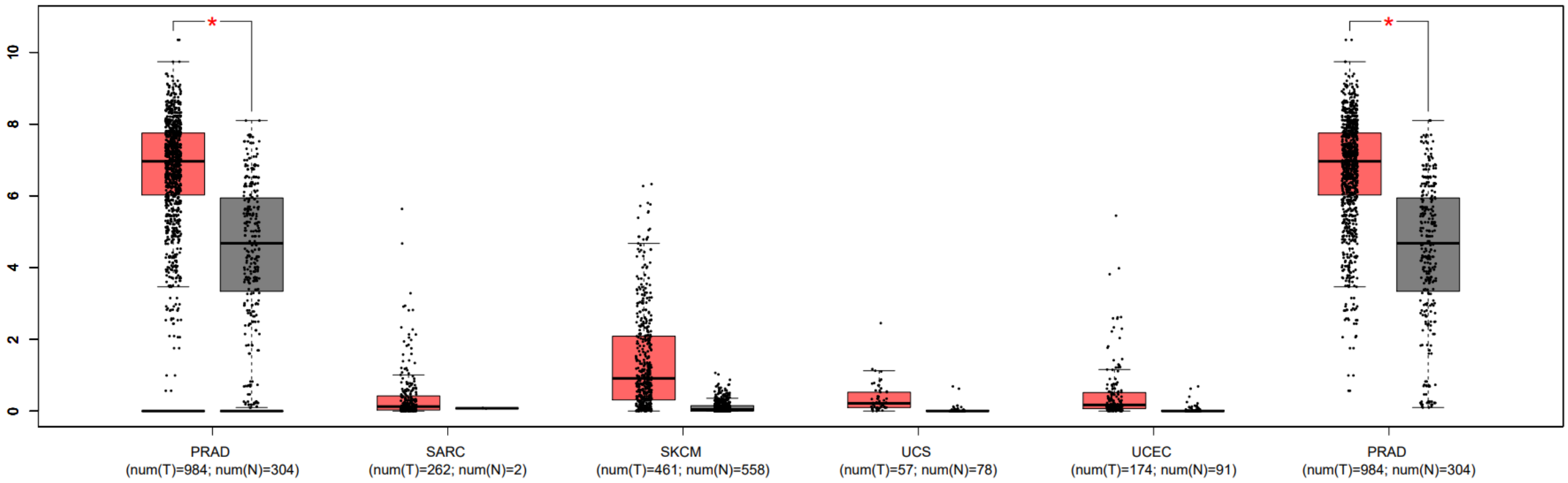

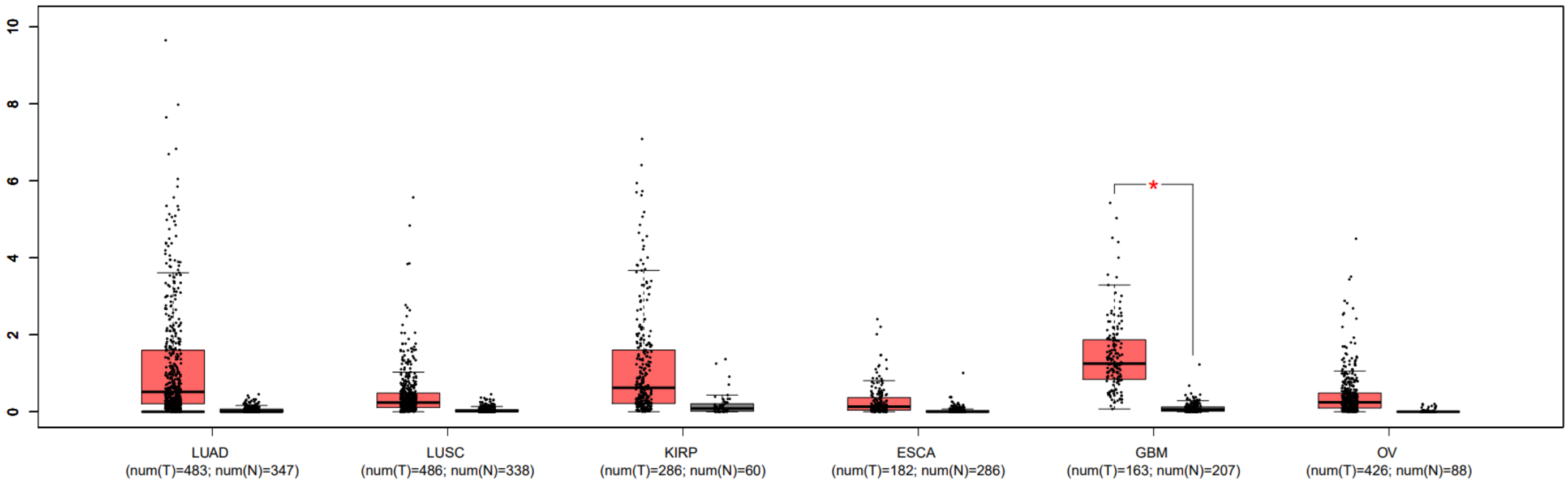

Figure S78: Differential mRNA expression profile of TRPM8 across different cancer type. Box plots illustrating the steady-state mRNA expression levels of TRPM8 in various human tumor tissues (red boxes) compared to their corresponding normal tissues (grey boxes). Expression data were obtained and analyzed via the GEPIA2 web server, utilizing harmonized RNA sequencing datasets from the TCGA and GTEx projects. The y-axis displays the transcript expression levels normalized as $LOG_2(TPM+1)$. Within each box, the transverse black line represents the median expression value. The exact number of tumor (T) and normal (N) samples analyzed for each respective cohort is indicated below the x-axis labels. Statistical significance was evaluated using one-way ANOVA, with red asterisks (*) denoting significant differential expression between tumor and normal groups. ($p<0.01$ $|LOG_2|FC>1$) **LUAD**: Lung Adenocarcinoma; **LUSC**: Lung Squamous Cell Carcinoma; **KIRP**: Kidney Renal Papillary Cell Carcinoma; **ESCA**: Esophageal Carcinoma; **GBM**: Glioblastoma **OV**: Ovarian Serous Cystadenocarcinoma; **PAAD**: Pancreatic Adenocarcinoma; **PRAD**: Prostate Adenocarcinoma; **SARC**: Sarcoma; **SKCM**: Skin Cutaneous Melanoma; **UCEC**: Uterine Corpus Endometrial Carcinoma; **UCS**: Uterine Carcinosarcoma

Table S3: Docking scores related to the selected poses of the compounds investigated through molecular docking.

Compounds	Docking score (kcal/mol)
**3**	-7.073
**5**	-9.559
**6**	-8.544
**7**	-8.752
**8**	-7.198
**9**	-9.454
**10**	-9.197
**12**	-8.611
**13**	-9.249
**23**	-8.864
**24**	-8.387
**25**	-5.903
**26**	-5.819
**27**	-7.970
**28**	-5.405
**29**	-6.777
**30**	-8.183
**31**	-6.068
**32**	-6.685
**34**	-7.269

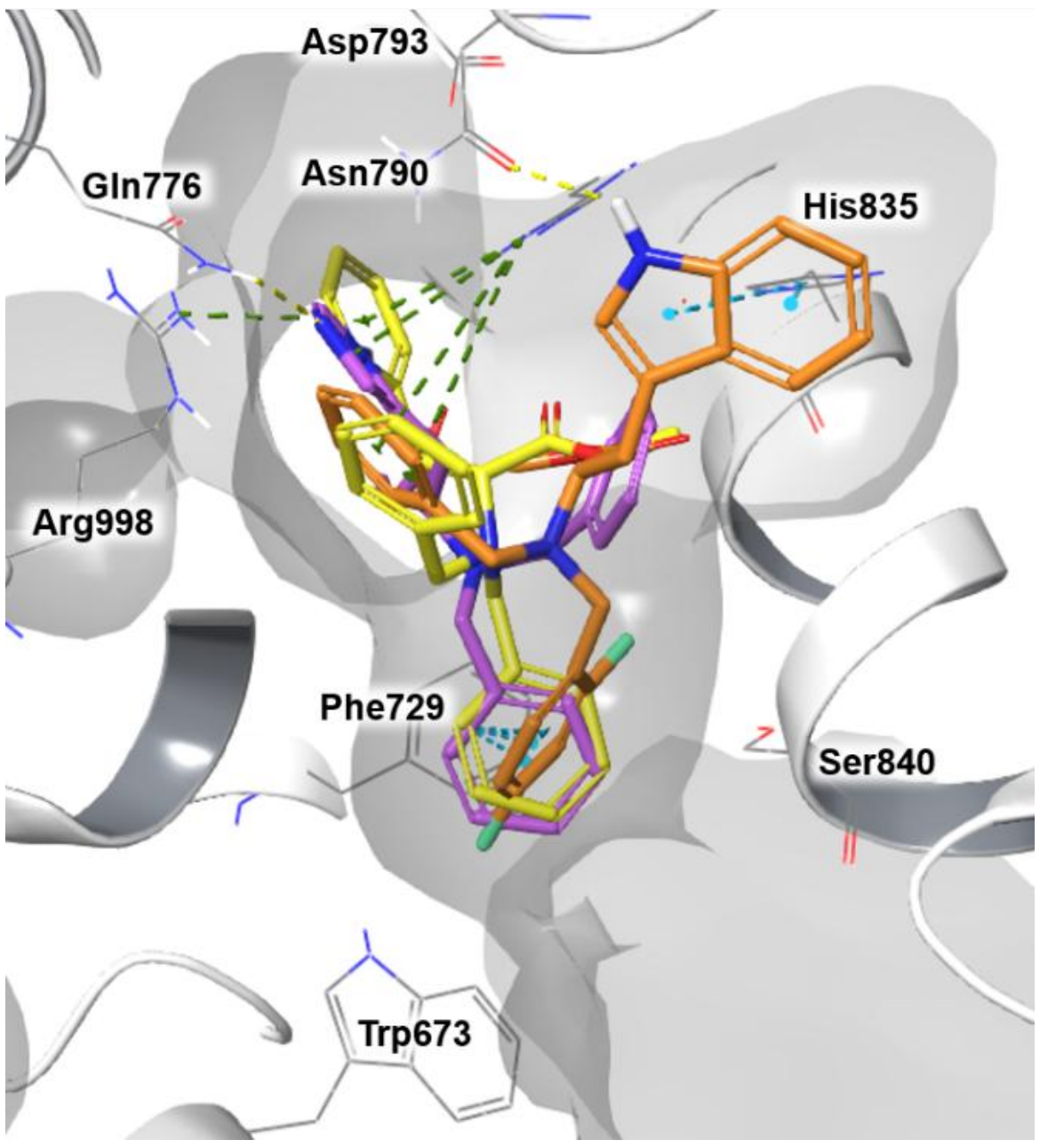

Figure S79. Superposition of the three most promising compounds emerged from the present study. The carbon atoms of compounds **7**, **29** and **34** are shown in orange red, yellow and faded plum, respectively.

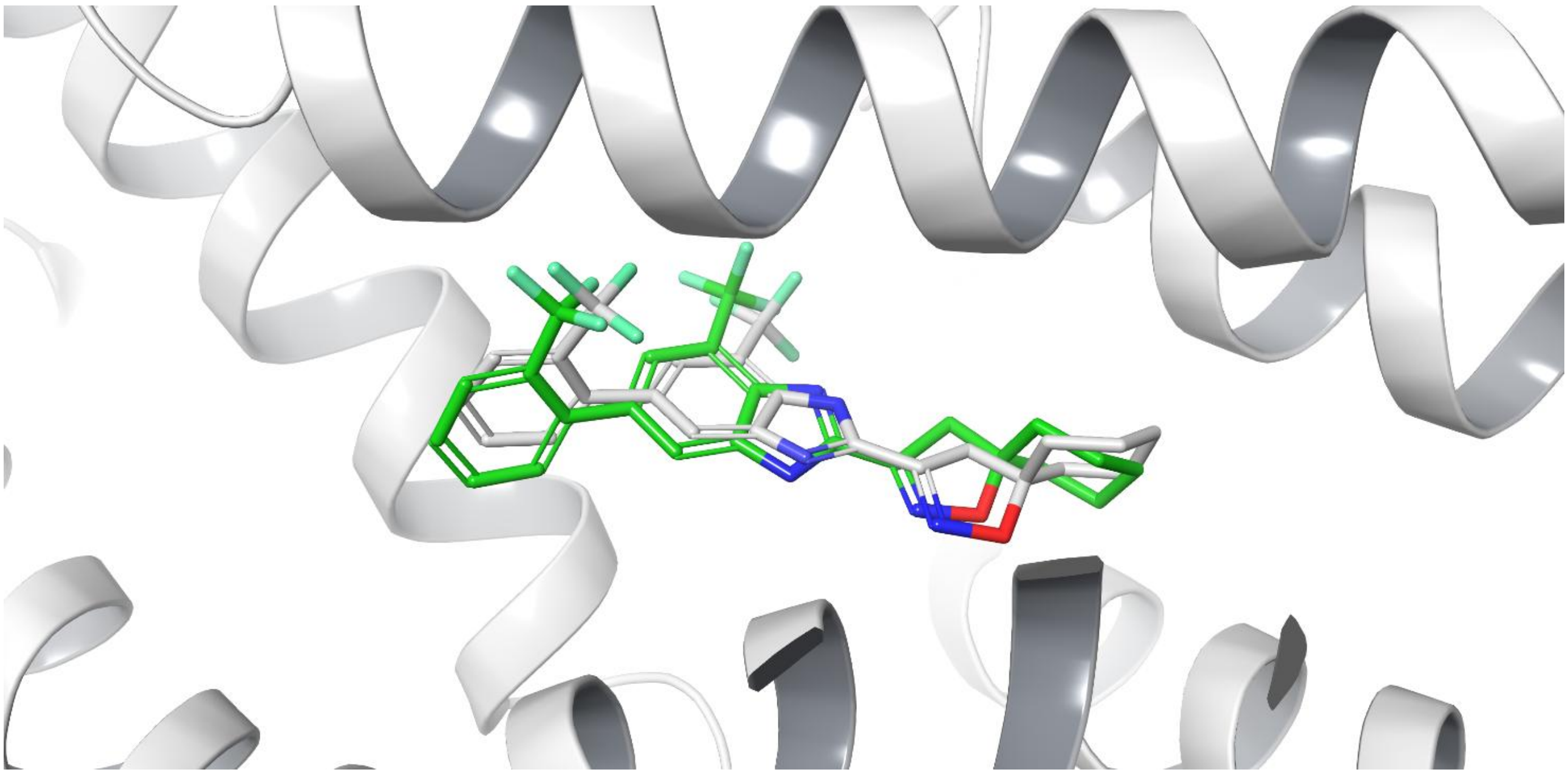

Figure S80. Superposition of the co-crystallized binding pose of TC-I 2014 (PDB ID: 6O72) (colored by atom type: C light grey, N blue, O red, F light green) and the pose obtained by redocking (colored by atom type: C light grey, N blue, O red, F light green). The high degree of overlap between the experimental and predicted binding modes, reflected by an RMSD of 0.94 Å (hydrogen atoms not considered in the computation of the RMSD), confirms the ability of the docking protocol to reliably reproduce the ligand binding orientation within the TRPM8 binding site.
